# Patient and Professional Perspectives on Long COVID: A Systematic Literature Review and Meta-Synthesis

**DOI:** 10.3390/ijerph22111620

**Published:** 2025-10-24

**Authors:** Sophia X. Sui, Lei Yu

**Affiliations:** 1 School of Psychology, The University of Adelaide, Adelaide, VIC 5005, Australia; 2 School of Northeast Asian Studies, Shandong University, Weihai 264209, China

**Keywords:** long COVID, post-COVID-19 condition, post-viral syndrome, qualitative meta-synthesis, patient experience, professional perspectives, stigma, pacing and rehabilitation, care coordination, mental health, health services

## Abstract

Background: Post-COVID-19 condition (‘long COVID’) involves fluctuating symptoms across multiple organ systems and disability or functional loss, which may be episodic, continuous, or permanent. Qualitative research is essential to capture lived experiences and explain how social and health system contexts may influence improvement, recovery, and service use. We synthesised perspectives from people living with long COVID and healthcare professionals to inform service design and policy. Methods: We conducted a systematic review and qualitative meta-synthesis. MEDLINE, Embase, PsycINFO, CINAHL, Scopus, and Web of Science were searched for studies published between 1 January 2020 and 19 August 2025. Eligible studies reported qualitative data from adults with long COVID (≥12 weeks after acute infection) and/or healthcare professionals in any setting. We excluded non-qualitative, non-primary, or non-English reports. Two reviewers independently screened, extracted, and appraised studies using the Critical Appraisal Skills Programme checklist. Data were synthesised thematically. The protocol was registered with the Open Science Framework. Findings: Of 1544 records screened, 49 studies met the inclusion criteria: 41 involving patients, two involving professionals, and six involving both. Eight patient themes (including symptom burden, identity disruption and stigma) and four professional themes (including recognition, care coordination and holistic care models) were identified. Recognition emerged as a cross-cutting mechanism: validation and consistent pacing guidance facilitated engagement and safer activity, whereas invalidation and inconsistent advice were associated with distress, avoidance, and disengagement. Trajectories showed gradual expansion of multidisciplinary care models, but major capacity and equity gaps persisted. Most studies had low methodological concerns, although heterogeneity in populations and settings was substantial. Interpretation: Long COVID is a chronic, biological condition that also intersects with social and psychological dimensions, and may present with episodic, continuous, or progressive trajectories. Healthcare services must prioritise early validation, provide consistent pacing and relapse prevention guidance, expand access to multidisciplinary and peer-supported rehabilitation, integrate mental healthcare, strengthen coordinated pathways, and support graded return to work. Explicit attention to equity is required to avoid widening disparities.

## 1. Panel: Research in Context

### 1.1. Evidence Before This Study

We searched MEDLINE, Embase, PsycINFO, CINAHL, Scopus, and Web of Science (1 January 2020–19 August 2025) for English-language studies using terms for long COVID, qualitative methods, and patient or professional perspectives. Eligible designs were primary qualitative or mixed methods with extractable qualitative data. Earlier syntheses, while valuable, often included a smaller number of studies and participants, were dominated by patient narratives, heavily UK-centred, and conducted before widespread service roll-out, which limited their generalisability.

### 1.2. Added Value of This Study

This review is the first to integrate patient and professional perspectives within a single analytic frame. By systematically synthesising qualitative data, including author-defined themes and direct quotations, our analysis provides a rich and in-depth account of lived experience and professional challenges. Situating these findings against evolving service and policy developments, it maps experiential and system trajectories from 2021 to 2025. Recognition, consistent guidance (especially on pacing), and coordinated care emerged as cross-cutting mechanisms shaping engagement and recovery.

### 1.3. Implications of All the Available Evidence

Health systems must embed validation at first contact, provide clear pacing and relapse guidance, expand multidisciplinary and peer-supported rehabilitation, integrate mental-healthcare, strengthen coordinated pathways anchored in primary care, and enable graded return-to-work. Explicit focus on equity is essential to prevent widening disparities. Continued qualitative and mixed-methods research will be needed to monitor long-term trajectories and evaluate the implementation of these recommendations for public health impact.

## 2. Introduction

Post-COVID-19 condition (‘long COVID’) is defined by the World Health Organization (WHO) as the presence of new, returning, or ongoing symptoms ≥ 3 months after acute severe acute respiratory syndrome coronavirus 2 infection, lasting for at least 2 months and not attributable to alternative diagnoses [1]. In 2024, the US National Academies issued a consensus definition emphasising long COVID as a chronic, systemic disease state with relapsing–remitting or progressive trajectories [2].

In addition to symptom-based definitions, biomedical research has highlighted potential pathophysiological mechanisms underlying long COVID, including immune dysregulation, autonomic nervous system dysfunction, endothelial and microvascular abnormalities, and organ-specific sequelae such as cardiac, pulmonary, and neurological involvement [3,4,5].

Although prevalence estimates vary widely, for example, a comprehensive prospective meta-analysis found that more than 50% of COVID-19 survivors experienced at least one symptom more than a year after infection [6]. Other studies report lower estimates: for instance, a systematic review found ~45% had unresolved symptoms at ~4 months [7], while another meta-analysis reported that 10–20% of survivors had developed long COVID [8]. This represents a substantial absolute burden. WHO continues to highlight long COVID as a global health priority, encouraging national authorities to plan and budget for multidisciplinary care programs and ensure equitable access to relevant therapies [9]. Beyond direct morbidity, long COVID has wide-ranging health-system, economic, and societal impacts, prompting calls for coordinated research and policy responses [10].

Clinical guidance now recognises the multisystem nature of long COVID, which may follow episodic, relapsing–remitting, continuous, or progressive trajectories, and highlights the need for integrated, patient-centred pathways [11,12]. The United Kingdom’s National Institute for Health and Care Excellence living guideline outlines the identification, assessment, and management of long COVID, including for children and young people [13]. In parallel, the National Health Service (NHS) England has issued commissioning guidance specifying multidisciplinary services and referral pathways [14]. Internationally, rehabilitation and activity advice remain debated, especially in relation to post-exertional malaise (PEM). Current guidelines (e.g., NICE 2021; CDC 2022) [15,16] caution against graded exercise therapy where PEM is present, as exertion can worsen symptoms and trigger clinical relapse. Instead, contemporary recommendations stress careful screening for PEM, pacing and energy-management strategies, and individualised, safety-netted rehabilitation approaches [17,18]. These evolving policies and ongoing uncertainties underscore the importance of understanding both patient and professional perspectives to inform service design [17,18].

Qualitative evidence has been central in documenting the lived experience of long COVID, including stigma and invalidation, fluctuating symptoms and functional loss, information gaps, and difficulties navigating fragmented services [19]. Early syntheses focused predominantly on patient narratives, were heavily UK-based, and predated widespread service roll-out [20]. More recent qualitative research and service evaluations have incorporated provider perspectives, highlighting fragmentation, role ambiguity, and the challenges of implementing coordinated long COVID care pathways [21,22]. Additionally, interprofessional collaboration literature from non–COVID settings underscores analogous systemic challenges in complex care transitions [23,24]. However, few reviews integrate patient and professional accounts within a single analytic frame, or trace how experiences and service provision co-evolved across the pandemic and post-pandemic periods.

This systematic review and meta-synthesis address that gap. Our aims were to (1) characterise common experiential domains across settings; (2) identify mechanisms through which services shaped engagement, coping, and recovery; and (3) map trajectories over time to inform clinical pathways and policy. By preserving verbatim quotations alongside higher-order interpretation, and situating findings against contemporary guidance, we sought to generate actionable insights for equitable, integrated long-COVID care.

## 3. Methods

### 3.1. Search Strategy and Selection Criteria

We conducted a systematic review and qualitative meta-synthesis of studies reporting the experiences of people with long COVID and/or health professionals caring for them. Eligible studies were primary qualitative investigations or qualitative components of mixed-methods designs with extractable findings (author-reported themes, subthemes, and/or verbatim quotations). The protocol for this qualitative meta-synthesis was prospectively registered with the Open Science Framework (registration DOI: https://doi.org/10.17605/OSF.IO/TVFKC). The registration outlines the study objectives, inclusion criteria, and synthesis methods.

We accepted a broad range of analytic approaches, including thematic and reflexive thematic analysis, grounded theory, interpretative phenomenological analysis, framework or content analysis, and narrative inquiry.

Reports were eligible if published in English between 1 January 2020 and 19 August 2025. We placed no restrictions on setting (community, primary care, hospital, rehabilitation, workplace, online, or hybrid) and did not impose a minimum sample size, as adequacy was assessed interpretively.

Exclusion criteria included quantitative-only studies; non-primary reports (editorials, commentaries, protocols); conference abstracts without full articles; studies of acute COVID-19 only (≤12 weeks); studies of generic sequelae without a long COVID focus; studies not involving long COVID patients or professionals caring for them; and studies limited to biological, imaging, pathophysiological, or tool-validation outcomes without experiential findings. Additional exclusions were non-English language publications outside the specified date range, preprints or grey literature, and duplicates. Full operationalised eligibility rules, adjudication procedures, and Preferred Reporting Items for Systematic Reviews and Meta-Analyses (PRISMA)-aligned exclusion categories are provided in Supplementary File S1 (Supplementary Materials).

We searched MEDLINE, Embase, PsycINFO, CINAHL, Scopus, and Web of Science, combining controlled vocabulary (MeSH/Emtree) with free-text terms for long COVID, qualitative methods, and patient/professional experiences. Reproducible search strategies are reported in Supplementary File S2. We also hand-searched reference lists of included studies and consulted grey-literature sources (Supplementary File S3).

**Figure 1:**
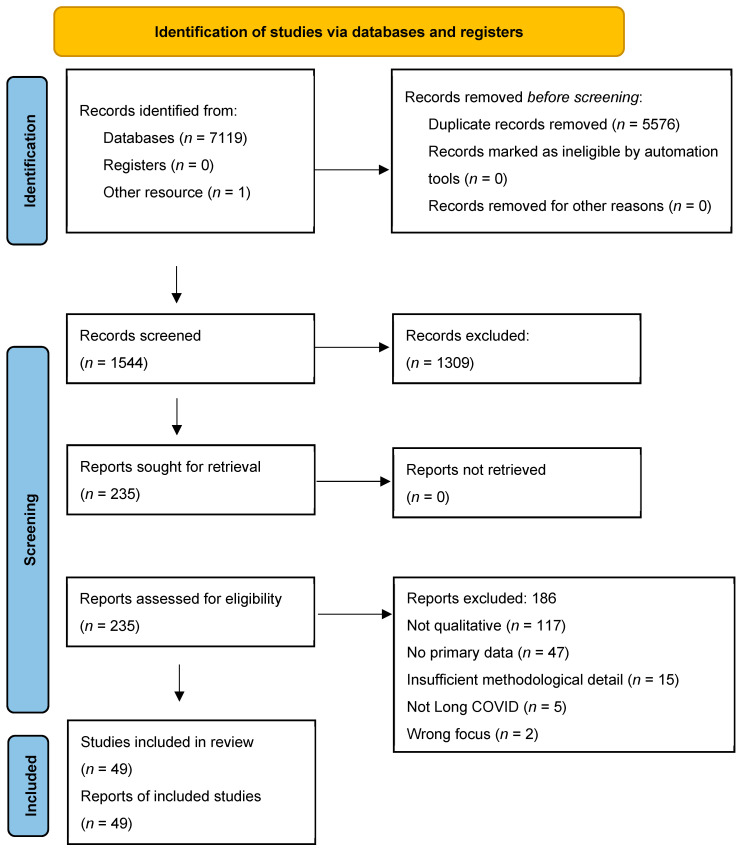
Study selection process (PRISMA 2020). Database searches identified 7119 records, with one additional record from other sources. After removal of 5576 duplicates, 1544 titles and abstracts were screened, leading to 235 full-text articles assessed for eligibility. Of these, 186 were excluded for reasons including not long COVID, not qualitative, no primary data, insufficient methodological detail, or wrong population/focus. In total, 49 unique studies were included in the synthesis.

Records were imported into EndNote (v21) for de-duplication. Two reviewers independently screened titles, abstracts, and full texts in Rayyan, recording exclusion reasons. Disagreements were resolved through discussion. Screening results are summarised in the PRISMA 2020 flow diagram (Figure 1), with detailed exclusion reasons. Two reviewers independently extracted: study identifiers; country and setting; sampling, data collection and analytic approaches; participant characteristics; and qualitative findings (themes, subthemes, quotations).

Methodological quality was appraised using the Critical Appraisal Skills Programme (CASP) qualitative checklist [25]. No study was excluded based on quality. CASP judgements (Supplementary File S4) informed the judgements (Supplementary File S4) informed the Grading of Recommendations Assessment, Development and Evaluation–Confidence in the Evidence from Reviews of Qualitative research (GRADE-CERQual) ratings across the four domains of methodological limitations, coherence, adequacy, and relevance, across the four domains of methodological limitations, coherence, adequacy, and relevance [26]. A Summary of Qualitative Findings (SoQF) and CERQual justifications are also reported in Supplementary File S5.

### 3.2. Data Synthesis

We conducted a thematic meta-synthesis in NVivo (v15). Author-reported themes and subthemes were coded inductively, then iteratively grouped into higher-order domains via constant comparison. Patient and professional corpora were synthesised separately before being compared to identify convergences and divergences. Verbatim quotations were preserved to retain participant voices.

We also mapped temporal trajectories (2021–2025) to examine how contextual mechanisms shaped engagement, coping, and access to care. Transparency was enhanced through the provision of a codebook excerpt (Supplementary File S6) and reflexive notes (Supplementary File S7). Rigour was supported by dual reviewer involvement at all stages, regular multidisciplinary reflexive discussions, and maintenance of brief reflexive memos.

The review followed Enhancing Transparency in Reporting the Synthesis of Qualitative Research (ENTREQ) guidance [27] and is reported according to PRISMA 2020 [28]. Ethics approval was not required.

## 4. Results

### 4.1. Study Selection

Database searches yielded 7119 records, with one additional record from other sources. After removing 5576 duplicates, 1544 records were screened at the title/abstract stage. Of these, 235 full-text articles were assessed, with 186 excluded (reasons: not long COVID; not qualitative; no primary data; insufficient methods detail; wrong population or focus). Full exclusion reasons are provided in Supplementary File S1. In total, 49 unique studies met the inclusion criteria and were synthesised (Figure 1).

### 4.2. Study Characteristics

Table 1 summarises core study characteristics, with the full detailed table provided in Supplementary File S8. The included studies spanned community, primary care, hospital, rehabilitation, and workplace settings, sampling people with long COVID, health professionals, and, in some cases, caregivers. Participant numbers and demographics were variably reported. Data were not pooled to avoid double-counting when qualitative components were embedded within mixed-methods studies. Quality appraisal outcomes are summarised in Supplementary File S5, and extended quotations with full citations are in Supplementary File S9. For clarity, we also provide definitions of technical qualitative methodologies in a glossary (Supplementary File S10).

**Figure 2:**
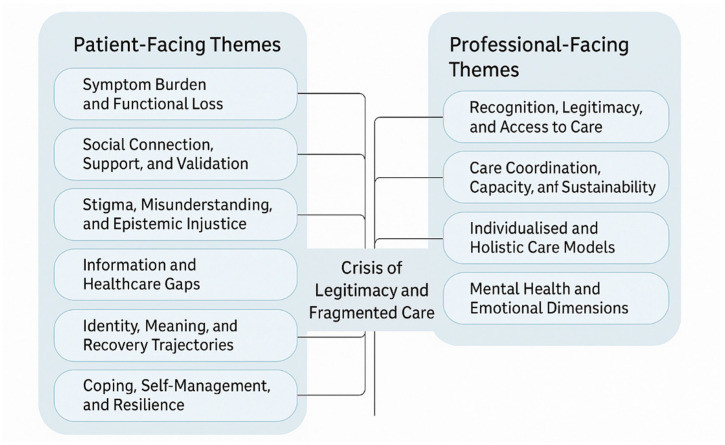
Patient and professional trajectories (2023–2025). Findings from a meta-synthesis of qualitative studies on long COVID, identifying key patient and professional themes.

The findings are structured around eight patient-facing themes and four professional-facing themes (Figure 2), with verbatim quotations and thematic aggregation grounded in the original research. Each section details the prevalence of the themes across the identified literature, demonstrating a shared, yet distinct, journey for patients and professionals defined by a crisis of legitimacy and fragmented care.

## 5. The Lived Experience of Long COVID: A Crisis of Legitimacy

### 5.1. Symptom Burden and Functional Loss

The qualitative evidence consistently portrays long COVID as a profoundly unpredictable and functionally limiting condition, with this theme reported in 38 studies spanning 63 author-generated themes. Patients’ narratives are dominated by a new, altered reality, where even the most basic activities require a disproportionate and exhausting effort [74]. This functional impairment is further compounded by the cyclical and unpredictable nature of symptoms, which creates a state of perpetual uncertainty and fear of relapse [75]. The illness’s “rollercoaster ride” of fluctuating health, with unpredictable episodes and severity, contributes to significant physical and cognitive burdens [68]. The pervasive and often invisible toll of the condition, encompassing physical complaints like chest pain and tightness [76], underscores a heavy burden that is largely unseen by others [45].

### 5.2. Social Connection, Support, and Validation

Reported in 32 articles and aggregating 26 author-generated themes, the transformative role of social connection emerges as a powerful counter-narrative to the isolation and invalidation experienced by many. Peer support networks, particularly online communities, are described as a critical “lifeline” and a “beacon of light” that fills the void left by inadequate professional care [70]. This communication provides crucial emotional and informational support, validating patients’ experiences and reassuring them that they are “not the only one” struggling with similar symptoms [70]. Furthermore, the therapeutic value of structured, professionally guided group interventions is evident, with participants describing feeling “released out of the cage… so free” in these supportive environments [69].

### 5.3. Stigma, Misunderstanding, and Epistemic Injustice

This theme was reported across 17 author-generated themes in 18 articles, demonstrating the pervasive impact of invalidation and dismissal. A central finding is the concept of epistemic injustice, where patients’ knowledge and testimony about their illness are unjustly dismissed due to a lack of institutional understanding [67]. Narratives highlighted that their “patient status often lacked comparable authenticity in others’ eyes” and that they encountered a lack of recognition and understanding of their ongoing need to recover [32]. This dismissal was often layered with pre-existing biases, as women disproportionately reported symptom trivialization by healthcare professionals [42]. These negative experiences eroded trust in the healthcare system and reinforced patients’ feelings of frustration and abandonment, while conversely, even basic compassion from a provider was deeply valued [56].

### 5.4. Information, Knowledge and Health Literacy

The search for reliable information and consistent care was a significant burden, reported in 43 studies across 48 author-generated themes. Patients described profound frustration navigating diagnosis within a disconnected system, noting, “The GP … said it is probably [Long Covid], but there’s no research on it” [35]. Many also felt isolated and ill-equipped to make sense of medical information—one participant admitted that “…the common person can’t do that [critically appraise medical journals]”, while another shared how ambiguous advice like “don’t push it” did little to guide recovery [75]. Systemic gaps further undermined their ability to access comprehensive care: one participant lamented, “The dream is… one place you go, and they coordinate all the care”, while others described being turned away for not having a positive COVID test or being offered treatments only after persistence [41]. One expressed relief when clinicians embraced exploratory treatment: “I’m willing to try… and my doctors at [telehealth clinic] are willing to try” [41].

### 5.5. Identity, Meaning, and Recovery Trajectories

Reported in 14 studies across 26 author-generated themes, the long COVID experience is described as a life-altering event that necessitates a profound shift in identity. Participants recounted having to adapt to an “altered life” [58,75], with a severe sense of loss in their social roles, capabilities, and pre-illness sense of self [42]. The recovery journey was consistently described as non-linear and unpredictable [32], with many struggling to reconcile their former identity with their new [69], post-COVID reality [42]. This process of making meaning involved making sense of their condition and accepting new limitations as they adjusted to a new normal [56].

### 5.6. Work, Finances, and Role Changes

The extensive disruptions to employment, finances, and social roles were a prominent theme reported in 22 studies across 25 author-generated themes. Physical and cognitive symptoms often curtailed participants’ ability to work [45,61], with many describing the challenge of returning to employment due to the relapsing-remitting and unpredictable nature of their symptoms [63,71]. This resulted in significant financial impact, including pay cuts and job loss [51,56], noting “I was losing my career, I was losing everything.” [42], which compounded the stress and highlighted the need for more robust support systems at the intersection of health and employment [39,66]

### 5.7. Coping, Self-Management, and Resilience

In response to the illness’s uncertainty and fluctuating symptoms, participants developed a spectrum of coping strategies, reported in 26 qualitative studies across 25 author-generated themes. Self-management, often patient-generated, emerged as a key strategy for navigating daily life [57]. This included developing a greater self-awareness through symptom monitoring and engaging in “most effective ways” to manage their condition [51]. The importance of pacing and physical therapy was highlighted as a way to facilitate stable improvements and counteract symptoms [36,53].

### 5.8. Healthcare Navigation, Access and System Gaps

Reported across 35 qualitative studies with 57 author-generated themes, patients detailed significant barriers to care. Patients described major barriers in navigating fragmented systems, from long waits: “With the cardiologist, you call ‘come in half a year’… I find that alarming in Germany” [53]. Referral bottlenecks and repetitive retelling of symptoms further compounded exhaustion [57], while some felt unsupported altogether [74]. Structural challenges were also pronounced. One patient described care as disjointed and exhausting to navigate: “They didn’t interact with each other… I had to bring all my research to people myself… and I’m already tired and sick” [41]. Meanwhile, medication costs were prohibitive: “The medication was about $800… we couldn’t afford it” (USA) [54]. Despite these barriers, some participants reported positive support: “Thank God I have a GP… who supports me 100%” [53] though such experiences were exceptions rather than the norm. Notably, references to medication appeared almost exclusively in patient-perspective studies, with professionals rarely discussing pharmacological management in the included literature. Despite these barriers, some participants reported positive support. 

## 6. Professional Perspectives: Navigating a New Frontier

### 6.1. Recognition, Legitimacy, and Access to Care

Healthcare professionals grappled with the dual challenges of diagnostic uncertainty and a lack of established care pathways. The significant information vacuum within the healthcare system forced a reliance on patient-generated information, with one general practitioner admitting, “I was relying on my patients… to tell me what the Long COVID clinic is like and come back to me. And then I can tell the next person” [44]. Access to care was further hindered by referral confusion and a reluctance to promote services due to capacity issues [57], resulting in patients “getting stuck in the system” [57].

### 6.2. Care Coordination, Capacity, and Sustainability

The systemic and structural barriers to effective long COVID care emerge from a healthcare system already overstretched by sustained pressures [37]. Duncan et al. (2023) reflect on how complex and fragmented the organisational delivery of community rehabilitation is, with multiple challenges to service coordination and accessibility [57], noting that services often operated like “cogs working in different directions” [44], which led to significant delays and patient frustration.

### 6.3. Individualised and Holistic Care Models

In response to these systemic challenges, professionals are increasingly advocating for integrated and person-centred care models. There is a broad consensus that a one-size-fits-all approach is ineffective for a condition as heterogeneous as long COVID, with clinicians emphasising that “individuality is absolutely essential” [21]. Emerging models advocate for a multidisciplinary approach [37] that tailors care to the unique needs of each patient, with references in several studies to physical rehabilitation interventions and psychoeducation.

### 6.4. Mental Health and Emotional Dimensions

From a professional standpoint, addressing the psychological and emotional burden of long COVID is a critical component of care, noting that “we’ve got them not experiencing support from their family, friends, and most importantly, their healthcare team. So, a lot of them are going through being gaslit about their symptoms and not having a lot of support. So, that adds to mental health strains.” [21]. Clinicians recognised the need for holistic supports, including psychoeducation, that provide patients with foundational knowledge about their condition and self-management frameworks focusing on nutrition, sleep, and activity balance [21]. These approaches were described in the included studies as ways to empower patients and help them navigate the psychological complexities of their illness.

## 7. The Convergence of Patient and Professional Trajectories

**Figure 3:**
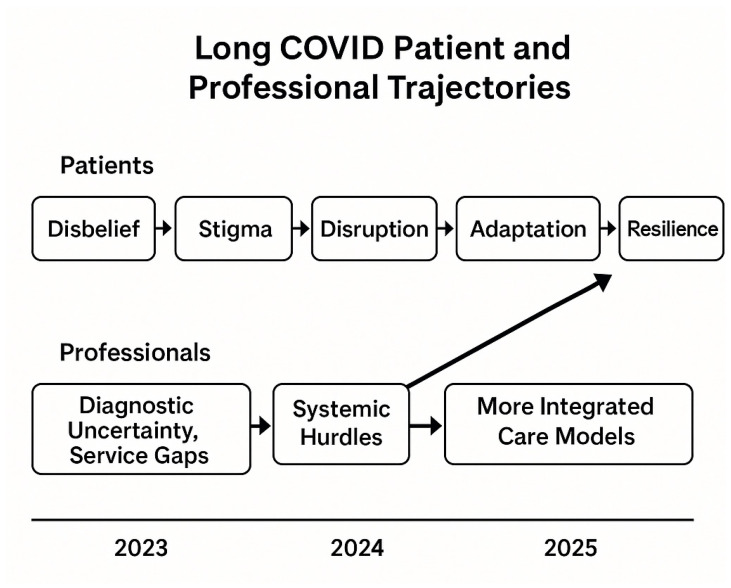
Patient and professional trajectories (2023–2025). Parallel pathways illustrate how patients transitioned from disbelief and disruption to resilience, while professionals progressed from uncertainty and service gaps toward integrated care models, converging on recognition and coordinated care.

Figure 3 maps the evolving trajectories of patients and professionals in relation to Long COVID between 2023 and 2025. Patients moved from disbelief and stigma, through disruption and adaptation, toward resilience. In parallel, professionals shifted from diagnostic uncertainty and fragmented service gaps to facing systemic hurdles, ultimately progressing toward more integrated models of care. Their convergence by 2025 highlights the interdependence of lived adaptation and professional recognition in shaping sustainable pathways of support.

Beyond this shared trajectory, the analysis of patient and professional narratives reveals a striking convergence of experience. Early encounters were often characterised by a wide gulf of understanding: patients confronted invalidation, dismissal, and profound physical debilitation [65,74], while professionals struggled with diagnostic uncertainty and fragmented service delivery [57]. Yet over time, both groups demonstrated a remarkable adaptive capacity.

Patients, faced with systemic barriers and ongoing relapses, developed pragmatic self-management strategies, including pacing and graded activity despite limited and inconsistent advice [75]. Peer networks also provided critical validation, offering both psychological support and practical guidance [74]. These patient-led innovations informed and, in many cases, pressured professional communities to reconsider long COVID not as a transient issue but as a chronic, relapsing condition requiring structured responses.

Professionals, in turn, have shifted from uncertainty to advocacy, emphasising the need for integrated, evidence-based, and person-centred care models. As Fang et al. (2024) [44] illustrate, fragmented services often operate like “cogs working in different directions,” but growing recognition of these challenges has led to stronger calls for coordination and sustainability. This mutual adaptation underscores that long COVID is not solely a biomedical condition but a complex social and systemic challenge. Its resolution requires deliberate collaboration: rebuilding trust, legitimising patient experiences, and reconfiguring health systems to respond effectively to the realities of chronic illness.

## 8. Discussion

### 8.1. Summary of Key Findings

This synthesis of 49 qualitative studies provides a comprehensive, multifaceted account of the long COVID experience, revealing a profound convergence between patient narratives and professional challenges. We identified eight patient-facing themes, ranging from the pervasive Symptom Burden and Functional Loss to Healthcare Navigation, Access and System Gaps. The patient journey is defined by a central crisis of recognition and validation, navigated through self-management, peer support, and a non-linear recovery trajectory. Concurrently, our analysis of professional perspectives revealed a system in crisis, characterised by four core themes: Recognition, Legitimacy, and Access to Care; Care Coordination, Capacity, and Sustainability; Individualised and Holistic Care Models; and Mental Health and Emotional Dimensions. The professional trajectory is one of adaptation, shifting from diagnostic uncertainty to a clear demand for systemic change.

### 8.2. Interpretation and Synthesis of Evidence

Our findings underscore that long COVID is not just a biomedical condition, but emblematic of a crisis of legitimacy, which is a failure of healthcare systems to recognise and validate symptoms that defy traditional diagnostic frameworks. This epistemic marginalisation inflicted real harm, silencing patients and exacerbating psychological distress [68].

Such invalidation often intersected with gendered biases, as women’s symptoms were more likely to be dismissed or trivialised, compounding distrust and emotional burden [42]. The psychosocial consequences of being doubted, invalidated, and socially isolated proved as devastating as the physical symptoms [54].

In response, patient-led peer support networks emerged as powerful vehicles for validation, shared knowledge, and emotional safety—filling voids left by overwhelmed formal services [68]. These grassroots initiatives mirror broader literature highlighting how lay expertise can challenge medical authority and support marginalised health experiences [77].

Concurrently, professional perspectives lamented fragmented, siloed services and the absence of a holistic approach, reflecting a convergence of systemic vulnerabilities, austerity-induced resource contains and workforce shortages exacerbated by pandemic pressures [78]. As detailed in Section 7 and illustrated in Figure 3, this convergence theme emerged inductively from both patient and professional perspectives. Yet amid the crisis, both patients and professionals displayed remarkable adaptive resilience. Patients became adept self-managers, reconstructing daily lives around relapse-recovery cycles. Professionals began advocating for individualised, integrated, and holistic care models that recognise long COVID’s complexity and chronicity [21].

While psychoeducation and physical rehabilitation were described in the included studies, these approaches require careful contextualisation. Psychoeducation may be useful as a supportive measure but cannot substitute for appropriate biomedical care, such as the management of cardiovascular, respiratory, or metabolic complications. Similarly, although rehabilitation was mentioned, it may be contraindicated in certain disease manifestations, such as myocarditis or post-exertional malaise (PEM), and cannot alone address the multi-system pathology of long COVID.

While such holistic supports (e.g., psychoeducation, nutrition, sleep, activity balance) were described in the included studies, these approaches must be contextualised as adjunctive. They cannot substitute for appropriate biomedical care for long COVID’s multi-system pathology, which may require cardiovascular, respiratory, or metabolic management. It is important to note that our synthesis was limited to qualitative studies of patient and professional perspectives; accordingly, biomedical treatments (such as anticoagulants or cardiac medication) were rarely reported. This absence reflects the scope of the qualitative literature we analysed rather than the unimportance of biomedical management, which remains central to long COVID care. These limitations highlight the importance of comprehensive, integrated models of care that combine biomedical treatment with psychosocial support.

This complementary evolution highlights a critical perspective for long COVID response: it is not sufficient to pursue purely biomedical solutions. Instead, we must intentionally rebuild trust, legitimise lived experience, and reconfigure health systems to support complex, chronic, often invisible illnesses—anchored in equity, responsiveness, and empathy.

### 8.3. Strengths and Limitations

A key strength of this synthesis is its robust methodology, which provides a rich, in-depth understanding of the experiences of both patients and healthcare professionals. The inclusion of studies with diverse participant populations, including those from ethnic minority backgrounds, and the involvement of patient advisory groups in study design and interpretation are significant strengths that enhance the validity and generalizability of the findings.

However, this review is subject to certain limitations. While the synthesis drew from a wide range of qualitative studies, there were few representations from older age groups, and some studies lacked a comparative analysis with people from White backgrounds to better understand the specific challenges faced by ethnic minorities [45]. Furthermore, some of the studies relied on self-reported symptoms and lacked evidence of how the researchers’ own perspectives influenced the research findings. The qualitative nature of the data means that findings, while rich, may not be generalisable to the wider population.

While this review synthesised qualitative research, and therefore did not directly assess biological mechanisms, it is important to acknowledge the growing body of biomedical evidence on long COVID pathophysiology. Studies highlight persistent inflammation, multi-organ sequelae, and cardiovascular, neurological, and immunological dysregulation [3,4,5]. Because our synthesis excluded pathophysiological research, it may under-represent biological abnormalities and associated treatments, while amplifying psychosocial dimensions more frequently discussed in qualitative literature. Our findings should thus be interpreted as complementary to, rather than exclusive of, biomedical research.

## 9. Conclusions and Recommendations

Our synthesis reveals that long COVID is not merely a medical condition; it is a biological condition that also intersects with social and psychological dimensions, challenging the foundations of how health systems understand, validate, and support chronic, invisible illness. Patients often grapple with profound stigma, invalidation, and a crisis of legitimacy that can be as disabling as their physical symptoms. In response, many have become innovative self-managers and creators of peer-led support networks that fill the gaps left by fragmented care. Meanwhile, professionals are increasingly recognising the value of these lived experiences and seeking to shift toward more integrated, empathetic, and holistic models of care, even as they contend with systemic resource constraints and service silos.

To move forward, health systems must commit to sustained, structurally embedded, and well-resourced models of care—that is, systems that prioritise continuity, coordination, and multidisciplinary collaboration over fragmented, time-limited services. Clinicians need standardised training rooted in empathy, validation, and shared decision-making, equipping them to support not just physical recovery, but also psychological and social rehabilitation. Public health must actively work to dismantle stigma, elevate legitimacy, and generate widespread societal understanding of long COVID through inclusive, compassion-driven campaigns. Finally, research must deepen our understanding of the evolving dynamics of patient–provider relationships and evaluate comparative models of care internationally, so we may learn, adapt, and build systems capable of responding to long COVID’s enduring and complex challenges.

## Figures and Tables

**Table 1:** Core study characteristics.

Study (Citation)	Country	Participants	Study Design, Data Collection, and Analysis Method	Sample Size (Total Number, Women)
Seers et al., 2025 [29]	UK (England & Wales)	People with Long COVID	Qualitative evaluation with framework & thematic analysis; Semi-structured interviews	45 (20)
Sarma et al., 2025 [30]	USA	People with Long COVID	Qualitative; semi-structured individual interviews	18 (14)
Nguyen et al., 2025 [21]	Canada	Healthcare professionals	Thematic analysis; Interviews	20 (15)
Milne et al., 2025 [31]	UK	People with Long COVID	Qualitative study (interpretive phenomenological approach); Semi-structured interviews	12 (7)
MacLean et al., 2025 [32]	UK	People with Long COVID	Comparative qualitative analysis; Qualitative interviews analysed in NVivo	93 (56)
J et al., 2025 [33]	India	People with Long COVID	Qualitative; In-depth interviews	N = 315; women = (40.6%)
Funk et al., 2025 [34]	Germany	People with Long COVID	Thematic analysis; Focus groups; online group,	22 (17)
Faux-Nightingale et al., 2025 [35]	UK	People with Long COVID + Carers/family	Thematic analysis; Interviews with patients; Focus groups with professionals	4 (4)-Patients 7 (NR)-professional
Buettikofer et al., 2025 [36]	Australia	People with Long COVID	Qualitative study; inductive thematic analysis; Semi-structured interviews	15 (11)
Turk et al., 2024 [37]	UK	People with Long COVID + Healthcare professional	Qualitative study and framework analysis; Interviews	Patients: 8 (3); Professional: 8 (5)
Reay et al., 2024 [38]	UK	People with Long COVID	Qualitative; Semi-structured interviews	10 (6)
Miller et al., 2024 [39]	UK	People with Long COVID	Qualitative focus groups; Framework Analysis; Eight focus groups (online + in-person)	25 (8)
Leggat et al., 2024 [40]	England, Wales	People with Long COVID	Reflexive thematic analysis; Interviews	18 (12)
Laestadius et al., 2024 [41]	USA	People with Long COVID	Semi-structured interviews; Framework analysis	30 (30)
Kalfas et al., 2024 [42]	UK	People with Long COVID + Health professionals	Interviews; Thematic analysis	19 (13)
Gamillscheg et al., 2024 [43]	Austria	Healthcare professionals + People with Long COVID	Semi-structured interviews; focus group; Qualitative thematic framework approach	Experts:15 (8) Patients:18 (13)
Fang et al., 2024 [44]	UK	People with Long COVID+ Healthcare professionals	Longitudinal qualitative study; Qualitative interviews; (longitudinal) reflexive thematic analysis	Patients 80 (56)- Healthcare provider 12 (NR)
Cooper et al., 2024 [45]	Scotland	People with Long COVID + Healthcare professionals	Interviews; Qualitative	Patient: 11 (10) GP: 13 (8)
Boutry et al., 2024 [46]	UK	People with Long COVID discussing return to work	Interviews; Qualitative study	34 (29)
Al-Jabr et al., 2024 [47]	UK	People with Long COVID	LC-OHP Programme sessions; Thematic analysis	26 (24)
Thomas et al., 2023 [48]	UK	People with Long COVID	Longitudinal qualitative diary study; Hand-written patient diaries analysed inductively	12 (11)
Stelson et al., 2023 [49]	USA	People with Long COVID	Internet-based mixed-method, cross-sectional survey; Thematic analysis, a directed content analysis approach	510 (296)
Skilbeck et al., 2023 [50]	UK	People with Long COVID	Semi-structured interviews; Interpretative phenomenological analysis (IPA) methodology	18 (13)
Silwal et al., 2023 [51]	Nepal	People with Long COVID + Carers/family	In-depth interviews; Thematic analysis	19 (NR)
Schmachtenberg et al., 2023 [52]	Germany	People with Long COVID	Interviews; Content analysis	25 (18)
Schmachtenberg et al., 2023 [53]	Germany	People with Long COVID	Focus groups; Content analysis	19 (12)
Messiah et al., 2023 [54]	USA	People with Long COVID + Carers/family	In-depth interviews; Qualitative	25 (13)
Kennelly et al., 2023 [19]	Canada	People with Long COVID	Focus groups; Thematic analysis	47 (28)
Horlick et al., 2023 [55]	Canada	Healthcare professionals	Semi-structured interviews; Content analysis	15 (NR)
Gyllensten et al., 2023 [56]	Sweden	People with Long COVID	Semi-structured focus group Interviews; Thematic analysis	19 (13)
Duncan et al., 2023 [57]	Scotland	People with Long COVID + Healthcare professionals	Interviews; Qualitative	38 (33)
Duan et al., 2023 [58]	USA	People with Long COVID	Survey with free answered questions; Grounded theory	134 (53)
Brehon et al., 2023 [59]	Canada	People with Long COVID	Interviews; Reflexive thematic analysis	56 (34)
Bogale et al., 2023 [60]	Ethiopia	People with Long COVID	Phenomenology; In-depth interviews; Thematic Analysis	23 (10)
Wurz et al., 2022 [61]	Canada	People with Long COVID	Open-ended survey; Thematic analysis	169 (149)
Schiavi et al., 2022 [62]	Italy	People with Long COVID	Interviews; Empirical phenomenological approach	56 (22)
Schaap et al., 2022 [63]	Netherlands	People with Long COVID	In-depth interviews; Reflexive thematic approach	24 (7)
Santiago-Rodriguez et al., 2022 [64]	USA	People with Long COVID	Thematic analysis; In-depth interviews	24 (9)
Piras et al., 2022 [65]	Italy	People with Long COVID	Semi-structured interviews; Qualitative	12 (3)
O’Hare et al., 2022 [66]	USA	Care Notes from Healthcare professionals	Professionals’ notes; Inductive content analysis	200 (27)
Loft et al., 2022 [67]	Denmark	People with Long COVID	Semi-structured interviews; Three-phase phenomenological-hermeneutic approach	19 (15)
Ireson et al., 2022 [68]	UK	People with Long COVID	Phenomenology; Patient stories submitted online; Thematic analysis; A three-stage process of analysis	66 (NR)
Gerlis et al., 2022 [69]	UK	People with Long COVID	Interviews, focus group, Survey; Thematic analysis	13 (6)
Day et al., 2022 [70]	UK	People with Long COVID	Semi-structured interviews; Thematic analysis	1 (3)
Chasco et al., 2022 [71]	USA	People with Long COVID + Carers/family	Interviews; Systematic thematic analysis	15 (10)
Callan et al., 2022 [72]	UK	People with Long COVID	Focus groups; Qualitative	50 (42)
Aghaei et al., 2022 [73]	USA	People with Long COVID	Semi-structured interviews; Thematic analysis	15 (NR)
Shelley et al., 2021 [74]	UK	People with Long COVID	Semi-structured interviews; Thematic analysis	48 (41)
Humphreys et al., 2021 [75]	UK	People with Long COVID	Semi-structured interviews; Qualitative	18 (9)

Key to abbreviations: LC-OHP = Long COVID Optimal Health Programme; IPA = Interpretative Phenomenological Analysis; NR = Not reported; GP = General practitioner; COVID = Coronavirus disease 2019; COVID+ = COVID-positive; UK = United Kingdom; USA = United States of America. Note: Detailed participant categories are available in Supplementary File S8.

## Data Availability

Extracted study characteristics, coding framework, and analytic memos are available in the Supplementary Materials; additional materials will be shared on reasonable request to the corresponding author. Full search strategies, screening form, data extraction template, codebook, and completed CERQual tables will be deposited in an open repository.

## Supplementary Materials

The following supporting information can be downloaded at: https://www.mdpi.com/article/10.3390/ijerph22111620/s1. Supplementary File S1: Inclusion & Exclusion Criteria—Long COVID Qualitative Review; Supplementary File S2: Database Search Strategies (Long COVID Qualitative Synthesis); Supplementary File S3: Grey Literature Sources and Citation Chasing Methods. Supplementary File S4: Summary of Qualitative Findings SoQF + CERQual Justifications and CERQual Confidence Rating; Supplementary File S5: Summary of Review Findings and CERQual Assessment; Supplementary File S6: Codebook; Supplementary File S7: Reflexive Notes on Coding Decisions; Supplementary File S8: Study & Participant Characteristics; Supplementary File S9: Patient Themes, Subthemes, Quotations and Citations; Supplementary File S10: Professional Themes, Subthemes, Quotations and Citations.

## References

[1] WHO (2021). A Clinical Case Definition of Post COVID-19 Condition by a Delphi Consensus.

[2] National Academies of Sciences, Engineering, and Medicine (2024). A Long COVID Definition: A Chronic, Systemic Disease State with Profound Consequences.

[3] Davis H.E., McCorkell L., Vogel J.M., Topol E.J. (2023). Long COVID: Major findings, mechanisms and recommendations. Nat. Rev. Microbiol..

[4] Xie Y., Xu E., Bowe B., Al-Aly Z. (2022). Long-term cardiovascular outcomes of COVID-19. Nat. Med..

[5] Crook H., Raza S., Nowell J., Young M., Edison P. (2021). Long covid-mechanisms, risk factors, and management. BMJ.

[6] Taher M.K., Salzman T., Banal A., Morissette K., Domingo F.R., Cheung A.M., Cooper C.L., Boland L., Zuckermann A.M., Mullah M.A. (2025). Global prevalence of post-COVID-19 condition: A systematic review and meta-analysis of prospective evidence. Health Promot. Chronic Dis. Prev. Can..

[7] O’Mahoney L.L., Routen A., Gillies C., Ekezie W., Welford A., Zhang A., Karamchandani U., Simms-Williams N., Cassambai S., Ardavani A. (2023). The prevalence and long-term health effects of Long Covid among hospitalised and non-hospitalised populations: A systematic review and meta-analysis. eClinicalMedicine.

[8] Agarwal M., Pandit P., Khan M., Jauhari S., Singh A., Mishra S., Verma S. (2024). Long-haul COVID-19 and its associated risk factors: A systematic review and meta-analysis. J. Public Health.

[9] WHO (World Health Organization) (2024). Clinical Management of COVID-19: Policy brief, 15 December 2024.

[10] Filip R., Gheorghita Puscaselu R., Anchidin-Norocel L., Dimian M., Savage W.K. (2022). Global Challenges to Public Health Care Systems during the COVID-19 Pandemic: A Review of Pandemic Measures and Problems. J. Pers. Med..

[11] Owen R., Ashton R.E., Bewick T., Copeland R.J., Ferraro F.V., Kennerley C., Phillips B.E., Maden-Wilkinson T., Parkington T., Skipper L. (2025). Profiling the persistent and episodic nature of long COVID symptoms and the impact on quality of life and functional status: A cohort observation study. J. Glob. Health.

[12] Greenhalgh T., Sivan M., Perlowski A., Nikolich J.Ž. (2024). Long COVID: A clinical update. Lancet.

[13] National Institute for Health and Care Excellence (2024). COVID-19 Rapid Guideline: Managing the Long-Term Effects of COVID-19 (NG188, Updated 2024).

[14] NHS (NHS England) (2023). Long COVID: Commissioning Guidance.

[15] Centers for Disease Control and Prevention (2022). Long COVID or Post-COVID Conditions.

[16] National Institute for Health and Care Excellence (2021). Myalgic Encephalomyelitis (or Encephalopathy)/Chronic Fatigue Syndrome: Diagnosis and Management.

[17] Wormgoor M.E.A., Rodenburg S.C. (2023). Focus on post-exertional malaise when approaching ME/CFS in specialist healthcare improves satisfaction and reduces deterioration. Front. Neurol..

[18] Stussman B., Camarillo N., McCrossin G., Stockman M., Norato G., Vetter C.S., Ferrufino A., Adedamola A., Grayson N., Nath A. (2025). Post-exertional malaise in Long COVID: Subjective reporting versus objective assessment. Front. Neurol..

[19] Kennelly C.E., Nguyen A.T.P., Sheikhan N.Y., Strudwick G., Ski C.F., Thompson D.R., Bartram M., Soklaridis S., Rossell S.L., Castle D. (2023). The lived experience of long COVID: A qualitative study of mental health, quality of life, and coping. PLoS ONE.

[20] Hibbert P.D., Stewart S., Wiles L.K., Braithwaite J., Runciman W.B., Thomas M.J.W. (2023). Improving patient safety governance and systems through learning from successes and failures: Qualitative surveys and interviews with international experts. Int. J. Qual. Health Care.

[21] Nguyen A.T.P., Ski C.F., Thompson D.R., Abbey S.E., Kloiber S., Sheikhan N.Y., Selby P., Shields R., Rossell S.L., Strudwick G. (2025). Health and social service provider perspectives on challenges, approaches, and recommendations for treating long COVID: A qualitative study of Canadian provider experiences. BMC Health Serv. Res..

[22] Manhas K.P., Horlick S., Krysa J., Kovacs Burns K., Brehon K., Laur C., Papathanassoglou E., Ho C. (2024). Implementation of a Provincial Long COVID Care Pathway in Alberta, Canada: Provider Perceptions. Healthcare.

[23] Cassidy S., Solvang Ø.S., Granja C., Solvoll T. (2024). Flipping healthcare by including the patient perspective in integrated care pathway design: A scoping review. Int. J. Med. Inform..

[24] Geese F., Schmitt K.U. (2023). Interprofessional Collaboration in Complex Patient Care Transition: A Qualitative Multi-Perspective Analysis. Healthcare.

[25] Critical Appraisal Skills Programme (CASP) (2018). CASP Qualitative Studies Checklist.

[26] Lewin S., Booth A., Glenton C., Munthe-Kaas H., Rashidian A., Wainwright M., Bohren M.A., Tunçalp Ö., Colvin C.J., Garside R. (2018). Applying GRADE-CERQual to qualitative evidence synthesis findings: Introduction to the series. Implement. Sci..

[27] Tong A., Flemming K., McInnes E., Oliver S., Craig J. (2012). Enhancing transparency in reporting the synthesis of qualitative research: ENTREQ. BMC Med. Res. Methodol..

[28] Page M.J., McKenzie J.E., Bossuyt P.M., Boutron I., Hoffmann T.C., Mulrow C.D., Shamseer L., Tetzlaff J.M., Akl E.A., Brennan S.E. (2021). The PRISMA 2020 statement: An updated guideline for reporting systematic reviews. BMJ.

[29] Seers K., Nichols V.P., Bruce J., Ennis S., Heine P., Patel S., Sandhu H.K., Underwood M., McGregor G. (2025). Qualitative evaluation of the Rehabilitation Exercise and psycholoGical support After COVID-19 InfectioN (REGAIN) randomised controlled trial (RCT): ‘you are not alone’. BMJ Open.

[30] Sarma N., Gage S., Hough C.L., Hope A.A. (2025). ‘We Don’t Have to Prove to People How We’re Feeling’: Understanding the Role of Peer Support Groups in Countering Epistemic Injustices in Long COVID at a US Centre. Health Expect.

[31] Milne A., Arnold D., Moore A. (2025). Understanding post-hospitalised patients’ experiences of long COVID—The PELCO study. J. Health Psychol..

[32] MacLean A., Driessen A., Hinton L., Nettleton S., Wild C., Anderson E., Brown A., Hoddinott P., O’Dwyer C., Ziebland S. (2025). Rethinking ‘Recovery’: A Comparative Qualitative Analysis of Experiences of Intensive Care With COVID and Long Covid in the United Kingdom. Health Expect.

[33] Koshy J.M.S.D., Narreddy S., Gowri S.M., Rupali P., Sathyendra S. (2025). Prevalence and predictors of long COVID at 1 year in a cohort of hospitalized patients: A multicentric qualitative and quantitative study. PLoS ONE.

[34] Funk M., Reinke M., Löwe B., Engelmann P. (2025). Development of an expectation management intervention for patients with Long COVID: A focus group study with affected patients. PLoS ONE.

[35] Faux-Nightingale A., Saunders B., Burton C., Chew-Graham C.A., Somayajula G., Twohig H., Welsh V. (2025). Perceptions and Significance of Long Covid Diagnoses From the Perspectives of Children and Young People With Long Covid, Their Parents and Professionals. Health Expect.

[36] Buettikofer T., Maher A., Rainbird V., Bennett M., Freene N., Mitchell I., Huang H.C., Gaughwin P., Johnson M., Paratz J. (2025). Consumer Experience of an Australian Multidisciplinary Long COVID Clinic That Incorporates Personalised Exercise Prescription: A Qualitative Analysis. Health Expect.

[37] Turk F., Sweetman J., Chew-Graham C.A., Gabbay M., Shepherd J., van der Feltz-Cornelis C. (2024). Accessing care for Long Covid from the perspectives of patients and healthcare practitioners: A qualitative study. Health Expect.

[38] Reay A., Dismore L., Aujayeb A., Dotchin C., Tullo E., Steer J., Swainston K. (2024). Analysing the patient experience of COVID-19: Exploring patients’ experiences of hospitalisation and their quality of life post discharge. J. Clin. Nurs..

[39] Miller A., Song N., Sivan M., Chowdhury R., Burke M.R. (2024). Identifying the needs of people with long COVID: A qualitative study in the UK. BMJ Open.

[40] Leggat F.J., Heaton-Shrestha C., Fish J., Siriwardena A.N., Domeney A., Rowe C., Patel I., Parsons J., Blair J., Jones F. (2024). An exploration of the experiences and self-generated strategies used when navigating everyday life with Long Covid. BMC Public Health.

[41] Laestadius L.I., Guidry J.P.D., Wahl M.M., Perrin P.B., Carlyle K.E., Dong X., Gharbo R., Campos-Castillo C. (2024). “The dream is that there’s one place you go”: A qualitative study of women’s experiences seeking care from Long COVID clinics in the USA. BMC Med..

[42] Kalfas M., Jolley C., Hart N., Rafferty G.F., Duncan E.L., Nicholson T., Ashworth M., Brewin D., Barrett B., Witard O.C. (2024). Exploring the Experiences of Living With the Post-COVID Syndrome: A Qualitative Study. Health Expect.

[43] Gamillscheg P., Łaszewska A., Kirchner S., Hoffmann K., Simon J., Mayer S. (2024). Barriers and facilitators of healthcare access for long COVID-19 patients in a universal healthcare system: Qualitative evidence from Austria. Int. J. Equity Health.

[44] Fang C., Baz S.A., Sheard L., Carpentieri J.D. (2024). “They seemed to be like cogs working in different directions”: A longitudinal qualitative study on Long COVID healthcare services in the United Kingdom from a person-centred lens. BMC Health Serv. Res..

[45] Cooper K., Duncan E., Hart-Winks E., Cowie J., Shim J., Stage E., Tooman T., Alexander L., Love A., Morris J.H. (2024). Exploring the perceptions and experiences of community rehabilitation for Long COVID from the perspectives of Scottish general practitioners’ and people living with Long COVID: A qualitative study. BMJ Open.

[46] Boutry C., Patel P., Holmes J., Radford K., Bolton C.E., Evangelou N., das Nair R., Morriss R. (2024). Returning to work with long covid in the UK during lockdown and other COVID-19 restrictions: A qualitative study. PLoS ONE.

[47] Al-Jabr H., Thompson D.R., Castle D.J., Ski C.F. (2024). Experiences of people with long COVID: Symptoms, support strategies and the Long COVID Optimal Health Programme (LC-OHP). Health Expect.

[48] Thomas C., Faghy M.A., Owen R., Yates J., Ferraro F., Bewick T., Haggan K., Ashton R.E.M. (2023). Lived experience of patients with Long COVID: A qualitative study in the UK. BMJ Open.

[49] Stelson E.A., Dash D., McCorkell L., Wilson C., Assaf G., Re’em Y., Wei H. (2023). Return-to-work with long COVID: An Episodic Disability and Total Worker Health^®^ analysis. Soc. Sci. Med..

[50] Skilbeck L., Spanton C., Paton M. (2023). Patients’ lived experience and reflections on long COVID: An interpretive phenomenological analysis within an integrated adult primary care psychology NHS service. J. Patient Rep. Outcomes.

[51] Silwal S., Parajuli K., Acharya A., Ghimire A., Pandey S., Pandey A., Poudyal A., Bista B., Gyanwali P., Dhimal M. (2023). Physical, mental and social status after COVID-19 recovery in Nepal: A mixed method study. PLoS ONE.

[52] Schmachtenberg T., Müller F., Kranz J., Dragaqina A., Wegener G., Königs G., Roder S. (2023). How do long COVID patients perceive their current life situation and occupational perspective? Results of a qualitative interview study in Germany. Front. Public Health.

[53] Schmachtenberg T., Königs G., Dragaqina A., Roder S., Müller F., Müllenmeister C., Schröder D., Dopfer-Jablonka A., Vieth K., El-Sayed I. (2023). “There is no one who helps you with it”: Experiences of people with long COVID regarding medical care, therapeutic measures, and barriers in the German healthcare system: Results of a qualitative study with four focus groups. BMC Health Serv. Res..

[54] Messiah S.E., Francis J., Weerakoon S., Mathew M.S., Shaikh S., Veeraswamy A., Lozano A., He W., Xie L., Polavarapu D. (2023). Persistent symptoms and conditions among children and adolescents hospitalised with COVID-19 illness: A qualitative study. BMJ Open.

[55] Horlick S., Krysa J.A., Brehon K., Pohar Manhas K., Kovacs Burns K., Russell K., Papathanassoglou E., Gross D.P., Ho C. (2023). Exploring Rehabilitation Provider Experiences of Providing Health Services for People Living with Long COVID in Alberta. Int. J. Environ. Res. Public Health.

[56] Gyllensten K., Holm A., Sandén H. (2023). Workplace factors that promote and hinder work ability and return to work among individuals with long-term effects of COVID-19: A qualitative study. Work.

[57] Duncan E., Alexander L., Cowie J., Love A., Morris J.H., Moss R., Ormerod J., Preston J., Shim J., Stage E. (2023). Investigating Scottish Long COVID community rehabilitation service models from the perspectives of people living with Long COVID and healthcare professionals: A qualitative descriptive study. BMJ Open.

[58] Duan E., Garry K., Horwitz L.I., Weerahandi H. (2023). “I Am Not the Same as I Was Before”: A Qualitative Analysis of COVID-19 Survivors. Int. J. Behav. Med..

[59] Brehon K., Miciak M., Hung P., Chen S.P., Perreault K., Hudon A., Wieler M., Hunter S., Hoddinott L., Hall M. (2023). “None of us are lying”: An interpretive description of the search for legitimacy and the journey to access quality health services by individuals living with Long COVID. BMC Health Serv. Res..

[60] Bogale K.A., Zeru T., Tarkegn M., Balew M., Worku M., Asrat A., Adamu A., Mulu Y., Getachew A., Ambaw F. (2023). Awareness and care seeking for long COVID symptoms among Coronavirus disease survivors in Bahir Dar City, Northwest Ethiopia: Phenomenological study. BMC Public Health.

[61] Wurz A., Culos-Reed S.N., Franklin K., DeMars J., Wrightson J.G., Twomey R. (2022). “I feel like my body is broken”: Exploring the experiences of people living with long COVID. Qual. Life Res..

[62] Schiavi M., Fugazzaro S., Bertolini A., Denti M., Mainini C., Accogli M.A., Bedogni G., Ghizzoni D., Esseroukh O., Gualdi C. (2022). “Like before, but not exactly”: The Qualy-REACT qualitative inquiry into the lived experience of long COVID. BMC Public Health.

[63] Schaap G., Wensink M., Doggen C.J.M., van der Palen J., Vonkeman H.E., Bode C. (2022). “It Really Is an Elusive Illness”-Post-COVID-19 Illness Perceptions and Recovery Strategies: A Thematic Analysis. Int. J. Environ. Res. Public Health.

[64] Santiago-Rodriguez E.I., Maiorana A., Peluso M.J., Hoh R., Tai V., Fehrman E.A., Hernandez Y., Torres L., Spinelli M.A., Gandhi M. (2022). Characterizing the COVID-19 Illness Experience to Inform the Study of Post-acute Sequelae and Recovery. Int. J. Behav. Med..

[65] Piras I., Piazza M.F., Piccolo C., Azara A., Piana A., Finco G., Galletta M. (2022). Experiences, Emotions, and Health Consequences among COVID-19 Survivors after Intensive Care Unit Hospitalization. Int. J. Environ. Res. Public Health.

[66] O’Hare A.M., Vig E.K., Iwashyna T.J., Fox A., Taylor J.S., Viglianti E.M., Butler C.R., Vranas K.C., Helfand M., Tuepker A. (2022). Complexity and Challenges of the Clinical Diagnosis and Management of Long COVID. JAMA Netw. Open.

[67] Loft M.I., Foged E.M., Koreska M. (2022). An Unexpected Journey: The Lived Experiences of Patients with Long-Term Cognitive Sequelae After Recovering from COVID-19. Qual. Health Res..

[68] Ireson J., Taylor A., Richardson E., Greenfield B., Jones G. (2022). Exploring invisibility and epistemic injustice in Long Covid-A citizen science qualitative analysis of patient stories from an online Covid community. Health Expect.

[69] Gerlis C., Barradell A., Gardiner N.Y., Chaplin E., Goddard A., Singh S.J., Daynes E. (2022). The Recovery Journey and the Rehabilitation Boat—A qualitative study to explore experiences of COVID-19 rehabilitation. Chron. Respir. Dis..

[70] Day H.L.S. (2022). Exploring Online Peer Support Groups for Adults Experiencing Long COVID in the United Kingdom: Qualitative Interview Study. J. Med. Internet Res..

[71] Chasco E.E., Dukes K., Jones D., Comellas A.P., Hoffman R.M., Garg A. (2022). Brain Fog and Fatigue following COVID-19 Infection: An Exploratory Study of Patient Experiences of Long COVID. Int. J. Environ. Res. Public Health.

[72] Callan C., Ladds E., Husain L., Pattinson K., Greenhalgh T. (2022). ‘I can’t cope with multiple inputs’: A qualitative study of the lived experience of ‘brain fog’ after COVID-19. BMJ Open.

[73] Aghaei A., Aggarwal A., Zhang R., Li X., Qiao S. (2022). Resilience resources and coping strategies of COVID-19 female long haulers: A qualitative study. Front. Public Health.

[74] Shelley J., Hudson J., Mackintosh K.A., Saynor Z.L., Duckers J., Lewis K.E., Davies G.A., Berg R.M.G., McNarry M.A. (2021). ‘I Live a Kind of Shadow Life’: Individual Experiences of COVID-19 Recovery and the Impact on Physical Activity Levels. Int. J. Environ. Res. Public Health.

[75] Humphreys H., Kilby L., Kudiersky N., Copeland R. (2021). Long COVID and the role of physical activity: A qualitative study. BMJ Open.

[76] Khashei M., Janiczak S., St Clair C., Liu W., Song J.J., Hua W., Falconer M., Eworuke E. (2023). Social media for early characterization of pandemic symptoms: A qualitative analysis of patient-reported COVID-19 experiences. Pharmacoepidemiol. Drug Saf..

[77] Chapman A., Buccheri A., Mohotti D., Wong Shee A., Huggins C.E., Alston L., Hutchinson A.M., Yoong S.L., Beks H., Mc Namara K. (2025). Staff-reported barriers and facilitators to the implementation of healthcare interventions within regional and rural areas: A rapid review. BMC Health Serv. Res..

[78] Habib M., Palachi A., Korman M.B., Steinberg R., Cocco C., Martin-Doto C., Tuka A., Cao X., Sinyor M., Ellis J. (2025). Patterns of Distress and Supportive Resource Use by Healthcare Workers During the COVID-19 Pandemic. Healthcare.

