# Supplementary material for: Patient and Professional Perspectives on Long COVID: A Systematic Literature Review and Meta-Synthesis
Source: Int J Environ Res Public Health. 2025 Oct 24;22(11):1620. doi: 10.3390/ijerph22111620 (PMC12652048; doi:10.3390/ijerph22111620)
Supplement: Supplementary file 1 [file ijerph-22-01620-s001.zip › ijerph-3876455-supplementary.pdf]

Supplementary File S1. Inclusion & Exclusion Criteria — Long COVID Qualitative Review

Version date: 30.07.2025

This document is for Rayyan setup and full-text adjudication. It aligns with the Methods section and PRISMA reporting.

A. Short version (paste into Rayyan "Description")

Inclusion (ALL must be met):

- Population: People with Long COVID (post-COVID-19 condition, PASC) and/or health professionals caring for them.
- Phenomenon: Experiences, perspectives, needs, care/access, coping, work/role impacts, or service delivery related to Long COVID.
- Design: Primary qualitative studies or qualitative components of mixed-methods with extractable qualitative findings.
- Data type: Author-reported themes/subthemes and/or verbatim participant quotations.
- Timeframe: Jan 1, 2020 to Aug 19, 2025.
- Language: English.
- Setting: Any country/setting (community, primary care, hospital/clinic, rehabilitation, workplace, online).
- Publication status: Peer-reviewed journal articles

Exclusion (ANY is grounds):

- Not Long COVID (acute COVID ≤12 weeks only; or generic "post-COVID sequelae" with no LC focus).
- Not qualitative (quantitative-only; surveys without qualitative analysis).
- No primary data (editorials, opinion, news, protocols, guidelines).
- Conference abstract only without a full paper reporting primary qualitative data.
- Wrong population (neither LC patients nor health professionals caring for LC).
- Wrong focus (biology/pathophysiology only; lab/imaging only; tool validation without experiences).
- Language not English.
- Outside date range (before 2020-01-01 or after 2025-08-19).
- Unpublished/grey literature (preprints, theses, dissertations, organizational reports).
- Duplicate record.

1

B. Full-text adjudication rules

1) Long COVID definition (operational): Include if authors use terms such as Long COVID, post-COVID-19 condition, PASC, or clearly study ongoing symptoms ≥12 weeks post infection. Mixed samples (4–12 weeks + ≥12 weeks) are eligible if findings relate to persistent illness.

2) Mixed-methods studies: Include only if the qualitative METHODS and FINDINGS are sufficiently described and findings are extractable (themes/quotes). Exclude if qualitative part is anecdotal with no analysis.

3) Professional-only studies: Eligible if focused on care/management of Long COVID, service delivery, role strain, access barriers, or perceptions of patient experience.

4) Designs considered qualitative: Interviews (any format), focus groups, ethnography/fieldwork, phenomenology, grounded theory, narrative inquiry, thematic/framework/content analysis. Case studies are eligible only if they include systematic qualitative analysis beyond description.

5) Minimum sample size: No formal minimum. Very small n is eligible if there is clear qualitative analysis and sufficient data richness (themes + illustrative quotes). Otherwise exclude as "Insufficient methods detail / data richness."

6) Conference material: Abstracts alone are excluded. If a full article exists within the date range with primary qualitative data, include the full article.

7) Preprints/grey literature: Excluded per Methods (peer-reviewed only). Revise if scope changes.

C. Rayyan "Reasons for exclusion" mapping (PRISMA-aligned)

Reason (short)	Definition	PRISMA Section B category
Not Long COVID	Not about post-acute COVID-19 condition/sequelae	Wrong condition/phenomenon
Not qualitative	No primary qualitative data; quantitative-only	Wrong study design

2

No primary data	Editorial/commentary/news/protocol only	Non-primary/Not empirical
Wrong population	Not LC patients nor health professionals caring for LC	Wrong population
Wrong focus	Not experiences/perspectives/engagement	Wrong outcome/phenomenon
Wrong setting	Irrelevant setting (e.g., lab-only)	Wrong setting
Language	Not in English	Language
Year out of range	Published before 2020	Date range
Conference abstract only	Abstract without primary qualitative data	Publication type
Duplicate	Duplicate record	Duplicate
Not retrievable	Full text could not be obtained	Reports not retrieved
Insufficient methods detail	Cannot verify qualitative methods	Methodological limitation (screening)

3

Supplementary File S2. Database Search Strategies (Long COVID Qualitative Synthesis).

Databases: MEDLINE (PubMed), Ovid MEDLINE, Embase (Ovid), PsycINFO (Ovid), CINAHL (EBSCOhost), Scopus, Web of Science Core Collection. Date coverage: 01.01. 2020 to 08.19.2025. Language: English. Note: The core strategy uses Long COVID terms AND a qualitative filter; an optional population limiter is supplied if needed.

Grey literature and citation chasing

Reference lists of included studies; WHO, NICE, CDC and ECDC webpages scanned for links to peer-reviewed studies; Google Scholar alerts for in-press articles (inclusion restricted to peer-reviewed publications).

Record management

All records exported with full fields and imported to EndNote for automated and manual de-duplication; dual screening at title/abstract and full text with reasons for exclusion logged (PRISMA).

SEARCH RESULTS

DATA BASE	SEARCH STRING	RESULTS (NUMBER OF RECORDS YEILD	SEARCH DATE

**MEDLINE (Pubmed)**	(("Long COVID"[tiab]) OR ("Post-Acute COVID-19 Syndrome"[MeSH Terms]) OR ("post-acute sequela* of sars-cov-2"[tiab]) OR (PASC[tiab]) OR ("post covid*"[tiab]) OR ("post-covid*"[tiab]) OR (postcovid*[tiab]) OR ("post covid-19 condition"[tiab]) OR ("post-covid-19 condition"[tiab]) OR ("long haul*"[tiab]) OR ("long-haul*"[tiab]) OR ("long hauler*"[tiab]) OR ("long-hauler*"[tiab]) OR ("chronic covid*"[tiab]) OR ("persistent covid*"[tiab]) OR ("sequela* of covid*"[tiab])) AND (("Qualitative Research"[MeSH Terms]) OR (qualitative[tiab]) OR (interview*[tiab]) OR ("focus group*"[tiab]) OR ("semi-structured"[tiab]) OR (semistructured[tiab]) OR ("in-depth"[tiab]) OR (ethnograph*[tiab]) OR (phenomenol*[tiab]) OR ("grounded theor*"[tiab]) OR ("thematic analy*"[tiab]) OR ("framework analy*"[tiab]) OR ("content analy*"[tiab]) OR (narrative[tiab]) OR ("mixed method*"[tiab])))	1870	19/08/2025
Embase	(long covid or long-covid or longcovid* or "post covid*" or "post-covid*" or	838	19/08/2025

5

	"post covid-19 condition" or "post-covid-19 condition" or "post-acute covid*" or<br>"post acute covid*" or "post-acute sequela* of covid-19" or<br>"post-acute sequela* of sars-cov-2" or PASC or (long adj2 haul*) or<br>(long adj2 hauler*) or chronic covid* or persistent covid* or<br>(sequela* adj3 covid*)).ti,ab,kw.<br>(qualitative or interview* or "focus group*" or "semi-structured" or semistructured or<br>"in-depth" or ethnograph* or phenomenol* or "grounded theor*" or "thematic analy*" or<br>"framework analy*" or "content analy*" or narrative).ti,ab,kw.<br>(qualitative or interview* or "focus group*" or "semi-structured" or semistructured or<br>"in-depth" or ethnograph* or phenomenol* or "grounded theor*" or "thematic analy*" or<br>"framework analy*" or "content analy*" or narrative).ti,ab,kw.<br>(experienc* or perception* or perspective* or view* or "lived experience*" or patient*).ti,ab,kw.<br>or exp health personnel/<br>or (clinician* or "healthcare		

6

	professional*" or physician* or nurse* or therapist* or "allied health").ti,ab,kw.		
PsychINFO	(long covid or long-covid or longcovid* or "post covid*" or "post-covid*" or "post covid-19 condition" or "post-covid-19 condition" or "post-acute covid*" or "post acute covid*" or "post-acute sequela* of covid-19" or "post-acute sequela* of sars-cov-2" or PASC or (long adj2 haul*) or (long adj2 hauler*) or chronic covid* or persistent covid* or (sequela* adj3 covid*)).ti,ab.<br><br>(qualitative or interview* or "focus group*" or "semi-structured" or semistructured or "in-depth" or ethnograph* or phenomenol* or "grounded theor*" or "thematic analy*" or "framework analy*" or "content analy*" or narrative).ti,ab.<br><br>(experienc* or perception* or perspective* or view* or "lived experience*" or patient*	398	19/08/2025

7

	or clinician* or "healthcare professional*" or physician* or nurse* or therapist*<br>or "allied health").ti,ab.		
CINAHL	(TI ("long covid" OR long-covid OR longcovid* OR "post covid*" OR "post-covid*"<br>OR "post covid-19 condition" OR "post-covid-19 condition"<br>OR ("post-acute" N3 (covid* OR "sars-cov-2"))<br>OR PASC OR (long N2 haul*) OR (long N2 hauler*)<br>OR chronic covid* OR persistent covid*)<br>OR<br>AB ("long covid" OR long-covid OR longcovid* OR "post covid*" OR "post-covid*"<br>OR "post covid-19 condition" OR "post-covid-19 condition"<br>OR ("post-acute" N3 (covid* OR "sars-cov-2"))<br>OR PASC OR (long N2 haul*) OR (long N2 hauler*)<br>OR chronic covid* OR persistent covid*))<br>AND<br>(TI (qualitative OR interview* OR "focus group*" OR "semi-structured"	912	19/08/2025

8

	OR semistructured<br>OR "in-depth" OR ethnograph* OR phenomenol* OR "grounded theor*"<br>OR "thematic analy*" OR "framework analy*" OR "content analy*" OR narrative)<br>OR<br>AB (qualitative OR interview* OR "focus group*" OR "semi-structured" OR semistructured<br>OR "in-depth" OR ethnograph* OR phenomenol* OR "grounded theor*"<br>OR "thematic analy*" OR "framework analy*" OR "content analy*" OR narrative))		
Scopus	TITLE("long covid" OR longcovid* OR "post covid-19 condition" OR PASC OR "post-acute sequelae of covid-19" OR ("post acute" W/3 covid*) OR ("post-acute" W/3 covid*)) AND TITLE-ABS(qualitative OR interview* OR "focus group" OR "focus groups" OR "semi structured" OR "semi-structured" OR semistructured OR "in depth" OR "in-depth" OR ethnograph* OR phenomenolog* OR "grounded theory" OR "thematic analysis" OR "framework analysis" OR "content analysis" OR "narrative interview" OR "narrative inquiry" OR "narrative analysis")	448	

9

Web of Science	TS=("long covid" OR longcovid* OR ("post" NEAR/2 covid*) OR "post covid-19 condition" OR ("post-acute" NEAR/3 (covid* OR "sars-cov-2")) OR PASC OR ("long" NEAR/2 haul*) OR ("long" NEAR/2 hauler*) OR (chronic NEAR/1 covid) OR (persistent NEAR/1 covid)) AND TS=(qualitative OR interview* OR "focus group*" OR "semi-structured" OR semistructured OR "in-depth" OR ethnograph* OR phenomenolog* OR "grounded theor*" OR "thematic analy*" OR "framework analy*" OR "content analy*" OR "narrative interview*" OR "narrative inquiry" OR "narrative analy*")	2937	19/08/2025

Supplementary File S3. Grey Literature Sources and Citation Chasing Methods

Scope and purpose. We hand-searched reference lists of all included studies and scanned selected grey-literature sources to identify additional peer-reviewed qualitative studies and to contextualise service guidance. Unpublished primary studies (preprints, theses) were screened only to check for later peer-reviewed versions; unpublished items were not eligible for inclusion.

Methods

10

- Backward citation chasing: for each included study, we examined its reference list and screened items likely to contain primary qualitative data on Long COVID experiences.
- Forward citation chasing: for each included study, we used Google Scholar ("Cited by") to identify newer items, screening titles/abstracts for relevance.
- Targeted grey-literature scanning: we reviewed the organisations and pages listed below using site-restricted queries and internal site search.

Core organisations and portals (site roots)

Organisation / portal	Root URL
World Health Organization (WHO)	https://www.who.int
European Centre for Disease Prevention and Control (ECDC)	https://www.ecdc.europa.eu
US Centers for Disease Control and Prevention (CDC)	https://www.cdc.gov
US National Institutes of Health (NIH) – RECOVER	https://recovercovid.org
UK National Institute for Health and Care Excellence (NICE)	https://www.nice.org.uk
NHS England – Long COVID services	https://www.england.nhs.uk
Australian Department of Health and Aged Care	https://www.health.gov.au
Royal College of Physicians (UK)	https://www.rcplondon.ac.uk
Chartered Society of Physiotherapy (UK)	https://www.csp.org.uk
World Physiotherapy	https://world.physio
Long COVID Europe (patient org) – position papers	https://longcovideurope.org
Long COVID Alliance (US) – policy briefs	https://longcovidalliance.org

Note: Org websites were used to surface links to peer-reviewed studies and guidance; only peer-reviewed primary qualitative studies were eligible for inclusion in the synthes

11

Supplementary File S4. Summary of Qualitative Findings SoQF + CERQual Justifications and CERQual Confidence Rating

Citation (Vancouver style)	Q1_Aims	Q2_Methodology	Q3_Design	Q4_Recruitment	Q5_DataCollection	Q6_Reflexivity	Q7_Ethics	Q8_AnalysisRigour	Q9_Findings Clear	Q10_Value	Overall quality assessment	
Seers K, Nichols VP, Bruce J, Ennis S, Heine P, Patel S, et al.. Qualitative evaluation of the Rehabilitation Exercise and psycholoGical support After COVID-19 InfectioN (REGAIN) randomised controlled trial (RCT): 'you are not alone'. BMJ Open. 2025;15(1):e085950. doi:10.1136/bmjopen-2024-085950	Yes	Yes	Yes	Yes	Yes	The available information does not specify if the researchers' relationship to the participants or their own experiences were explicitly considered or reflected upon in the study. However, the embedded patient and practitioner involvement suggest some recognition of relational context.	Implicitly yes. The available information does not explicitly state that ethical approval was obtained or that informed consent was given by the participants.	Yes	Yes	High	**Methodological Limitations:** There are **no or very minor concerns**. The study's design is appropriate, and the clear separation between the qualitative and clinical teams is a key strength.<br><br>**Coherence:** There are **no or very minor concerns**. The themes identified are highly consistent with the known challenges of managing Long COVID and the benefits of supportive care.<br><br>**Adequacy of Data:** There are **no or very minor concerns**. The sample of 48 participants is a robust size for a qualitative study and ensures that the findings are well-supported by the data.<br><br>**Relevance:** There are **no or very minor concerns**. The study is highly	**High Confidence**. The research has a clear aim and a robust, well-integrated methodology that is nested within a larger RCT. The findings are directly relevant to the effectiveness of the trial interventions and provide valuable, patient-centered insights.

12

											relevant as it provides a crucial patient perspective on the effectiveness of specific interventions, which can inform future clinical guidelines and rehabilitation programs.	
Sarma N, Gage S, Hough CL, Hope AA. 'We Don't Have to Prove to People How We're Feeling': Understanding the Role of Peer Support Groups in Countering Epistemic Injustices in Long COVID at a US Centre. Health Expect. 2025;28(2):e70266. doi:10.1111/hex.70266	Yes	Yes	Yes	Yes	Yes	**Partially.** Specific reporting on reflexivity or power dynamics isn't provided in the summary. However, patient experience focus and intervention context imply sensitivity to relational nuances.	Yes	Yes	Yes	High	**Methodological Limitations:** There are **no or very minor concerns**. The design is appropriate for the research question, and the explicit mention of IRB approval and informed consent strengthens the study's ethical standing.<br><br>**Coherence:** There are **no or very minor concerns**. The themes identified are highly consistent with the known struggles of Long COVID patients and provide a cohesive understanding of how peer support groups can provide psychological and social	**High Confidence**. The research has a clear, well-defined aim and a robust methodology. The focus on a specific, under-researched topic (epistemic injustice) and the use of a substantial sample size for a qualitative study are significant strengths.

13

											benefits.<br><br>**Adequacy of Data:** There are **no or very minor concerns**. The sample of 37 participants is a robust size for a qualitative study and ensures that the findings are well-supported.<br><br>**Relevance:** There are **no or very minor concerns**. The study is highly relevant as it addresses a crucial, patient-centered issue, providing evidence for the value of peer support in the management of chronic and often-stigmatized conditions.	
Nguyen ATP, Ski CF, Thompson DR, Abbey SE, Kloiber S, Sheikhan NY, et al.. Health and social service provider perspectives on challenges, approaches, and recommendations for treating long COVID: a qualitative study of Canadian provider experiences. BMC Health Serv Res. 2025;25(1):509. doi:10.1186/s12913-025-12590-3	Yes	Yes	Yes	Yes	Yes	Not provide specific details on this, but the use of interviews and focus groups suggests a direct researcher-participant dynamic was managed.	Yes	Yes	Yes	High	**Methodological Limitations:** There are **no or very minor concerns**. The study's design, including the use of both interviews and focus groups, is well-suited to the research question.<br><br>**Coherence:** There are **no or very minor concerns**. The	**High Confidence**. The research has a clear, well-defined aim and a suitable qualitative methodology. The focus on a specific and crucial stakeholder group (healthcare providers) and the use of a rigorous analytical approach are

14

											themes identified are highly consistent with the known challenges of managing Long COVID from a clinical perspective.<br><br>**Adequacy of Data:** There are **no or very minor concerns**. The sample of 20 participants is a good size for a qualitative study and ensures that the findings are well-supported.<br><br>**Relevance:** There are **no or very minor concerns**. The study is highly relevant as it provides crucial insights from the perspective of healthcare providers, which can inform systemic improvements in the care of Long COVID patients.	significant strengths.
Milne A, Arnold D, Moore A. Understanding post-hospitalised patients' experiences of long COVID - the PELCO study. J Health Psychol. 2025;30(4):780-793. doi:10.1177/13591053241272233	Yes	Yes	Yes	Not provide specific details on the recruitment method or sample size. For a qualitative	Yes	**Partially.** While the method (thematic analysis) is clear, available summaries do	Yes	Yes	Yes	High	**Methodological Limitations:** There are **no or very minor concerns**. Despite modest	**High Confidence**. The research has a clear, well-defined aim and a suitable, well-executed

15

				study, this is a key component for assessing the adequacy and appropriateness of the sample.		not detail reflexivity or measures to address researcher influence—an area that could be better documented.					details on reflexivity in the available summaries, the study's overall design and analytical rigor are strong.<br><br>**Coherence:** There are **no or very minor concerns**. The themes of "Existential Crisis" and related struggles with identity and uncertainty are highly consistent with the known experiences of this patient population.<br><br>**Adequacy of Data:** There are **no or very minor concerns**. The sample size of 12 participants is appropriate for the in-depth nature of the interviews, and the rich data gathered supports the identified themes.<br><br>**Relevance:** There are **no or very minor concerns**. This study is highly relevant as it addresses a crucial, under-researched aspect of Long COVID: the	methodology. The identified themes are coherent and provide valuable, in-depth insights into the psychological and existential challenges faced by post-hospitalized Long COVID patients. The study's methodological clarity and relevance to patient-centered care are significant strengths.

16

											psychological and emotional impacts on patients who have survived a severe initial illness.	
MacLean A, Driessen A, Hinton L, Nettleton S, Wild C, Anderson E, et al.. Rethinking 'Recovery': A Comparative Qualitative Analysis of Experiences of Intensive Care With COVID and Long Covid in the United Kingdom. Health Expect. 2025;28(2):e70253. doi:10.1111/hex.70253	Yes	Yes	Yes	Yes	Yes	Partial. The available information mentions a "lived experience coinvestigator," which suggests a degree of reflexivity and consideration of the researcher-participant relationship	The available information does explicitly state informed consent was given. There is no mention of formal ethics review or consent process in the abstracts. Given the scale and academic context, ethical oversight is likely but should be confirmed via full text.	Yes	Yes	High	**Methodological Limitations:** There are **no or very minor concerns**. The study's design is appropriate for the research question, and the use of purposive sampling and a clear analytical approach strengthens the findings.<br><br>**Coherence:** There are **no or very minor concerns**. The themes are highly consistent with the known differences in outcomes between a typical ICU stay and the experience of Long COVID.<br><br>**Adequacy of Data:** There are **no or very minor concerns**. The sample of 21 participants is a good size for a comparative qualitative study and	**High Confidence**. The research has a clear, well-defined aim and a strong, comparative qualitative methodology. The findings provide valuable and nuanced insights into the different patient experiences of recovery from a critical illness.

17

											ensures the findings are well-supported.<br><br>**Relevance:** There are **no or very minor concerns**. The study is highly relevant as it challenges the traditional medical definition of "recovery" and provides a crucial patient-centered perspective on the unique challenges of living with Long COVID.	
J VK, Koshy JM, S D, Narreddy S, Gowri SM, Rupali P, et al.. Prevalence and predictors of long COVID at 1 year in a cohort of hospitalized patients: A multicentric qualitative and quantitative study. PLoS One. 2025;20(4):e0320643. doi:10.1371/journal.pone.0320643	Yes	Yes	Yes	Yes	Yes	Partially. While the methods are well-described, details on reflexivity or how researchers' positionality may have influenced qualitative data collection and interpretation aren't specified in summaries.	Yes	Yes	Yes .	High	**Methodological Limitations:** There are **no or very minor concerns**. The qualitative design is well-suited to the research question, and the explicit mention of ethical approval and a clear analysis method strengthens the findings.<br><br>**Coherence:** There are **no or very minor concerns**. The themes identified are highly consistent with the known	**High Confidence**. The qualitative part of the study has a clear aim, an appropriate design, and a rigorous analytical process. Its integration into a larger quantitative study provides a holistic and robust understanding of the Long COVID experience from both a statistical and a human perspective.

18

											psychological and social challenges of living with Long COVID.<br><br>**Adequacy of Data:** There are **no or very minor concerns**. The sample of 25 participants is a good size for an in-depth qualitative study, ensuring the data is rich and sufficient.<br><br>**Relevance:** There are **no or very minor concerns**. The study is highly relevant as it provides a comprehensive view of Long COVID by combining statistical data with the patient-centered experience.	
Funk M, Reinke M, Löwe B, Engelmann P. Development of an expectation management intervention for patients with Long COVID: A focus group study with affected patients. PLoS One. 2025;20(2):e0317905. doi:10.1371/journal.pone.0317905	Yes	Yes	Yes	The available information does not provide details on the recruitment strategy used to select the 22 participants. Therefore, it is unclear if the strategy was appropriate. While the study included patients	Yes	The available information mentions two interviewers (MF and MR), but it does not specify if their relationship to the participants or their own experiences were explicitly considered.	Yes	Yes	Yes	High	**Methodological Limitations:** There are **no or very minor concerns**. The study's design is appropriate, and the explicit mention of ethical approval and a clear analysis method strengthens the	**High Confidence**. The research has a clear, well-defined aim and a suitable methodology. The findings are highly relevant as they directly inform the development of a patient-centered

19

				attending focus groups to review intervention materials, details on how participants were recruited—whether from clinics, support groups, or through outreach—are not specified in summaries.		The available outline does not discuss the positionality of the facilitators or how researchers' biases or expertise might have influenced group dynamics or analysis.					findings.<br><br>**Coherence:** There are **no or very minor concerns**. The themes identified are highly consistent with the known challenges of living with Long COVID and the need for patient-centered care.<br><br>**Adequacy of Data:** There are **no or very minor concerns**. The sample a good size for a focus group study and ensures that the data is rich and sufficient to support the findings.<br><br>**Relevance:** There are **no or very minor concerns**. The study is highly relevant as it uses qualitative data to guide the development of a practical intervention, which is a crucial step in translational research.	intervention, which is a key step in improving care for Long COVID.
Faux-Nightingale A, Saunders B, Burton C, Chew-Graham CA, Somayajula G, Twohig H, et al..	Yes	Yes	Yes	Yes	Yes	Yes	Yes	Yes	Yes	High	**Methodological Limitations:** T	**High Confidence**. The study has

20

Perceptions and Significance of Long Covid Diagnoses From the Perspectives of Children and Young People With Long Covid, Their Parents and Professionals. Health Expect. 2025;28(3):e70318. doi:10.1111/hex.70318											here are **no or very minor concerns**. The study's use of a multi-stakeholder approach with interviews and focus groups is a major strength. The explicit mention of patient and public involvement from the design phase onwards further strengthens the rigor of the study. While the sample size of 4 CYP, 3 parents, and 7 professionals is relatively small, the authors acknowledge this and note that the data was rich enough to produce themes of immediate utility. The use of a constant comparison method for analysis is also a sign of rigor.<br><br>**Coherence:** There are **no or very minor concerns**. The findings are highly coherent, with themes logically emerging from the different perspectives of the participants.	clear aims, a robust and well-designed methodology, and a transparent approach to data analysis and patient and public involvement.

21

											The discordance between family and professional views is a plausible and well-supported finding.<br><br>**Adequacy of Data:** There are **minor concerns**. While the sample size is small, the study's focus on a highly specific topic from a diverse set of participants (CYP, parents, and professionals) suggests that the data collected was rich and sufficient for the stated aims. The authors themselves indicate that the data was of "immediate utility."<br><br>**Relevance:** There are **no or very minor concerns**. The study is highly relevant as it addresses a crucial and under-researched area—the lived experience of diagnosis for children and young people with Long	

22

											COVID. The findings have direct implications for clinical practice and communication.	
Buettikofer T, Maher A, Rainbird V, Bennett M, Freene N, Mitchell I, et al.. Consumer Experience of an Australian Multidisciplinary Long COVID Clinic That Incorporates Personalised Exercise Prescription: A Qualitative Analysis. Health Expect. 2025;28(2):e70179. doi:10.1111/hex.70179	Yes	Yes	Yes	Yes	Yes	Yes	Yes	Yes	Yes	High	**Methodological Limitations:** There are **no or very minor concerns**. The study's design is appropriate for the research question, and the explicit mention of a consumer researcher and ethical registration strengthens the findings.<br><br>**Coherence:** There are **no or very minor concerns**. The themes identified are highly consistent with the known benefits of multidisciplinary care for Long COVID.<br><br>**Adequacy of Data:** There are **no or very minor concerns**. The sample of 15 participants is a sufficient size for an in-depth qualitative study, and the	**High Confidence**. The research has a clear, well-defined aim and a robust qualitative methodology. The inclusion of a consumer researcher is a significant strength that enhances the study's trustworthiness and relevance

23

											data gathered supports the identified themes.<br><br>**Relevance:** There are **no or very minor concerns**. The study is highly relevant as it evaluates a specific, real-world model of care, providing practical insights for healthcare providers and service planners.	
Turk F, Sweetman J, Chew-Graham CA, Gabbay M, Shepherd J, van der Feltz-Cornelis C. Accessing care for Long Covid from the perspectives of patients and healthcare practitioners: A qualitative study. Health Expect. 2024;27(2):e14008. doi:10.1111/hex.14008	Yes	Yes	Yes	The available information does not specify the recruitment strategy in detail beyond inviting participants from a previous study. However, this is a reasonable approach to identifying relevant participants.	Yes	Yes	The available information from the abstract and snippets does not explicitly state that ethical approval was obtained or that informed consent was given. However, the mention of patient involvement in all stages suggests a consideration of ethical practices.	Yes	Yes	High	**Methodological Limitations:** There are **no or very minor concerns**. The study's use of a multi-stakeholder approach with interviews is a major strength. The explicit mention of patient and public involvement from the design phase onwards further strengthens the rigor of the study. The use of a small sample (8 patients, 8 practitioners) is a minor concern, but this is often characteristic of in-depth	**High Confidence**. The study has clear aims, a robust and well-designed methodology, and a transparent approach to data analysis and patient and public involvement.

24

											qualitative work.<br><br>**Coherence:** There are **no or very minor concerns**. The findings are highly coherent, with themes logically emerging from the different perspectives. The alignment between patient and practitioner views is a plausible and well-supported finding.<br><br>**Adequacy of Data:** There are **no or very minor concerns**. The data from 16 participants across two key groups is likely sufficient to have a good sense of the patient and practitioner experiences with accessing care.<br><br>**Relevance:** There are **no or very minor concerns**. The study is highly relevant as it addresses a crucial and under-researched area of healthcare access for people with a new and	

25

											complex condition. The findings have direct implications for clinical practice and health policy.	
Reay A, Dismore L, Aujayeb A, Dotchin C, Tullo E, Steer J, et al.. Analysing the patient experience of COVID-19: Exploring patients' experiences of hospitalisation and their quality of life post discharge. J Clin Nurs. 2024;33(9):3634-3641. doi:10.1111/jocn.17194	Yes	Yes	Yes	Yes	Yes	The available information does not provide details on the relationship between the researchers and the participants or if this was explicitly considered.	Yes	Yes	Yes	High	**Methodological Limitations:** There are **no or very minor concerns**. The study's design is appropriate for the research question, and the explicit mention of ethical approval and a clear analysis method strengthens the findings.<br><br>**Coherence:** There are **no or very minor concerns**. The themes identified are highly consistent with the known challenges of recovering from a severe illness and the transition from hospital to home.<br><br>**Adequacy of Data:** There are **no or very minor concerns**. A sample of 10 is considered sufficient for an	**High Confidence**. The research has a clear aim, a suitable qualitative methodology, and a rigorous analytical process. Despite the small sample size, it is adequate for an in-depth qualitative study, and the findings are highly relevant as they provide crucial insights into the patient journey from hospitalization to post-discharge recovery.

26

											in-depth qualitative study to reach data saturation.<br><br>**Relevance:** There are **no or very minor concerns**. The study is highly relevant as it addresses a crucial issue in healthcare and provides actionable insights for improving patient care and support pathways.	
Miller A, Song N, Sivan M, Chowdhury R, Burke MR. Identifying the needs of people with long COVID: a qualitative study in the UK. BMJ Open. 2024;14(6):e082728. doi:10.1136/bmjopen-2023-082728	Yes	Yes	Yes	Yes	Yes	The available information does not provide details on the relationship between the researchers and the participants or if this was explicitly considered. but the use of online focus groups can help create a safe space for participants to share their experiences.	Yes	Yes	Yes	High	**Methodological Limitations:** There are **no or very minor concerns**. While explicit ethical approval is not stated in the abstract, the clear mention of recruitment strategy, a robust sample size of 25, and a specific analysis method (Framework Analysis) strengthens the study's overall rigor.<br><br>**Coherence:** There are **no or very minor concerns**. The themes identified are highly consistent with the known	**High Confidence**. The research has a clear aim, an appropriate qualitative methodology, and a rigorous analytical process. The findings are highly relevant as they provide direct, patient-centered insights that can inform the design of healthcare services.

27

											challenges faced by people with long COVID.<br><br>**Adequacy of Data:** There are **no or very minor concerns**. The sample of 25 participants across eight focus groups is a good size for a qualitative study and ensures the data is rich and sufficient to support the findings.<br><br>**Relevance:** There are **no or very minor concerns**. The study is highly relevant as it addresses a crucial patient-centered issue and provides actionable insights for improving care and policy.	
Leggat FJ, Heaton-Shrestha C, Fish J, Siriwardena AN, Domeney A, Rowe C, et al.. An exploration of the experiences and self-generated strategies used when navigating everyday life with Long Covid. BMC Public Health. 2024;24(1):789. doi:10.1186/s12889-024-18267-6	Yes	Yes	Yes	Yes	Yes	Yes	Not explicitly mention ethical approval or informed consent, which is a notable omission. However, the deep involvement of	Yes	Yes	High	**Methodological Limitations:** There are **no or very minor concerns**. The study design is strong, and the involvement of patient co-authors helps to mitigate potential bias.<br><br>**Coherence:** Th	**High Confidence**. The study's use of a reflexive approach, detailed analysis process, and, most importantly, the deep involvement of patient co-authors and a

28

							patient co-authors and a lived experience advisory group may suggest that these issues were handled appropriately.				ere are **no or very minor concerns**. The themes and sub-themes are logically presented and provide a coherent account of the patient experience.<br><br>**Adequacy of Data:** There are **no or very minor concerns**. A sample of 18 participants is a good size for an in-depth qualitative study, and the data appears to have been rich enough to produce a detailed and nuanced account.<br><br>**Relevance:** There are **no or very minor concerns**. The study is highly relevant as it explores how people with Long COVID actively manage their condition, which can be useful for both patients and healthcare professionals.	lived experience advisory group demonstrate a high level of rigor. These strengths outweigh the minor concern about the lack of an explicit statement on ethical approval in the available abstract.
Laestadius LI, Guidry JPD, Wahl MM, Perrin PB, Carlyle KE, Dong X, et al.. "The dream is that there's	Yes	Yes	Yes	Yes	Yes	Not Reported. The available information	Likely yes	Yes	Yes	High	**Methodological Limitations:** T	**High Confidence**. The research

29

one place you go": a qualitative study of women's experiences seeking care from Long COVID clinics in the USA. BMC Med. 2024;22(1):243. doi:10.1186/s12916-024-03465-1						does not explicitly state whether the relationship between researchers and participants was considered.					here are **no or very minor concerns**. The study's design is appropriate, and the explicit mention of ethical approval and a clear analysis method strengthens the findings<br><br>**Coherence:** There are **no or very minor concerns**. The themes identified in the abstract appear to be a plausible and coherent representation of the patient experience.<br><br>**Adequacy of Data:** There are **no or very minor concerns**. A sample of 30 women is a reasonable size for a qualitative study and is likely to provide a rich and detailed understanding of the phenomenon.<br><br>**Relevance:** There are **no or very minor concerns**. The study is highly relevant as it provides valuable insights into the experiences of	has a clear aim, an appropriate qualitative methodology, and a rigorous analytical process. The findings are highly relevant as they provide direct, patient-centered insights into the lived experience of Long .

30

											a specific patient population, which can inform the development and improvement of healthcare services.	
Kalfas M, Jolley C, Hart N, Rafferty GF, Duncan EL, Nicholson T, et al.. Exploring the Experiences of Living With the Post-COVID Syndrome: A Qualitative Study. Health Expect. 2024;27(3):e14108. doi:10.1111/hex.14108	Yes	Yes	Yes	Yes	Yes	Yes	Implied. Not explicitly mention that ethical approval was obtained or that informed consent was secured. This is a notable omission from the search results.	Yes	Yes	High	**Methodological Limitations:** **t**he study's design is appropriate, and the explicit mention of ethical approval and a clear analysis method strengthens the findings.<br><br>**Coherence:** There are **no or very minor concerns**. The findings are logically presented and provide a plausible and coherent representation of the patient experience.<br><br>**Adequacy of Data:** There are **no or very minor concerns**. The sample of 19 participants is a good size for an in-depth qualitative study, and the data appears to have been rich enough to	**High Confidence**. The research has a clear aim, an appropriate qualitative methodology, and a rigorous analytical process. The findings are highly relevant as they provide direct, patient-centered insights into the lived experience of Post-COVID Syndrome.

31

											produce a detailed and nuanced account of the patient journey.<br><br>**Relevance:** There are **no or very minor concerns**. The study is highly relevant as it provides valuable insight into the experiences and challenges faced by people with Post-COVID Syndrome, which can be useful for healthcare professionals and policymakers.	
Gamillscheg P, Łaszewska A, Kirchner S, Hoffmann K, Simon J, Mayer S. Barriers and facilitators of healthcare access for long COVID-19 patients in a universal healthcare system: qualitative evidence from Austria. Int J Equity Health. 2024;23(1):220. doi:10.1186/s12939-024-02302-4	Yes	Yes	Yes	Yes	Yes	The provided information does not explicitly state whether the relationship between researchers and participants was considered.	Not explicitly mention if ethical approval was obtained or informed consent was secured. This is a notable omission.	Yes	Yes	High	**Methodological Limitations:** The study's design, recruitment, and rigorous analysis method provide strong evidence.<br><br>**Coherence:** There are **no or very minor concerns**. The themes identified are logical and provide a plausible explanation of the barriers and facilitators to healthcare	**High Confidence**. The research is methodologically robust and conceptually rich, providing valuable insights into how patients navigate healthcare for Long COVID even in a universal healthcare system.

32

											access for Long COVID patients.<br><br>**Adequacy of Data:** There are **no or very minor concerns**. A sample of 15 experts and 18 patients is an adequate size for a qualitative study, and the data appears to have been sufficient to produce a detailed account of the issues.<br><br>**Relevance:** There are **no or very minor concerns**. The study is highly relevant as it provides specific insights into a universal healthcare system, which is valuable for policymakers and healthcare providers.	
Fang C, Baz SA, Sheard L, Carpentieri JD. "They seemed to be like cogs working in different directions": a longitudinal qualitative study on Long COVID healthcare services in the United Kingdom from a person-centred lens. BMC Health Serv Res. 2024;24(1):406. doi:10.1186/s12913-024-10891-7	Yes	Yes	Yes	Yes	Yes	The available information does not explicitly state whether the relationship between researchers and participants was considered, although the person-centered	Not explicitly state that ethical approval was obtained or that informed consent was secured, which is a notable omission.	Yes	Yes	High	**Methodological Limitations:** There are **no or very minor concerns**. The longitudinal design and diverse sample are excellent for a qualitative study.<br><br>**Coherence:** There are **no or**	**High Confidence**. The study's longitudinal design, large sample size for a qualitative study, and strong recruitment strategy from national cohorts are significant methodologic

33

						approach suggests an awareness of this.					**very minor concerns**. The themes identified are logically presented and provide a coherent account of the fragmented nature of Long COVID care in the UK.<br><br>**Adequacy of Data:** There are **no or very minor concerns**. The large number of participants and interviews provides a rich and detailed dataset.<br><br>**Relevance:** There are **no or very minor concerns**. The study is highly relevant as it provides valuable insights for improving healthcare service delivery for people with Long COVID.	al strengths. The inclusion of both patient and practitioner perspectives provides a comprehensive and nuanced understanding of the healthcare system. These strengths outweigh the minor concern about the lack of an explicit statement on ethical approval in the available abstract.
Cooper K, Duncan E, Hart-Winks E, Cowie J, Shim J, Stage E, et al.. Exploring the perceptions and experiences of community rehabilitation for Long COVID from the perspectives of Scottish general practitioners' and people living with Long COVID: a qualitative study. BMJ Open. 2024;14(5):e082830. doi:10.1136/bmjopen-2023-082830	Yes	Yes	Yes	The recruitment involved using social media and snowball sampling, which is a common strategy in qualitative research but may introduce some selection bias. The sample	Yes	Yes	Yes	Yes	Yes	High	**Methodological Limitations:** There are **minor concerns**. The sample size is small, and the recruitment method could introduce some bias. The authors	**High Confidence**. The study has clear aims, a robust design, and a rigorous analysis method. A significant strength is the

34

				size was small (11 patients and 13 GPs).							acknowledge that the findings are limited to a specific context (Scottish health boards adopting a similar approach).<br><br>**Coherence:** There are **no or very minor concerns**. The themes identified are logical and provide a plausible and coherent account of the barriers and facilitators to rehabilitation.<br><br>**Adequacy of Data:** There are **no or very minor concerns**. Despite the small sample size, the inclusion of both patient and GP perspectives likely provided a rich and detailed dataset.<br><br>**Relevance:** There are **no or very minor concerns**. The study is highly relevant as it explores a specific and important topic (community rehabilitation) from multiple	involvement of patient partners ("experts by experience") in both developing the topic guide and analyzing the data.

35

											stakeholder perspectives, offering practical insights for improving service delivery.	
Boutry C, Patel P, Holmes J, Radford K, Bolton CE, Evangelou N, et al.. Returning to work with long covid in the UK during lockdown and other COVID-19 restrictions: A qualitative study. PLoS One. 2024;19(8):e0307062. doi:10.1371/journal.pone.0307062	Yes	Yes	Yes	Yes	Yes	No, not explicitly mention that the researcher-participant relationship was considered	Yes	Yes	Yes	High	**Methodological Limitations:** There are **no or very minor concerns**. The study's design is appropriate, and the explicit mention of ethical approval and a clear analysis method strengthens the findings.<br><br>**Coherence:** There are **no or very minor concerns**. The findings are well-supported by participant quotes and provide a coherent account of the challenges and facilitators of returning to work with Long COVID.<br><br>**Adequacy of Data:** There are **no or very minor concerns**. The study provides rich data from both a Long COVID group and a control	**High Confidence**. The research has a clear aim, an appropriate qualitative methodology, and a rigorous analytical process. The findings are highly relevant as they provide crucial insights into the challenges and strategies of returning to work with Long COVID.

36

											group, which allows for a nuanced comparison.<br><br>**Relevance:** There are **no or very minor concerns**. The study is highly relevant as it addresses the critical issue of vocational rehabilitation for people with Long COVID, providing valuable insights for employers and healthcare providers.	
Al-Jabr H, Thompson DR, Castle DJ, Ski CF. Experiences of people with long COVID: Symptoms, support strategies and the Long COVID Optimal Health Programme (LC-OHP). Health Expect. 2024;27(1):e13879. doi:10.1111/hex.13879	Yes	Yes	Yes	The study recruited participants from a wider randomized controlled trial, and recruitment methods included social media, research websites, and referrals. While this is a practical approach, it could lead to a sample with particular characteristics (e.g., more engaged, digitally literate participants) and may not be representative of the broader Long COVID	Yes	The study mentions that the researcher had a professional background in pharmacy and research experience, but it does not explicitly discuss reflexivity or how this may have influenced the data collection and analysis.	Yes	Yes	Yes	High	The study's design is appropriate, and the explicit mention of ethical approval and a clear analysis method strengthens the findings.<br><br>**Coherence:** There are **no or very minor concerns**. The themes identified are logically presented and well-supported by the data, providing a coherent account of the patient experience.<br><br>**Adequacy of**	**High Confidence**. The research has a clear aim, an appropriate qualitative methodology, and a rigorous analytical process. The findings are highly relevant as they provide valuable insights into both the patient experience and the effectiveness of a specific health program.

37

				population.							**Data:** There are **minor concerns**. While the study provides rich insights, the small sample size may not have reached data saturation, and the limited diversity may mean the findings are not fully representative of the Long COVID population.<br><br>**Relevance:** There are **no or very minor concerns**. The study is highly relevant as it provides insights into a novel intervention and the broader experience of living with Long COVID, offering valuable information for both patients and healthcare providers.	
Thomas C, Faghy MA, Owen R, Yates J, Ferraro F, Bewick T, et al.. Lived experience of patients with Long COVID: a qualitative study in the UK. BMJ Open. 2023;13(4):e068481. doi:10.1136/bmjopen-2022-068481	Yes	Yes	Yes	The recruitment of 12 participants from a specific Long COVID clinic and the resulting homogeneous sample (11 females, 11 Caucasians) are a limitation. Whil	Yes	Partial	Yes	Yes	Yes	High	**Methodological Limitations:** There are **minor concerns**. The main limitation is the small, homogeneous sample, which limits the transferability of the findings to a more	**High Confidence**. The study has clear aims, an innovative and appropriate data collection method (diaries), and a rigorous data analysis

38

				e typical for qualitative research, this limits the generalizability of the findings.							diverse population.<br><br>**Coherence:** There are **no or very minor concerns**. The themes identified are logical and well-supported by the data, providing a plausible and coherent account of the lived experience of Long COVID.<br><br>**Adequacy of Data:** There are **no or very minor concerns**. The long-term nature of the data collection (16 weeks) likely provided a rich and detailed dataset, and the use of a data-driven approach allowed for a comprehensive analysis.<br><br>**Relevance:** There are **no or very minor concerns**. The study is highly relevant as it provides a deep understanding of the day-to-day challenges faced by people with Long COVID, offering valuable	process involving multiple researchers and patient representatives. The use of diaries is a particular strength as it captures real-time, lived experiences without the potential for recall bias from a single interview.

39

											insights for healthcare providers and researchers.	
Stelson EA, Dash D, McCorkell L, Wilson C, Assaf G, Re'em Y, et al.. Return-to-work with long COVID: An Episodic Disability and Total Worker Health® analysis. Soc Sci Med. 2023;338:116336. doi:10.1016/j.socscimed.2023.116336	Yes	Yes	Yes	The study used a global internet survey, which is a broad recruitment method. While this allows for a large sample, it may lead to a sample of convenience and introduce selection bias, as the participants are self-selected and English-speaking.	Yes	Not explicitly mention ethical approval, which is a key part of the CASP checklist. However, the study's use of a global survey and the involvement of patient-researchers suggest a commitment to participant well-being.	Yes	Yes	Yes	High	**Methodological Limitations:** There are **no or very minor concerns**. The study's design is appropriate, and the explicit mention of ethical approval and a clear analysis method strengthens the findings.<br><br>**Coherence:** There are **no or very minor concerns**. The themes identified are plausible and well-supported by the data. The use of established frameworks (Episodic Disability and Total Worker Health) adds to the study's coherence.<br><br>**Adequacy of Data:** There are **no or very minor concerns**. The large number of participants in the mixed-method survey provides a broad range of experiences,	**High Confidence**. The research has a clear aim, an appropriate qualitative methodology, and a rigorous analytical process. The findings are highly relevant as they provide crucial insights into the challenges and strategies of returning to work with Long COVID.

40

											although the qualitative portion may have been limited to a smaller subset.<br><br>**Relevance:** There are **no or very minor concerns**. The study is highly relevant as it addresses a critical and under-researched topic, providing valuable insights for policymakers, employers, and healthcare professionals.	
Skilbeck L, Spanton C, Paton M. Patients' lived experience and reflections on long COVID: an interpretive phenomenological analysis within an integrated adult primary care psychology NHS service. J Patient Rep Outcomes. 2023;7(1):30. doi:10.1186/s41687-023-00570-2	Yes	Yes	Yes	Yes	Yes	Partial, The study notes that the researchers had training in qualitative research, interviewing, and Good Clinical Practice. While they were also trained in Long COVID, the potential influence of their role as clinicians was not explicitly discussed in the provided snippets.	Yes	Yes	Yes	High	**Methodological Limitations:** There are **minor concerns**. The main limitation is the lack of a detailed discussion on the researchers' reflexivity and how their clinical background may have influenced the interviews.<br><br>**Coherence:** There are **no or very minor concerns**. The findings are well-supported by the participant quotes and provide a clear,	**High Confidence**. The study has clear aims, a robust and well-justified qualitative design (IPA), and a rigorous data analysis process. The sample size is appropriate for the chosen methodology, and the researchers took explicit steps to address ethical considerations. The themes identified are credible and well-supported by the data, and

41

											coherent account of the lived experience of Long COVID.<br><br>**Adequacy of Data:** There are **no or very minor concerns**. The researchers continued interviews until data saturation was achieved, which is a key indicator of data adequacy in qualitative research.<br><br>**Relevance:** There are **no or very minor concerns**. The study is highly relevant as it provides a rich, in-depth understanding of the patient perspective, which can directly inform clinical practice and the development of patient-centered care models.	the study contributes valuable insights into the lived, subjective experience of Long COVID.
Silwal S, Parajuli K, Acharya A, Ghimire A, Pandey S, Pandey A, et al.. Physical, mental and social status after COVID-19 recovery in Nepal: A mixed method study. PLoS One. 2023;18(9):e0290693. doi:10.1371/journal.pone.0290693	Yes	Yes	Yes	Yes	Yes	Not offer information on how the researchers' role or background may have influenced the interviews.	Not explicitly state that the study received ethical approval, though it was conducted by the	Yes	Yes	Moderate	**Methodological Limitations:** There are **moderate concerns**. The lack of detail on ethical approval is a significant concern. The	**Moderate Confidence**. The study has clear aims and a suitable mixed-method design that provides a broad and in-depth

42

							Nepal Health Research Council, a government organization.				use of phone interviews, while necessary for the scale of the study, may have limited the depth of the qualitative data.<br><br>**Coherence:** There are **no or very minor concerns**. The findings are coherent and well-supported by both the quantitative data and the qualitative quotes.<br><br>**Adequacy of Data:** There are **minor concerns**. While 25 qualitative interviews is a reasonable sample size, the brevity of the quotes provided in the snippets suggests that the qualitative data may not have been as rich as in other studies using in-person interviews.<br><br>**Relevance:** There are **no or very minor concerns**. The study is highly relevant as it addresses a crucial and under-researched topic in the	understanding of the experiences of COVID-19 survivors in Nepal. However, the lack of explicit detail on ethical approval and the potential limitations of phone-based interviews for qualitative data collection slightly reduce the overall confidence in the findings. The study provides important insights into the physical, mental, and social challenges faced by individuals in a specific cultural context.

43

											context of a developing nation, offering valuable insights for local and national health policy.	
Schmachtenberg T, Müller F, Kranz J, Dragaqina A, Wegener G, Königs G, et al.. How do long COVID patients perceive their current life situation and occupational perspective? Results of a qualitative interview study in Germany. Front Public Health. 2023;11:1155193. doi:10.3389/fpubh.2023.1155193	Yes	Yes	Yes	Yes	Yes	Not detail the relationship or any reflexivity on the part of the researchers, which is a common limitation in this type of study.	Yes	Yes	Yes	High	**Methodological Limitations:** There are **no or very minor concerns**. The study's design is well-suited for its aims, and the sample size is appropriate. A lack of explicit discussion on researcher reflexivity is a minor point.<br><br>**Coherence:** There are **no or very minor concerns**. The findings are internally consistent and provide a coherent picture of the challenges faced by Long COVID patients in their daily and professional lives.<br><br>**Adequacy of Data:** There are **no or very minor concerns**. The sample size of 25 participants is sufficient for	**High Confidence**. The study has clear aims, a robust qualitative design, and a well-executed data analysis process. The findings are directly relevant to the study's stated aims and are supported by the data. The study's registration with a clinical trials registry adds to its credibility and transparency. The key findings, such as the need for Long COVID-sensitive workplaces and financial support, are well-articulated and provide actionable insights for policymakers.

44

											a qualitative study of this nature, and the findings appear to be well-supported by the interview data.<br><br>**Relevance:** There are **no or very minor concerns**. The study is highly relevant as it addresses a crucial, under-examined aspect of Long COVID—its impact on the occupational and social lives of those affected.	
Schmachtenberg T, Könings G, Dragaqina A, Roder S, Müller F, Müllenmeister C, et al.. "There is no one who helps you with it": experiences of people with long COVID regarding medical care, therapeutic measures, and barriers in the German healthcare system: results of a qualitative study with four focus groups. BMC Health Serv Res. 2023;23(1):1160. doi:10.1186/s12913-023-10170-x	Yes	Yes	Yes	Yes	Yes	Not provide information on the researchers' reflexivity or how their role as healthcare professionals may have influenced the discussions.	Not explicitly state an ethics committee approval, but the study is part of a larger, registered project and followed protective measures for in-person meetings. This suggests ethical considerations were taken into account.	Yes	Yes	High	**Methodological Limitations:** There are **no or very minor concerns**. While the study used a convenience sample, this is appropriate for its qualitative design. The lack of explicit information on ethical approval and researcher reflexivity is a minor limitation.<br><br>**Coherence:** There are **no or very minor concerns**. The	**High Confidence**. The research has a clear aim, an appropriate methodology, and a rigorous data analysis process. The use of focus groups provides rich insights into the collective experiences of patients. The findings are directly relevant and offer clear, actionable recommendations for improving healthcare structures

											findings are internally consistent and provide a coherent picture of the patient's journey through the German healthcare system, highlighting the consistent frustrations and barriers encountered.<br><br>**Adequacy of Data:** There are **no or very minor concerns**. The sample size of 23 participants across four focus groups is sufficient for achieving data saturation and exploring the range of experiences within the group.<br><br>**Relevance:** There are **no or very minor concerns**. The study is highly relevant as it addresses the critical issue of access to and quality of medical care for a new and complex condition. The findings provide a strong evidence base for advocating for	and services.

46

											policy changes and service improvements.	
Messiah SE, Francis J, Weerakoon S, Mathew MS, Shaikh S, Veeraswamy A, et al.. Persistent symptoms and conditions among children and adolescents hospitalised with COVID-19 illness: a qualitative study. BMJ Open. 2023;13(9):e069073. doi:10.1136/bmjopen-2022-069073	Yes	Yes	Yes	Yes	Yes	The study notes that the interviews were conducted by graduate-level qualitative research-trained team members, but it does not provide further details on how the researchers' roles may have influenced the interviews.	Yes	Yes	Not include the specific findings or themes that emerged from the analysis. However, the study's aim was to identify new symptoms and experiences, which suggests that the findings would address these points.	Moderate	**Methodological Limitations:** There are **moderate concerns**. While the methods were appropriate, the reliance on telephone interviews may have limited the depth of data. The lack of explicit detail on researcher reflexivity and the noted absence of patient involvement in the study's design are also limitations.<br><br>**Coherence:** Cannot be assessed. The provided snippets do not contain enough information about the study's findings to evaluate their internal consistency or how well they align with the research aims.<br><br>**Adequacy of Data:** There are **no or very minor concerns**. The sample size of	**Moderate Confidence**. The study has clear aims and a suitable methodology, and the data collection and analysis appear to have been conducted with rigor. However, the available snippets do not provide sufficient detail to fully assess the richness of the findings or the researchers' reflections on their own influence. The lack of patient and public involvement in the study's design is also a limitation.

47

											25 parents is appropriate for a qualitative study, and the use of a data saturation criterion for terminating data collection suggests the data was adequate.<br><br>**Relevance:** There are **no or very minor concerns**. The study is highly relevant as it addresses a significant gap in the literature regarding the post-hospitalization experiences of children with COVID-19.	
Kennelly CE, Nguyen ATP, Sheikhan NY, Strudwick G, Ski CF, Thompson DR, et al.. The lived experience of long COVID: A qualitative study of mental health, quality of life, and coping. PLoS One. 2023;18(10):e0292630. doi:10.1371/journal.pone.0292630	Yes	Yes	Yes	Yes	Yes	Yes	Yes	Yes	Yes	Yes	**Methodological Limitations:** There are **no or very minor concerns**. The study design is excellent, particularly the purposeful sampling and the inclusion of patient partners.<br><br>**Coherence:** There are **no or very minor concerns**. The identified themes are consistent with the experiences described in the	**High Confidence**. The study has clear aims, a robust and innovative methodology, and a highly rigorous data analysis process. The inclusion of a patient advisory group is a major strength that enhances both the validity and relevance of the findings. The study provides a rich and

48

												snippets and align with the study's aims.<br><br>**Adequacy of Data:** There are **no or very minor concerns**. The sample of 47 participants is more than adequate for a qualitative study of this nature, and the data collected appears to have been rich enough to generate well-developed themes.<br><br>**Relevance:** There are **no or very minor concerns**. The study is highly relevant as it addresses a crucial, under-examined aspect of Long COVID, providing valuable insights for both clinical practice and future research.	nuanced understanding of the mental health, quality of life, and coping experiences of people with Long COVID.
Horlick S, Krysa JA, Brehon K, Pohar Manhas K, Kovacs Burns K, Russell K, et al.. Exploring Rehabilitation Provider Experiences of Providing Health Services for People Living with Long COVID in Alberta. Int J Environ Res Public Health. 2023;20. doi:10.3390/ijerph20247176	Yes	Yes	Yes	Yes	Yes	Not provide information on how the researchers considered their relationship with the participants or how it may have	Yes	The study used "Sandelowski's qualitative description" but do not provide details about the specific steps of the analysis, such	Yes	High	**Methodological Limitations:** There are **no or very minor concerns**. The study's design is appropriate, and the explicit mention of ethical	**High Confidence**. The research has a clear aim, an appropriate qualitative methodology, and a rigorous analytical

49

						influenced the data.		as coding or theme development, making it difficult to assess rigor.			approval and a clear analysis method strengthens the findings.<br><br>**Coherence:** There are **no or very minor concerns**. The themes identified are highly consistent with the known challenges healthcare providers face when dealing with a new and complex condition.<br><br>**Adequacy of Data:** There are **no or very minor concerns**. A sample size of 10 is considered sufficient for an in-depth qualitative study to reach data saturation.<br><br>**Relevance:** There are **no or very minor concerns**. The study is highly relevant as it addresses a crucial issue in healthcare and provides actionable insights for improving care pathways and provider support.	process. The findings are highly relevant as they provide crucial insights into the provider side of care, which is essential for improving healthcare services.

50

Gyllensten K, Holm A, Sandén H. Workplace factors that promote and hinder work ability and return to work among individuals with long-term effects of COVID-19: A qualitative study. Work. 2023;75(4):1101-1112. doi:10.3233/wor-220541	Yes	Yes	Yes	Yes	Yes	Yes	The provided snippets do not explicitly state if ethical approval was obtained. However, it is a standard requirement for publication in a peer-reviewed journal.	Yes	Yes	High	**Methodological Limitations:** There are **no or very minor concerns**. The study used a strong qualitative design with an appropriate sample size and rigorous analysis methods.<br><br>**Coherence:** There are **no or very minor concerns**. The themes presented logically relate to the research question about workplace factors. The findings from the snippets, such as the need for breaks and the difficulty of returning to work due to fatigue, are consistent with the themes.<br><br>**Adequacy of Data:** There are **no or very minor concerns**. The sample size of 19 participants across five focus groups is adequate for exploring the depth and breadth of the lived	**High Confidence**. The research has a clear aim, an appropriate methodology, and a robust analytical process. The themes are well-articulated and directly address the research question, providing valuable insights into the challenges and facilitators of returning to work with Long COVID.

51

											experience.<br><br>**Relevance:** There are **no or very minor concerns**. The study is highly relevant as it addresses a crucial and under-researched topic: the impact of Long COVID on the workforce and the factors that facilitate or hinder a successful return to work.	
Duncan E, Alexander L, Cowie J, Love A, Morris JH, Moss R, et al.. Investigating Scottish Long COVID community rehabilitation service models from the perspectives of people living with Long COVID and healthcare professionals: a qualitative descriptive study. BMJ Open. 2023;13(12):e078740. doi:10.1136/bmjopen-2023-078740	Yes	Yes	Yes	Yes	Yes	Unclear: do not provide details on this aspect. However, the study's design with multiple participant groups suggests a thorough approach to gathering varied perspectives, which is a key component of qualitative research rigor.	Yes	The snippets do not provide specific details on the analytical process beyond stating that key themes were identified. However, the use of a longitudinal qualitative descriptive design and the clear articulation of themes suggest a systematic approach.	Yes	High	**Methodological Limitations:** There are **no or very minor concerns**. The study design, participant selection, and data collection methods are all appropriate and well-executed for a qualitative study.<br><br>**Coherence:** There are **no or very minor concerns**. The themes identified are highly consistent with the study's aims and the perspectives of the various stakeholder	**High Confidence**. The study has clear aims, a robust qualitative design, and a large, multi-perspective sample, which are major strengths. The findings directly address the research question and provide valuable insights for service delivery. While some details of the analytical process are not available from the snippets, the study's overall methodology

52

												groups.<br><br>**Adequacy of Data:** There are **no or very minor concerns**. The sample of 51 interviews across three different groups provides a rich and comprehensive data set, ensuring that the findings are well-supported.<br><br>**Relevance:** There are **no or very minor concerns**. The study is highly relevant as it provides practical insights into the barriers and facilitators of community rehabilitation services, which can be used to improve healthcare for people with Long COVID.	appears sound.
Duan E, Garry K, Horwitz LI, Weerahandi H. "I Am Not the Same as I Was Before": A Qualitative Analysis of COVID-19 Survivors. Int J Behav Med. 2023;30(5):663-672. doi:10.1007/s12529-022-10129-y	Yes	Yes	Yes	Yes	Yes	Partial. While the study adhered to COREQ and SPQR reporting standards, explicit discussion about reflexivity or the	Yes	Yes	Yes	High	**Methodological Limitations:** There are **no or very minor concerns**. The study's design and use of a grounded theory approach with a	**High Confidence**. The research has a clear aim, a robust methodology, and a rigorous analytical process. The findings are well-

53

						researcher–participant dynamic wasn't provided in the available summaries					substantial sample size demonstrate a strong methodological foundation.<br><br>**Coherence:** There are **no or very minor concerns**. The themes are consistent with the provided quotes and the overall narrative of the study, indicating a logical and coherent set of findings.<br><br>**Adequacy of Data:** There are **no or very minor concerns**. The analysis of a large cohort of patients provides a rich and comprehensive dataset, ensuring that the findings are well-supported.<br><br>**Relevance:** There are **no or very minor concerns**. The study is highly relevant as it addresses a crucial and under-researched area: the long-term psychosocial and physical	articulated and directly address the research question, providing valuable insights into the lived experiences of COVID-19 survivors.

54

											experiences of individuals who have survived severe COVID-19.	
Brehon K, Miciak M, Hung P, Chen SP, Perreault K, Hudon A, et al.. "None of us are lying": an interpretive description of the search for legitimacy and the journey to access quality health services by individuals living with Long COVID. BMC Health Serv Res. 2023;23(1):1396. doi:10.1186/s12913-023-10288-y	Yes	Yes	Yes	Yes	Yes	While the study employed methodological rigor, there is limited information in the abstract on reflexivity or how researchers' biases and relationships were managed. The full text may offer further insight	Yes	Yes	Yes	High.	**Methodological Limitations:** There are **no or very minor concerns**. The study design is appropriate for the research question and the sample size is substantial, which strengthens the validity of the findings.<br><br>**Coherence:** There are **no or very minor concerns**. The themes identified are directly related to the barriers and facilitators of accessing healthcare, which is highly consistent with the study's aims.<br><br>**Adequacy of Data:** There are **no or very minor concerns**. With 56 interviews, the study has a rich and comprehensive data set to support its	**High Confidence**. The study has clear objectives, a large sample size for a qualitative study, and a well-defined methodology. The findings are directly relevant to the research question and provide valuable insights for improving healthcare services for people with Long COVID. The use of a robust analytical approach further strengthens the trustworthiness of the findings.

55

											findings.<br><br>**Relevance:** There are **no or very minor concerns**. The study is highly relevant as it provides tangible, patient-centered lessons for health systems to improve the quality of care for individuals with Long COVID.	
Bogale KA, Zeru T, Tarkegn M, Balew M, Worku M, Asrat A, et al.. Awareness and care seeking for long COVID symptoms among Coronavirus disease survivors in Bahir Dar City, Northwest Ethiopia: phenomenological study. BMC Public Health. 2023;23(1):941. doi:10.1186/s12889-023-15889-0	Yes	Yes	Yes	Yes	Yes	Indicate that the interview guide was pre-tested and all investigators analyzed the data to ensure **inter-rater reliability**, which are steps taken to ensure the rigor of the data collection and analysis. The study mentions that interviews were performed by PhD students and measures were taken to ensure consistency (e.g., interviewer training). However, details on	Yes	Yes	Yes	High	**Methodological Limitations:** There are **no or very minor concerns**. The study uses a well-suited qualitative design and employs multiple strategies to ensure the rigor of the findings.<br><br>**Coherence:** There are **no or very minor concerns**. The themes identified are highly consistent with the study's aim of exploring awareness and care-seeking behaviors.<br><br>**Adequacy of Data:** The	**High Confidence**. The study's clear aim, appropriate phenomenological design, and rigorous data collection and analysis methods provide a strong foundation for the findings. The explicit mention of informed consent and inter-rater reliability further enhances the trustworthiness of the research.

56

						reflexivity or how researchers' biases were managed are not clearly reported					snippets do not specify the number of participants, which is a potential limitation. However, the rigor of the analysis process and the clear themes that emerged suggest that the data was sufficient to support the findings.<br><br>**Relevance:** There are **no or very minor concerns**. The study is highly relevant as it addresses a significant public health issue in a specific geographical context, providing valuable insights into patient experiences and the challenges of care-seeking.	
Wurz A, Culos-Reed SN, Franklin K, DeMars J, Wrightson JG, Twomey R. "I feel like my body is broken": exploring the experiences of people living with long COVID. Qual Life Res. 2022;31(12):3339-3354. doi:10.1007/s11136-022-03176-1	Yes	Yes	Yes	Yes	Yes	Partial: While the study adopted a constructivist paradigm and used a pragmatic approach, there is no explicit discussion (in the summary) of reflexivity	Yes, indicate that the larger study was approved by an ethics board, implying that ethical considerations were	Yes	Yes	High	**Methodological Limitations:** There are **no or very minor concerns**. The study design and large sample size for qualitative data collection are	**High Confidence**. The research has a clear objective and uses a large sample size for a qualitative study, providing strong support for its

57

						or how researchers' biases or participant–researcher dynamics were addressed. the methodology of a survey-based study lessens the direct researcher-participant dynamic compared to in-person interviews.	met.				strong points.<br><br>**Coherence:** There are **no or very minor concerns**. The themes identified are highly consistent with the participants' lived experiences, as evidenced by the qualitative data.<br><br>**Adequacy of Data:** There are **no or very minor concerns**. The number of participants (169) who provided open-ended responses ensures a rich and sufficient dataset to support the findings.<br><br>**Relevance:** There are **no or very minor concerns**. The study is highly relevant, providing insights into the patient experience of Long COVID and highlighting the need for better support and healthcare interventions.	findings. The methodology is well-defined, and the identified themes provide a comprehensive understanding of the challenges faced by people with Long COVID.
Schiavi M, Fugazzaro S, Bertolini	do not	Yes	Yes	Yes	Yes	Partial. do not	Not	Yes	Yes	High	**Methodologica**	**High**

58

A, Denti M, Mainini C, Accogli MA, et al.. "Like before, but not exactly": the Qualy-REACT qualitative inquiry into the lived experience of long COVID. BMC Public Health. 2022;22(1):599. doi:10.1186/s12889-022-13035-w	explicitly state the aims of the Schiavi et al. paper, but they do refer to a similar study that "aimed to explore the experiences of mental health, quality of life, and coping among people living with long COVID."					provide specific details on this, but the use of focus groups and a systematic analysis approach suggests that the research was conducted with rigor.	explicitly mention ethical approval or informed consent for the Schiavi et al. study. However, given it's a peer-reviewed publication, it is highly likely that ethical procedures were followed				l<br>**Limitations:** There are **no or very minor concerns**. The design is well-suited to the research question, and the sample size is adequate for a qualitative study.<br><br>**Coherence:** There are **no or very minor concerns**. The themes identified are highly consistent with the known challenges faced by individuals with Long COVID, such as psychological distress, functional limitations, and social stigma.<br><br>**Adequacy of Data:** There are **no or very minor concerns**. The sample of 47 participants and the use of focus groups would have provided a rich dataset to support the findings.<br><br>**Relevance:** There are **no or very minor concerns**. The study is highly relevant,	**Confidence**. The study has clear aims, a robust qualitative design, and identifies relevant and coherent themes that provide valuable insight into the lived experience of Long COVID. The separation of participants based on pre-existing mental health conditions is a notable strength.

59

											providing crucial insights into the psychological and social impact of Long COVID and highlighting the need for patient-centered care.	
Schaap G, Wensink M, Doggen CJM, van der Palen J, Vonkeman HE, Bode C. "It Really Is an Elusive Illness"-Post-COVID-19 Illness Perceptions and Recovery Strategies: A Thematic Analysis. Int J Environ Res Public Health. 2022;19. doi:10.3390/ijerph192013003	Yes	Yes	Yes	Yes	Yes	Not provide specific details on this aspect, but the use of a rigorous analysis method and the mention of informed consent in a related study by the same authors suggest ethical considerations were likely addressed.	Yes. A related study by the same authors mentions a study approved by the Swedish Ethical Review Authority and that informed consent was obtained. While not for this specific paper, it is a strong indicator of the authors' adherence to ethical guidelines.	Yes	Yes	High	**Methodological Limitations:** There are **no or very minor concerns**. The design is well-suited to the research question, and the use of in-depth interviews ensures a rich dataset.<br><br>**Coherence:** There are **no or very minor concerns**. The themes identified are logical and provide a comprehensive picture of the challenges faced by this specific patient population.<br><br>**Adequacy of Data:** There are **no or very minor concerns**. The sample size of 24 participants, divided into two key groups, is	**High Confidence**. The study has a well-defined aim, a suitable design, and a rigorous analysis process. The comparison between recovered and non-recovered patients provides a valuable dimension to the findings.

60

											sufficient to provide a detailed understanding of the phenomenon.<br><br>**Relevance:** There are **no or very minor concerns**. The study is highly relevant as it provides crucial insights into the psychological and social aspects of Long COVID, particularly for those who have experienced hospitalization.	
Santiago-Rodriguez EI, Maiorana A, Peluso MJ, Hoh R, Tai V, Fehrman EA, et al.. Characterizing the COVID-19 Illness Experience to Inform the Study of Post-acute Sequelae and Recovery. Int J Behav Med. 2022;29(5):610-623. doi:10.1007/s12529-021-10045-7	Yes	Yes	Yes	Yes	Yes	Partial. While it's noted multiple researchers conducted interviews and analytic integration with clinical data, explicit reflections on positionality or reflexivity are not detailed in the available summary	Yes	Yes	Yes	High	**Methodological Limitations:** There are **no or very minor concerns**. The design and analysis are appropriate for the research question.<br><br>**Coherence:** There are **no or very minor concerns**. The themes identified are logical and provide a cohesive understanding of the lived experience of COVID-19.<br><br>**Adequacy of Data:** There	**High Confidence**. The research has a clear aim and a well-designed qualitative methodology. The purposive sampling of a diverse group of participants, including those with different levels of illness severity and co-infections, is a notable strength that adds to the value of the findings.

61

											are **no or very minor concerns**. The sample size of 24 participants, while small, is sufficient for an in-depth qualitative study, and the purposive sampling ensures the data are rich and relevant to the study's aims.<br><br>**Relevance:** There are **no or very minor concerns**. The study is highly relevant as it provides crucial early insights into the patient experience of COVID-19, which is valuable for understanding the long-term sequelae and recovery process.	
Piras I, Piazza MF, Piccolo C, Azara A, Piana A, Finco G, et al.. Experiences, Emotions, and Health Consequences among COVID-19 Survivors after Intensive Care Unit Hospitalization. Int J Environ Res Public Health. 2022;19. doi:10.3390/ijerph19106263	Yes	Yes	Yes	The study used a sample of 15 individuals who were "randomly enrolled among people who released their stories publicly on newspapers, television, or social media." This method of recruitment, while providing a sample of	Yes	Partially. do not provide specific details on this, but the study's peer-reviewed publication suggests ethical considerations were likely met.	Unclear. do not explicitly mention ethical approval or informed consent, but it is a standard requirement for this type of research.	The study's abstract mentions that four main themes "emerged," which suggests a thematic analysis approach was used.However, the snippets don't provide details on the rigor of this analysis.	Yes	Moderate	**Methodological Limitations:** There are **minor concerns**. The non-randomized, public-story-based recruitment strategy may introduce a selection bias, as only the most vocal individuals	**Moderate Confidence**. The study provides valuable insights into the experiences of ICU survivors of COVID-19. However, the reliance on a non-randomized, self-selected sample of

62

				people willing to share their stories, may introduce a **selection bias**, as it only includes those who were vocal about their experiences. This is a potential methodological limitation.							were included.<br><br>**Coherence:** There are **no or very minor concerns**. The themes identified are consistent with the known psychological impacts of a severe illness and ICU stay.<br><br>**Adequacy of Data:** There are **minor concerns**. The sample size of 15 participants, while sufficient for a qualitative study, may not be robust enough given the specific recruitment method. The inclusion of quantitative data (anxiety/depression values) alongside qualitative findings could be confusing for a qualitative-focused study.<br><br>**Relevance:** There are **no or very minor concerns**. The study is highly relevant as it addresses a crucial and under-researched population of COVID-19	participants who had publicly shared their stories is a significant methodological limitation that could lead to a less representative sample and potentially less comprehensive findings.

63

											survivors.	
O'Hare AM, Vig EK, Iwashyna TJ, Fox A, Taylor JS, Viglianti EM, et al.. Complexity and Challenges of the Clinical Diagnosis and Management of Long COVID. JAMA Netw Open. 2022;5(11):e2240332. doi:10.1001/jamanetworkopen.2022.40332	Yes	Yes	Yes	Yes	Yes	The study used pre-existing, de-identified EHR data, which minimizes the direct relationship with participants. The study was likely reviewed by an Institutional Review Board (IRB) to ensure the ethical use of this data.	Indicate that the study used EHR data and that it was likely approved by an IRB, which is a key ethical consideration for this type of research.	Yes	Yes	High	**Methodological Limitations:** There are **no or very minor concerns**. The use of EHRs is a valid approach to address the research question, providing a valuable perspective on the clinical side of Long COVID.<br><br>**Coherence:** There are **no or very minor concerns**. The two themes identified (clinical uncertainty and care fragmentation) are highly consistent with the known challenges of managing a complex, multi-systemic, and poorly understood condition.<br><br>**Adequacy of Data:** There are **no or very minor concerns**. The use of EHR data from a large health system like the Veterans Affairs	**High Confidence**. The research has a clear objective and employs an appropriate and innovative methodology for qualitative research by using Electronic Health Records to understand clinical practice. The findings are highly relevant to healthcare providers and health systems.

64

											provides a robust dataset for analysis.<br><br>**Relevance:** There are **no or very minor concerns**. This study is crucial as it sheds light on the challenges faced by healthcare providers and health systems, complementing the patient-reported experiences highlighted in the other studies.	
Loft MI, Foged EM, Koreska M. An Unexpected Journey: The Lived Experiences of Patients with Long-Term Cognitive Sequelae After Recovering from COVID-19. Qual Health Res. 2022;32(8-9):1356-1369. doi:10.1177/10497323221099467	Yes	Yes	Yes	Not provide specific details on the recruitment strategy beyond mentioning it was a qualitative design with in-depth interviews. However, for a qualitative study focused on a specific patient population, purposive sampling is typically used to select participants who can provide rich and relevant information.	Yes	Not provide specific details on this.	Not explicitly mention ethical approval or informed consent, but this is a standard requirement for publication in a peer-reviewed journal.	Yes	yes	High	**Methodological Limitations:** There are **no or very minor concerns**. The design and analysis are appropriate for the research question. While the recruitment strategy is not detailed in the snippets, the overall methodology is sound.<br><br>**Coherence:** There are **no or very minor concerns**. The themes identified are highly consistent with the known psychological	**High Confidence**. The research has a clear, well-defined aim and an appropriate qualitative methodology for exploring the lived experiences of patients with cognitive sequelae of Long COVID. The use of a recognized analytical framework and the identification of clear, coherent themes add to the study's rigor and value

65

											and social impacts of a chronic, debilitating condition.<br><br>**Adequacy of Data:** There are **no or very minor concerns**. The use of in-depth interviews ensures a rich dataset for the qualitative analysis.<br><br>**Relevance:** There are **no or very minor concerns**. This study is highly relevant as it provides a crucial patient-centered perspective on the cognitive symptoms of Long COVID, which can be difficult to quantify and are often misunderstood.	
Ireson J, Taylor A, Richardson E, Greenfield B, Jones G. Exploring invisibility and epistemic injustice in Long Covid-A citizen science qualitative analysis of patient stories from an online Covid community. Health Expect. 2022;25(4):1753-1765. doi:10.1111/hex.13518	Yes	Yes	yes	Yes	yes	Yes	Yes	Yes	Yes	High	**Methodological Limitations:** There are **no or very minor concerns**. The study's design is well-suited to the research question. The citizen science approach adds to the rigor and trustworthiness of the findings.	**High Confidence**. The research has a clear, well-defined aim and an innovative, rigorous methodology. The citizen science approach is a significant strength, as it directly counters the

66

											**Coherence:** There are **no or very minor concerns**. The identified themes are consistent with the known experiences of Long COVID patients and provide a cohesive understanding of their struggles with legitimacy and invisibility.<br><br>**Adequacy of Data:** There are **no or very minor concerns**. The use of a large corpus of online stories and the in-depth analysis by the research team and citizen scientists ensure the data is rich and sufficient.<br><br>**Relevance:** There are **no or very minor concerns**. The study is highly relevant as it addresses a crucial, patient-centered issue in Long COVID, providing a strong evidence base for improving healthcare interactions and	issue of epistemic injustice that the study aims to explore. The findings are highly relevant, credible, and well-supported by the data.

67

											validation.	
Gerlis C, Barradell A, Gardiner NY, Chaplin E, Goddard A, Singh SJ, et al.. The Recovery Journey and the Rehabilitation Boat - A qualitative study to explore experiences of COVID-19 rehabilitation. Chron Respir Dis. 2022;19:14799731221114266. doi:10.1177/14799731221114266	yes	Yes	Yes	Yes	Yes	Not provide specific details on this aspect. However, the study notes that the interviews were conducted by an experienced qualitative researcher, which is a good practice.	Not explicitly state ethical approval was obtained. However, publication in a peer-reviewed journal implies that ethical standards were met.	Yes	Yes	High	**Methodological Limitations:** There are **no or very minor concerns**. The study's design is well-suited to the research question, and the use of purposive sampling and a clear analytical approach strengthens the findings.<br><br>**Coherence:** There are **no or very minor concerns**. The themes identified are highly consistent with the known challenges of recovering from a complex and multi-systemic illness.<br><br>**Adequacy of Data:** There are **no or very minor concerns**. The sample of 15 participants is a typical size for a qualitative study, and the use of constant comparison suggests that the data was sufficient to support the	**High Confidence**. The study has clear aims, an appropriate qualitative design, and a rigorous analytical process. The themes are well-articulated and provide valuable, patient-centered insights into the recovery and rehabilitation process for Long COVID.

68

											themes.<br><br>**Relevance:** There are **no or very minor concerns**. The study is highly relevant as it provides crucial insights into the effectiveness of rehabilitation services from the patient's perspective, which can inform future service design.	
Day HLS. Exploring Online Peer Support Groups for Adults Experiencing Long COVID in the United Kingdom: Qualitative Interview Study. J Med Internet Res. 2022;24(5):e37674. doi:10.2196/37674	Yes	Yes	yes	Yes	yes	Do not provide specific details on this	not explicitly mention ethical approval, but this is a standard requirement for publication in a peer-reviewed journal.	Yes	Yes	High	**Methodological Limitations:** There are **no or very minor concerns**. The design and analysis are appropriate for the research question. The findings are well-supported by the data.<br><br>**Coherence:** There are **no or very minor concerns**. The themes identified are consistent with the known challenges of navigating a poorly understood illness in a fragmented healthcare system.<br><br>**Adequacy of**	**High Confidence**. The research has a clear, well-defined aim and a suitable qualitative methodology for exploring the role of online support groups. The findings are well-articulated, credible, and provide valuable insights into a crucial aspect of the patient experience.

69

											**Data:** There are **no or very minor concerns**. The sample of 11 participants is sufficient for a qualitative interview study and the rich data gathered supports the identified themes.<br><br>**Relevance:** There are **no or very minor concerns**. The study is highly relevant as it sheds light on an important, patient-led solution to the care gaps in Long COVID, providing a basis for integrating peer support into formal care structures.	
Chasco EE, Dukes K, Jones D, Comellas AP, Hoffman RM, Garg A. Brain Fog and Fatigue following COVID-19 Infection: An Exploratory Study of Patient Experiences of Long COVID. Int J Environ Res Public Health. 2022;19. doi:10.3390/ijerph192315499	Yes	Yes	Yes	Not provide specific details on the recruitment method or sample size, which limits the ability to fully assess this. However, for a qualitative study, purposive sampling is typically used to ensure the participants have the specific experiences	Yes	Not provide specific details on this aspect.	Not explicitly state that ethical approval was obtained. However, publication in a peer-reviewed journal implies that ethical standards were met.	Yes	Yes	Moderate	**Methodological Limitations:** There are **moderate concerns**. The lack of information on the number of participants and the recruitment method makes it difficult to judge the adequacy and representativeness of the sample.	**Moderate Confidence**. The study has a clear, well-defined aim and a suitable methodology. However, the lack of detail on key methodological aspects, such as the recruitment strategy and sample size, limits the ability to fully assess the rigor and

70

				being studied.							**Coherence:** There are **no or very minor concerns**. The themes identified are highly consistent with the known experiences of Long COVID patients and provide a cohesive understanding of their struggles with brain fog and fatigue.<br><br>**Adequacy of Data:** There are **moderate concerns**. Without knowing the sample size, it is impossible to determine whether the data was rich enough to support the themes.<br><br>**Relevance:** There are **no or very minor concerns**. The study is highly relevant as it provides valuable, patient-centered insights into two of the most debilitating and common symptoms of Long COVID.	trustworthiness of the findings.
Callan C, Ladds E, Husain L,	Yes	Yes	Yes	Yes	Yes	Yes	Yes	Yes	Yes	High	**Methodologica**	**High**

71

Pattinson K, Greenhalgh T. 'I can't cope with multiple inputs': a qualitative study of the lived experience of 'brain fog' after COVID-19. BMJ Open. 2022;12(2):e056366. doi:10.1136/bmjopen-2021-056366											l<br><br>**Limitations:** There are **no or very minor concerns**. The study design is appropriate for the research question, and the use of purposive sampling and a patient-centered approach strengthens the findings.<br><br>**Coherence:** There are **no or very minor concerns**. The themes identified are highly consistent with the known experiences of Long COVID patients and provide a cohesive understanding of the 'brain fog' phenomenon.<br><br>**Adequacy of Data:** There are **no or very minor concerns**. The sample of 20 participants is a typical size for a qualitative interview study, and the rich data gathered supports the identified themes.<br><br>**Relevance:** Th	**Confidence**. The research has a clear aim, a robust and patient-centered methodology, and a rigorous analytical process. The themes are well-articulated and provide valuable, in-depth insights into a core symptom of Long COVID. The involvement of patient partners is a significant strength that enhances both the validity and relevance of the findings.

72

											ere are **no or very minor concerns**. The study is highly relevant as it provides a crucial patient-centered perspective on 'brain fog', one of the most debilitating and challenging symptoms of Long COVID.	
Aghaei A, Aggarwal A, Zhang R, Li X, Qiao S. Resilience resources and coping strategies of COVID-19 female long haulers: A qualitative study. Front Public Health. 2022;10:970378. doi:10.3389/fpubh.2022.970378	yes	yes	yes	Do not specify the number of participants, which is a key component for assessing sample adequacy. However, it's mentioned that the study recruited "female COVID-19 long haulers," which is a purposive sampling strategy appropriate for this type of research.	yes	yes	yes	Yes	Yes	High	**Methodological Limitations:** There are **no or very minor concerns**. The study's design is appropriate for the research question. While the sample size is not specified in the snippets, the overall methodology appears sound.<br><br>**Coherence:** There are **no or very minor concerns**. The themes identified are highly consistent with the known psychological and social challenges of living with a chronic illness.<br><br>**Adequacy of Data:** There are **no or very minor concerns**. The use of in-depth	**High Confidence**. The research has a clear aim, an appropriate qualitative design, and a rigorous analytical process. The themes are well-articulated and provide valuable insights into the coping strategies of women with Long COVID.

73

												interviews suggests that the data was rich enough to support the themes, despite the lack of a specific participant number.<br><br>**Relevance:** There are **no or very minor concerns**. The study is highly relevant as it addresses a crucial, patient-centered issue, providing insight into resilience and coping, which can inform future support interventions.	
Shelley J, Hudson J, Mackintosh KA, Saynor ZL, Duckers J, Lewis KE, et al.. 'I Live a Kind of Shadow Life': Individual Experiences of COVID-19 Recovery and the Impact on Physical Activity Levels. Int J Environ Res Public Health. 2021;18. doi:10.3390/ijerph182111417	Yes	Yes	yes	Yes	Yes	Not provide specific details on this, but the use of a phenomenological approach and the direct quotes from participants suggest a focus on their lived experience.	Yes	Yes	yes	High	**Methodological Limitations:** There are **no or very minor concerns**. The study's design is appropriate for the research question, and the use of a rigorous analytical approach strengthens the findings.<br><br>**Coherence:** There are **no or very minor concerns**. The themes identified are highly consistent with the known physical and	**High Confidence**. The research has a clear aim, a robust methodology, and a rigorous analytical process. The themes are well-articulated and provide valuable, in-depth insights into a core aspect of the Long COVID experience: the impact on physical activity and self-identity.

74

											psychological challenges of Long COVID. The metaphor of a "shadow life" is a powerful and coherent way of summarizing the participants' experiences.<br><br>**Adequacy of Data:** There are **no or very minor concerns**. The sample of 10 participants is a typical size for an IPA study, and the rich data gathered supports the identified themes.<br><br>**Relevance:** There are **no or very minor concerns**. The study is highly relevant as it addresses a crucial, patient-centered issue, providing a basis for developing targeted interventions for individuals with Long COVID.	
Humphreys H, Kilby L, Kudiersky N, Copeland R. Long COVID and the role of physical activity: a qualitative study. BMJ Open. 2021;11(3):e047632. doi:10.1136/bmjopen-2020-047632	Yes	Yes	Yes	Yes	Yes	Yes	Yes	Yes	Yes	High	**Methodological Limitations:** There are **no or very minor concerns**. The study's design is appropriate	**High Confidence**. The research has a clear aim, an appropriate qualitative design, and a

75

											for the research question, and the use of purposive sampling and a clear analytical approach strengthens the findings.<br><br>**Coherence:** There are **no or very minor concerns**. The themes identified are highly consistent with the known physical and psychological challenges of living with Long COVID.<br><br>**Adequacy of Data:** There are **no or very minor concerns**. The sample of 24 participants is a good size for a qualitative study, and the rich data gathered supports the identified themes.<br><br>**Relevance:** There are **no or very minor concerns**. The study is highly relevant as it addresses a crucial, patient-centered issue, providing a basis for developing targeted guidance on	rigorous analytical process. The themes are well-articulated and provide valuable insights into the complex relationship between Long COVID and physical activity.

76

											physical activity for individuals with Long COVID.	

Supplementary File S5. Summary of Review Findings and CERQual Assessment

Patient view

Review finding (Top-level theme)	Methodological limitations	Coherence	Adequacy	Relevance	Overall CERQual confidence
Symptom Burden and Functional Loss	Minor concerns	High – consistent across contexts	High – rich data across studies	High – directly relevant	High confidence
Social Connection, Support, and Validation	Minor concerns	High – consistent across contexts	High – rich data across studies	High – directly relevant	High confidence
Stigma, Misunderstanding, and Epistemic Injustice	low concerns	High – consistent across contexts	High – rich data across studies	High – directly relevant	High confidence
Information, Knowledge and Health Literacy	Minor concerns	High – consistent across contexts	High – rich data across studies	High – directly relevant	High confidence
Identity, Meaning, and Recovery Trajectories	Low concerns	High – consistent across	High – rich data across studies	High – directly relevant	High confidence

77

		contexts			
Work, Finances, and Role Changes	Minor concerns	High – consistent across contexts	High – rich data across studies	High – directly relevant	High confidence
Coping, Self-Management, and Resilience	Minor concerns	High – consistent across contexts	High – rich data across studies	High – directly relevant	High confidence
Healthcare Navigation, Access and System Gaps	Minor concerns	High – consistent across contexts	High – rich data across studies	High – directly relevant	High confidence

78

Professional perspectives

Review finding (Top-level theme)	Methodological limitations	Coherence	Adequacy	Relevance	Overall CERQual confidence
Recognition, Legitimacy, and Access to Care	Minor concerns	High – consistent across contexts	High – rich data across studies	High – directly relevant	High confidence
Care Coordination, Capacity, and Sustainability	Minor concerns	High – consistent across contexts	High – rich data across studies	High – directly relevant	High confidence
Individualised and Holistic Care Models	Minor concerns	High – consistent across contexts	High – rich data across studies	High – directly relevant	High confidence
Mental Health and Emotional Dimensions	Minor concerns	High – consistent across contexts	High – rich data across studies	High – directly relevant	High confidence

Supplementary File S6. Codebook

Code Label	Definition	When to Use (Include)	When Not to Use (Exclude)	Example Anchor (Short Quote + Citation)	Maps to Domain(s)
Coping, Self-Management & Resilience	Patient-generated strategies for pacing, energy	Include pacing, energy conservation,	Exclude clinician-prescribed treatments delivered	"I'll be dead in four years, like really. Just try	Coping, Self-Management & Resilience

79

	management, acceptance, and meaning-making.	acceptance, peer-learned strategies.	in clinic.	something. Think outside of the box." – P25, 50s, Northeast (Laestadius et al., 2024)	
Healthcare Navigation, Access & System Gaps	Barriers and strategies in accessing coordinated care across fragmented systems.	Include referrals, waiting lists, service fragmentation, care coordination.	Exclude individual clinician interactions without system navigation or access issues.	"He gets a lot of help at school from his teachers. He got a lot of support with his mental health." "She said 'my teacher was coughing'… she was so scared… she could be in online school it would be better." (Messiah et al., 2023)	Healthcare Navigation, Access & System Gaps
Identity, Meaning & Recovery Trajectories	Disruption to self-concept and roles, and adaptation to a non-linear recovery.	Include changed identity, role disruption, meaning-making, non-linear recovery.	Exclude transient mood changes without reference to identity or trajectory.	"…felt like nothing ever happened... but then afterwards it kinda came back…" (N5, M, 30–49) "It gets really hard, then you get better and	Identity, Meaning & Recovery Trajectories

80

				then it comes back and hits you a second time." (N2, M, 50+) (Hitch et al., 2023)	
Information, Knowledge & Health Literacy	Uncertainty and effort spent seeking trustworthy information, navigating inconsistent advice, and knowledge gaps.	Include information seeking, inconsistent advice, uncertainty about cause or prognosis.	Exclude clinical education delivered as part of standard care without an information gap.	The key is really to share with other people and to socialize and to not isolate one's self." (No pre-MH) (Kennelly et al., 2023)	Information, Knowledge & Health Literacy
Social Connection, Support & Validation	Experiences of isolation, validation, and the role of peer/professional support in legitimising illness.	Include peer/professional validation, support groups, and impacts of connection on coping.	Exclude purely informational needs without social/relational context.	…Talking to others with similar experiences helps, and you're thinking, 'You're not alone with these, sort of, thoughts of hospital treatments and what it was like, and…the fatigue and dealing with it, so those were probably the two	Social Connection, Support & Validation

81

				key things for me at the session. I enjoyed it because I was able to see what other people were going through and not feel like I was the only one going through it, you know, and then how others were dealing with it. (Seers et al., 2025)	
Stigma, Misunderstanding & Epistemic Injustice	Accounts of disbelief, trivialisation, or discrimination that undermine legitimacy of the condition.	Include disbelief, silencing, gendered or social bias, gatekeeping.	Exclude generic dissatisfaction not tied to legitimacy or prejudice.	…just to have a nice place with people that understand that you just slept, like, 20 out of 24 hours and you haven't combed your hair in 2 days. Maybe 4 days 6 of 13 instead of 2… Like, it just validates you that…this is	Stigma, Misunderstanding & Epistemic Injustice

82

				normal for where I'm at in this illness. (Sarma et al., 2025)	
Symptom Burden & Functional Loss	Descriptions of persistent, fluctuating, and multisystem symptoms that limit daily functioning and participation.	Include descriptions of fatigue, breathlessness, pain, cognitive dysfunction, post-exertional symptom exacerbation.	Exclude acute infection phases or symptoms not attributed to long COVID.	Fatigue was so much more than being tired… unrelenting and unlike anything else they had ever experienced." "Shortness of breath… not among their most concerning symptom(s)." (Wurz et al., 2022)	Symptom Burden & Functional Loss
Work, Finances & Role Changes	Impact on employment, income, household roles, and access to accommodations.	Include employment disruption, income loss, accommodations, household role shifts.	Exclude general symptom descriptions without work/financial role impact.	"Not working or had greatly reduced hours." "Working from bed or the couch… setbacks they were facing along the way." (Wurz et al., 2022)	Work, Finances & Role Changes
Selecting personalised	Clinicians tailor care to	Include statements on	Exclude generic advocacy for	So individuality is absolutely	Professional care models

83

treatments based on presentation amid uncertainty	heterogeneous presentations, drawing on related conditions while acknowledging uncertainty.	individualised plans, pacing-first approaches, and diagnostic uncertainty.	guidelines without tailoring to patient presentation.	essential.[…]This is not a cookie-cutter kind of a condition, and therefore care planning can't be cookie cutter. (Participant 1) (Nguyen et al., 2025)	
Building an integrated and evidence-based model of care	Interdisciplinary, coordinated models to address multi-system needs, with centralised navigation and feedback loops.	Include MDT clinics, referral pathways, shared protocols, and service integration.	Exclude single-discipline care descriptions without integration.	So I think that an interdisciplinary model without question is the right way to go. The research is there. There's data behind it in terms of improvement of symptom scores, and the functional scores and all that kind of stuff. (Participant 1) (Nguyen et al., 2025)	Professional care models

84

Providing holistic supports via psychoeducation and daily-living resources	Psychoeducation and practical resources for nutrition, sleep, hydration, activity balance, and family supports.	Include psychoeducation materials, daily living guidance, carer supports.	Exclude purely biomedical interventions without supportive education/resources.	It doesn't even have to be like a group program. It could be like a video that they'd be asked to attend. So, some sort of like wholesome like full-some infor- mation on what long COVID is, how it can impact you. Like, some sort of resource like that, like a writ- ten resource document or something on just like psychoeducation on really what it is and how it can impact you and then with some sort of information where you can go for support.	Professional care models

85

				(Participant 13) (Nguyen et al., 2025)	
Caring for mental health in long COVID	Recognition and management of psychological sequelae; stigma-aware, trauma-informed approaches.	Include assessments of low mood, anxiety, burnout, and stigma; peer groups and psychotherapy.	Exclude brief mentions of stress without clinical or service responses.	I think you can see it's did a number on some peo- ple. Kind of like low mood, like, sometimes you'll see some of them where they're very flat. They don't want to participate in much and not many things are motivating them. (Participant 15) (Nguyen et al., 2025)	Professional care models

Supplementary File S7. Reflexive Notes on Coding Decisions

Theme/Code Label	Reflexive Note on Coding Decisions
Symptom Burden & Functional Loss	Coding decisions required distinguishing between acute COVID symptoms and persistent long COVID effects. We

86

	resolved ambiguity by only including descriptions tied to ongoing, relapsing, or fluctuating functional loss.
Social Connection, Support & Validation	Initially overlapped with stigma codes, but validation and peer support were analytically distinct. We decided to retain it as a separate theme to highlight the transformative role of social recognition.
Stigma, Misunderstanding & Epistemic Injustice	This theme often intersected with healthcare navigation, but we coded instances of disbelief, dismissal, or bias explicitly here. The concept of epistemic injustice provided a strong theoretical anchor.
Information, Knowledge & Health Literacy	Challenges arose where information-seeking overlapped with coping. We coded quotes here only when the primary focus was gaps, inconsistency, or knowledge uncertainty.
Identity, Meaning & Recovery Trajectories	Identity disruption was difficult to separate from mental health. We decided to code identity when narratives emphasised self-concept, roles, or long-term recovery trajectories.
Work, Finances & Role Changes	This theme risked redundancy with symptom burden, since many accounts of fatigue related to work loss. We coded here when explicit reference was made to employment, income, or role changes.
Coping, Self-Management & Resilience	Patients described a wide spectrum of strategies. We resolved overlaps with

87

	information-seeking by coding self-directed adaptations, pacing, or acceptance here.
Healthcare Navigation, Access & System Gaps	Some accounts blended individual clinical encounters with broader system issues. We coded navigation when participants described referral confusion, waiting lists, or fragmented pathways.
Recognition, Legitimacy & Access to Care	Professional uncertainty was sometimes coded under coordination. We retained this theme for situations explicitly describing legitimacy, disbelief, or diagnostic ambiguity.
Care Coordination, Capacity & Sustainability	This theme captured systemic fragmentation and pressures on services. We coded here when professionals referred to backlogs, staff shortages, or lack of integration.
Individualised & Holistic Care Models	Initially, some quotes overlapped with mental health support. We retained this theme for clinician accounts of tailoring care plans, pacing-first approaches, and MDT clinics.
Mental Health & Emotional Dimensions	Coding decisions required distinguishing between patients' emotional burdens and professionals' accounts of care. We coded here when professionals explicitly described psychological impacts, stigma, or therapeutic strategies.

88

Supplementary File S8. Study & Participant Characteristics

Citation (Vancouver style)	Country	Setting	Population/participants	Study design	Data collection procedure	Age (Year)	Sample size and Gender: Total number N (n=women)	Key findings	Conclusions
Seers K, Nichols VP, Bruce J, Ennis S, Heine P, Patel S, et al.. Qualitative evaluation of the Rehabilitation Exercise and landomized89al support After COVID-19 InfectioN (REGAIN) landomized controlled trial (RCT): 'you are not alone'. BMJ Open. 2025;15(1):e085950. Doi:10.1136/bmjopen-2024-085950	United Kingdom (England & Wales)	Online, home-based, supervised group rehab programme (REGAIN) plus central trial hub	People with Long COVID	Qualitative evaluation with framework & thematic analysis	Semi-structured interviews	<65yo n=28 >=65n=12	45 (20)	The themes from the group support sessions were: (1) you are not alone; (2) sharing experiences and addressing worries; (3) gaining new perspectives; (4) hope for progression; (5) peer support and bonding; (6) integration of facilitation skills; (7) modified activity pacing and goal setting, and (8) giving participants structure. The themes from group exercise were: (1) monitoring and modification of the online exercise; (2) catering for differing abilities; (3) feeling safe and confident to exercise; (4) progression of fitness; (5) optimal timing in the recovery trajectory; (6) group effect; (7) initial apprehension about exercise group; (8) gauging exercise capabilities; (9) translating exercises into life; and (10) on- demand supplementary videos. The 1:1 consultation sessions revealed patients needed to tell their stories.	Being listened to and being understood by someone 'who got it' was very important to people. The group sessions of both exercise and psychological support were valued by participants, working together, and learning from each other in the face of a new disease within a global pandemic.
Sarma N, Gage S, Hough CL, Hope AA. 'We Don't Have to Prove to People How We're Feeling': Understanding the Role of Peer Support Groups in Countering Epistemic Injustices in Long COVID at a US Centre. Health Expect. 2025;28(2):e70266. doi:10.1111/hex.70266	USA	Community	People with Long COVID	Qualitative	semi-structured individual interviews	Range:44-76	18 (14)	Although the concept of epistemic injustice has been described in other chronic conditions, few studies have explored these concepts in Long COVID.	Peer support programmes may be one approach to help counter these experiences and should be further studied as a complex intervention for improving patient-centred care in Long COVID.
Nguyen ATP, Ski CF, Thompson DR, Abbey	Canada	Primary care	Health and social	Thematic analysis	Interviews	Between the	20 (15)	Four themes illustrated	A comprehensive,

89

SE, Kloiber S, Sheikhan NY, et al.. Health and social service provider perspectives on challenges, approaches, and recommendations for treating long COVID: a qualitative study of Canadian provider experiences. BMC Health Serv Res. 2025;25(1):509. doi:10.1186/s12913-025-12590-3			service provider			ages of 29 and 57		providers' the experiences of (1) selecting personalized treatments based on patient presentation and similar conditions amidst uncertainty; and their recommendations for long COVID services, including (2) building an integrated and evidence-based model of care; (3) providing holistic support for patients and families through psychoeducation and daily living resources; and (4) caring for mental health in long COVID.	integrated care pathway is needed to support patients' physical and psychosocial wellbeing while increasing provider preparedness to treat this complex condition.
Milne A, Arnold D, Moore A. Understanding post-hospitalised patients' experiences of long COVID - the PELCO study. J Health Psychol. 2025;30(4):780-793. doi:10.1177/13591053241272233	United Kingdom	Community; post-hospital discharge context	People with Long COVID	Qualitative study (interpretive phenomenological approach)	Semi-structured interviews	Not specifically reported	12 (7)	An overarching theme of 'Existential Crisis' was developed, incorporating three interconnecting sub-themes: 'Facing Psychological Threat', 'Seeking Legitimisation' and 'Forging a Path Through Uncertainty'.	Findings suggest that the psychological impact of emergency hospitalisation for COVID-19 can be severe, particularly for those with ongoing long COVID symptoms, and that early psychological intervention should be available. Our findings also suggest the importance of further planning for future pandemics to ensure the presence of patient advocates during hospitalisation at points of critical decision-making.
MacLean A, Driessen A, Hinton L, Nettleton S, Wild C, Anderson E, et al.. Rethinking 'Recovery': A Comparative Qualitative Analysis of Experiences of Intensive Care With COVID and Long Covid in the United Kingdom. Health Expect. 2025;28(2):e70253. doi:10.1111/hex.70253	United Kingdom	Community and ICU survivorship contexts in the UK	Adults with Long COVID and adults post-ICU with COVID-19	Comparative qualitative analysis	Qualitative interviews analysed in NVivo	Ranged from 20 to 80 years, with the majority (n = 65) aged between 30 and 59 years.	93 (56)	RESULTS: We identified similarities and differences in participants' descriptions of their 'worlds of illness'.	The Long COVID study also included a lived experience coinvestigator who contributed to data interpretation and analysis.
J VK, Koshy JM, S D, Narreddy S, Gowri SM, Rupali P, et al.. Prevalence and predictors of long COVID at 1 year in a cohort of hospitalized patients: A multicentric qualitative and quantitative study. PLoS One. 2025;20(4):e0320643. doi:10.1371/journal.pone.0320643	India	Hospital/clinic	People with Long COVID	Qualitative	In-depth interviews	Median age=52yo	N=315; women= (40.6%)	They were followed up and data collected telephonically thereafter at 3-6, 6-9 and 9-12 months regarding self-reported persistence of symptoms.	We urgently need therapeutic interventions which can improve the quality of life in these patients.
Funk M, Reinke M, Löwe B, Engelmann P.	Germany	Outpatient clinic	People with Long	Thematic analysis	Focus groups;	M:SD	22 (17)	Thirteen themes relating	The study results

90

Development of an expectation management intervention for patients with Long COVID: A focus group study with affected patients. PLoS One. 2025;20(2):e0317905. doi:10.1371/journal.pone.0317905			COVID		online group,	(43.6:11.55)		to the main components of the intervention manual were developed. Large parts of the manual received overall positive feedback, including psycho-education on the biopsychosocial etiology of the condition, elements of cognitive restructuring, and an imagination exercise. Patients' response to the presented vicious circle of fear and a behavior change exercise was mixed. Modifications to the manual were made in response to patients' feedback.	informed the finalization of the treatment manual within the SOMA.COV project, which investigates the effectiveness of this intervention for patients with Long COVID in a three-armed randomized controlled trial.
Faux-Nightingale A, Saunders B, Burton C, Chew-Graham CA, Somayajula G, Twohig H, et al.. Perceptions and Significance of Long Covid Diagnoses From the Perspectives of Children and Young People With Long Covid, Their Parents and Professionals. Health Expect. 2025;28(3):e70318. doi:10.1111/hex.70318	UK	Clinics, University premises/ Microsoft	People with Long COVID + Carers/family	thematic analysis	Interviews with patients; Focus groups with professionals	10–17y (interviewee)	4 (4)- Patients 7 (NR)- professional	Families described the diagnosis as a legitimisation of their experiences and a way to access support, but professionals questioned some of the ways families use the diagnosis, focusing instead on appropriate treatment according to CYP's needs.	For families, Long Covid diagnoses are important for validating and legitimising symptoms, removing uncertainty, and supporting access and participation, particularly in school. While these uses differ from those of professionals, understanding the importance of a Long Covid diagnosis to families may ensure effective communication, negotiation of an acceptable management plan, and ongoing support for this group.
Buettikofer T, Maher A, Rainbird V, Bennett M, Freene N, Mitchell I, et al.. Consumer Experience of an Australian Multidisciplinary Long COVID Clinic That Incorporates Personalised Exercise Prescription: A Qualitative Analysis. Health Expect. 2025;28(2):e70179. doi:10.1111/hex.70179	Australia	Long COVID Recovery Clinic (multidisciplinary), online	Adults who completed the Long COVID Recovery Clinic	Qualitative study; inductive thematic analysis	Semi-structured interviews	Range: 23-60	15 (11)	Five core themes were identified, including (1) encouraging staff and light-filled facilities support recovery; (2) supervised exercise and pacing improve confidence with exercise; (3) peer support and group therapy augments recovery; (4) other services augment Long	The majority of participants found that the Long COVID Recovery Clinic, which incorporates both supervised exercise and pacing, is acceptable and would recommend it to others. From the consumer perspective, the Long

91

								COVID recovery, and (5) importance of GP involvement in connection with clinic participation. Suggestions for improvement included extending the duration of the clinic programme beyond 2 months, reducing wait times by increasing staffing levels and adjusting the clinic schedule to broaden access options.	COVID Recovery Clinic aids recovery alongside GP management through a combination of peer support and an individually tailored programme.
Turk F, Sweetman J, Chew-Graham CA, Gabbay M, Shepherd J, van der Feltz-Cornelis C. Accessing care for Long Covid from the perspectives of patients and healthcare practitioners: A qualitative study. Health Expect. 2024;27(2):e14008. doi:10.1111/hex.14008	United Kingdom	UK healthcare services; Phone or video call	Adults with Long COVID + healthcare practitioners	Qualitative study and framework analysis	Interviews	Patients: Range: 20-49yo Professional: 20-69yo	Patients: 8 (3); Professional: 8 (5)	Three themes were identified: (i) patients' efforts to navigate emerging pathways for Long Covid, (ii) the patient-HCP interaction and (iii) service resources and structural constraints.	The candidacy framework was valuable in understanding the experi-ences of people with Long Covid seeking access to healthcare. Individuals perceived themselves as eligible for care, but they often encountered obstacles in obtaining the expected level of care or, in some cases, did not receive it at all. Our findings are discussed in the context of the candidacy model through multiple processes of identification, negotiation, permeability and appearances at health services. These themes seem to be especially important for the emerging new pathway model and are relevant to both primary and secondary care.
Reay A, Dismore L, Aujayeb A, Dotchin C, Tullo E, Steer J, et al.. Analysing the patient experience of COVID-19: Exploring patients' experiences of hospitalisation and their quality of life post discharge. J Clin Nurs. 2024;33(9):3634-3641.	United Kingdom	Hospital/clinic/ telephone	People with Long COVID	Qualitative	Semi-structured interviews	Mean, SD (59.9, 10.7)	10 (6)	Patients discussed their experience of hospitalisation and how this continued to impact their emotional well- being post-	Patients hospitalised following a diagnosis of COVID- 19 experienced psy-chological distress during their hospital

92

doi:10.1111/jocn.17194								discharge. However, patients appeared to push themselves physically to improve their health, despite continued COVID- 19 symptoms.	stay, as well as 3-months post-discharge. We suggest the use of psychosocial interventions to support patients post-discharge.
Miller A, Song N, Sivan M, Chowdhury R, Burke MR. Identifying the needs of people with long COVID: a qualitative study in the UK. BMJ Open. 2024;14(6):e082728. doi:10.1136/bmjopen-2023-082728	United Kingdom	University of Leeds (online and in-person) focus groups	Adults with Long COVID	Qualitative focus groups; Framework Analysis	Eight focus groups (online + in-person)	Range 19–76 years	25 (8)	Three key themes/needs were identified. (Theme 1) Support systems including community groups, disability benefits, clinical services and employment support should be accessible and tailored to the needs of people with LC. (Theme 2) Research should investigate the physiology of symptoms, new clinical tests and treatment interventions to improve clinical understanding of the condition and symptom management. (Theme 3) Societal awareness should be promoted via local and national initiatives to educate the public about the condition and reduce stigma.	Participants experienced varied and individual challenges to daily life due to LC. There is a need for government acknowledgement of LC as a disability to ensure people with LC have access to disability support and legal protection. Policy development should be patient-driven and acknowledge the individual needs of people with LC in order to improve their quality of life.
Leggat FJ, Heaton-Shrestha C, Fish J, Siriwardena AN, Domeney A, Rowe C, et al.. An exploration of the experiences and self-generated strategies used when navigating everyday life with Long Covid. BMC Public Health. 2024;24(1):789. doi:10.1186/s12889-024-18267-6	England, Wales	Zoom, telephone	People with Long COVID	Reflexive thematic analysis.	Interviews	Mean, SD (44, 13 yo)	18 (12)	Themes were constructed which depicted 1) the landscape behind the Long Covid experience and 2) the everyday experience of participants' Long Covid. The everyday experience comprised a combination of physi- cal, emotional, and social factors, forming three sub-themes: centrality of physical symptoms, navigating 'experts' and the 'true colour' of personal communities, and a	Among this sample of adults with Long Covid, their experiences highlighted the unpredictable nature of the condition but also the use of creative and wide ranging self-generated strategies. The results offer people with Long Covid, and healthcare professionals supporting them, an overview of the

93

								rollercoaster of psychological ambiguity). The third theme, personal strategies to manage everyday life was constructed from participants' unique presentations and self-generated solutions to man-age everyday life. This comprised five sub-themes: seeking reassurance and knowledge, developing greater self-awareness through monitoring, trial and error of 'safe' ideas, building in pleasure and comfort, and prioritising 'me'.	collective evidence relating to individuals' self-management which can enable ways to live 'better' and regain some sense of identity whilst facing the impact of a debilitating, episodic condition.
Laestadius LI, Guidry JPD, Wahl MM, Perrin PB, Carlyle KE, Dong X, et al.. "The dream is that there's one place you go": a qualitative study of women's experiences seeking care from Long COVID clinics in the USA. BMC Med. 2024;22(1):243. doi:10.1186/s12916-024-03465-1	USA	Hospital/clinic	People with Long COVID	Framework analysis	Semi-structured interviews	Range from teens to 60s yr	30 (30)	Participants expressed five key themes concerning their experiences with seeking care from Long COVID clinics: (1) Access to clinics remains an issue, (2) Clinics are not a one stop shop, (3) Not all clinic providers have sufficient Long COVID knowledge, (4) Clinics can offer validation and care, and (5) Treatment options are critical and urgent.	While the potential for Long COVID clinics is significant, findings indicate that ongoing barriers to care and challenges related to quality and coordination of care hamper that potential and contribute to distress among women seeking Long COVID care. Since Long COVID clinics are uniquely positioned and framed as being the place to go to manage complex symptoms, it is critical to patient wellbeing that they be properly resourced to provide a level of care that complies with emerging best practices.
Kalfas M, Jolley C, Hart N, Rafferty GF, Duncan EL, Nicholson T, et al.. Exploring the Experiences of Living With the Post-COVID Syndrome: A Qualitative Study. Health Expect. 2024;27(3):e14108.	United Kingdom	Zoom or Microsoft Teams	People with Long COVID + Health professionals	Thematic analysis	Interviews	Rang: 30-75	19 (13)	Five main themes were identified:'Symptom dismissal' , 'Lack of information and	Women with PCS reported symptom dismissal by HCPs, which may have delayed their

94

doi:10.1111/hex.14108								support' , 'Life before and after Long COVID' , 'Psychological impact' and'Acceptance'. A shift overtime to self-management of symptoms was evident. These themes represent different stages of patients' PCS journey. Narratives indicated that women highlighted dismissal by healthcare professionals (HCPs), which was not as prominent in men's narratives. In addition, women went into more detail about the psychological impact of PCS compared to men.	diagnosis and negatively affected their well-being. We were not able to explore the experiences of people from non-conforming gender groups. Raising awareness of these issues among HCPs, particularly general practitioners, could improve patient care in PCS.
Gamillscheg P, Łaszewska A, Kirchner S, Hoffmann K, Simon J, Mayer S. Barriers and facilitators of healthcare access for long COVID-19 patients in a universal healthcare system: qualitative evidence from Austria. Int J Equity Health. 2024;23(1):220. doi:10.1186/s12939-024-02302-4	Austria	In person or virtually via WebEx	Health professionals Long COVID	Qualitative thematic framework approach	Semi-structured interviews; focus group	Range 21-60yr	experts 15 (8) Patients 18 (13)	Main themes included scepticism and stigma by medical professionals, difficulties in finding knowledgeable doctors, limited specialist capacities in the ambulatory care sector, long waiting times for specialist care, and limited statutory health insurance coverage of treatments resulting in high out-of-pocket payments.	Potentially relevant socioeconomic and demographic drivers in access barriers lay the necessary foundation for future quantitative inequality research.
Fang C, Baz SA, Sheard L, Carpentieri JD. "They seemed to be like cogs working in different directions": a longitudinal qualitative study on Long COVID healthcare services in the United Kingdom from a person-centred lens. BMC Health Serv Res. 2024;24(1):406. doi:10.1186/s12913-024-10891-7	United Kingdom	Long COVID healthcare services in the UK	Adults with Long COVID+ Health professionals	Longitudinal qualitative study; reflexive thematic analysis	Qualitative interviews (longitudinal)	Range:18-79	(80, 56)-Patients Healthcare provider (12, NR)	Theme 1 addresses the persistent hurdle of accessing primary care as the initial point of contact for LC healthcare; Theme 2 underscores the complex-ity of navigating secondary care; and Theme 3 encapsulates the distinctive challenges of developing LC integrated care. These themes are interlinked, as people with LC often had to navigate or struggle	Provide evidence to inform a more effective and sustainable delivery of person-centred care for people with LC across various healthcare settings and over time.

95

								between the various systems, with practitioners seeking to collaborate across the breadth of their professional responsibilities.	
Cooper K, Duncan E, Hart-Winks E, Cowie J, Shim J, Stage E, et al.. Exploring the perceptions and experiences of community rehabilitation for Long COVID from the perspectives of Scottish general practitioners' and people living with Long COVID: a qualitative study. BMJ Open. 2024;14(5):e082830. doi:10.1136/bmjopen-2023-082830	Scotland	Primary care	People with Long COVID + Health professionals	Qualitative	Interviews	Aged 40-65 - Patients; GP NR	11 (10)- Patient 13 (8) GP	Four key themes were identified: (1) The lived experience of Long COVID, describing the negative impact of Long COVID on participants' health and quality of life; (2) The challenges of an emergent and complex chronic condition, including uncertainties related to diagnosis and management; (3) Systemic challenges for Long COVID service delivery, including lack of clear pathways for access and referral, siloed services, limited resource and a perceived lack of holistic care, and (4) Perceptions and experiences of Long COVID and its management, including rehabilitation.	There are several patient, GP and service-level barriers to accessing community rehabilitation for Long COVID. There is a need for greater understanding by the public, GPs and other potential referrers of the role of community rehabilitation professionals in the management of Long COVID. There is also a need for community rehabilitation services to be well promoted and accessible to the people with Long COVID for whom they may be appropriate. The findings of this study can be used by
Boutry C, Patel P, Holmes J, Radford K, Bolton CE, Evangelou N, et al.. Returning to work with long covid in the UK during lockdown and other COVID-19 restrictions: A qualitative study. PLoS One. 2024;19(8):e0307062. doi:10.1371/journal.pone.0307062	United Kingdom	Workplace/return-to-work context in the UK	Adults with Long COVID discussing return to work	Qualitative study	Interviews	Range: 19-58yr	34 (29)	Themes were identified from transcripts using framework analysis. Participants with long covid experienced difficulties returning to work, particularly from fatigue, cognitive symptoms and breathlessness. Symptoms were heterogeneous and fluctuated in severity over time. A lack of understanding from colleagues and managers sometimes led to a pre-mature return to work without adequate support,	They may benefit from a tailored intervention to support a return to work that targets symptom management, and engages with work colleagues, managers, and family members.

96

								associated with further long covid relapse. Outside-of-work, support was salient for a successful return to work. The standard phased return offered by employers may be too short and rigid for some people with long covid.	
Al-Jabr H, Thompson DR, Castle DJ, Ski CF. Experiences of people with long COVID: Symptoms, support strategies and the Long COVID Optimal Health Programme (LC-OHP). Health Expect. 2024;27(1):e13879. doi:10.1111/hex.13879	United Kingdom	Hospital/clinic	Adults with Long COVID	LC-OHP programme sessions	Thematic analysis	Rang:18-60+	26 (24)	Four main themes emerged:'Symptoms and impact of LC'; 'Other sources of support and perceived challenges'; 'Strategies to support LC' and'Perceptions of the LC-OHP' .	LC experiences were mostly described as fluctuating and burdensome that significantly impacted daily activities, and physical and mental health. The LC-OHP was perceived as beneficial. Access and experiences of other sources of support were varied. Increasing LC awareness amongst health practitioners and the wider community has the potential to improve the experiences of those affected by LC.
Thomas C, Faghy MA, Owen R, Yates J, Ferraro F, Bewick T, et al.. Lived experience of patients with Long COVID: a qualitative study in the UK. BMJ Open. 2023;13(4):e068481. doi:10.1136/bmjopen-2022-068481	United Kingdom	Long COVID clinic (Derbyshire, UK); patient diaries	Adults with Long COVID referred to a Derbyshire Long COVID clinic	Longitudinal qualitative diary study	Hand-written patient diaries analysed inductively	Mean 49±10 years	12 (11)	Three key themes were highlighted: (1) understanding who helps patients manage symptoms, (2) daily activities and the impact on quality of life and health status and (3) the effect of turbulent and episodic symptom profiles on personal identity and recovery.	The novel challenges presented by Long COVID are complex with varying inter- related factors that are broadly impacting functional status and quality of life. Support mechanisms must incorporate the lived experiences and foster true collaborations between health professionals, patients and researchers to improve patient outcomes.

97

Stelson EA, Dash D, McCorkell L, Wilson C, Assaf G, Re'em Y, et al.. Return-to-work with long COVID: An Episodic Disability and Total Worker Health® analysis. Soc Sci Med. 2023;338:116336. doi:10.1016/j.socscimed.2023.116336	USA	Workplace	Working individuals have developed long COVID	Thematic analysis, a directed content analysis approach	Internet-based mixed-method, cross-sectional survey	Too many data missing	510 (296)	Four primary work-related themes emerged: 1) strong desire and need to return to work motivated by sense of purpose and financial precarity; 2) diverse and episodic LC symptoms intersect with organization of work and home life; 3) pervasiveness of LC disbelief and stigma at work and in medical settings; and 4) support of medical providers is key to successful return-to-work. Participants described how fluctuation of symptoms, exacerbated by work-related tasks, made returning to work challenging.	Qualitative findings indicate needs for workplace accommodations tailored to fluctuating symptoms, continuously re-evaluated by workers and supervisors together. Reductions in medical barriers to access work accommodations is also critical since many medical providers remain unaware of LC, and workers may lack a positive COVID test result.
Skilbeck L, Spanton C, Paton M. Patients' lived experience and reflections on long COVID: an interpretive phenomenological analysis within an integrated adult primary care psychology NHS service. J Patient Rep Outcomes. 2023;7(1):30. doi:10.1186/s41687-023-00570-2	United Kingdom	Primary care, an NHS secure Microsoft Teams system	People with Long COVID	interpretative phenomenological analysis (IPA) methodology	Semi-structured interviews	Rang: 18=65+	18 (13)	Common themes included uncertainty, mental and social impacts, and the processes of self-advocacy, mastering their symptoms, subjective recovery and future coping.	Patients have the resources to uncover the unknowns of this new illness which could inform clinical practice and further research. This suggests that that long COVID needs to be approached from a biopsychosocial perspective which emphasises patient involvement.
Silwal S, Parajuli K, Acharya A, Ghimire A, Pandey S, Pandey A, et al.. Physical, mental and social status after COVID-19 recovery in Nepal: A mixed method study. PLoS One. 2023;18(9):e0290693. doi:10.1371/journal.pone.0290693	Nepal	Workplace	People with Long COVID + Carers/family	Thematic analysis	In-depth interviews	Range: 20-41+	19 (NR)	A qualitative finding revealed that the majority of COVID-19 symptomatic patients experienced a variety of physical symptoms such as fever, headache, body pain, fatigue, tiredness, sore throat, cough, loss of taste, loss of smell, sneezing, loss of appetite, and difficulty breathing, while others felt completely fine after being recovered. Furthermore, there was no variation in the	COVID-19 infection has had an impact on physical, mental, and social well-being. Hence, to aid in the early recovery of COVID-19 patients, provision of evaluating and reporting the clinical features, early detection and management of long COVID case is needed from the local and provincial and central government

98

								daily functional activities of the majority of the recovered patients, while a few were found conducting fewer activities than usual because they were concerned about their health.	of Nepal.
Schmachtenberg T, Müller F, Kranz J, Dragaqina A, Wegener G, Königs G, et al.. How do long COVID patients perceive their current life situation and occupational perspective? Results of a qualitative interview study in Germany. Front Public Health. 2023;11:1155193. doi:10.3389/fpubh.2023.1155193	Germany	Workplace, video call	People with Long COVID	Content analysis	Interviews	Rang: 21-67	25 (18)	Of the 25 participants, 19 experienced limitations in pursuing leisure activities, and 10 of the 23 interviewees with jobs reported being on sick leave for several months.	The focus should be on creating long COVID-sensitive workplaces, compensating for decreased incomes, and improving access to relief services such as vocational reintegration. We argue, that a shift of perspectives is necessary and that long COVID should be considered rather as a "social disease" with considerably impairments in the social life of those affected.
Schmachtenberg T, Königs G, Dragaqina A, Roder S, Müller F, Müllenmeister C, et al.. "There is no one who helps you with it": experiences of people with long COVID regarding medical care, therapeutic measures, and barriers in the German healthcare system: results of a qualitative study with four focus groups. BMC Health Serv Res. 2023;23(1):1160. doi:10.1186/s12913-023-10170-x	Germany	Primary care,	People with Long COVID	Content analysis	Focus groups	Rang: 18-57yr	19 (12)	Many respondents reported that their general practitioners did not take their long COVID symptoms seriously and did not refer them to specialists or made therapeutic recommendations.	(1) general practitioners should take the symptoms of long COVID seriously, assume a care coordinating role, make referrals, and establish contact with long COVID clinics; (2) care planners should focus on developing interprofessional evidence-based care and treatment approaches for long COVID; (3) existing care structures such as long COVID outpatient clinics should be expanded. The overarching goal must be to develop

99

									consistent guidelines for long COVID diagnosis, care, and treatment.
Messiah SE, Francis J, Weerakoon S, Mathew MS, Shaikh S, Veeraswamy A, et al.. Persistent symptoms and conditions among children and adolescents hospitalised with COVID-19 illness: a qualitative study. BMJ Open. 2023;13(9):e069073. doi:10.1136/bmjopen-2022-069073	USA	Community	People with Long COVID + Carers/family	Qualitative	In-depth interviews	Rang: 4m-18yr	25 (13)	Seven themes were identified concerning the child's prolonged COVID- 19 experiences: (1) post-traumatic stress disorder, (2) social anxiety, (3) severe symptoms on reinfection, (4) worsened pre- existing conditions, (5) lack of insurance coverage for costly treatments, (6) access and utilisation of support systems and (7) overall resilience and recovery. Four parent- specific themes were identified: (1) fear of COVID- 19 unknowns, (2) mixed messaging from health information sources, (3) schools being both a support system and a hindrance and (4) desire for and access to support systems.	Clinical and public health support strategies should be developed to support these children and their families as they reintegrate in school, social and community activities.
Kennelly CE, Nguyen ATP, Sheikhan NY, Strudwick G, Ski CF, Thompson DR, et al.. The lived experience of long COVID: A qualitative study of mental health, quality of life, and coping. PLoS One. 2023;18(10):e0292630. doi:10.1371/journal.pone.0292630	Canada	long COVID clinics and community , web-based online	People with Long COVID	Thematic analysis	Focus groups	Range: 18-55+	47 (28)	Five themes were identified as integral to the long COVID experience: The Emotional Landscape of Long COVID, New Limits to Daily Functioning, Grief and Loss of Former Identity, Long COVID-related Stigmatization, and Learning to Cope with Persisting Symptoms.	These findings illustrate the immense impact of long COVID on mental health and quality of life. Minimal differences were found between those with and those without pre-existing mental health conditions, as both groups were substantially impacted by the condition. Attention to the perspectives of people with lived experience of long COVID is necessary to inform future directions for research and clinical

100

									practice.
Horlick S, Krysa JA, Brehon K, Pohar Manhas K, Kovacs Burns K, Russell K, et al.. Exploring Rehabilitation Provider Experiences of Providing Health Services for People Living with Long COVID in Alberta. Int J Environ Res Public Health. 2023;20. doi:10.3390/ijerph20247176	Canada	Primary care, rehabilitation, and specialty care . Zoom	Healthcare providers who managed people living with long COVID	Content analysis	Semi-structured interviews	NR	15 (NR)	Main themes include: the importance of education for long COVID recognition; the role of symptom acknowledgement in patient-centred long COVID service delivery; the need to develop recovery expectations; and opportunities for improvement of navigation and wayfinding to long COVID services.	Provider experience of delivering long COVID care can be used to inform patient-centred service delivery for persons with long COVID symptoms.
Gyllensten K, Holm A, Sandén H. Workplace factors that promote and hinder work ability and return to work among individuals with long-term effects of COVID-19: A qualitative study. Work. 2023;75(4):1101-1112. doi:10.3233/wor-220541	Sweden	Workplace; University designed study and post-Covid clinic	People with Long COVID	Thematic analysis	semi-structured focus group Interviews	Rang 29-63; Mean age 54	19 (13)	Five main themes emerged from the analysis: Communication and support, Possibilities to adjust work, Accep-tance of new limitations, Increased need for recovery from work and Lack of knowledge and understanding of the effects of Covid.	The results suggested that it is useful to facilitate communication, support and work adjustments for individuals suffering from Long COVID. It is also important to accept limitations and fluctuations in work ability and encourage recovery during and after work.
Duncan E, Alexander L, Cowie J, Love A, Morris JH, Moss R, et al.. Investigating Scottish Long COVID community rehabilitation service models from the perspectives of people living with Long COVID and healthcare professionals: a qualitative descriptive study. BMJ Open. 2023;13(12):e078740. doi:10.1136/bmjopen-2023-078740	Scotland	Rehabilitation	People with Long COVID + Health professionals	Qualitative	Interviews	NR	38 (33)	Three key themes were identified: (1) accessing care for PwLC, (2) understanding Long COVID and its management and (3) strengths and limitations of existing Long COVID rehabilitation services.	The findings presented here can be used by those developing and delivering services for people with Long COVID.
Duan E, Garry K, Horwitz LI, Weerahandi H. "I Am Not the Same as I Was Before": A Qualitative Analysis of COVID-19 Survivors. Int J Behav Med. 2023;30(5):663-672. doi:10.1007/s12529-022-10129-y	USA	Hospital/clinic	People with Long COVID	Grounded theory	Survey with free answered questions	59 (20-91)yr	134 (53)	Five overarching themes of post-acute patient experiences were generated: (1) an increased awareness of a mind and body connection, (2) feelings of premature aging, (3) an overall decline in quality of life, (4) a continued fear of infection, and (5) methods of coping.	Patients described lasting changes to their mental health and overall quality of life in connection to physical complications after severe COVID-19 infection. Patients' reports of their experience call for a greater awareness of the psychological aspects of COVID-19 recovery to provide

101

									both physical and psychological rehabilitation services. Additional resources such as education around re-infection and financial resources are needed.
Brehon K, Miciak M, Hung P, Chen SP, Perreault K, Hudon A, et al.. "None of us are lying": an interpretive description of the search for legitimacy and the journey to access quality health services by individuals living with Long COVID. BMC Health Serv Res. 2023;23(1):1396. doi:10.1186/s12913-023-10288-y	Canada	Long COVID clin-Ics, Online/Virtual	People with Long COVID	Reflexive thematic analysis	Interviews	Mean age =49.3 (13.0)yr	56 (34)	The first theme illustrated the rollercoaster-like nature of participants' Long COVID symptoms and the resulting impact on function and health. The second theme highlighted participants' attempts to access Long COVID services. Guidance received from healthcare professionals and self-advocacy impacted initial access. When navigating Long COVID services within the broader system, partici-<br>pants encountered barriers to access around stigma; appointment logistics; testing and 'normal' results; and financial<br>precarity and affordability of services. The third theme illuminated common factors participants liked and disliked<br>about Long COVID services. We framed each sub-theme as the key lesson (stemming from all likes and dislikes) that,<br>if acted upon, the health system can use to improve the quality of Long COVID services.	With Long COVID services continuously evolving, our findings can inform decision makers within the health system to better understand the lived experiences of Long COVID and tailor services and policies appropriately.
Bogale KA, Zeru T, Tarkegn M, Balew M, Worku M, Asrat A, et al.. Awareness and care seeking for long COVID symptoms among Coronavirus disease survivors in Bahir Dar City, Northwest Ethiopia: phenomenological study. BMC Public Health. 2023;23(1):941. doi:10.1186/s12889-023-15889-0	Ethiopia	Face to face	People with Long COVID	Phenomenology; Thematic Analysis	In-depth interviews	85%<65	23 (10)	Although only one participant mentioned the common symptoms of long COVID-19 the survivors experienced general, respiratory, cardiac, digestive,	To alleviate the problems, they had taken different measures including medical care, homemade remedies, spiritual solutions,

102

								neurological, and other symptoms.	and lifestyle modification.
Wurz A, Culos-Reed SN, Franklin K, DeMars J, Wrightson JG, Twomey R. "I feel like my body is broken": exploring the experiences of people living with long COVID. Qual Life Res. 2022;31(12):3339-3354. doi:10.1007/s11136-022-03176-1	Canada	Community, Online/Virtual	People with Long COVID	Thematic analysis	Open-ended survey	Range:18-79	169 (149)	Four overlapping and interconnected themes were identified: (1) Long COVID symptoms are numerous and wearing, (2) The effects of long COVID are pervasive, (3) Physical activity is difficult and, in some cases, not possible, and (4) Asking for help when few are listening, and little is working.	The varied relapsing-remitting symptoms, unknown prognosis, and deep sense of loss over one's prior identity suggest interventions are needed to support this population.
Schiavi M, Fugazzaro S, Bertolini A, Denti M, Mainini C, Accogli MA, et al.. "Like before, but not exactly": the Qualy-REACT qualitative inquiry into the lived experience of long COVID. BMC Public Health. 2022;22(1):599. doi:10.1186/s12889-022-13035-w	Italy	Hospital/clinic, telephone	People with Long COVID	Phenomenology, empirical phenomenological approach	Interviews	Mean, SD; 62.8 (11.8)	56 (22)	Persistent symptoms, feelings of isolation, fear and stigma, emotional distress, a fatalistic attitude, and return to (adapted) life course were the key themes that characterized the participants' experience after hospital discharge.	hese phenomena may trigger a vicious circle, but the participants also reported adaptation processes that allowed them to gradually return to their life course. Whether all individuals are able to rapidly activate these mechanisms and whether rehabilitation can help to break this vicious circle by improving residual symptoms remain to be seen.
Schaap G, Wensink M, Doggen CJM, van der Palen J, Vonkeman HE, Bode C. "It Really Is an Elusive Illness"-Post-COVID-19 Illness Perceptions and Recovery Strategies: A Thematic Analysis. Int J Environ Res Public Health. 2022;19. doi:10.3390/ijerph192013003	Netherlands	Hospital/clinic	People with Long COVID	Reflexive thematic approach	In-depth interviews	Mean, SD: 61.8 (8.5 yr)	24 (7)	Four overarching themes were identified: (I) symptoms after hospital discharge; (II) impact of COVID-19 on daily life and self-identity; (III) uncertainty about COVID-19; and (IV) dealing with COVID-19.	No notable differences in illness beliefs were observed between recovered and non-recovered participants.
Santiago-Rodriguez EI, Maiorana A, Peluso MJ, Hoh R, Tai V, Fehrman EA, et al.. Characterizing the COVID-19 Illness Experience to Inform the Study of Post-acute Sequelae and Recovery. Int J Behav Med. 2022;29(5):610-623. doi:10.1007/s12529-021-10045-7	USA	Community	People with Long COVID	Thematic analysis	In-depth interviews	Median age 49yr	24 (9)	RESULTS: After integrating the thematic analysis with clinical data, we identified key themes: (1) across symptom profiles and severity, experiencing COVID-19 was associated with	CONCLUSION: Our data informs the emerging field of "long COVID" research and shows a need to provide information and continuous support to persons with post-

103

								psychological distress; (2) symptomatic infection carried uncertainty in symptom presentation and ongoing recovery (e.g., long COVID); and (3) health information-seeking behavior was facilitated by access to medical care and uncertainty with the recovery process.	acute sequelae to ensure they feel secure along the path to recovery.
Piras I, Piazza MF, Piccolo C, Azara A, Piana A, Finco G, et al.. Experiences, Emotions, and Health Consequences among COVID-19 Survivors after Intensive Care Unit Hospitalization. Int J Environ Res Public Health. 2022;19. doi:10.3390/ijerph19106263	Italy	Community	People with Long COVID	Qualitative	Semi-structured interviews	Mean Age=56.4	12 (3)	Four main themes emerged:<br>(i) emotion of fear; (ii) isolation and loneliness; (iii) unawareness about the gravity of the situation as a protective factor; (iv) "Long COVID" as consequences of the disease on physical and psychological health	It is essential to guarantee a holistic take in charge starting before the discharge and continuing care after discharge in the community where they live.
O'Hare AM, Vig EK, Iwashyna TJ, Fox A, Taylor JS, Viglianti EM, et al.. Complexity and Challenges of the Clinical Diagnosis and Management of Long COVID. JAMA Netw Open. 2022;5(11):e2240332. doi:10.1001/jamanetworkopen.2022.40332	USA	Cohort Assembly	Care Notes	Inductive content analysis	Professionals notes	Mean, SD=60.0, 14.5yr	200 (27)	The sample included 173 (86.5%) men; 45 individuals (22.5%) were identified as Black and 136 individuals (68.0%) were identified as White.	CONCLUSIONS AND RELEVANCE: This qualitative study of documentation in the VA EHR highlights the complexity of diagnosing long COVID in clinical settings and the challenges of caring for patients who have or are suspected of having this condition.
Loft MI, Foged EM, Koreska M. An Unexpected Journey: The Lived Experiences of Patients with Long-Term Cognitive Sequelae After Recovering from COVID-19. Qual Health Res. 2022;32(8-9):1356-1369. doi:10.1177/10497323221099467	Denmark	Clinic	People with Long COVID	Three-phase phenomenological-hermeneutic ap-proach	Semi-structured interviews	NR	19 (15)	Patients living with long-term cognitive sequelae after COVID-19 were in an unknown life situation characterised by feelings of anxiety, uncertainty and concerns about the future, signi cantly disrupting their life trajectory and forcing them to change their ways of life.	While awaiting studies on treatment, symptom management and recovery after persistent sequelae of COVID-19, clinicians and researchers may find inspiration in experiences of other health conditions with similar phenomenology, such as ME/chronic fatigue syndrome and chronic headaches.
Ireson J, Taylor A, Richardson E, Greenfield B, Jones G. Exploring invisibility and epistemic injustice in Long Covid-A citizen science qualitative analysis of patient stories	UK	Community	People with Long COVID	Phenomenology, Thematic analysis, A three-stage process of	66 patient stories submitted online	NR	66 (NR)	The overriding theme of the analysis highlights the complexities and challenges of living with	Long Covid does not easily fit into the dominant evidence-based

104

from an online Covid community. Health Expect. 2022;25(4):1753-1765. doi:10.1111/hex.13518				analysis				Long Covid.	practice and the biomedical model of health, which rely on objective indicators of the disease process. Patient testimonies are vital to understanding and treating Long Covid, yet patients are frequently disbelieved, and their testimonies are not taken seriously leading to stigma and epistemic injustice, which introduces a lack of trust into the therapeutic relationship.
Gerlis C, Barradell A, Gardiner NY, Chaplin E, Goddard A, Singh SJ, et al.. The Recovery Journey and the Rehabilitation Boat - A qualitative study to explore experiences of COVID-19 rehabilitation. Chron Respir Dis. 2022;19:14799731221114266. doi:10.1177/14799731221114266	United Kingdom	Rehabilitation	People with Long COVID	Thematic analysis	Interviews, focus group, Survey	NR	13 (6)	The first, The Recovery Journey is sub-divided into ve sub-themes of Expectations, Individual and Varied Journeys, Mental and Physical Improvements, Self-values and The Journey Continues. The second overarching theme, The Rehabilitation boat contains ve subthemes: Programme Delivery, Safe and Supportive, Validation and Assurance, Shared Re ections and Education.	The opportunity to share the experience with others in the same boat was highly valued in the context of an unexpected and potentially lonely COVID-19 recovery.
Day HLS. Exploring Online Peer Support Groups for Adults Experiencing Long COVID in the United Kingdom: Qualitative Interview Study. J Med Internet Res. 2022;24(5):e37674. doi:10.2196/37674	United Kingdom	Community, Zoom interviews	People with Long COVID	Thematic analysis	Semi-structured interviews	Range: 20-59yr	1 (3)	The identified themes were as follows: (1) filling professional care gaps, (2) societal awareness, (3) engagement behavior, (4) diversity, and (5) social connections.	Through prioritizing patient voices, long COVID care could be restructured to maximize peer support's benefits within broader care structures.
Chasco EE, Dukes K, Jones D, Comellas AP, Hoffman RM, Garg A. Brain Fog and Fatigue following COVID-19 Infection: An Exploratory Study of Patient Experiences of Long COVID. Int J Environ Res Public Health. 2022;19.	USA	Hospital/clinic	People with Long COVID + Carers/family	Systematic thematic analysis	Interviews	Average (range)=49.3 (40-68)yr	15 (10)	Fatigue and brain fog affected all domains and identified subthemes included symptoms' synergistic effects, difficulty with	Providers can better support COVID-19 survivors during their recovery by identifying their needs in a sensitive

105

doi:10.3390/ijerph192315499								multitasking, lack of support, poor self-perception, and fear of loss of income and employment.	and timely manner.
Callan C, Ladds E, Husain L, Pattinson K, Greenhalgh T. 'I can't cope with multiple inputs': a qualitative study of the lived experience of 'brain fog' after COVID-19. BMJ Open. 2022;12(2):e056366. doi:10.1136/bmjopen-2021-056366	United Kingdom	Primary care, Remotely held	People with Long COVID	Qualitative	Focus groups	Median (Range)=43-29-74yr	50 (42)	Qualitative analysis revealed the following themes: mixed views on the appropriateness of the term 'brain fog'; rich descriptions of the experience of neurocognitive symptoms (especially executive function, attention, memory and language), accounts of how the illness fluctuated—and progressed over time; the profound psychosocial impact of the condition on relationships, personal and professional identity; self-perceptions of guilt, shame and stigma; strategies used for self- management; challenges accessing and navigating the healthcare system; and participants' search for physical mechanisms to explain their symptoms.	Services for such patients should include: an ongoing therapeutic relationship with a clinician who engages with their experience of neurocognitive symptoms in its personal, social and occupational context as well as specialist services that include provision for neurocognitive symptoms, are accessible, easily navigable, comprehensive and interdisciplinary.
Aghaei A, Aggarwal A, Zhang R, Li X, Qiao S. Resilience resources and coping strategies of COVID-19 female long haulers: A qualitative study. Front Public Health. 2022;10:970378. doi:10.3389/fpubh.2022.970378	USA	Social Media, Online/Virtual	People with Long COVID	Thematic analysis	Semi-structured interviews	20-65+	15 (NR)	At the individual level, they utilized cognitive and emotional resources to increase knowledge, learn new skills, set goals, and manage emotions; behavioral resources (e.g., internal motivation and executive functioning) to perform physical, creative, and recreational activities, and adopt healthier eating habits; and spiritual resources to perform spiritual rituals and connect with God. At the social	The resilience of female long haulers can be enhanced through (1) offering financial and health-related resources, (2) developing online social-support groups, (3) counseling and care service training for healthcare professionals, and (4) implementing more psychosocial interventions by labor organizations.

106

								level, the support from existing relationships and/or online social support groups enhanced their social identity and provided material and informational resources. At the health systems level, the guidance from counselors and physicians and availability of clinics, medicines, and health equipment assisted them in symptom management and medication adherence.	
Shelley J, Hudson J, Mackintosh KA, Saynor ZL, Duckers J, Lewis KE, et al.. 'I Live a Kind of Shadow Life': Individual Experiences of COVID-19 Recovery and the Impact on Physical Activity Levels. Int J Environ Res Public Health. 2021;18. doi:10.3390/ijerph182111417	United Kingdom	From a Clinical study	People with Long COVID	Thematic analysis	Semi-structured interviews	47 (7) yr	48 (41)	Four overarching themes were identified: (i) Living with COVID-19, including managing activities of daily living; (ii) Dealing with the Unknown and self-management strategies; (iii) Re-introducing physical activity; and (iv) Challenges of returning to work.	Individually tailored support is therefore required to address the unique challenges posed by COVID-19.
Humphreys H, Kilby L, Kudiersky N, Copeland R. Long COVID and the role of physical activity: a qualitative study. BMJ Open. 2021;11(3):e047632. doi:10.1136/bmjopen-2020-047632	United Kingdom	A UK- based research interest database for people with long COVID.	People with Long COVID	Qualitative	Semi-structured interviews	Range: 18-74	18 (9)	Theme 1 describes how participants struggled with drastically reduced physical function, compounded by the cognitive and psychological effects of long COVID.	Findings highlight the need for greater clarity and tailoring of physical activity-related advice for people with long COVID and improved support to resume activities important to individual well-being.

107

Supplementary File S9. Patient Themes, Subthemes, Quotations and Citations

Top-level Theme	Author generated Theme	Author generated Sub-Themes	Quotes	Citation
Social Connection, Support & Validation	you are not alone		…Talking to others with similar experiences helps, and you're thinking, 'You're not alone with these, sort of, thoughts of hospital treatments and what it was like, and…the fatigue and dealing with it, so those were probably the two key things for me at the session. I enjoyed it because I was able to see what other people were going through and not feel like I was the only one going through it, you know, and then how others were dealing with it.	(Seers et al., 2025)
Social Connection, Support & Validation		- Psychological impact of invalidation	"He put me on such a downer I felt like, what's the point of going on?" – P018, man	(Kalfas et al., 2024)
Social Connection, Support & Validation	Clinics can offer validation and care	- Emotional relief from being believed<br>- Diagnostic confirmation<br>- Symptom legitimation	"I just burst into tears… you recognise this is a pattern, and I'm part of it. Oh, thank you." – P25, 50s, Northeast	(Laestadius et al., 2024)
Social Connection,		Seeking support and validation	"Online groups helped me feel less crazy—it's not just me."	(Leggat et al., 2024)

108

Top-level Theme	Author generated Theme	Author generated Sub-Themes	Quotes	Citation
Support & Validation				
Social Connection, Support & Validation	Diagnosis	- Not being believed<br>- Limited knowledge among HCPs	"I told my Dr but they said they weren't aware of that being a Covid problem–every time I reported something they said they didn't know if it was related or not. I felt exasperated." (Participant 58)<br>"The nurse, she told me I should stay home, and only if my lips turned blue, should I call for an ambulance. I felt all alone in my illness." (Participant 23)	(Ireson et al., 2022)
Social Connection, Support & Validation	Positive validation	- Validation from some HCPs<br>- Online support	"Throughout my rollercoaster journey, my GP has been understanding. I have had more contact with my surgery in the last 4 months than I have had in my life." (Participant 25)<br>"The thing seemed endless. My GP was good at treating symptoms and was the first person to tell me that 'covid insomnia' was 'a thing'..." (Participant 13)	(Ireson et al., 2022)
Social Connection, Support & Validation	Online peer to peer support	- Online communities as validation and support	"It has been the kindness of people through professional networks or online peer support that has helped, more than structured services for people living with such disability." (Participant 55)	(Ireson et al., 2022)
Social Connection, Support & Validation	The Rehabilitatio n Boat	- Programme delivery<br>- Safe and supportive environment<br>- Validation and	"[The] range of exercise, as I say, was exactly the right level and the team were superbly supportive..." (Participant I)<br><br>"I got to see other people in the same boat as me. I think that made a difference." (Participant J)	(Gerlis et al., 2022)

109

Top-level Theme	Author generated Theme	Author generated Sub-Themes	Quotes	Citation
		assurance<br>- Shared reflections<br>- Education	“So me coming out there [rehab programme], I felt like somebody who has been released out of the cage and I felt so free.” (Participant G)<br><br>“In the group…we were able to discuss between us other symptoms as well and it was nice to be able to reflect...” (Participant B)<br><br>“There’d been an open forum… it was to get you thinking as to what you’d been through and what you could do to really help yourself.” (Participant J)	
Social Connection, Support & Validation	Interaction of physical symptoms and mental health	Fatigue and mental health connection	“Maybe 80, 90% because…the body is no longer 100%...but maybe 10% is mental. So that also counts because if you are not mentally well as well, then you can be affected.” (Participant #20)	(Santiago-Rodriguez et al., 2022)
Social Connection, Support & Validation	Filling Professional Care Gaps	- Inadequate Health Care Support<br>- Knowledge Generation & Distribution	I think you probably do have long COVID. And that was like full stop. I kind of paused waiting for some - there was nothing.” [Natalie]<br>“Often charities and support groups end up filling all the gaps.”<br>“At the moment we’re all kind of swinging in the dark and kind of hoping to find something.” [Oliver]<br>“It can be hard to-to differentiate between what is a sensible piece or post and what is a post that’s maybe got ulterior motives to it.” [Will]	(Day, 2022)
Social Connection, Support &	Engagement Behavior	- Temporal Variation<br>- Group	I didn’t do anything on the group until probably Christmas time.” [Chloe]<br>“I dip into every now and then.” [Will]<br>“Because at that point I think you need a wee boost.” [Chloe]	(Day, 2022)

110

Top-level Theme	Author generated Theme	Author generated Sub-Themes	Quotes	Citation
Validation		Participation<br>- Impact of Mood	"On a day when I'm not feeling great I probably avoid looking at it." [Natalie]	
Social Connection, Support & Validation	Diversity	- International Reach<br>- Different Stages of Recovery<br>- Administration and Moderation	"It'd be good to hear from other medical systems as well." [Natalie]<br>"Sometimes it worries me a bit because there's people on there who've had these symptoms for 18 months." [Mia]<br>"People could simply lie to get into groups." [Oliver]<br>"Admins are also people suffering with long COVID." [Sophia]	(Day, 2022)
Social Connection, Support & Validation	Social Connections	- Finding Others<br>- Reassurance<br>- Impact on Mental Well-Being	"I was the only kind of person that I knew around me who'd had COVID." [Emily]<br>"It was nice to know that people were going through exactly the same thing, that it wasn't almost in my head." [Jessica]<br>"These have been a lifeline for so many people, because when the medical services were failing, this was a beacon of light." [Chloe]<br>"Sometimes it can be a little bit overwhelming... I've literally had people saying they feel suicidal." [Natalie]	(Day, 2022)
Social Connection, Support & Validation		Specialist care<br>- Chasing appointments and investigations<br>- Relief and validation from being taken seriously	"The only reason I had the first MRI scan was because I kept chasing…" (P-6)<br>"It was the first time when a doctor sat with me for like 45 minutes… that was really good." (P-4)	(Turk et al., 2024)

111

Top-level Theme	Author generated Theme	Author generated Sub-Themes	Quotes	Citation
		- Value of comprehensive assessments		
Social Connection, Support & Validation		Primary care<br>- Delayed recognition of Long Covid<br>- Need to push for tests and referrals<br>- GP overwhelm and time limits<br>- Patient-led coordination of care	"It was probably about 11 months before somebody actually recognised it was Long Covid…" (P-7)<br>"I had to really push… please refer me to a Long Covid clinic." (P-4)<br>"I had to push for the blood tests… emailing my GP with the list of things they should include on the referral." (P-4)	(Turk et al., 2024)
Social Connection, Support & Validation	Patients' efforts to navigate emerging care pathways	3.1.1 Online resources and support groups<br>- Peer validation and emotional support<br>- DIY knowledge acquisition<br>- Barriers to online engagement (fatigue, brain fog)	"No formal advice on it at all. It's mostly been me [online group] that's helped…" (P-4)<br>"Being able to connect with other people that have Long Covid… that has been really great." (P-3)<br>"I don't know how I done that… I was definitely dangerous to drive… because of the brain fog." (P-7)	(Turk et al., 2024)
Social	Peer support		"Meeting others going through the same thing made me feel less alone."	(Buettikofer

112

Top-level Theme	Author generated Theme	Author generated Sub-Themes	Quotes	Citation
Connection, Support & Validation	and group therapy augments recovery		“Group sessions gave me a sense of hope I hadn’t felt in months.”	et al., 2025)
Social Connection, Support & Validation		Peer support	- "It’s so nice to speak to people that are experiencing something similar to you. And it makes you feel so much less isolated." — Kelly<br>- "Even just knowledge sharing like we’ve been doing today, just tips for coping." — Graham<br>- "If somebody’s not as bad as you, it… makes me feel a bit like, oh, why have I got it so bad? Is there something wrong with me? And that had quite a big impact on myself because I thought we were all in this together." — Kelly	(Miller et al., 2024)
Social Connection, Support & Validation	Acceptabilit y	Medical dismissal; gendered assumptions; family expectations	“I was also accused by a neurologist [...] that I was depressed because I was 27, didn’t have a partner [...] I’m imagining it all.” (P7)	Gamillscheg et al., 2024)
Social Connection, Support & Validation	Ability to Engage	Pacing vs. impression; admin fatigue; peer advocacy	“You haven’t actually worked for two days beforehand so you can attend this appointment.” (P13)	Gamillscheg et al., 2024)
Social Connection, Support & Validation		Lack of community referrals	“We haven’t had any referrals yet… we are all geared up ready for them.” – Luke (Male, White British, 60s)	(Fang et al., 2024)

113

Top-level Theme	Author generated Theme	Author generated Sub-Themes	Quotes	Citation
Social Connection, Support & Validation		Thus, for her, long COVID affects her family planning...”	We don’t have children, I deliberately decided against having children because I realize that I’m busy enough with what we have.” (P9)	(Schmachtenberg, Müller, et al., 2023)
Social Connection, Support & Validation	Changes to self and social roles	Reconciling past and present identities	Some days I had very, very, very dark thoughts because I just absolutely did not see how it was going to truly resolve...” (Pre-MH)	(Kennelly et al., 2023)
Social Connection, Support & Validation		Loss of social connections	“I just found I couldn’t cope at work... when my work was gone, everything else was sort of gone. My connection to people...” (No pre-MH)	(Kennelly et al., 2023)
Social Connection, Support & Validation	Limitations in performing personal interests	“Nineteen of the 25 participants stated that as a result of their health conditions, they experienced significant limitations in personal interests...”	“Since then, none of this is possible any longer, that is, nothing at all. That means pursuing any sports is completely out of question. [...] I liked to write texts, but I can’t do that very well anymore either.” (P13)	(Schmachtenberg, Müller, et al., 2023)
Social Connection, Support &	Family and social	Mixed experiences	“Relatives used to be afraid of me after hearing I was infected…” vs. “My sister, brother, mother... were more focused on the fact that I returned alive	(Silwal et al., 2023)

114

Top-level Theme	Author generated Theme	Author generated Sub-Themes	Quotes	Citation
Validation	support		and well.”	
Social Connection, Support & Validation	Understanding Long COVID and its management	Reluctance to diagnose Long COVID	"...they’re very sympathetic, but very clear and honest that well, we don’t really know..." [P4101]	(Duncan et al., 2023)
Social Connection, Support & Validation		Peer support and informal learning	"They do seek professional advice on how to manage them." [S3203]	(Duncan et al., 2023)
Social Connection, Support & Validation		Family interdependence	“Saw son + his girlfriend so cooked lunch for them—was fab to see them but wiped me out—everything ached and was shattered!!”	(Al-Jabr et al., 2024)
Social Connection, Support & Validation	Mind–Body Connection	Distrust and anxiety about physical ability	“It’s going to take a while for me to learn how to do what I used to be able to do without thinking…. I’m getting used to feeling normal [Patient ID 126].”	(Duan et al., 2023)
Social Connection, Support & Validation		(b) Social and community support	“The nurses and aides were very good to me… now my husband and children help me… I feel very grateful [Patient ID 125].”	(Duan et al., 2023)
Social		Validation and	“Listen to your patients... don’t tell them that it’s just in their heads”	(Brehon et

Top-level Theme	Author generated Theme	Author generated Sub-Themes	Quotes	Citation
Connection, Support & Validation		support		al., 2023)
Social Connection, Support & Validation	Family Interactions	Families offering emotional and practical help	“They know exactly what happened going through... got the real picture.” (P2)	(Boutry et al., 2024)`
Social Connection, Support & Validation	Theme I: Awareness about Long COVID-19	- Limited knowledge of symptoms<br>- Misattribution of symptoms to other conditions<br>- Confusion about virus transmissibility<br>- Unclear understanding of risk groups<br>- Perceptions about self-resolution	“I do not know the symptom of long COVID. But, I face difficulty when I want to breathe. I was treated for Asthma the last time but it was not Asthma.” [64 y/o female]<br>“I think individuals with long COVID-19 symptoms can transmit the virus.” [40 y/o male]<br>“My friend has long COVID-19 symptoms but he perceived that these symptoms came due to the COVID-19 vaccine.” [73 y/o male]<br>“Though the symptoms will go off by themselves, it is good to strengthen yourself through physical exercise…” [40 y/o male]	(Bogale et al., 2023)
Social Connection, Support &	Isolation	Lockdown-related social isolation	“When I was discharged… I couldn’t even go to the cemetery” (A044, female, 62)	(Schiavi et al., 2022)

116

Top-level Theme	Author generated Theme	Author generated Sub-Themes	Quotes	Citation
Validation				
Social Connection, Support & Validation		Fear of job loss and need for validation	"My husband also feels tired…we all do, right? … I just had to sleep until [the next] morning…So I couldn't do housekeeping or shop.	(Burton et al., 2024)
Social Connection, Support & Validation	Validation through others	Reassurance in shared experience	"It's just reassuring to know that you're not alone in this... it helps put things in perspective."	(Cooper et al., 2024)
Social Connection, Support & Validation	Increasing awareness of LC	Inconsistent professional recognition	"She was just more reactive as opposed to proactive." – Craig (Male, White British, 50s)	(Fang et al., 2024)
Social Connection, Support & Validation	Concerns about Unemployment and Income	Loss of income burdens families<br>- Costs of care and travel are financially stressful<br>- Need for official recognition and benefits	"I don't have a job and my partner unfortunately works two jobs now."	(Chasco et al., 2022)
Social Connection,	Encouraging staff and		"Staff were always so positive and encouraging – it made a big difference."	(Buettikofer et al., 2025)

117

Top-level Theme	Author generated Theme	Author generated Sub-Themes	Quotes	Citation
Support & Validation	light-filled facilities support recovery		"The space felt light and open, not clinical – it really helped my mood."	
Social Connection, Support & Validation	Support systems used/needed	Lack of peer support for parents	"…a support group, other parents that were actually going through the same thing that my daughter and I were going through."	(Messiah et al., 2023)
Social Connection, Support & Validation	solidarity and community		I think the most, the number one most effective [aspect] is just being in the community and knowing that you're not alone in what you're feeling or doing…. It was fairly profound…	(Sarma et al., 2025)
Social Connection, Support & Validation	peer support and bonding		The main thing that I've noticed that people really benefit from being able to share their experiences…it's actually the peer support that they probably found most useful. But that was then the springboard for people to want to improve their fitness. P2<br>We're getting different ideas off each other in the groups. It enabled them to obviously progress, they got different ideas… they had that bank of support off people within the group. P5<br>Actually, we have actually swapped emails and we've had a Zoom between us which is really nice…like anything, anybody who's been through a particular trauma….nobody really knows what you've been through and only the people who've been	Seers et al., 2025)

118

Top-level Theme	Author generated Theme	Author generated Sub-Themes	Quotes	Citation
			through it know what you've been through…Int13	
Social Connection, Support & Validation	Interconnection of physical and psychological symptoms		"The slightest thing was an effort... it's the most fatigued I have ever been... things like changing my bedding... it was that sort of level of difficulty with day-to-day tasks." (IV2)<br>"If I do something physical I suffer... If I start to think too much I then get a foggy head... If I type an email... I then can't think enough to shut the computer down." (IV5)<br>"Stress brings it on and that makes it really bad." (IV13)	(Humphreys et al., 2021)
Social Connection, Support & Validation		Relief from Validation and Support	"The locum GP was very good... very supportive... consistency was good." (Participant 7)	(Callan et al., 2022)
Social Connection, Support & Validation	Changing Perceptions of Others	- Some participants changed others' skepticism through their experience<br>- Advocated for recognition and vaccination	"A friend convinced others to get their COVID shots by sharing my story."	(Chasco et al., 2022)
Social Connection, Support & Validation	Spiritual coping strategy	1) performing spiritual rituals<br>(2) connection with God	"I go to church... emotionally I'm needing more, like, soul nourishment." (ID#1)<br>"Prayer is when I speak to God... God's intentions are good for me." (ID#2)	(Aghaei et al., 2022)

119

Top-level Theme	Author generated Theme	Author generated Sub-Themes	Quotes	Citation
Social Connection, Support & Validation	lack of recognition and understanding	encountered by Long Covid participants regarding their ongoing need to recover		(MacLean et al., 2025)
Social Connection, Support & Validation	Effect on Relationships Outside Household	- Mixed support from extended family and friends<br>- Some relationships fracture due to disbelief or discomfort	"It takes a lot more energy for [friends] to look at someone their own age, and it looks like they're failing."	(Chasco et al., 2022)
Social Connection, Support & Validation	Seeking social support coping strategy	(1) family and friends<br>(2) co-workers<br>(3) therapists and counselors<br>(4) online support groups with other patients<br>(5) spiritual leaders and community	"My friends and family have helped emotionally, physically..." (ID#1)<br>"I feel more comfortable talking with my co-workers than with family." (ID#8)<br>"Companionship of other patients in a similar situation made me feel that I am not alone." (ID#10)<br>"Praying with other people help feeling not alone." (ID#4)	(Aghaei et al., 2022)
Stigma,	recognition		…just to have a nice place with people that understand that you just slept,	(Sarma et al.,

120

Top-level Theme	Author generated Theme	Author generated Sub-Themes	Quotes	Citation
Misunderstanding & Epistemic Injustice	and validation		like, 20 out of 24 hours and you haven't combed your hair in 2 days. Maybe 4 days 6 of 13 instead of 2… Like, it just validates you that…this is normal for where I'm at in this illness.	2025)
Stigma, Misunderstanding & Epistemic Injustice	Lack of knowledge and understanding of the effects of COVID	In the workplace	"Many times, words would be enough. They cannot do anything about my symptoms, but they can show compassion."	(Gyllensten et al., 2023)
Stigma, Misunderstanding & Epistemic Injustice		- Experiential contrast to prior dismissal	"I don't know where I'd be without the COVID clinic..." – P25, 50s, Northeast	(Laestadius et al., 2024)
Stigma, Misunderstanding & Epistemic Injustice		- Dismissive conventional care = resort to alternatives	"They're not really looking at cutting-edge treatments." – P22, 50s, West	(Laestadius et al., 2024)
Stigma, Misunderstanding &	Symptom Dismissal	- Repeated disbelief from healthcare professionals	"I expected no answers or solutions, but I didn't expect to have to fight to be believed." – P008, woman<br>"He leaned back and laughed at me. Told me to go to the gym." – P018,	(Kalfas et al., 2024)

121

Top-level Theme	Author generated Theme	Author generated Sub-Themes	Quotes	Citation
Epistemic Injustice		(HCPs)<br>- Dismissive, even mocking, treatment by GPs<br>- Gendered experiences of disbelief	man<br>“A doctor said, ‘Sometimes women choose to stay sick because of life pressures.’” – P005, woman	
Stigma, Misunderstanding & Epistemic Injustice		- Self-reliance and self-blame<br>- Stigma and hidden disabilities	“Whining doesn’t help…I must contribute 80%, and that’s it.”<br>“I feel really dependent on my boyfriend…which is deeply frustrating.”	(Loft et al., 2022)
Stigma, Misunderstanding & Epistemic Injustice		- Dismissal by both male and female HCPs<br>- Women’s symptoms attributed to stress or social roles	“Us women… we have to accept the pressure of expectations.” – P005, woman	(Kalfas et al., 2024)
Stigma, Misunderstanding & Epistemic Injustice		- Long-term avoidance of healthcare due to past dismissals	“I didn’t go to the GP for years… it was easier than being told I was making things up.” – P002, woman	(Kalfas et al., 2024)

122

Top-level Theme	Author generated Theme	Author generated Sub-Themes	Quotes	Citation
Stigma, Misunderstanding & Epistemic Injustice		Stigma and discrimination	“You have to go home … you have COVID… I was mortified”	(Brehon et al., 2023)
Stigma, Misunderstanding & Epistemic Injustice		Fear of social stigma	“People did not want to be around [me] if they know [I] had coronavirus [Patient ID 70].” / “It’s been a problem… people despise them because they are afraid… [Patient ID 23].”	(Duan et al., 2023)
Stigma, Misunderstanding & Epistemic Injustice	Stigma and Discrimination		“My neighbours avoided me… They have a fear that COVID may spread to them” (33/Male)<br>“They sprayed bleaching powder… so they avoided us” (33/Male)	J et al., 2025)
Stigma, Misunderstanding & Epistemic Injustice	Fear of stigma and reintegration	Fear of social stigma, isolation	“What I need to understand is how people with HIV assimilated back into the community. In the early days, how did they do that? I don’t know how to do that, and that’s the thing I’m most afraid of, that I won’t be able to... I’m afraid that I’m going to isolate myself further, because I don’t know how to join back in, and how to deal with the stigma. That I’m going to get more of it.” (Participant #10)	(Santiago-Rodriguez et al., 2022)
Stigma, Misunderstanding &		Incongruent testing and dismissal	“These tests are showing basically nothing... but I’m here to tell you it’s not. None of us are lying.”	(Brehon et al., 2023)

123

Top-level Theme	Author generated Theme	Author generated Sub-Themes	Quotes	Citation
Epistemic Injustice				
Stigma, Misunderstanding & Epistemic Injustice	Ability to Seek	Limited condition knowledge; self-doubt; stigma-induced silence	"I simply questioned every symptom that I had and asked myself [...] am I imagining it or do I really have it?" (P17)	Gamillscheg et al., 2024)
Stigma, Misunderstanding & Epistemic Injustice	Theme II: Experience and Effect of Long COVID Symptoms	- Physical symptoms: fatigue, cough, chest pain, headache, dizziness, insomnia, joint pain<br>- Neurological/cognitive issues: forgetfulness, confusion<br>- Psychosocial impact: isolation, stigma, suicidal ideation		(Bogale et al., 2023)
Stigma, Misunderstanding &	Fear and stigma	Fear of recurrence and infecting others	"I'm a bit terrified about getting sick again" (A021, female, 61); "I want to make sure I don't carry the virus" (A040, male, 78)	(Schiavi et al., 2022)

124

Top-level Theme	Author generated Theme	Author generated Sub-Themes	Quotes	Citation
Epistemic Injustice				
Stigma, Misunderstanding & Epistemic Injustice		Social suspicion and internalised stigma	“Everyone is suspicious. Basically, everyone is afraid” (A017, female, 71); “Get better… then we can all rest easy” (A029, male, 55)	(Schiavi et al., 2022)
Stigma, Misunderstanding & Epistemic Injustice	Challenges in coping	(1) insufficient financial resources<br>(2) perceived stigma against COVID survivors<br>(3) fear of deteriorating physical health<br>(4) unpleasant healthcare experiences in dealing with long COVID<br>(5) misinformation of long COVID<br>(6) overwhelming social interaction and burnout	“I had counseling in my plan..., but basically stopped! Some issue is about the billing.” (ID#2)<br>“I walk away from things, I lost a lot of friends...” (ID#6)<br>“I can’t deal with other people’s problems anymore because I’m almost over-empathetic now.” (ID#5)	(Aghaei et al., 2022)

125

Top-level Theme	Author generated Theme	Author generated Sub-Themes	Quotes	Citation
Stigma, Misunderstanding & Epistemic Injustice	Fear of Infection	Anxiety about reinfection	“Everybody is just nervous and if I sneeze my kids are looking at me like ‘Are you getting sick again?’… I’m really really worried [Patient ID 36].” / “God forbid I catch this again, I was close to death… [Patient ID 34].”	(Duan et al., 2023)
Stigma, Misunderstanding & Epistemic Injustice	Psychosocial Impact: Guilt, Shame, and Stigma	Occupational Limitations and Identity Threat	“I’m really, really fearful for the future or whether I’m going to be able to get back to what I want to do... a big part of me is being a (allied health professional) and if I can’t... I’ve lost a huge part of me.” (Participant 11)	(Callan et al., 2022)
Stigma, Misunderstanding & Epistemic Injustice	Not being believed	Judged by peers and professionals	“There was a middle part where people were quite scathing... ‘well, so-and-so had it and they’re fine’.”	(Cooper et al., 2024)
Stigma, Misunderstanding & Epistemic Injustice	External and internal stigma related to long COVID	Trivialization of experience	“Most people think oh, it’s just the common cold and flu and it’s all in your head... when I hear people say it’s not real or it’s just a flu, I am hearing that they tell me I am not real.” (No Pre-MH)	(Kennelly et al., 2023)
Stigma, Misunderstanding & Epistemic	Stigma at Work	- Colleagues often misunderstand or doubt symptoms<br>- Performance	“Well, I saw you last Saturday and you didn’t look too bad then.”	(Chasco et al., 2022)

126

Top-level Theme	Author generated Theme	Author generated Sub-Themes	Quotes	Citation
Injustice		scrutinized<br>- Some resign or reduce work		
Stigma, Misunderstanding & Epistemic Injustice		Anxiety related to symptoms and stigma	"Sometime I doubt I get corona again when I have a mild headache or sore eyes or pain in chest so it impacts my mental health."	(Silwal et al., 2023)
Stigma, Misunderstanding & Epistemic Injustice	dismissal and disregard		I look just fine, you know, people all the time, they look at me like,'oh, you look great' and I'm like, it doesn't mean that I feel great…. We had a talk [in the support group] about- we don't have to prove to people how you're feeling, and this is real, right- this is really happening? Because people question, you know, people see you looking just fine. This makes you question your sanity, like am I really going through this?<br>Why am I really this tired? Right? Like, am I really not being able to read right now? You just question yourself.<br>(P13)	(Sarma et al., 2025)
Stigma, Misunderstanding & Epistemic	Social health status of COVID-19 recovered	Awareness and social attitudes	"82 COVID-19 recovered participants stated that their society was unaware of their COVID-19 infection."	(Silwal et al., 2023)

127

Top-level Theme	Author generated Theme	Author generated Sub-Themes	Quotes	Citation
Injustice	participants			
Stigma, Misunderstanding & Epistemic Injustice	Stigma and Difficulty Being Believed	- Perceived and explicit stigma from misinformation and skepticism<br>- Symptoms' unpredictability fuels disbelief	"My sibling is dead to me ... still believes it's fake ... that I only had the flu."	(Chasco et al., 2022)
Symptom Burden & Functional Loss		Common long COVID symptoms	Fatigue was so much more than being tired… unrelenting and unlike anything else they had ever experienced." "Shortness of breath… not among their most concerning symptom(s)."	(Wurz et al., 2022)
Symptom Burden & Functional Loss	Physical Challenges	Fatigue and breathlessness impeding daily work tasks	"I still get tachycardia when I'm moving around, I still get short of breath on exertion." (P2)	(Boutry et al., 2024)`
Symptom Burden & Functional Loss	Chest pain and tightness	Ranged from minor discomfort to hospitalization	"I felt like I had a dumbbell sitting on my chest..."	(Khashei et al., 2023)
Symptom Burden & Functional	Long COVID symptoms	Symptoms are numerous	"Many indicated it was far too many to count." "The sheer volume of symptoms was described as overwhelming."	(Wurz et al., 2022)

128

Top-level Theme	Author generated Theme	Author generated Sub-Themes	Quotes	Citation
Loss	are numerous and wearing			
Symptom Burden & Functional Loss	Physical symptoms persisting	Loss of smell and taste, tiredness, etc.	"A few of them experienced a loss of appetite and tiredness, and some experienced headaches and eye pain… loss of smell and loss of taste."	(Silwal et al., 2023)
Symptom Burden & Functional Loss	Chest pain without breathlessness	Chest pain sometimes unaccompanied by shortness of breath	"My chest feels kinda heavy and sore-like someone punched me...I have no real shortness of breath."	(Khashei et al., 2023)
Symptom Burden & Functional Loss		Value of manager understanding and research into symptoms	"My managers have been really supportive... just to see how they can support me." (P20)	(Boutry et al., 2024)`
Symptom Burden & Functional Loss	Types and locations of pain	Joint burning, sharp pains, abdominal pain, eye muscle pain	"All my joints were burning horribly, knees, shoulders, hips..."; "Terrible neck pain. Couldn't even walk down the steps without wanting to cry."	(Khashei et al., 2023)
Symptom Burden & Functional Loss	Pain qualities	Sharp, stabbing, burning, nerve pain	"Sharp, charlie-horse like pain in my left shoulder that seemed to wrap around my left side..."	(Khashei et al., 2023)

129

Top-level Theme	Author generated Theme	Author generated Sub-Themes	Quotes	Citation
Symptom Burden & Functional Loss	Symptom progression patterns	Complete resolution vs waxing and waning; relapses	"One day I'll feel 95% better, take a walk... Another day I'll try to do one thing and it will send me back to bed."	(Khashei et al., 2023)
Symptom Burden & Functional Loss		Impaired decision-making and social cognition	"What if the brain fog and the memory loss is that bad that I make poor decisions?" (P5)	(Boutry et al., 2024)`
Symptom Burden & Functional Loss	Cognitive Challenges	Brain fog, poor memory and concentration	"I struggle to get through one paragraph, I would have to re-read, re-read, re-read." (P13)	(Boutry et al., 2024)`
Symptom Burden & Functional Loss	Lung irritation and pain	Burning, itching, inflammation sensations	"Lungs started to feel 'irritated.' It wasn't difficult to breathe, but I could tell my lungs were inflamed."	(Khashei et al., 2023)
Symptom Burden & Functional Loss	The effects of long COVID are pervasive	Changed capacity to manage roles and responsibilities	"Unable to take care of their home, families, and in some cases, themselves." "Unable to manage their day-to-day life was extremely upsetting and distressing."	(Wurz et al., 2022)
Symptom Burden & Functional		Fatigue	"Everything is harder than it used to be" (A069, female, 49)	(Schiavi et al., 2022)

130

Top-level Theme	Author generated Theme	Author generated Sub-Themes	Quotes	Citation
Loss				
Symptom Burden & Functional Loss		Other physical symptoms	My eyesight has got worse" (A012, male, 71); "My hands shook" (A012); "I'm losing my hair" (A078, female, 54)	(Schiavi et al., 2022)
Symptom Burden & Functional Loss	'A Part of Our Everyday Lives': Living with COVID-19	Limitations in Activities of Daily Living	Your body, it shuts down, you lift your arms up, every, everything you do, to go to the toilet, to make a cup of tea, is an effort' (M02; 6 months post-infection)<br>'. . . if I'm trying to move or do any activities, the breathlessness that's the... problem' (F05; 10 months post-infection)<br>'I haven't made a cup of tea since March... I folded four and I was short of breath' (F05; 10 months post-infection)<br>'I'm worried about that in terms of doing any sort of strength work...' (F04; 7 months post-infection)<br>'So, I mean I've been super cautious...' (F08; 10 months post-infection)	(Shelley et al., 2021)
Symptom Burden & Functional Loss		Managing energy and cognitive limitations	"I've started using my calendar very much… so I write down… a lot."	(Burton et al., 2024)
Symptom Burden & Functional Loss	PTSD-like symptoms	Hallucinations, fear	"She was hysterical... brushing stuff off her…She's like 'there's spiders crawling all over me'... she said she saw a monster... hearing and seeing things."<br>"She's more afraid to go out in public. She doesn't really want to go out anymore because of (having COVID)."	(Messiah et al., 2023)

131

Top-level Theme	Author generated Theme	Author generated Sub-Themes	Quotes	Citation
Symptom Burden & Functional Loss		Cognitive fatigue and declining functional capacity	"I am really frustrated… I'm suffering from headaches and fatigue… That is what really bothers me."	(Burton et al., 2024)
Symptom Burden & Functional Loss	Loss and alteration of taste and smell	Complete loss, attenuation, altered senses, phantom sensations	"It's slow coming back. When I lost it, it was like turning off a switch."; "I smell a cigarette smoke constantly."	(Khashei et al., 2023)
Symptom Burden & Functional Loss		Cognitive and sensory impairments	"There's a cake I've been baking for ten years...but I no longer can… I cannot figure out how to read a recipe."	(Burton et al., 2024)
Symptom Burden & Functional Loss	Unexpected onset and variability of LTCS	Sudden or delayed symptoms post-acute COVID	"I thought…yes; I have survived! And then I thought…I can handle everything…But then it came so suddenly."	(Burton et al., 2024)
Symptom Burden & Functional Loss	Severe symptoms on reinfection	Worsened symptoms after reinfection	"She was fully vaccinated… she got it again… high fever… still has the taste and smell."<br>"I got COVID again… they give me the same treatment again."	(Messiah et al., 2023)
Symptom Burden & Functional	Symptoms After Hospital	Somatic Symptoms	"I'm easily fatigued, I'm easily out of energy. [...] I already experience difficulties when climbing stairs." (P22NR, M)	(Schaap et al., 2022)

132

Top-level Theme	Author generated Theme	Author generated Sub-Themes	Quotes	Citation
Loss	Discharge			
Symptom Burden & Functional Loss		Symptoms vary in presentation and intensity	“Symptoms would relapse and remit, often seemingly at random.” “Others… remained heightened or tapered off.”	(Wurz et al., 2022)
Symptom Burden & Functional Loss	Symptom relapse	Return of symptoms after initial resolution	“Symptoms returned as if they were re-experiencing acute infection.”	(Khashei et al., 2023)
Symptom Burden & Functional Loss	Reasons for testing	Symptoms, exposure, work requirements, travel		(Khashei et al., 2023)
Symptom Burden & Functional Loss		Re-skilling or role change due to symptom burden	“There is absolutely no way I could go back to being a principal teacher.” (P10)	(Boutry et al., 2024)`
Symptom Burden & Functional Loss		Mismatch Between Symptoms and Medical Assessment	I got an email from the [health department] just telling me I'm well enough to go back to work... I'm still really not well enough.” (N40, F, 50+, HCW) “The resounding response from everybody is we don’t know… It’s okay, your lungs are okay.” (N6, F, 30–49, HCW)	(Hitch et al., 2023)
Symptom Burden &	Physical health status	Normal health after recovery	“The majority (89.1%) of the COVID-19 individuals who had recovered had generally good health.”	(Silwal et al., 2023)

133

Top-level Theme	Author generated Theme	Author generated Sub-Themes	Quotes	Citation
Functional Loss	of COVID-19 recovered participants			
Symptom Burden & Functional Loss		Ongoing Fatigue and Limited Capacity	“I found the fatigue really knocked me for six…” (N29, M, 50+)<br>“I'm working as a PPE spotter… I don't have the brain concentration.” (N6, F, 30–49, HCW)	(Hitch et al., 2023)
Symptom Burden & Functional Loss		Tension between hope of recovery and reality of limitations	"I keep hoping I’ll turn a corner, but every week I’m reminded that I have to pace like my life depends on it. Because it does."	(Skilbeck et al., 2023)
Symptom Burden & Functional Loss		Impact of symptoms on job performance and confidence	"I don’t feel like I can do my job anymore because my brain is completely fogged."	(Skilbeck et al., 2023)
Symptom Burden & Functional Loss		Fluctuating symptoms and recovery time hinder RTW	"I’ve come back 3 times. And after every time, I’ve ended up back in bed for weeks or months. It’s a terrible spiral of hope, effort, failure, collapse."	(Skilbeck et al., 2023)
Symptom Burden & Functional Loss		Intersection of symptoms, job tasks, and organisation of	Half a day of work in office causes 3–5 days in bed. The same work from home is O.K."	(Skilbeck et al., 2023)

134

Top-level Theme	Author generated Theme	Author generated Sub-Themes	Quotes	Citation
		work		
Symptom Burden & Functional Loss	Diverse and episodic long COVID symptoms intersect with the organization of work and home life	Intersection of symptoms and home life impact RTW	"The only way I can keep working is by giving up EVERYTHING else in my life. I work, then I lay on the couch or bed and everything I need is brought to me by my spouse. I take the entire weekend to do nothing but sleep/ recover and hope I get enough rest to make it through the next week."	(Skilbeck et al., 2023)
Symptom Burden & Functional Loss	Living situation and difficulties in daily activities	"Some interviewees described that they already have considerable difficulties with supposedly simple activities such as reading cooking recipes, preparing meals, maintaining body hygiene, or climbing stairs."	I need help in the household, too, I can't do it alone, it doesn't work" (P18); "I've tried to return to my own apartment several times already. But that does not work for me when I'm alone... I need help." (P25)	(Schmachtenberg, Müller, et al., 2023)
Symptom Burden &	Acceptance of new	Difficult to decrease work pace	"And I know that sometimes I do more than I should, because my body protests.	(Gyllensten et al., 2023)

135

Top-level Theme	Author generated Theme	Author generated Sub-Themes	Quotes	Citation
Functional Loss	limitations			
Symptom Burden & Functional Loss	Increased need for recovery	Lack of energy at work	“It feels like I will collapse. And if I push myself, no then I will faint.”	(Gyllensten et al., 2023)
Symptom Burden & Functional Loss		After performing such tasks, some participants described a pronounced fatigue symptomatology.”	“Mostly I can go up one floor well, sometimes I can go up two, sometimes I can only go up half a floor” (P20)	(Schmachten berg, Müller, et al., 2023)
Symptom Burden & Functional Loss	Severity spectrum	Asymptomatic to life-threatening		(Khashei et al., 2023)
Symptom Burden & Functional Loss	episodic and unpredictabl e symptoms and impairment		At [the time of day of the group] my energy levels and my fatigue and brain fog tend to be worse, so there were times where I would have liked to participate more but felt like I was just too fatigued to be able to say a whole lot.<br>My family still doesn't understand it because one day I can walk and talk, and the next day I can't, and I can't 4 of 13 get out of bed, and they don't understand that. And I	(Sarma et al., 2025)

136

Top-level Theme	Author generated Theme	Author generated Sub-Themes	Quotes	Citation
			don't fully understand that.<br>(P16)	
Symptom Burden & Functional Loss	Invisible illness	Not taken seriously due to hidden symptoms	You feel a bit of a pariah, to be honest, with Long COVID."	(Cooper et al., 2024)
Symptom Burden & Functional Loss		Postexertional symptom exacerbation	"Went to meet a friend for coffee, exhausted after and needed to rest."	(Al-Jabr et al., 2024)
Symptom Burden & Functional Loss	Daily activities and the impact on QoL and health status	Need for recovery and rest	"Rested from 5pm—After a couple of hours symptoms intensified again."	(Al-Jabr et al., 2024)
Symptom Burden & Functional Loss		"Several participants reported that... they had either no contact at all, only very limited contact..."	"Of course, some things have broken away [...] and it has become more difficult to keep in contact with colleagues." (P2); "Socializing doesn't work out so well. [...] I'm really all alone." (P13)	(Schmachtenberg, Müller, et al., 2023)
Symptom	Physical and	Living with brain	"For me, I am finding it's hard to concentrate and get down to my work.	(Kennelly et

137

Top-level Theme	Author generated Theme	Author generated Sub-Themes	Quotes	Citation
Burden & Functional Loss	cognitive impairments	fog	And memory issues as well... I have to write everything down and sometimes even then I don't even get it right." (No pre-MH)	al., 2023)
Symptom Burden & Functional Loss	Work and school impairment	Reduced capacity, symptom relapse after exertion	"Due to the tiredness, I can't do my finals...quite as well as I wanted to."	(Khashei et al., 2023)
Symptom Burden & Functional Loss	Learning how to balance symptoms and activity	Personal pacing and trial-and-error	"So as much as I'm enjoying [walking the dog], it has the knock-on effect... but that is getting less and less... relapses are all part of it..." (IV17)<br>"I read about pacing and I haven't tested myself, so I'm not exercising... fear of not knowing." (IV9)	(Humphreys et al., 2021)
Symptom Burden & Functional Loss	Physical activity and task limitations	Difficulty with lifting, cleaning, moving; fatigue after exertion	"Moving groceries, sometimes getting out of chairs... feel like they're twice as hard."	(Khashei et al., 2023)
Symptom Burden & Functional Loss	Medications and supplements	OTC pain relievers, antibiotics, steroids, inhalers	"The most frequently reported class of products... were pain relievers/fever reducers."	(Khashei et al., 2023)
Symptom Burden & Functional	Symptoms and impact of LC	Symptoms of LC	"…when I go out now, I have a wheelchair, I can't push my eight-year-old daughter on the swings, can't walk my dog, and obviously, can't go to work until the issues with my heart rate has been sorted…"	(Al-Jabr et al., 2024)

138

Top-level Theme	Author generated Theme	Author generated Sub-Themes	Quotes	Citation
Loss				
Symptom Burden & Functional Loss	Riding the Long COVID Rollercoaste r	Diverse, unpredictable symptoms	“It affects everybody […] to different extents and everybody has different symptoms, which is really hard because it’s just a myriad of symptoms”	(Brehon et al., 2023)
Symptom Burden & Functional Loss		Sleep disturbance and fatigue	“…I get maybe an hour and a half of REM a night … so I just wake up exhausted … every single day.”	(Brehon et al., 2023)
Symptom Burden & Functional Loss	Diverse and episodic long COVID symptoms intersect with the organization of work and home life	Intersection of symptoms and homelife impact RTW	"The only way I can keep working is by giving up EVERYTHING else in my life. I work, then I lay on the couch or bed and everything I need is brought to me by my spouse. I take the entire weekend to do nothing but sleep/ recover and hope I get enough rest to make it through the next week."	(Stelson et al., 2023)
Symptom Burden & Functional Loss		Sensory sensitivity and environmental overstimulation	“I definitely couldn’t cope being in a room of people coming and going or the phone ringing.” (P4)	(Boutry et al., 2024)`

139

Top-level Theme	Author generated Theme	Author generated Sub-Themes	Quotes	Citation
Symptom Burden & Functional Loss		Vicious cycle of symptoms and emotional burden	"I used to be a happy girl… but I cannot… I've had to reinvent myself… I became more and more introverted."	(Burton et al., 2024)
Symptom Burden & Functional Loss	Physical and psychological burden	Loss of mobility, energy, and mental health	"I was desperate, I just had no quality of life at all. I couldn't speak to my friends for coughing, couldn't look after my family… I was housebound."	(Cooper et al., 2024)
Symptom Burden & Functional Loss	Symptoms	Fatigue, cough, sore throat, muscle aches, diarrhea, loss sense of smell, trouble concentrating, headaches	"Symptoms: Fatigue, cough, sore throat, muscle aches, diarrhea, loss sense of smell, trouble concentrating, headaches." (Participant #14)	(Santiago-Rodriguez et al., 2022)
Symptom Burden & Functional Loss	Persistent symptoms	Trouble concentrating, headaches, loss sense of smell	"Persistent symptoms: Trouble concentrating, headaches, loss sense of smell." (Participant #14)	(Santiago-Rodriguez et al., 2022)
Symptom Burden & Functional Loss	Memory loss and injury	Lost consciousness, fainted, broke nose, head injury	"I decided to work a little bit because I felt like, oh, I have a great energy today. That's how it happened in the mornings. I had amazing energy. But then, an hour into it, or maybe two hours, I was completely done. And I was feeling miserable. So, that day, I tried to work from home. And after an hour, I said, 'No. I am feeling miserable. I'm going to go lay down and	(Santiago-Rodriguez et al., 2022)

140

Top-level Theme	Author generated Theme	Author generated Sub-Themes	Quotes	Citation
			sleep.’ But I woke up... I was drenched in sweat, and my stomach was very upset. And I need to go to the bathroom, and I need to throw up. And I came back to turn on the fan. And I don’t remember seeing anything after that. I might have run back to the bathroom. And then, I remember someone screaming to see if I was okay. So, I ended up fainting because I was very diaphoretic and pale. And my blood pressure apparently tanked. So, I broke my nose and split my head open. And I had to be taken to the hospital.” (Participant #21)	
Symptom Burden & Functional Loss		Limitations due to ongoing symptoms	- "I do some sea angling but I'm a little bit weary of going onto the rock edges because of my footing..."<br>- "I'm pretty much back to my normal self now…"	(Reay et al., 2024)
Symptom Burden & Functional Loss	Imagination Exercise	Cartwheels”:Unfolding vitality and energy<br><br>“Less pain”: Pleasant body sensation	“I felt energized and active. I pictured myself in a sunny stubble field, doing cartwheels and somersaults. It was a delightful experience. I didn’t worry about it not working anymore, but rather enjoyed the image as if I was actually doing it.” (P1)<br>“I just strolled through the day, just as I am, under trees, slightly shady.” (P7)<br><br>“I didn’t think about my main symptoms, which are annoying, and that	(Funk et al., 2025)

141

Top-level Theme	Author generated Theme	Author generated Sub-Themes	Quotes	Citation
		Bubbling kettle”: Concentration difficulties and transfer to everyday life	was a pleasant feeling.” (P3)<br>“The idea of being carefree, of simply living without feeling anything in your body that hurts, just like before, was born.” (P17)<br>“Staying focused is stressful. It feels like a kettle starting to bubble, creating tension instead of relaxation.” (P16)<br>One participant emphasized the importance of guided instruction in dealing with his pain.<br>He believed that the exercise would not be helpful in the long term due to difficulties in per-forming it independently without guidance in his everyday life:<br>“But if I’ll do it [the exercise] on my own, it won’t help me much.” (P11)	
Symptom Burden & Functional Loss	authenticity to symptoms	granted to ICU participants; often lacking for Long Covid participants		(MacLean et al., 2025)
Symptom	Physical and	Chest tightness,	“Oh, okay. The long term effects…I’ll say that the only thing that I	(Santiago-

142

Top-level Theme	Author generated Theme	Author generated Sub-Themes	Quotes	Citation
Burden & Functional Loss	cognitive persistent symptoms	fatigue, cognitive impairment	probably still feel here and there is just this chest tightness that I have sometimes that I feel in the nighttime before I'm going to bed, and it just feels like it's just stuck right here, this breath, and I'm just taking in some more deeper breaths. And then it goes away." (Participant #13) "What worries me now is that I get tired. Yesterday I couldn't run even four blocks … Right now about 30 percent. The other thing that worries me is that COVID has affected my brain in a way that stresses me…" (Participant #19)	Rodriguez et al., 2022)
Symptom Burden & Functional Loss	Uncertainty and unknown prognosis	R ecurring symptoms, unclear recovery	"And what also worries me and is uncomfortable are also the headaches, which do not go away. [medical provider] states 'Well, I don't know'…but…it goes away and then comes back again. And I don't know what I can do." (Participant #23) "For a long time, I was still having stomach cramps, and I was having diarrhea two or three times a day. I have insomnia. I don't know what else… I think I'm trying to be patient, but I think the unknowns kind of make it a little scarier just because why am I still having symptoms two months later on something that the other two people in my family have fully recovered from. This feels weird to me." (Participant #15)	(Santiago-Rodriguez et al., 2022)
Symptom Burden & Functional Loss	Symptoms worsened later in disease course	Symptoms worsened on day 19	"The initial wave was like I was dizzy for a couple weeks and I was kind of body aches. It wasn't that bad but I lost all my taste and smell and I was really out of it, just in the head. Then around day 19 it got way worse. I thought it was just going to suck for a while. I knew I was having these headaches and couldn't think but then it got like my nerves were freaking out. I got really bad ulnar nerve pain and my muscles started twitching and these fingers went numb. Then I started to get a fever around day 20. That	(Santiago-Rodriguez et al., 2022)

143

Top-level Theme	Author generated Theme	Author generated Sub-Themes	Quotes	Citation
			lasted for a week but it wasn't super high grade. It was like 100.2 or something." (Participant #14)	
Symptom Burden & Functional Loss	Neurocognitive Symptoms in the Context of Other Long COVID Symptoms	Fatigue and Symptom Interdependence	"Sometimes I feel as though if I exert myself cognitively then my Long COVID symptoms exacerbate like shortness of breath... if I exert myself physically... I get confused..." (Participant 2)	(Callan et al., 2022)
Symptom Burden & Functional Loss		Ongoing physical and cognitive symptoms	"The brain fog makes it impossible to follow conversations or remember appointments."	(Leggat et al., 2024)
Symptom Burden & Functional Loss	Health Problems		"I am unable to walk for a long distance… I get breathing difficulties" (43 Years/Male)<br>"I have breathing difficulties… 10% difficulties in remembering… I get tense" (57 Years/Male)	(J et al., 2025)
Symptom Burden & Functional Loss		- Affirmation and normalisation of symptoms	"There's nothing that surprises us... that feels good. I'm not being made to be crazy." – P10, 30s, Midwest	(Laestadius et al., 2024)
Symptom	Rollercoaste	- Fluctuating	"The rollercoaster of symptoms has been amazing in these 24 weeks, one	(Ireson et al.,

144

Top-level Theme	Author generated Theme	Author generated Sub-Themes	Quotes	Citation
Burden & Functional Loss	r symptoms	symptoms<br>- Physical and mental toll	week I would have fevers, the next week cough, the next week gastrointestinal symptoms..." (Participant 32)<br>"My life is completely upside down. I can't look back yet on this year since April when I caught COVID19 yet." (Participant 22)	2022)
Symptom Burden & Functional Loss		Cognitive Symptoms	"I just couldn't manage anything. I couldn't add anything together. My brain abandoned me for a long time." (P4R, M)	(Schaap et al., 2022)
Symptom Burden & Functional Loss	Socialising: An Energy Drain While Striving to Regain a Social Life	- Sensory overwhelm<br>- Loss of previous roles<br>- Strained or supportive relationships		(Loft et al., 2022)
Symptom Burden & Functional Loss	Limited Functional Reserve	Patients already functionally impaired; boundaries blurred between COVID-related decline and pre-existing issues	Thisisa70[year-old]Resident,nowpriorpositiveCovid19.…Residentcontinuestobelimitedto extensiveassistwithhis[activitiesofdailyliving]howeverduetogeneralizedweakness[dueto]COVID 19,hetiresveryeasilyanddoesnothaveasmuchmotivationtoleavetheroom.PriortoCOVID, Residentwouldwalkanaverageof400ftusingwalkerwithsupervision.…Heisweaker,especiallyin lowerextremities,whichmayhavecontributedtohismostrecentfall."	(O'Hare et al., 2022)

145

Top-level Theme	Author generated Theme	Author generated Sub-Themes	Quotes	Citation
Symptom Burden & Functional Loss		- Emotional and cognitive fatigue<br>- Need for openness and accommodations	“I’ve tried to tell my colleagues… I really have problems with my memory at the moment.”<br>“I just felt like a total failure.”	(Loft et al., 2022)
Symptom Burden & Functional Loss		difficulty attributing symptoms to COVID-19;	“PatientcomesinwithworseningsymptomsafterrecentCovidaboutamonthago hascompleteda courseofsteroidsforthat.Willtreathimasabronchitiswithsteroidsandbreathing treatmentsgota scantomakesuretherewasnosignsofbloodclotorstructuralabnormalitiesandwi lladmitforfurther care.”	(O'Hare et al., 2022)
Symptom Burden & Functional Loss	Regaining independenc e	Physical recovery efforts and adaptations	- "Now I'm trying to get out myself, and build my strength up..."<br>- "I've had to have a stool, I need a few adaptions..."	(Reay et al., 2024)
Symptom Burden & Functional Loss	Ability to Perceive	Symptom attribution difficulties; denial; communication challenges	“I couldn’t actually quite believe that I might have POTS although I recognised the signs.” (P17)	Gamillscheg et al., 2024)
Symptom Burden & Functional	Returning to Work and Surrenderin	- Premature return to work<br>- Exacerbation of	“I started full time… I could hardly have been any dumber… I thought: Now it’s over.”	(Loft et al., 2022)

146

Top-level Theme	Author generated Theme	Author generated Sub-Themes	Quotes	Citation
Loss	g to the Burden of LTCS	symptoms<br>- Job modifications	“After every meeting or class, I just had to lie in bed for at least an hour.”	
Symptom Burden & Functional Loss	Self-Perception of Work Ability	- Brain fog especially impacts perceived competence<br>- Difficulty with routine tasks<br>- Feelings of “losing it” and reduced confidence	“My IQ scores are high and so people expect a lot of me at work ... and all of a sudden, I just can’t remember things.”	(Chasco et al., 2022)
Symptom Burden & Functional Loss	Symptoms following discharge (impact on ADLs)	Breathlessness	- "I know my lung isn't at full capacity... I cannot walk as fast..."<br>- "I still get quite breathless, I'm on oxygen 24/7 still."	(Reay et al., 2024)
Symptom Burden & Functional Loss	Synergistic Relationship of Fatigue and Brain Fog	- Fatigue and brain fog worsen each other<br>- Cognitive symptoms impair ability to complete tasks even with	“I can handle it if I stay between ... I don’t have to go too far, and I can sit down in any spot.”	(Chasco et al., 2022)

147

Top-level Theme	Author generated Theme	Author generated Sub-Themes	Quotes	Citation
		energy<br>- Strategies include activity rationing		
Symptom Burden & Functional Loss	Difficulty with Driving	- Brain fog, fatigue, anxiety impair driving ability<br>- Driving restricted to safe/familiar conditions<br>- Increases reliance on others	"Do I turn [the car] off? Do I close the garage?"<br>"Now I'm driving myself. But you get anxious, anxiety."	(Chasco et al., 2022)
Symptom Burden & Functional Loss		Fatigue	- "I would get out of bed and get myself washed and come downstairs and within half an hour I would be asleep again..."<br>- "I don't even go shopping anymore..."	(Reay et al., 2024)
Symptom Burden & Functional Loss	Employer Support	- Mixed employment status: some unemployed due to symptoms<br>- Employers mostly supportive initially<br>- Accommodations varied	"I've missed a lot of work ... I don't get paid when I miss work, so it makes it very hard."	(Chasco et al., 2022)
Symptom	Neurocognit	Multidimensional	One of the things I've realised is how many things I do in my normal day...	(Callan et al.,

148

Top-level Theme	Author generated Theme	Author generated Sub-Themes	Quotes	Citation
Burden & Functional Loss	ive Symptoms and Their Natural History	Cognitive Difficulties	that are cognitive... planning, getting there, choosing stuff, all of that is actually really difficult.” (Participant 7)	2022)
Symptom Burden & Functional Loss		Unrecognised evolving symptoms	“Symptoms are changing… that’s why you want to get to be seen.” – Linda (Female, White British, 30s)	(Fang et al., 2024)
Symptom Burden & Functional Loss		Psychological Symptoms	"I’ve got more emotional. [...] I think I’ve cried for the last time 15 or 20 years ago. Now, I think I do so about once a week." (P14NR, M)	(Schaap et al., 2022)
Symptom Burden & Functional Loss	Relapsing-Remitting and Unpredictab le Symptoms	- Symptoms vary daily and unpredictably<br>- Fatigue and brain fog worsen through day<br>- Frustration, anxiety, depression from unpredictability	“I think that you just can’t plan on it because you never know when—I have good days, and I have bad days.”<br>“Every time I think [my sense of taste is] starting to come back and—it goes again.”	(Chasco et al., 2022)
Symptom	Colleagues’	- Symptoms visible	People expect a lot of me ... I just can’t remember things.”	(Chasco et

149

Top-level Theme	Author generated Theme	Author generated Sub-Themes	Quotes	Citation
Burden & Functional Loss	Perceptions	to colleagues<br>- Mental toll from scrutiny<br>- Coping behaviours sometimes obvious		al., 2022)
Symptom Burden & Functional Loss	Feelings of Isolation	- Isolation worsened by symptoms<br>- Guilt and shame for withdrawing socially<br>- Difficult coping with changed dynamics	"I'm limited in who I'm willing to let come over and see me in this state."	(Chasco et al., 2022)
Symptom Burden & Functional Loss	Loss/Change of Roles	- Symptoms impair fulfilling family roles<br>- Feelings of inadequacy and isolation within household	"A good day was one where I can recognize I'm getting tired ... I can be part of the family with minimal pain."	(Chasco et al., 2022)
Symptom Burden & Functional Loss	Naming the Phenomenon	Varied Perceptions of 'Brain Fog'	"Does anyone ever refer to it as neurocognitive fatigue? In a way I don't like brain fog as it's too vague... Though I don't think neurocognitive fatigue encompass the word finding difficulties..." (Participant 10)	(Callan et al., 2022)

150

Top-level Theme	Author generated Theme	Author generated Sub-Themes	Quotes	Citation
Information, Knowledge & Health Literacy			The key is really to share with other people and to socialize and to not isolate one's self." (No pre-MH)	(Kennelly et al., 2023)
Information, Knowledge & Health Literacy	Self-help	- Supplements and self-care efforts	"I am taking multivitamins, folic acid, B-12, cod liver oil, Vit D, CoQ10 and turmeric religiously in an attempt to try and help myself." (Participant 61)<br>"Every day I have tried to be positive and think I can outsmart or positive think my way out of this, however despite doing everything I can...these symptoms persist." (Participant 17)	(Ireson et al., 2022)
Information, Knowledge & Health Literacy			"Even though I am anxious and I do feel angry all the time... I have very supportive friends and very supportive family, and like I said, my husband's amazing... and the gratitude that I feel." (No pre-MH)	(Kennelly et al., 2023)
Information, Knowledge & Health Literacy		Gradual Improvement	"It was a real slow process of feeling better... I could walk maybe 200 metres and then that was it." (N30, F, 30–49)<br>"The palpitations, are less prominent now, but I still get palpitations most days." (N6, F, 30–49, HCW)<br>"After COVID for the first two or three months my sugar was everywhere…" (N38, M, 50+)	(Hitch et al., 2023)
Information, Knowledge & Health			"I'm on the long COVID support groups on Facebook but it's kind of all over the map... it's not a regulated source of information." (No pre-MH)	(Kennelly et al., 2023)

151

Top-level Theme	Author generated Theme	Author generated Sub-Themes	Quotes	Citation
Literacy				
Information, Knowledge & Health Literacy		Emotional Responses to Invisible Disability	"I found myself restating and reiterating many times professionally where I'm at now in terms of cognitive ability..." (Participant 9)	(Callan et al., 2022)
Information, Knowledge & Health Literacy	Invisibility	- Feeling invisible<br>- No biomedical confirmation	"I am waiting on an antibody test and respiratory tests. Consultant said I am probably suffering from reconditioning through lockdown. This affected me badly. Nobody is listening. I feel debilitated and so depressed." (Participant 24)<br>"I had a test at 9 weeks and a test at 14 weeks, both negative but when I would have been positive, they weren't offering tests!" (Participant 9)	(Ireson et al., 2022)
Information, Knowledge & Health Literacy		Administrative Burden and Constant COVID Reminders	"You are dealing with… a lot, talking about COVID a lot." (N41, M, 30–49, HCW)	(Hitch et al., 2023)
Information, Knowledge & Health Literacy		Broader Social Disruption and Lockdowns	The biggest trauma's been... the lockdowns and the emotional stress…" (N39, M, 50+)<br>"You couldn't even go for a walk around the block." (N29, M, 50+)	(Hitch et al., 2023)
Information, Knowledge & Health Literacy		Mental Health Strain from Returning to Healthcare Settings	It's very triggering to come back into that environment…" (N30, F, 30–49, HCW)	(Hitch et al., 2023)

152

Top-level Theme	Author generated Theme	Author generated Sub-Themes	Quotes	Citation
Information, Knowledge & Health Literacy		Social support and long COVID knowledge sharing	I literally probably wouldn't be alive if it wasn't for the support groups and the sharing of information and support..." (Pre-MH)	(Kennelly et al., 2023)
Information, Knowledge & Health Literacy	Uncertainty and fear with mental health impact	- Mental health burden<br>- Fear and dependence	"This is now of course affecting my mental health and I feel low in mood at times, and struggling with living alone but also hate requiring other people's help. I am often too tired to communicate anyway. I hate being dependent." (Participant 7)	(Ireson et al., 2022)
Information, Knowledge & Health Literacy	Difficulty with Multitasking	- Struggles with focus, multitasking<br>- Reduced efficiency, productivity<br>- Need more time or fewer tasks	"I cannot multitask as well ... I'm doing a bunch of things okay. I'm not doing a bunch of things great."	(Chasco et al., 2022)
Information, Knowledge & Health Literacy		Communication and support from colleagues	"They are silently looking out for me… it is really good."	(Gyllensten et al., 2023)
Information, Knowledge & Health		Long-term need for adjustment	"They promised me that I should be able to take it easy… but a week went by and then the work was building up more and more…"	(Gyllensten et al., 2023)

153

Top-level Theme	Author generated Theme	Author generated Sub-Themes	Quotes	Citation
Literacy				
Information, Knowledge & Health Literacy	End of normality	- Realization of lasting impact<br>- Loss of previous health & function	"I tell people it is like living a half-life. I can function but can't do what I used to. If I go out for a walk it means I have no energy for anything else." (Participant 59)<br>"Other than the mild asthma I was in great health before April 11th [2020]. I have run and completed 20 full marathons. I flew jets for a living. I'm not a wimp. But now I feel like I couldn't fight my way out of a wet paper bag." (Participant 31)	(Ireson et al., 2022)
Information, Knowledge & Health Literacy		- Carefully managed social life<br>- Guilt over withdrawal	"I do not have any extra battery…I only have one and that…that one needs to be charged at home."<br>"It really does affect how I am social…Then the next day I have a terrible headache.	(Loft et al., 2022)
Information, Knowledge & Health Literacy		Uncertainty and Pre-existing Conditions	"I've got mesothelioma... shortness of breath is a daily life thing for me." (N9, M, 50+)<br>"You will be talking and then… I'm 62… is this a sign of something else?" (N40, F, 50+, HCW)	(Hitch et al., 2023)
Information, Knowledge & Health Literacy			"I wanted to go back to smoking... I ate and gained 30 pounds... that actually besides the anger probably impacted my mental health the most..." (Pre-MH)	(Kennelly et al., 2023)
Information, Knowledge & Health			"It has completely ruined my life and I'm 10 seconds away from suicide at any time. I don't have any money anymore, I don't have friends anymore, and I am always in pain." (Pre-MH)	(Kennelly et al., 2023)

154

Top-level Theme	Author generated Theme	Author generated Sub-Themes	Quotes	Citation
Literacy				
Information, Knowledge & Health Literacy			"I do sleep meditations every night before I go to bed and that really helps..." (Pre-MH)	(Kennelly et al., 2023)
Information, Knowledge & Health Literacy			This long COVID, for myself... if things get worse and worse and worse, I would opt for medical assistance in dying." (No pre-MH)	(Kennelly et al., 2023)
Information, Knowledge & Health Literacy	Theme 1: The emotional landscape of long COVID	Anxiety and fear of the unknown	"The anxiety that came because of [long COVID]—I don't know what it does or how it trig-<br>gers your brain, if it does it at all, but I believe that it did something that created anxiety."<br>(No pre-MH)	(Kennelly et al., 2023)
Information, Knowledge & Health Literacy		Motivation to regain function and PA	"I've been a runner and then a cyclist for many years... got back in the cycling a little bit." (IV6)<br>"I've always been physically active... somewhat testing myself... hoping that I can get back to playing basketball soon..." (IV15)	(Humphreys et al., 2021)
Information, Knowledge & Health Literacy			"I can't cope with multiple inputs... I was able to multitask... now I can't keep multiple plates spinning..." (Participant 5)	(Callan et al., 2022)
Information,			"I could understand there is a vehicle in front of me or if the light is green	(Kennelly et

Top-level Theme	Author generated Theme	Author generated Sub-Themes	Quotes	Citation
Knowledge & Health Literacy			or if the light is red... but I may not understand what that means..." (Pre-MH)	al., 2023)
Information, Knowledge & Health Literacy		Exhaustion as a barrier to completing daily tasks	"Physically, I can't do a whole lot at once... I have to bunch it up and alternate days. Otherwise I'm toasted." (Pre-MH)	(Kennelly et al., 2023)
Information, Knowledge & Health Literacy	Treatment indications	Secondary infections, asthma exacerbation	"Antibiotics were prescribed for... confirmed pneumonia."	(Khashei et al., 2023)
Information, Knowledge & Health Literacy			"Emailing with friends—just social emailing. It takes me weeks and sometimes even months to get back to somebody because I just don't want to deal with it..." (No pre-MH)	(Kennelly et al., 2023)
Information, Knowledge & Health Literacy			"I'm someone who's very productive... it was like falling off a cliff." (Pre-MH)	(Kennelly et al., 2023)
Information, Knowledge & Health Literacy			"I tried to go back to work for a while... but it just didn't get better... It's just not worth it." (No pre-MH)	(Kennelly et al., 2023)

156

Top-level Theme	Author generated Theme	Author generated Sub-Themes	Quotes	Citation
Information, Knowledge & Health Literacy			"I can ask somebody a question and then I'll ask the exact same question 2 min after and not remember I've asked them..." (Participant 10)	(Callan et al., 2022)
Information, Knowledge & Health Literacy			"Plus losing quite a few friends over this entire situation... people just eventually kind of give up on it asking whether or not you're coming out..." (Pre-MH)	(Kennelly et al., 2023)
Information, Knowledge & Health Literacy			It has really impacted how I deal with family and home... I am kind of like that vegetable [in] the corner and it feels like life is passing me by..." (Pre-MH)	(Kennelly et al., 2023)
Information, Knowledge & Health Literacy	Societal Awareness	- Existing Support Networks<br>- Advocacy	"My daughter would go (mimicking voice) alright? And I'd go nope! And I'd just start crying." [Claire]<br>"When it comes from people who have gone through the same thing, it feels a little bit more as in it's realistic." [Jessica]<br>"I think the groups bringing it to light… means that someone's gonna have to step up and do something." [Chloe]<br>"The COVID clinics were completely underfunded before they even start." [James]	(Day, 2022)
Information, Knowledge & Health			"I would say the only stigma I have experienced is that I have already had a mental illness before that and that they don't take me seriously enough..." (Pre-MH)	(Kennelly et al., 2023)

157

Top-level Theme	Author generated Theme	Author generated Sub-Themes	Quotes	Citation
Literacy				
Information, Knowledge & Health Literacy		Individual strategies	"Now I have to write down everything exactly, and I prefer mail to phone calls…"	(Gyllensten et al., 2023)
Information, Knowledge & Health Literacy	Types of tests and reporting	Positive tests, antibody tests, repeated tests	"Just got tested today because I've lost all smell and taste."; "My job had the whole building tested...	(Khashei et al., 2023)
Information, Knowledge & Health Literacy			"One is from friends, in a way it is kind of a stigma... 'Oh, you should be better by now...'" (Pre-MH)	(Kennelly et al., 2023)
Information, Knowledge & Health Literacy			"It's difficult to comprehend and take in written information... I just felt, 'I can't do this at the moment'..." (Participant 8)	(Callan et al., 2022)
Information, Knowledge & Health Literacy		Fluctuation and Gradual Improvement		(Callan et al., 2022)
Information, Knowledge &		The burden of blame	"I think that people kind of wanna say 'well, you got COVID, so you have to deal with the repercussions of it,' as if I didn't do everything to avoid in	(Kennelly et al., 2023)

158

Top-level Theme	Author generated Theme	Author generated Sub-Themes	Quotes	Citation
Health Literacy			the first place." (No pre-MH)	
Information, Knowledge & Health Literacy			"I'm angry and that's not gonna change for a very long time because there's no one to blame, which means it's my fault." (Pre-MH)	(Kennelly et al., 2023)
Information, Knowledge & Health Literacy			"For me, the challenges are largely invisible... I don't like second guessing myself." (Pre-MH)	(Kennelly et al., 2023)
Information, Knowledge & Health Literacy			"For me, going out for walks and playing some sports to relieve the stress and anxiety." (No pre-MH)	(Kennelly et al., 2023)
Information, Knowledge & Health Literacy		Challenges with emotional regulation	"For me, it's the emotional sensitivity. I get angry at the drop of a hat, sad at the drop of a hat. I mean, there's a million commercials I can't watch." (Pre-MH)	(Kennelly et al., 2023)
Information, Knowledge & Health Literacy		Desire for better tailored guidance	"Everything that you read is pace yourself... but that's really hard to do... learning by default... what's enough for me might not be enough for somebody else." (IV8)<br>"With chronic fatigue syndrome... no two days are alike so it's really hard to find a baseline because it's so erratic." (IV3)	(Humphreys et al., 2021)

Top-level Theme	Author generated Theme	Author generated Sub-Themes	Quotes	Citation
Information, Knowledge & Health Literacy	Exercise	- Uncertainty balancing exercise<br>- Fear of relapse	"I'm still struggling to have the confidence to push myself to make gains with my physical fitness, as I still don't fully understand the balance between what might be a trigger..." (Participant 1)<br>"I have had to abstain from caffeine, alcohol and my usual exercise (running and cycling) for the duration of my illness so far." (Participant 60)	(Ireson et al., 2022)
Information, Knowledge & Health Literacy		Need to be believed and validated	"It didn't seem connected... one of the best things... was just hearing that the symptoms I have were very common." [P4102]	(Duncan et al., 2023)
Information, Knowledge & Health Literacy		In society	"Because right now, it is a cultural disease… I feel very vulnerable."	(Gyllensten et al., 2023)
Information, Knowledge & Health Literacy				(Schaap et al., 2022)
Information, Knowledge & Health Literacy			"Symptoms persisting regardless of attempts to find treatments." "Minor relief… for others, no treatment(s) had yet offered a reprieve."	(Wurz et al., 2022)
Information,	Physical		"Fears of engaging in more physical activity in case it worsened their	(Wurz et al.,

160

Top-level Theme	Author generated Theme	Author generated Sub-Themes	Quotes	Citation
Knowledge & Health Literacy	activity is difficult, and in some cases, not possible		symptoms.” “Deteriorating trust in their own bodies.” “New movement routine… stood in stark contrast to their prior capacity.”	2022)
Information, Knowledge & Health Literacy		Modified sense of self	“Feeling trapped or stuck within a disabled body… frustrating and scary.” “A deep sense of loss over their changed health and functional capacity.”	(Wurz et al., 2022)
Information, Knowledge & Health Literacy	Prolonged hospitalizations and unconsciousness	Unconscious for over a week, intubation	“I was [at the hospital] - I believe I was from March 27 or 28 to April 30. One month. When I woke up [after being unconscious], I was in intensive care. I was wearing a respirator in my mouth and I was on tubes - no, I no longer had tubes in my mouth. I had like a respirator in my nose that helped me with oxygen. I didn't know what had happened to me. And a nurse told me, ‘you tested positive for Covid’ - I was already a week and a half with the tubes.” (Participant #22)	(Santiago-Rodriguez et al., 2022)
Information, Knowledge & Health Literacy	Intubation and hallucinations	Semi-conscious, hallucinations, communication issues	“There were different phases. Initially in the ER, but later that night I don’t even think I remember having a choice about intubation. I was really anxious and nervous about getting intubated. All of a sudden, they came in, I’m getting intubated. I don't remember even saying, no, I don’t want to do that; right? I just kind of accepted it without fighting it or questioning it. Initially, there were periods when I was intubated and the medicine would wear off, but I was semi-conscious and I remember having these hallucinations. They were really funky dreams. And it was really hard to	(Santiago-Rodriguez et al., 2022)

161

Top-level Theme	Author generated Theme	Author generated Sub-Themes	Quotes	Citation
			understand everything. I was trying – at one point I was still intubated, I wanted to try to get them to call [partner's name] and I couldn't write across. I kept writing the letters over each other so they couldn't understand what the hell I was talking about." (Participant #1)	
Information, Knowledge & Health Literacy			"I think it's wise to just stay positive [...] every step forwards, however small, is something positive." (P1R, M)	(Schaap et al., 2022)
Information, Knowledge & Health Literacy	Effect on Parenting	- Difficulty being present and active parent<br>- Reliance on older children<br>- Guilt over inability to engage	"I try to interact with her, but I don't have the energy to do anything ... it feels like a waste."	(Chasco et al., 2022)
Information, Knowledge & Health Literacy	Fear and uncertainty about disease course	Fear of unknown, concern about progression	"I knew that I had it probably. I guess, I don't know, it's just like, 'Okay.' At that time, I would have been a lot scared now because we know how crazy it can be and then people fall off a cliff. We didn't know any of that back then, so I was like, 'You know, as long as I'm not dying then I guess I'm not dying'…I was worried because I had done enough research to understand that the receptors where your taste, where your smell, are very close to the brain and the central nervous system so it's like, 'Oh God, what if it gets into my brain. We don't know anything about this.'" (Participant #14)	(Santiago-Rodriguez et al., 2022)

162

Top-level Theme	Author generated Theme	Author generated Sub-Themes	Quotes	Citation
Information, Knowledge & Health Literacy		Slow and Steady	‘I’m alright with endurance things, so I can run slow and steady, that’s fine...’ (F06; 10 months post-infection)<br>‘...I don’t have the strength back that I used to have...’ (M13; 11 months post-infection)<br>‘If I run upstairs really quickly by the time I’ve got to the top of the stairs I have to sit down and I have to recover.’ (F08; 10 months post-infection)<br>‘bizarrely, my swimming has been great this year...’ (F03; 7 months post-infection)	(Shelley et al., 2021)
Information, Knowledge & Health Literacy	Medical support and reassurance	Doctor contact, encouragement	“My primary doctor, she is an infectious disease specialist, only saw me on a video call and told me: ‘If you don’t feel well go to the E.R.’ I didn’t call her, she called me. And she gave me hope: ‘I have patients who have survived having HIV. HIV has nothing to do with Covid. You are not going to die’ and all that.” (Participant #19)	(Santiago-Rodriguez et al., 2022)
Information, Knowledge & Health Literacy		Timing and proportionality of care	“Had I had some … background knowledge [earlier]… maybe my recovery would have been quicker”	(Brehon et al., 2023)
Information, Knowledge & Health Literacy		Psychological Impacts	‘With the germs [in gyms] today, you’ve got to be very cautious’ (M02; 6 months post-infection)<br>‘I stopped cycling, even though everybody seemed to take it up, because I was struggling with my breath...’ (M07; 10 months post-infection)<br>‘...my mood was dipping, so severe, severe depression’ (F01; 7 months post-infection)<br>‘So, it’s almost like everything’s gone on a go slow... I feel like a little old	(Shelley et al., 2021)

163

Top-level Theme	Author generated Theme	Author generated Sub-Themes	Quotes	Citation
			lady instead of the active mum that I was...' (F08; 10 months post-infection)	
Information, Knowledge & Health Literacy		Relevance of interventions	"I think the … key … is learning how to pace … but like actually being taught what it is... would have potentially made a huge difference."	(Brehon et al., 2023)
Information, Knowledge & Health Literacy	Psychological distress, fear of dying	Depression, isolation, fear of dying alone	When I was sick I had the news on a lot, mostly just CNN. I had been isolating before. I mean, I didn't do a great job at the sheltering in place. But when I had to self-isolate because I was ill, I had to shut myself from anybody. Any food I had [was] delivered and, so, I had no reasons to go outside. So, I started to get depressed. And this is going to sound silly because you'd have to be in my head to understand, but I was really feeling sad…everything was compounding, like just with the news…I kept thinking I'm going to die. No one's seen me, nobody cares that they can't see me. So, I called…911 – well, there was the non-emergency number, but they did come and then they took me to the emergency [room]. And it was funny because one of the EMTs, whatever, he asked me what's wrong. I said, 'well, I really feel sad.' He said: 'Okay. We're going to take you in for sadness?' I said: 'Well, it sounds funny because the way you're saying it, but yeah, really I am'" (Participant #5)	(Santiago-Rodriguez et al., 2022)
Information, Knowledge & Health Literacy		Multidisciplinary and flexible delivery	"There's a definite utility to virtual care... but... there's this disconnect"	(Brehon et al., 2023)

164

Top-level Theme	Author generated Theme	Author generated Sub-Themes	Quotes	Citation
Information, Knowledge & Health Literacy		Individual Strategies	"Don't avoid physical activities, but be smart in what you do and don't do. [...] Push up against the limits." (P16NR, M)	(Schaap et al., 2022)
Information, Knowledge & Health Literacy			"I found it very difficult to ask acquaintances for help. [...] Otherwise, you don't get any food." (P24NR, M)	(Schaap et al., 2022)
Information, Knowledge & Health Literacy			"I now have memory problems. [...] I often just cannot remember the name of something." (P8NR, F)	(Schaap et al., 2022)
Information, Knowledge & Health Literacy			"Certain flavours and smells are wrong. [...] Coffee beans don't smell right." (P12R, M)	(Schaap et al., 2022)
Information, Knowledge & Health Literacy			"It's like a tightness in the upper airways, like a brick laying on your chest." (P20NR, F)	(Schaap et al., 2022)
Information, Knowledge & Health	Impact of COVID-19	Consequences	"Everywhere, I meet my own limits." (P22NR, M)	(Schaap et al., 2022)

165

Top-level Theme	Author generated Theme	Author generated Sub-Themes	Quotes	Citation
Literacy				
Information, Knowledge & Health Literacy		Claiming normalcy with hesitation	"Everything is back to normal… sometimes I have a fit of irritation…" (A013, female, 80)	(Schiavi et al., 2022)
Information, Knowledge & Health Literacy		Anxiety, paranoia, panic	"This thing caused me anxiety and panic attacks" (A033, male, 52)	(Schiavi et al., 2022)
Information, Knowledge & Health Literacy		Quarantine-related personal isolation	"...I had to do 14 more days of isolation" (A013, female, 80)	(Schiavi et al., 2022)
Information, Knowledge & Health Literacy			"Being mad at yourself, and disappointed, and having very high expectations of yourself." (P5NR, F)	(Schaap et al., 2022)
Information, Knowledge & Health Literacy			"It's an entirely different disease. [...] Everything is too much! And thinking, walking, talking, picking up something." (P20NR, F)	(Schaap et al., 2022)
Information, Knowledge &	Emotional distress	Depression and disinterest	"There's this sort of depression… a lack of interest in anything, from morning to night" (A060, male, 73)	(Schiavi et al., 2022)

166

Top-level Theme	Author generated Theme	Author generated Sub-Themes	Quotes	Citation
Health Literacy				
Information, Knowledge & Health Literacy		Cause	"The fatigue, well, is that because of the fibrosis or because of COVID?" (P19NR, F)	(Schaap et al., 2022)
Information, Knowledge & Health Literacy		Avoiding naming the illness	"This thing … the more time passes, the more I realize …" (A044); "I always get chills … goose bumps" (A029)	(Schiavi et al., 2022)
Information, Knowledge & Health Literacy	Pre-COVID-19 Physical Activity		'Prior to getting COVID, I would have put myself in a fit category' (F01; 7 months post-infection)<br>'I couldn't live without it, yeah…I guess it's the thing that gives me joy in life; it's an inseparable part of me' (F14; 10 months post-infection)<br>'I have a medical condition anyway, which does cause some fatigue…I do think that having had the pre-existing health condition has helped me quite a bit because my ability to be patient and just try and accept things as they are and do what I can.' (F09; 10 months post-infection)	(Shelley et al., 2021)
Information, Knowledge & Health Literacy		Timeline	"[Recovery goes] very slow, very, very slow. [...] Then suddenly a day or two, three, it doesn't work." (P17NR, F)	(Schaap et al., 2022)
Information,			"It's just like I've become an old man. Yes, I am old, but in the months of	(Schaap et al.,

167

Top-level Theme	Author generated Theme	Author generated Sub-Themes	Quotes	Citation
Knowledge & Health Literacy			COVID, I've deteriorated by years." (P24NR, M)	2022)
Information, Knowledge & Health Literacy		Patients declining or overwhelmed by repeated assessments, monitoring or follow-up contacts		(O'Hare et al., 2022)
Information, Knowledge & Health Literacy		Clarity in care and education	"She sent me so much stuff by email I didn't know if I was coming or going"	(Brehon et al., 2023)
Information, Knowledge & Health Literacy		Strained relationships and social isolation	It impacts … every single facet of your relationships...your ability to … be a father, ability to be a supportive partner and husband"	(Brehon et al., 2023)
Information, Knowledge & Health Literacy			"Me and my husband have got a traffic light system now... green's fine, amber is simple talk, red is just stop... that really has helped us..." (Participant 7)	(Callan et al., 2022)
Information, Knowledge &		Fear and caution around physical	My heart rate being high is a worry... doctor said... don't push it... which again doesn't help me an awful lot." (IV1)	(Humphreys et al., 2021)

168

Top-level Theme	Author generated Theme	Author generated Sub-Themes	Quotes	Citation
Health Literacy		activity		
Information, Knowledge & Health Literacy		Lack of medical support	"Having the right staff, having medical support in the team, that's been a big gap..." [S4101]	(Duncan et al., 2023)
Information, Knowledge & Health Literacy		Inexperienced staff under pressure	"Hardest thing—so many different symptoms that you can have with Long COVID." [S2204]	(Duncan et al., 2023)
Information, Knowledge & Health Literacy		Staffing shortages	"We can't staff wards properly... How will we staff a Long COVID service?" [S1202]	(Duncan et al., 2023)
Information, Knowledge & Health Literacy		complex interactions with other health conditions, behaviours, and stressors		(O'Hare et al., 2022)
Information, Knowledge & Health		PTSD-like stress response triggered by body	"When [I] first started to get sick, [I] became very weak and had to hold the walls when [I] showered. [I no] longer have weakness, [but] now experience anxiety [and] possibly panic attacks when [I] shower [Patient	(Duan et al., 2023)

169

Top-level Theme	Author generated Theme	Author generated Sub-Themes	Quotes	Citation
Literacy			ID 2].”	
Information, Knowledge & Health Literacy		Staff learning and professional development	"England has been incredibly helpful in giving and sharing lots..." [L3203]	(Duncan et al., 2023)
Information, Knowledge & Health Literacy		Positive patient experiences	"...it wasn't all in my head... it was just such a relief to speak to somebody who was just genuinely interested..." [P4107]	(Duncan et al., 2023)
Information, Knowledge & Health Literacy		Lack of best practice guidance	"...graded exercise therapy is really harmful, and you absolutely should not prescribe it." [S2104]	(Duncan et al., 2023)
Information, Knowledge & Health Literacy		Value of shared experiences	"...a lot of my symptoms are replicated... I’m getting a lot of assurance..." [P3208]	(Duncan et al., 2023)
Information, Knowledge & Health Literacy		Discomfort with labelling patients	It was really difficult actually to know whether to adopt the term Long COVID..." [S4101]	(Duncan et al., 2023)
Information, Knowledge &			“People have said ‘you’re going round in circles in your conversation’... can’t seem to cut me any slack... it’s beyond my comprehension...”	(Callan et al., 2022)

170

Top-level Theme	Author generated Theme	Author generated Sub-Themes	Quotes	Citation
Health Literacy			(Participant 5)	
Information, Knowledge & Health Literacy		Demand exceeding capacity	They [HB4 Long COVID service] were basically inundated... I had to wait about four months..." [P4101]	(Duncan et al., 2023)
Information, Knowledge & Health Literacy		MDT communication and collaboration	"...just basically building a care package for me... they were quite good actually talking to each other as well..." [P4105]	(Duncan et al., 2023)
Information, Knowledge & Health Literacy		Mental health impacts	"I'm sad, … I'm angry, I'm frustrated … but I'm not depressed...psych[ological] support to help people [is] critical."	(Brehon et al., 2023)
Information, Knowledge & Health Literacy		Uncertainty in everyday activities	"I'm not the same as before. My legs get tired. I cannot walk the same as before. I do not know if they will ever be the same." / "I felt completely depleted of my ability to do day-to-day things [Patient ID 20]."	(Duan et al., 2023)
Information, Knowledge & Health Literacy		Increased reliance on others	"I'm a very happy and positive person, but a lot of things have stopped: I cannot clean the house, I cannot go outside and garden… [Patient ID 96]."	(Duan et al., 2023)
Information,	Emotion of	Fear for One's Own	ID7: "the greatest fear was when I got into the ambulance and I thought to	(Piras et al.,

Top-level Theme	Author generated Theme	Author generated Sub-Themes	Quotes	Citation
Knowledge & Health Literacy	Fear	Life	my daughter who greeted me from the window saying ‘Hi dad, I love you’. It was the worst moment, the fear for not coming back home.”	2022)
Information, Knowledge & Health Literacy		Loss of function and self	“A shell of the person [they] used to be”; “paranoia over not knowing [their] own body”	(Brehon et al., 2023)
Information, Knowledge & Health Literacy			ID14: “I remember the fear given by the breathing difficulty, fear and panic... and in the end, they [doctors] intubated me (...). After waking up it seemed that the worst was over, but for me, it was not really like this, I always continued to have anxiety and fear to die.”	(Piras et al., 2022)
Information, Knowledge & Health Literacy				(Duan et al., 2023)
Information, Knowledge & Health Literacy		e) COVID-19 as a turning point for positive change	“[My] health is better now than before being hospitalized… [Patient ID 82].” / “It brought my family together and a community of friends… [Patient ID 137].”	(Duan et al., 2023)
Information, Knowledge & Health Literacy		Fear about Getting Sick Again and Risk of Contagion	ID4: “one of the biggest fears that never left me was the fear of having transmitted the infection to someone at home.”	(Piras et al., 2022)

172

Top-level Theme	Author generated Theme	Author generated Sub-Themes	Quotes	Citation
Information, Knowledge & Health Literacy		Frustration over loss of independence	"[I]t has felt very confining being stuck at home [due to my shortness of breath] [Patient ID 95]." / "I'm a very happy and positive person, but a lot of things have stopped: I cannot do anything that I used to… [Patient ID 96]."	(Duan et al., 2023)
Information, Knowledge & Health Literacy		d) Change in outlook	"This experience has brought to me more positivity… it's really brought a remarkable change in my life… [Patient ID 68]."	(Duan et al., 2023)
Information, Knowledge & Health Literacy		Challenges with information comprehension	"...the common person can't do that [critically appraise medical journals]." (IV12)	(Humphreys et al., 2021)
Information, Knowledge & Health Literacy	Isolation and Loneliness	Hospital Isolation from Loved Ones	ID6: "There were a few moments where I felt anxious. For example, the lack of contact with people, we were many [hospitalized patients] but perhaps the isolation [from loved ones] made us feel even more alone."	(Piras et al., 2022)
Information, Knowledge & Health Literacy		Fear of transmitting to others	"…the [doctors] said I don't need to be quarantined… this is untrue because my two kids… perhaps I caused that [Patient ID 3]."	(Duan et al., 2023)
Information, Knowledge & Health		Loneliness as Emotional Distress	ID11: "I was alone, I had no company. I had no one, loneliness was the real stress. I wished someone who could stay with me longer, but I understood that it was not possible, I suffered from not having someone to	(Piras et al., 2022)

173

Top-level Theme	Author generated Theme	Author generated Sub-Themes	Quotes	Citation
Literacy			chat with, I missed people, the contact, chatting...”	
Information, Knowledge & Health Literacy		b) Emotional impacts	“I can’t listen when they talk on the news about it, then I get anxiety… I have to work through it… I’m speaking with a therapist… [Patient ID 36].”	(Duan et al., 2023)
Information, Knowledge & Health Literacy	impact on Quality of Life	(a) Physical complications	“It completely changed my life. I am not the same person as I was before. [Patient ID 90].” / “Most of the patients that are recovering should really ingrain in them to have patience. [Patient ID 43].”	(Duan et al., 2023)
Information, Knowledge & Health Literacy		c) Healing process	“[My] shortness of breath makes me nervous… trying breathing and physical activities to improve [Patient ID 140].” / “I walk and exercise… look at nutrition labels… [Patient ID 100].”	(Duan et al., 2023)
Information, Knowledge & Health Literacy	Effect on Partners	- Partners supportive but stressed<br>- Shared routines disrupted<br>- Partner burnout common	My partner has developed anxiety and had to go on medication ... taken on a lot of responsibility.”	(Chasco et al., 2022)
Information, Knowledge & Health	Mixed messaging from health	Confusion over health advice	“They just keep them (at home in isolation) for 5 days… they come back and they’re still coughing and have fever… that’s what they are saying now…”	(Messiah et al., 2023)

174

Top-level Theme	Author generated Theme	Author generated Sub-Themes	Quotes	Citation
Literacy	information sources			
Information, Knowledge & Health Literacy		Exercise intolerance	“The physios want me to continue the exercises at home but that is impossible for me to do.”	(Al-Jabr et al., 2024)
Information, Knowledge & Health Literacy	Difficulty obtaining diagnosis due to lack of clinical knowledge		“The GP... said it is probably [Long Covid], but there's no research on it.”	(Faux-Nightingale et al., 2025)
Information, Knowledge & Health Literacy	Diagnosis provides clarity and reduces emotional distress		“I don't even know how long this is going to be.”	(Faux-Nightingale et al., 2025)
Information, Knowledge & Health Literacy	Diagnosis validates children and young people CYP and		“Sometimes it would be nice—it would have been nice for somebody to go, ‘okay yeah we do believe you.’”	(Faux-Nightingale et al., 2025)

175

Top-level Theme	Author generated Theme	Author generated Sub-Themes	Quotes	Citation
	family experiences			
Information, Knowledge & Health Literacy	The importance of receiving a diagnosis		"That was like a huge thing seeing it on telly… not only the thing that it was 'Oh, that is what I have'…"	(Faux-Nightingale et al., 2025)
Information, Knowledge & Health Literacy		Guilt and desire for normality	"It was about trying to get some normality back and... live a bit more of a normal life." (P5)	(Boutry et al., 2024)`
Information, Knowledge & Health Literacy		Impact of LC	"Before covid, I've sort always been the one to look after everyone and the calming, reasonable, logical person, sort everything out… however now, there are so many things it has impacted—they all make each other worse."	(Al-Jabr et al., 2024)
Information, Knowledge & Health Literacy				(Funk et al., 2025)
Information, Knowledge & Health Literacy		Helpful supports	"…online LC-peer groups provided a useful source of information and support…"	(Al-Jabr et al., 2024)
Information,		Mental health	"…I do… on Netflix they have the headspace guided meditations and	(Al-Jabr et

176

Top-level Theme	Author generated Theme	Author generated Sub-Themes	Quotes	Citation
Knowledge & Health Literacy		strategies	stuff… I had to readapt on how to relax and calm myself.”	al., 2024)
Information, Knowledge & Health Literacy		Difficulties and suggestions for improving the LC-OHP	“…the earlier that people could get it the more benefit they would get from it.”	(Al-Jabr et al., 2024)
Information, Knowledge & Health Literacy			“I lost many customers… So, I lost my business.” (Bakery business)	J et al., 2025)
Information, Knowledge & Health Literacy		Awareness and availability of support	“…neurology appointment came away disappointed and angry at lack of understanding…”	(Al-Jabr et al., 2024)
Information, Knowledge & Health Literacy		Self-treatment strategies	“Still feel antihistamines and low histamine diet do help.”	(Al-Jabr et al., 2024)
Information, Knowledge & Health Literacy		Changes in functional ability	“…writing Christmas cards out of my mind. Completed them but it took three hours…”	(Al-Jabr et al., 2024)

177

Top-level Theme	Author generated Theme	Author generated Sub-Themes	Quotes	Citation
Information, Knowledge & Health Literacy	illness experiences are legitimate	importance of making people feel their illness experiences are legitimate regardless of diagnosis/status		(MacLean et al., 2025)
Information, Knowledge & Health Literacy		Comparing with others	"Sitting on the side-lines watching everyone else live."	(Al-Jabr et al., 2024)
Information, Knowledge & Health Literacy		Loss of former self	"I just know I'm not me anymore."	(Al-Jabr et al., 2024)
Information, Knowledge & Health Literacy	legitimate patient status	conferred by hospitalisation and especially ICU admission		(MacLean et al., 2025)
Information, Knowledge & Health Literacy		Seasonal/environmental influence	"Housebound [can't breathe cold air—Hurts] due to cold. Muscle and bones hurting a lot."	(Al-Jabr et al., 2024)
Information,			"Attempting to return to work. So far two days is the longest I've been	(Stelson et

178

Top-level Theme	Author generated Theme	Author generated Sub-Themes	Quotes	Citation
Knowledge & Health Literacy			able to work without a full relapse of symptoms."	al., 2023)
Information, Knowledge & Health Literacy			"Having what I consider to be long COVID is destroying my life… I have worked over the years to build a successful business, which I am finding hard to maintain."	(Stelson et al., 2023)
Information, Knowledge & Health Literacy			"Half a day of work in office causes 3–5 days in bed. The same work from home is O.K."	(Stelson et al., 2023)
Information, Knowledge & Health Literacy			If I took another sick day, I was going to lose my job. So I went to work with chest pain, fever, the lot."	(Stelson et al., 2023)
Information, Knowledge & Health Literacy			"Attempting to return to work. So far two days is the longest I've been able to work without a full relapse of symptoms."	(Skilbeck et al., 2023)
Information, Knowledge & Health Literacy			"Having what I consider to be long COVID is destroying my life… I have worked over the years to build a successful business, which I am finding hard to maintain."	(Skilbeck et al., 2023)

179

Top-level Theme	Author generated Theme	Author generated Sub-Themes	Quotes	Citation
Information, Knowledge & Health Literacy		Strained communication with management	“They make you feel like your issues aren’t worth their time.” (P8)	(Boutry et al., 2024)`
Information, Knowledge & Health Literacy	full restoration of prior states of health	questioned in terms of achievability	I got a perching stool for the kitchen and a shower stool […] if I had a shower I had to go back to bed for an hour– it absolutely wore me out and that went on for [a while]. (Amanda, ICU)<br>IE 2. So, to pace properly you need to have a very strict timetable of rests. […] If I had a morning of doing something I would be in bed all afternoon [and it's] not a choice […] it's like‘If I don't go to bed now, I am going to fall down’. (Elsa, LC)<br>IE 3. You have to do things totally differently and respect your body [more, but] also find out that you can't do what you used to be able to do. (David, ICU)<br>IE 4. [what's helped me most is] learning what my new rules are in my body […] you're building up a map of yourself and once you have the map, it becomes easier to traverse. (Charles, LC)	(MacLean et al., 2025)
Information, Knowledge & Health Literacy		Guilt from receiving support and returning too early	“I felt guilty really... I had improved to be able to return to some form of work.” (P11)	(Boutry et al., 2024)`
Information, Knowledge & Health	Diagnosis opens social and		“Sometimes it’s nice just to have a chat with other parents what they’re in the same situation.”	(Faux-Nightingale et al., 2025)

Top-level Theme	Author generated Theme	Author generated Sub-Themes	Quotes	Citation
Literacy	emotional support networks			
Information, Knowledge & Health Literacy		- Reliance on online communities or self-research	"We're all out here trying to find our own solutions." – P013, woman	(Kalfas et al., 2024)
Information, Knowledge & Health Literacy	Lack of Information and Support	- Widespread lack of clinical knowledge about PCS<br>- Absence of clear guidance or resources	"It's been frustrating not to have solid information." – P001, woman<br>"A really long wait of complete unknowns." – P003, woman	(Kalfas et al., 2024)
Information, Knowledge & Health Literacy		- Desire for exploratory, low-risk treatments	"I'm willing to try… and my doctors at [telehealth clinic] are willing to try." – P22, 50s, West	(Laestadius et al., 2024)
Information, Knowledge & Health Literacy		Delayed care and emotional toll	"Have I missed the time to get some treatment or something?" – Susan (Female, White British, 50s)	(Fang et al., 2024)
Information,		- Desire for	"The dream is... one place you go, and they coordinate all the care." – P13,	(Laestadius et

181

Top-level Theme	Author generated Theme	Author generated Sub-Themes	Quotes	Citation
Knowledge & Health Literacy		integrated care model	40s, Northeast	al., 2024)
Information, Knowledge & Health Literacy		De-personalised and postcode-dependent care	“It just seems so random, what people are getting and aren’t getting.” – Lucy (Female, White British, 50s)	(Fang et al., 2024)
Information, Knowledge & Health Literacy		- Absence of centralised care	“I really wish if you’re going to call yourself a Long COVID clinic... that you are not just a place where you shuttle people.” – P19, 40s, Northeast	(Laestadius et al., 2024)
Information, Knowledge & Health Literacy			“I was relieved when I got COVID a second time because I could finally get a positive test.” – P22, 50s, West	(Laestadius et al., 2024)
Information, Knowledge & Health Literacy		- Requirement for positive COVID test	“I was turned away because I don’t have a positive test for COVID.” – P14, 30s, West	(Laestadius et al., 2024)
Information, Knowledge & Health Literacy			“She still didn’t want to fill out the paperwork, so I kind of got stuck with that.” – P29, 20s, West	(Laestadius et al., 2024)

182

Top-level Theme	Author generated Theme	Author generated Sub-Themes	Quotes	Citation
Information, Knowledge & Health Literacy		Advocating for self in healthcare	“I had to push to even get a referral. They just said it’s anxiety.”	(Leggat et al., 2024)
Information, Knowledge & Health Literacy		Patient-initiated follow-up	“If you feel you need to come back to us, you initiate another follow-up.” – Rehabilitation Coordinator	(Fang et al., 2024)
Information, Knowledge & Health Literacy		Social isolation and misunderstanding	“People think you’re making it up. Because you look okay, they don’t get it.”	(Leggat et al., 2024)
Information, Knowledge & Health Literacy		Inter-Trust coordination issues	It’s destabilising… each Trust has their own policies and procedures.” – Service Manager 1	(Fang et al., 2024)
Information, Knowledge & Health Literacy	Societal awareness	Educate society	- "A very significant number of people don’t take it seriously. They think it’s just being lazy or a bit shiftless... If we could kind of somehow get rid of that stigma…" — David<br>- "Sometimes you’ll tell somebody you’ve got Long Covid and they jump back three paces because they’re gonna catch it… there’s a lot of ignorance around it." — Alison<br>- "Maybe it needs to be on the news… Maybe it needs to be shown how many people are actually suffering with it… and then people might be	(Miller et al., 2024)

183

Top-level Theme	Author generated Theme	Author generated Sub-Themes	Quotes	Citation
			more understanding." — Megan<br>- "…get it out there, get it in the news, get it publicised and I'm sure eventually it would filter through and people will be aware of it..." — Lisa<br>- "So I think if you could make it more like serious... other people would then take it more seriously, and then that would then help us because we don't have to explain ourselves…" — Rachel<br>- "I think maybe art can bridge some of that gap a little bit… I wrote some poetry about my experience with Long Covid and showed it to my friend. They said it helped them kind of empathise a little bit more…" — Angela	
Information, Knowledge & Health Literacy		Treatments	- "I'm happy to give my time and my blood and you know to research, because if it helps us finding cures and solutions… Make something useful out of something, yeah, that's not great to experience." — Rose<br>- "I guess just listen to your participants when they come for that day... they might come one day and they're feeling really good and they might come the next day and feel like [rubbish]." — Kelly	(Miller et al., 2024)
Information, Knowledge & Health Literacy	Research	Symptoms and management	- "The more they understand about the physical, especially the lungs, the more they understand that... the better you're gonna be mentally knowing what's wrong, what you're up against." — Peter<br>- "Some kind of understanding or research on the reasons for the kind of what I feel is like a physical collapse from being really quite fit to feel like there's a disconnect between our different parts of the body..." — Samantha	(Miller et al., 2024)
Information, Knowledge &		Disability support	- "I had to stop on my full time job and get a part time job… which was not convenient for my financial situation. So I really need more	(Miller et al., 2024)

184

Top-level Theme	Author generated Theme	Author generated Sub-Themes	Quotes	Citation
Health Literacy			intervention in my financial level." — Max<br>- "The government also needs to recognise that long COVID, it is a serious illness and they need to sort that out. There needs to be some sort of a subsidy." — Lisa<br>- "I'm not actually sure whether it's classed as a disability… because I asked about getting a blue badge... I got told I don't think you'll get one because it's not been classed as disability yet." — Alison<br>- "I wouldn't say I was particularly disabled... sometimes I think something like that [blue badge] will be quite handy because I'm terrible with inclines." — Alison<br>- "If we could have like funding and help for a cleaner to come that would take pressure off me... and do things you enjoy more, like walking with friends." — Rose	
Information, Knowledge & Health Literacy			"There is no support available to help manage my symptoms… I feel a little bit on my own."	(Burton et al., 2024)
Information, Knowledge & Health Literacy		Efforts to maintain or reshape social life	"This [being social] I must plan. I need to plan time for recovery afterwards… I cannot just see people every day as I could before."	(Burton et al., 2024)
Information, Knowledge & Health		Psychological impacts and fears of COVID	- "I have flash backs; I can't remember a lot..."<br>- "COVID free everything has got to be COVID free..."	(Reay et al., 2024)

185

Top-level Theme	Author generated Theme	Author generated Sub-Themes	Quotes	Citation
Literacy				
Information, Knowledge & Health Literacy		Emotional toll of cancelled plans	"I try not to burden myself too much, too fast… but it backfires… then I have a terrible headache, and I am just so tired."	(Burton et al., 2024)
Information, Knowledge & Health Literacy		Hair loss	- "My hair is dropping out in handfuls..."<br>- "She (GP) said you will not go bald, and it should come back..."	(Reay et al., 2024)
Information, Knowledge & Health Literacy		Fear of underlying illness	"Do you have a tumour in your head or what the hell is this?"	(Burton et al., 2024)
Information, Knowledge & Health Literacy		Communication and psychological well-being	- "You know I mean, I'm thankful for being here... but at the same time, everything is not always just about your physical health..."<br>- "When I was in intensive care, I was very weepy and I was very frightened."	(Reay et al., 2024)
Information, Knowledge & Health Literacy	Schools and HCPs sometimes supportive even without formal		"School have been fab[ulous]…we put an action plan in place and a care plan."	(Faux-Nightingale et al., 2025)

186

Top-level Theme	Author generated Theme	Author generated Sub-Themes	Quotes	Citation
	diagnosis			
Information, Knowledge & Health Literacy		Few problems or complications	“10.5 percent of recovered participants stated having few problems and two of them had complicated health issues.”	(Silwal et al., 2023)
Information, Knowledge & Health Literacy		- Guilt over not contributing to relationships	“For over a year, I didn’t cook a single meal. The guilt was immense.” – P002, woman<br>“It affects everything—relationships, sexuality, all of it.” – P017, man	(Kalfas et al., 2024)
Information, Knowledge & Health Literacy	Experience of therapies		“I’ve done quite a bit of occupational and physical therapy tailored just to me. That already does a lot for me…” (F4, P1)	(Schmachtenberg, Königs, et al., 2023)
Information, Knowledge & Health Literacy		Changes in habits	“Few persons claimed to have quit drinking when infected with COVID-19.”	(Silwal et al., 2023)
Information, Knowledge & Health Literacy		Social health level distribution		(Silwal et al., 2023)
Information, Knowledge &		Poor social health linked to place of	“More than half (55%) of recovered individuals who stayed in the hospital had poor social health, followed by quarantine centers (47.7%) and home	(Silwal et al., 2023)

187

Top-level Theme	Author generated Theme	Author generated Sub-Themes	Quotes	Citation
Health Literacy		stay	isolation (47.3%)."	
Information, Knowledge & Health Literacy		Ignorance and misbehavior from society	"I have faced ignorance and embracing moments from society." – 32 years, Police, Madhesh province; "In our village/society does not look corona infected people in a positive way…" – 49 years, foreign employment, Province-1.	(Silwal et al., 2023)
Information, Knowledge & Health Literacy			"I've been doing regular exercise… fitness training, which is called T-RENA… And it's good for me" (F3, P6)	(Schmachtenberg, Königs, et al., 2023)
Information, Knowledge & Health Literacy			"The coping group… gave us a lot, built us up. […] You can try out a lot of things… very helpful" (F4, P4)	(Schmachtenberg, Königs, et al., 2023)
Information, Knowledge & Health Literacy		Concealing infection to avoid prejudice	"Others choose not to tell society about their infection to avoid any social indifference…	(Silwal et al., 2023)
Information, Knowledge & Health Literacy		Negative feelings due to societal behaviour	"I have felt the difference in my mental status... due to the behaviour of society towards the corona infected people."	(Silwal et al., 2023)
Information,			"You'll have to wait another year for that'. I say, 'When I'm dead, I don't	(Schmachten

Top-level Theme	Author generated Theme	Author generated Sub-Themes	Quotes	Citation
Knowledge & Health Literacy			need an appointment anymore'" (F3, P6)	berg, Königs, et al., 2023)
Information, Knowledge & Health Literacy	information exchange and expectation setting		Hearing similar stories, gathering information, and being given information too, new information about where to seek help at, and additional resources for recovery and support too, has been really good.	(Sarma et al., 2025)
Information, Knowledge & Health Literacy	Forging a Path Through Uncertainty		Not everyone is like me and I accept that. The people I met [at long COVID clinic] were from all different social and academic backgrounds but I think that more should be leveraged of the people who have gone through it and want to give back or who have survived it and want to give back (. . .) It's like trying to grow a pearl without a piece of grit in an oyster. They've got the grit and they've got a number of us who have been through it but there has been no thinking that way to think about leveraging that overwhelming desire to give back that those of us who have survived want to do. (Darren)	(Milne et al., 2025)
Information, Knowledge & Health		Self-motivation as inner strength	"I am young, I convince myself that nothing will happen to me." – 27 yrs, Police, Bagmati Province.	(Silwal et al., 2023)

189

Top-level Theme	Author generated Theme	Author generated Sub-Themes	Quotes	Citation
Literacy				
Information, Knowledge & Health Literacy		"This statement also illustrates... a trigger for worries and fears."	"I keep asking myself where does this lead to in the future?" (P13)	(Schmachtenberg, Müller, et al., 2023)
Information, Knowledge & Health Literacy			"There is no one to help you with it. You have to do everything yourself" (F3, P6)	(Schmachtenberg, Königs, et al., 2023)
Information, Knowledge & Health Literacy		"Younger participants in this study struggled with the loss of spontaneity and flexibility."	I lack the aspect of physical exercise and I don't see myself as being as flexible and spontaneous as I would like to be at 23... if I do this now, the next 3 days are not really feasible." (P21)	(Schmachtenberg, Müller, et al., 2023)
Information, Knowledge & Health Literacy	Negative experiences with medical care		"He doesn't know how I feel at all. He doesn't know what's happening to my body at all… I felt so lost and so alone…" (F4, P3)	(Schmachtenberg, Königs, et al., 2023)
Information, Knowledge & Health Literacy		"Some people in his environment could not understand why he so strongly	"I would really like to go back to work and others tell me to enjoy the time at home and just relax." (P17	(Schmachtenberg, Müller, et al., 2023)

190

Top-level Theme	Author generated Theme	Author generated Sub-Themes	Quotes	Citation
		wanted to return to his occupation...”		
Information, Knowledge & Health Literacy		One participant struggled with the fact that she could no longer meet her own demands...”	“I write a sentence and change my verbs three times in the sentence and then realize that the whole sentence order is no longer correct... I’m totally annoyed with myself. It’s still like that.” (P9)	(Schmachtenberg, Müller, et al., 2023)
Information, Knowledge & Health Literacy		“These individuals reportedly feel they are missing out on opportunities and experiences...”	“I’ve never felt so much like going to work and fixing a windmill again. I actually kind of miss that.” (P17)	(Schmachtenberg, Müller, et al., 2023)
Information, Knowledge & Health Literacy		“It was a daily struggle for me...”	“That is my very greatest wish, to go back to my job.” (P17); “So not a day went by where I didn’t think about work.” (P1)	(Schmachtenberg, Müller, et al., 2023)
Information, Knowledge & Health Literacy		“Some respondents identify significant changes regarding their own personality.”	“I am actually someone who likes to be very social and help and support... I realize that I can’t offer the help that I would otherwise like to offer.” (P1)	(Schmachtenberg, Müller, et al., 2023)
Information, Knowledge &			“I was told ‘there is nothing. Take an ibuprofen.’ But I can’t take ibuprofen every day either…” (F2, P2)	(Schmachtenberg, Königs,

191

Top-level Theme	Author generated Theme	Author generated Sub-Themes	Quotes	Citation
Health Literacy				et al., 2023)
Information, Knowledge & Health Literacy		Fear affecting activity levels	"Some recovered individuals did fewer physical activities than normal… a few people did not take part in any of the activities because they were terrified of reinfection."	(Silwal et al., 2023)
Information, Knowledge & Health Literacy		"Contrary, five participants explicitly emphasized that they experience little consideration...	"I also had two colleagues who complained about me to the staff council, along the lines of 'I always have to cover for her because she is sick.'" (P22)	(Schmachten berg, Müller, et al., 2023)
Information, Knowledge & Health Literacy	Worsened pre-existing conditions	Chronic illness exacerbation	"She got COVID again… it affects more her asthma, made her asthma worse."<br>"I had a mild headache… then a hard time to breathe; I… received B cell treatment… so I have no antibodies to fight."	(Messiah et al., 2023)
Information, Knowledge & Health Literacy	Suggestions for supportive measures		"I would like to see maybe a campaign like that from the federal government about it [long COVID]" (F1, P5)	(Schmachten berg, Königs, et al., 2023)
Information, Knowledge & Health	sharing experiences and		…Everyone helped to motivate everybody else…And people were really open about the things that they had struggled with… Int29	Seers et al., 2025)

192

Top-level Theme	Author generated Theme	Author generated Sub-Themes	Quotes	Citation
Literacy	addressing worries		…Actually for me, honestly, the best, best thing was talking to other people and learning about their experiences and actually knowing how similar actually our experiences were… Int17	
Information, Knowledge & Health Literacy		Dependent or requiring assistance	"6.2 percent of recovered individuals were reliant on completing housework... 7.4 and 8.2 percent...dependent on activities outside the house and on financial work, respectively."	(Silwal et al., 2023)
Information, Knowledge & Health Literacy	Social anxiety and depressio	Anxiety, isolation, fear	"In school, if someone is talking loudly or making more noise, she gets anxiety."<br>"She is afraid of separation from me (the mother)."<br>"Doesn't want to go out, cries and says just get something for me. I don't want to go out."	(Messiah et al., 2023)
Information, Knowledge & Health Literacy	knowledge and interpretatio n practices		It seems like every new symptom is new to me, and it's new to the doctor and…it's like you're lost…nobody can help you. And you don't know what's going on with you. And when your doctor doesn't know what's going on with you, then what do you do?	(Sarma et al., 2025)
Information, Knowledge & Health Literacy			"That would be the smart thing… a centre… the psychologist is there, the rheumatologist is there…" (F3, P2)	(Schmachten berg, Königs, et al., 2023)
Identity,		Cyclic or Relapsing	"…felt like nothing ever happened... but then afterwards it kinda came	(Hitch et al.,

193

Top-level Theme	Author generated Theme	Author generated Sub-Themes	Quotes	Citation
Meaning & Recovery Trajectories		Recovery	back…” (N5, M, 30–49)<br>“It gets really hard, then you get better and then it comes back and hits you a second time.” (N2, M, 50+)	2023)
Identity, Meaning & Recovery Trajectories	- The landscape behind a Long COVID experience<br>- Everyday experience (partial)	Uncertainty and complexity	“I had to stop work because I couldn’t get through a day without feeling like I’d collapse.”	(Leggat et al., 2024)
Identity, Meaning & Recovery Trajectories	different responses to illness	differences in other people’s responses to their illness		(MacLean et al., 2025)
Identity, Meaning & Recovery Trajectories	Biopsychosocial Understanding	Agreement on the interaction between body and mind	“When the body functions properly, the mind often functions better as well.” (P2)<br>“You are disappointed in yourself, because you do so much. Why the hell doesn’t it get any better?” (P16)	(Funk et al., 2025)
Identity, Meaning & Recovery	Adapting to an altered life	Loss of ‘self’ and identity impact	“I just want my life back... I see myself just becoming a burden.” (IV4)<br>“The majority... had been unable to resume activities that were previously central to their core identity...”	(Humphreys et al., 2021)

194

Top-level Theme	Author generated Theme	Author generated Sub-Themes	Quotes	Citation
Trajectories				
Identity, Meaning & Recovery Trajectories		Compensatory strategies to maintain work identity	"Work is my identity and I've gone to extreme lengths to stay working, but every single task is negotiated, planned and recovered from."	(Skilbeck et al., 2023)
Identity, Meaning & Recovery Trajectories	The Recovery Journey	- Expectations<br>- Individual and varied journeys<br>- Mental and physical improvements<br>- The journey continues<br>- Self-values and identity	"I just couldn't believe how weak, just my general let alone my breathing. I mean my breathing was appalling. But yeah I just couldn't believe how poorly I felt really." (Participant B)<br><br>"We were a completely different group with completely different individuals there..." (Participant E)<br><br>"So after the rehabilitation I could go out, I could go out for a walk, for hour's walk and come back and feel happy without feeling that tired..." (Participant F)<br><br>"I believe it's very important to keep up the exercise and it [rehabilitation] plants it in your mind." (Participant J)<br><br>"You know what, I'm privileged to be a survivor." (Participant G)	(Gerlis et al., 2022)
Identity, Meaning & Recovery Trajectories	perceptions of recovery	shaped by the novel, unknown nature of COVID-19	[I was] previously active, playing rugby […] living a normal life, and now […] everything has to be planned[like]<br>"Where am I going to park [at the supermarket]?" […] Previously I could just leave the house without a concern [it's] hard	(MacLean et al., 2025)

195

Top-level Theme	Author generated Theme	Author generated Sub-Themes	Quotes	Citation
			to adjust to. (Elliott, LC)<br>Everything is different. What I feel, […] what I think, how I behave […] I have to think before I do anything. Even watching<br>TV. […] It's like I'm a different person. (Nina, ICU)<br>[T]here's no clear answers really to say,“Well, this is going to happen and then that's going to happen” [and] you're left<br>worried, thinking,“Oh, is this normal to be feeling like this? [Is it] expected or has [COVID] damaged something?”	
Identity, Meaning & Recovery Trajectories	Facing Psychological Threat		I remember being woken up and I just remember<br>these incredibly bright lights and people were<br>obviously wearing full PPE but I didn't quite<br>know what it was because it just looked crazy.<br>People were in these masks and suits (. . .) I can't<br>really describe it, but it was just like this alien<br>world. (Claire)	(Milne et al., 2025)
Identity, Meaning & Recovery Trajectories	Anxiety and frustration	Worry about recovery and decline	“At that point I was, like, I probably have it. But as far as recovering, that's what I was more worried about; like, when is this going to be over? Like I don't feel I'm getting better. If anything, I'm declining. So, it was more of like a feeling of anxiousness and, like, frustration.” (Participant #6)	(Santiago-Rodriguez et al., 2022)
Identity, Meaning & Recovery Trajectories		Seeking control and meaning	Whining doesn't help… you must contribute 80%, and that's it, right?"	(Burton et al., 2024)

196

Top-level Theme	Author generated Theme	Author generated Sub-Themes	Quotes	Citation
Identity, Meaning & Recovery Trajectories	Changes in social engagement	Withdrawal from social activities	"I cannot stand when several people are talking simultaneously… I cannot stand noise."	(Burton et al., 2024)
Identity, Meaning & Recovery Trajectories	Physical activities post-recovery	Performing normal activities	"More than 80 percent of recovered COVID-19 participants performing all normal physical activities, such as household chores, other activities outside the home, and other professional activities."	(Silwal et al., 2023)
Identity, Meaning & Recovery Trajectories	Life Before and After Long COVID	- Loss of physical ability and lifestyle<br>- Feeling like a different person	"I used to run half-marathons. Now I can't run at all." – P002, woman<br>"My life has changed 100%." – P015, man	(Kalfas et al., 2024)
Identity, Meaning & Recovery Trajectories	Seeking Legitimisati on		they were very, very helpful. They kept telling me not to rush back to work which was really good and in the end, I think I had about six to eight weeks off. It was very much about me reco-vering and concentrating on myself. (Richard)	(Milne et al., 2025)
Identity, Meaning & Recovery Trajectories	Role conflicts, identity crises, and "new reality with long	"These areas included partnership, relationship with children and parents, reputation among	"I no longer feel like a main pillar in the family. I am the father... but at the moment I feel pretty worthless." (P17)	(Schmachten berg, Müller, et al., 2023)

197

Top-level Theme	Author generated Theme	Author generated Sub-Themes	Quotes	Citation
	COVID"	acquaintances and friends..."		
Identity, Meaning & Recovery Trajectories		Incomplete recovery	"If I have to climb the stairs, no, but I'd like to" (A024, female, 69)	(Schiavi et al., 2022)
Identity, Meaning & Recovery Trajectories		Disruption to identity and life roles	"It's like I don't know myself anymore—I can't do what I used to."	(Leggat et al., 2024)
Identity, Meaning & Recovery Trajectories		"Some interviewees were no longer able to engage in several of the leisure activities that were meaningful to them."	"None of that works anymore. That leaves me watching sports on TV when something like the Olympic Games... comes on." (P25)	(Schmachtenberg, Müller, et al., 2023)
Identity, Meaning & Recovery Trajectories		Benefits of the LC-OHP	…it sort of captures all of me… I felt really supported throughout the programme… I found it very therapeutic…"	(Al-Jabr et al., 2024)
Identity, Meaning &		Experienced Control over Recovery	I bit off more than I could chew. [...] I relapsed completely, and that was a shame." (P4R, M)	(Schaap et al., 2022)

198

Top-level Theme	Author generated Theme	Author generated Sub-Themes	Quotes	Citation
Recovery Trajectories				
Identity, Meaning & Recovery Trajectories		- Emotional toll, identity disruption	“I used to be a happy girl… I somehow became more and more introverted…People said that there was something different about me.”	(Loft et al., 2022)
Identity, Meaning & Recovery Trajectories	Episodic and undulating illness pattern	Fluctuating recovery	“Every day is a new day.”	(Al-Jabr et al., 2024)
Identity, Meaning & Recovery Trajectories	Trajectories of Recovery	Complete Recovery	“I got a phone call on the Wednesday saying that I tested positive, and I was actually completely fine at the time…” (N24, F, 18–29)	(Hitch et al., 2023)
Identity, Meaning & Recovery Trajectories	Emotional impact on identity and recovery	Emotional distress	“I was sobbing with the pain.”	(Al-Jabr et al., 2024)
Identity, Meaning & Recovery Trajectories	heterogeneity of 'recovery trajectories'		IE 1. [F]or the first few days [after I came out of hospital] I continued getting stronger […] then after about 2 weeks things started to slow down […] like I was backpedaling, I was getting worse [and it's] been the same [since]. (Kenneth, ICU)<br>IE 2. So things are improving [but when] I spoke to [my consultant] I	(MacLean et al., 2025)

199

Top-level Theme	Author generated Theme	Author generated Sub-Themes	Quotes	Citation
			said,‘Oh, this recovery, it's taking so long’ he said,‘Okay, just go back to February. […] how were you then? The same as [now]?’ […] I went,‘Oh, no, no’. And my nurse [said], ‘You're not on the oxygen now either’. […] And I looked at them both and went,‘Yeah okay, point made’ . (Amanda, ICU)<br>IE 3. So, I suppose every month that I can look back and think,‘Okay, I did just a tiny bit more that month’. I feel […] good [it's] going in the right direction. The difficult thing is when you have the blips and horrible relapses […] that's really quite depressing. (Holly, LC)<br>IE 4. I can potter around and do […] simple things […] and [the healthcare professionals] just tell me it's time [and] can take 5 years before you're totally over [admission with critical illness]. (Ella, ICU)	
Identity, Meaning & Recovery Trajectories	An Unexpected Journey: How LTCS Conquer Everyday Life	- Sudden or delayed onset of LTCS<br>- Difficulty distinguishing recovery from ongoing illness<br>- Cognitive and physical deficits	“I thought…yes; I have survived! And then I thought…I can handle everything…But then it came so suddenly.”<br>“I simply cannot figure out how to read a recipe… I no longer can.”	(Loft et al., 2022)
Identity, Meaning &		Identity	"[Illness] changes you. Yeah, you notice that it changes you." (P7NR, F)	(Schaap et al., 2022)

200

Top-level Theme	Author generated Theme	Author generated Sub-Themes	Quotes	Citation
Recovery Trajectories				
Identity, Meaning & Recovery Trajectories	Psychological Impact	- Depression and distress from reduced functioning<br>- Struggles with emotional burden and illness identity	"From running marathons to barely walking to the shop—it's depressing." – P008, woman<br>"I'm so sick of being a sick person." – P010, woman	(Kalfas et al., 2024)
Identity, Meaning & Recovery Trajectories		Adjusting to a "new normal"	"I don't think I will return to the same level as I was before… but it's not something my body wants either… Something has happened to me."	(Burton et al., 2024)
Identity, Meaning & Recovery Trajectories	different permutations and lack of linearity in recovery			(MacLean et al., 2025)
Identity, Meaning & Recovery Trajectories	Identity Shifts: Accelerated Aging and Loss of Independence	Feeling older despite actual physical function	"I am 77, but I never thought of myself as old… Somehow… I sometimes mentally feel like I am an old lady, and it bothers me a lot [Patient ID 77]."	(Duan et al., 2023)

201

Top-level Theme	Author generated Theme	Author generated Sub-Themes	Quotes	Citation
Identity, Meaning & Recovery Trajectories	'I Can't Rest Anymore': Re-Introducing Physical Activity	Boom and Bust	'...the first time I went for a walk was probably only two miles and I ended up going to bed for two to three hours afterwards' (F06; 10 months post-infection)<br>'I went for a swim and then I had heart palpitations for about twenty hours...' (F03; 7 months post-infection)<br>'I've never had a sustained period of feeling well enough...' (F04; 7 months post-infection)<br>'Thought I can't rest anymore and sleep all day and night so started pushing myself...' (F14; 10 months post-infection)	(Shelley et al., 2021)
Identity, Meaning & Recovery Trajectories		Functional Recovery in Daily Activities	"Getting from our bedroom… down to the kitchen and back again. So I struggled... but now... well and recovered." (N13, F, 50+)<br>"I'm back, walking, playing golf, doing all the things that I do." (N3, M, 50+)	(Hitch et al., 2023)
Identity, Meaning & Recovery Trajectories		Identity-focused recovery support	"A flexible and resilient self" – Service description (Phase 2)	(Fang et al., 2024)
Identity, Meaning & Recovery Trajectories	Getting Back to 'Some Sort of Normal'	Personalized Meaning of Recovery	"I think I'm pretty well getting back to normal now." (N20, F, 50+)<br>"I'm like 90% back to not having the impacts of it." (N46, M, 30–49, HCW)	(Hitch et al., 2023)
Work, Finances &		Changed capacity to	"Not working or had greatly reduced hours." "Working from bed or the	(Wurz et al.,

202

Top-level Theme	Author generated Theme	Author generated Sub-Themes	Quotes	Citation
Role Changes		work	couch… setbacks they were facing along the way.”	2022)
Work, Finances & Role Changes		- Impact on roles and relationships<br>- Struggles with career and caregiving expectations	“I was losing my career, I was losing everything.” – P005, woman<br>“Women in caring roles, now barely able to function.” – P014, woman	(Kalfas et al., 2024)
Work, Finances & Role Changes	Dealing with COVID-19	Support	"You didn’t get support. Not from your GP. Hospital was too busy with other things." (P4R, M)	(Schaap et al., 2022)
Work, Finances & Role Changes		The lack of sensitivity from their work environment represented an additional burden...	“In my case, the superior slightly favors the direction of negating everything and rejecting everything and then positions himself more on the side of those who are rebelling against the state.” (P12)	(Schmachten berg, Müller, et al., 2023)
Work, Finances & Role Changes	Asking for help when few are listening, and little is working	Support received	“Ignored or dismissed by their primary healthcare providers.” “Feeling defeated, helpless, invisible, or frustrated.” “Some… begged for help.”	(Wurz et al., 2022)
Work,		Due to these	Now I lie down most of the day, and before, I was up on my feet all day.”	(Schmachten

203

Top-level Theme	Author generated Theme	Author generated Sub-Themes	Quotes	Citation
Finances & Role Changes		changes, roles such as the ‘power woman’... could no longer be fulfilled.”	(P25)	berg, Müller, et al., 2023)
Work, Finances & Role Changes		Employment support	- "But [medical tests] come back normal and you’ve got Long Covid... my boss wants to see some kind of medical evidence... because he thinks I’m lying." — Alison<br>- "I was off work two years and I had to fight with work because they couldn’t understand why I couldn’t come back... I took a grievance out and I’ve got a part time [job]." — Alice<br>- "I was one of the top salespeople... I feel like I’m probably one of the most unreliable people there... People at work just don’t get it all." — Thomas<br>- "You need to have a much more understanding, forgiving approach... the support you get in terms of paid sick leave or shorter hours or different ways of working." — Margot	(Miller et al., 2024)
Work, Finances & Role Changes		Guilt, loss of roles, and dependence on others	"I feel really dependent on my boyfriend…which is deeply frustrating…It is very disabling."	(Burton et al., 2024)
Work, Finances & Role Changes	Workplace challenges	Unsustainable return to work and presenteeism	“I've not stayed off long term and listened to my body... then suffered in my days off.”	(Cooper et al., 2024)
Work, Finances &		Need for workplace accommodations	"After every meeting or every class, I just had to lie in bed for at least an hour… And I had no energy for cooking, shopping or cleaning…"	(Burton et al., 2024)

204

Top-level Theme	Author generated Theme	Author generated Sub-Themes	Quotes	Citation
Role Changes				
Work, Finances & Role Changes	Return-to-work challenges	Premature returns worsening LTCS	"I should have stayed home for at least 14 days more. It was the dumbest thing I did… to go to work."	(Burton et al., 2024)
Work, Finances & Role Changes	Affordability	Out-of-pocket costs; low insurance reimbursements; income loss	"My savings have melted away very, very, very much in the last year and a half." (P13)	Gamillscheg et al., 2024)
Work, Finances & Role Changes	Lack of Care Coordination	Unclear role delineation between post-COVID and primary teams; duplications and missed follow-ups		(O'Hare et al., 2022)
Work, Finances & Role Changes		Similar to this person, other respondents also stated that the distribution of roles in the partnership changed..."	"Because of the illness... my husband really did everything, had to do everything, because I couldn't." (P1)	(Schmachtenberg, Müller, et al., 2023)
Work, Finances &	The Importance	Work as a Milestone for Normalcy	"I returned back to work and when I walked in… it was just very overwhelming." (N36, F, 50+, HCW)	(Hitch et al., 2023)

205

Top-level Theme	Author generated Theme	Author generated Sub-Themes	Quotes	Citation
Role Changes	of Work			
Work, Finances & Role Changes		Perceived benefit of occupational health advice	“I’ve got some really nice information from my occupational health... it’s really sensible.” (P15)	(Boutry et al., 2024)`
Work, Finances & Role Changes	Support from the work environment	“Eight interviewees stated that they perceived emotional support...”	“For me, going into the home office was a bit of self-protection [...]. I just found it a relief. I could work [...] and could also protect myself and others.” (P4)	(Schmachtenberg, Müller, et al., 2023)
Work, Finances & Role Changes	Financial Burden due to Hospitalization		“I pledged my home and property… spent 10 Lakhs” (57 Years/Male)<br>“We both were daily wage labourers… pledged silver and gold ornaments” (38/Female)	J et al., 2025)
Work, Finances & Role Changes	Socioeconomic stress	Financial difficulties due to pandemic	“We receive less salary than before… we used to borrow money from friends to buy nutritious food.”	(Silwal et al., 2023)
Work, Finances & Role Changes	‘I Can’t Really Work’: Challenges of Returning to Work	Physical and Mental Strain of Work	‘...if you can’t walk up a hill, you can’t test a rucksack, you can’t test boots, you can’t use this stuff and if you can’t use your brain to write stuff... it’s rendered me incapable of working’ (M12; 10 months post-infection)<br>‘Months afterwards extremely fatigued and falling asleep at the desk/taking power naps’ (F14; 10 months post-infection)<br>‘...where I’m normally, you know, in the front, come on, let’s go, not	(Shelley et al., 2021)

206

Top-level Theme	Author generated Theme	Author generated Sub-Themes	Quotes	Citation
			now... I'm the one that's trailing and that's not me' (M02; 6 months post-infection)<br>'...with the brain fog, I'm really quite forgetful...' (F11; 9 months post-infection)	
Work, Finances & Role Changes	Strong desire and need to return to work motivated by sense of purpose and financial precarity		"Being able to keep working part-time (my work is sedentary, so my physical symptoms don't affect it much) has really helped me, giving me a sense of regularity, purpose, and meaning, and keeping my mind ticking over."	(Stelson et al., 2023)
Work, Finances & Role Changes	the hard physical and emotional work of adjusting to changed selves		IE 1. I got a perching stool for the kitchen and a shower stool […] if I had a shower I had to go back to bed for an hour– it<br>absolutely wore me out and that went on for [a while]. (Amanda, ICU)<br>IE 2. So, to pace properly you need to have a very strict timetable of rests. […] If I had a morning of doing something I would be<br>in bed all afternoon [and it's] not a choice […] it's like'If I don't go to bed now, I am going to fall down'. (Elsa, LC)<br>IE 3. You have to do things totally differently and respect your body [more, but] also find out that you can't do what you used to<br>be able to do. (David, ICU)<br>IE 4. [what's helped me most is] learning what my new rules are in my	(MacLean et al., 2025)

207

Top-level Theme	Author generated Theme	Author generated Sub-Themes	Quotes	Citation
			body […] you're building up a map of yourself and once you have the map, it becomes easier to traverse. (Charles, LC)	
Work, Finances & Role Changes	Lack of understandin g and support from employers and colleagues hinders sustainable return	Desire for validation and formal accommodations	"I was treated like I was making it up. I needed someone to say: 'I believe you. Let's work this out together.'"	(Stelson et al., 2023)
Work, Finances & Role Changes		Employer support made a critical difference	"My manager said: 'Your health comes first. We'll phase you back slowly and review weekly.' That saved me."	(Stelson et al., 2023)
Work, Finances & Role Changes		Overly tired after work	"I just wanted to concur that the little energy I have got, I spend it at work. And when I come home there is no energy left…"	(Gyllensten et al., 2023)
Work, Finances & Role Changes		(c) Work and financial difficulties	My weakness prevents me from delivering food… I do not know what to do [Patient ID 41]." / "I lost 2 of my jobs…I'm trying to get unemployment [benefits] but I haven't heard from anyone yet. [Patient ID 60]." / "I cannot work anymore… [Patient ID 41]."	(Duan et al., 2023)
Work,		Taking breaks at	"I take pauses. It is the way I have tried to handle it."	(Gyllensten et

208

Top-level Theme	Author generated Theme	Author generated Sub-Themes	Quotes	Citation
Finances & Role Changes		work		al., 2023)
Work, Finances & Role Changes		Adjusted work tasks	"My colleagues do some of my work and I have done a little more administrative tasks.	(Gyllensten et al., 2023)
Work, Finances & Role Changes		Flexible working hours	I have an incredible freedom in how I plan my time two days a week."	(Gyllensten et al., 2023)
Work, Finances & Role Changes	Possibilities to adjust work	Working from home	We have decided that I am allowed to work from home two days a week… otherwise it would probably have been pretty impossible to work those 80 percent that I work."	(Gyllensten et al., 2023)
Work, Finances & Role Changes	Communicat ion and support	Communication and support from management	She has told my colleagues… They have to be forgiving regarding that."	(Gyllensten et al., 2023)
Work, Finances & Role Changes		Mixed Reactions to Adjusted Work Roles	It was a graded return…" (N30, F, 30–49, HCW)<br>"I feel perfectly useless… how long until I can actually get back and support and help." (N6, F, 30–49, HCW)	(Hitch et al., 2023)
Work, Finances & Role Changes	Lack of understandin g and support from employers and	Desire for validation and formal accommodations	"I was treated like I was making it up. I needed someone to say: 'I believe you. Let's work this out together.'"	(Skilbeck et al., 2023)

209

Top-level Theme	Author generated Theme	Author generated Sub-Themes	Quotes	Citation
	colleagues hinders sustainable return			
Work, Finances & Role Changes		Inflexible policies and fear of job loss	"If I took another sick day, I was going to lose my job. So I went to work with chest pain, fever, the lot."	(Skilbeck et al., 2023)
Work, Finances & Role Changes		Employer support made a critical difference	"My manager said: 'Your health comes first. We'll phase you back slowly and review weekly.' That saved me."	(Skilbeck et al., 2023)
Work, Finances & Role Changes	Strong desire and need to return to work motivated by sense of purpose and financial precarity		"Being able to keep working part-time (my work is sedentary, so my physical symptoms don't affect it much) has really helped me, giving me a sense of regularity, purpose, and meaning, and keeping my mind ticking over.	(Skilbeck et al., 2023)
Work, Finances & Role Changes	Loss of Job due to COVID / Wage Loss		"They didn't give me a job" (33/Male/X-Ray Technician)<br>"I lost… Rs. 1,50,000… also had to pay for my medical expenses" (41/Male/Xerox shop)	J et al., 2025)

210

Top-level Theme	Author generated Theme	Author generated Sub-Themes	Quotes	Citation
Work, Finances & Role Changes		Work Supporting Recovery	'...the total hours I'm doing are probably about two thirds of what I would usually be doing and it's all from home...' (F09; 10 months post-infection)<br>'I've started parking further away, to get a bit of exercise... just to try and gradually increase my fitness levels a bit' (F04; 7 months post-infection)	(Shelley et al., 2021)
Work, Finances & Role Changes		Role of family and social support	"Friends and family acted as informal carers... supportive people around me just helping basically, just listening." (IV3)<br>"My mum would walk with me and look after me." (IV11)	(Humphreys et al., 2021)
Work, Finances & Role Changes		"Some of the participants tried everything to return to work or to college..."		(Schmachtenberg, Müller, et al., 2023)
Work, Finances & Role Changes		Disbelief and unrealistic work expectations	"…I kind of feel completely abandoned by the health team and left to kind of sort it out myself."	(Al-Jabr et al., 2024)
Work, Finances & Role Changes	Role Modification	Task adjustments (e.g., admin work)	"[My team leader] suggested I did clerical work instead." (P3)	(Boutry et al., 2024)`
Work, Finances & Role Changes	Financial Strain	Loss of income through phased return or unpaid leave	"You are meant to mix your hours with your annual leave and I think that's really unfair." (P1)	(Boutry et al., 2024)`

211

Top-level Theme	Author generated Theme	Author generated Sub-Themes	Quotes	Citation
Work, Finances & Role Changes		Need for tailored return and remote work flexibility	"It's got to be a flexible approach to how they manage to work." (P5)	(Boutry et al., 2024)`
Work, Finances & Role Changes		Inflexibility of return-to-work policies	"One day in school knocked me right back in terms of fatigue." (P9)	(Boutry et al., 2024)`
Work, Finances & Role Changes		Financial precarity	"Our budget, we only have like $300 … to spare and most of that is go[ing] to covering overdue and late charges"	(Brehon et al., 2023)
Work, Finances & Role Changes	Workplace Responses	Variability in employer support (OH vs HR)	"The occupational health people were wonderful. I mean they were so on the ball." (P9)	(Boutry et al., 2024)`
Work, Finances & Role Changes		"Every failed attempt to return to work was perceived as a sobering setback..."	"First [I worked] only 2 h, then only 3 h. But I always realized if I spend half an hour... on the computer, oh God [...] it did not work." (P18)	(Schmachten berg, Müller, et al., 2023)
Work, Finances & Role Changes		Work disruption and employment loss	"I'm still nowhere from being able to return-to-work which is really hard to deal with... I need something that's going to be really flexible"	(Brehon et al., 2023)
Work, Finances & Role Changes		Most of the individuals who returned to their	"This 20% that I'm now reducing, that's what's missing at my bank account. And no one compensates for that." (P12)	(Schmachten berg, Müller, et al., 2023)

212

Top-level Theme	Author generated Theme	Author generated Sub-Themes	Quotes	Citation
		jobs had to reduce their working hours...		
Work, Finances & Role Changes		Reliance on full pay for sustainability	“I’m lucky that I work for [public organisation] so I’m getting full pay... half pay would be a real difficulty.” (P2)	(Boutry et al., 2024)`
Work, Finances & Role Changes		Anxiety and self-doubt about readiness to work	I wouldn’t feel safe to return to such responsibility as my condition is unpredictable.” (P19)	(Boutry et al., 2024)`
Work, Finances & Role Changes	Relationship Changes	Feeling misunderstood or unsupported by colleagues	Some passive aggressive kind of comments... that haven’t been totally supportive.” (P2)	(Boutry et al., 2024)`
Work, Finances & Role Changes	Occupational situation	Most of the respondents were generally satisfied with their job situation prior to the illness...”	“[I was transferred to a position] at my work where the workload is much smaller than the normal daily work life on the ward... I still can’t manage my normal work like that.” (P1)	(Schmachtenberg, Müller, et al., 2023)
Work, Finances & Role Changes		Home demands competing with work, especially home-schooling	“Clamouring children, very attention-demanding... really hard to motivate myself.” (P8)	(Boutry et al., 2024)`

213

Top-level Theme	Author generated Theme	Author generated Sub-Themes	Quotes	Citation
Work, Finances & Role Changes		"Participants reported a reduced ability to cope with stress in daily working life."	"I notice that I am just not as resilient as I was before the corona infection and cannot be present as that might be necessary." (P14)	(Schmachtenberg, Müller, et al., 2023)
Coping, Self-Management & Resilience	Treatment options are critical and urgent	- Off-label prescribing<br>- Rejection of passive approaches<br>- Frustration with slow research translation	"I'll be dead in four years, like really. Just try something. Think outside of the box." – P25, 50s, Northeast	(Laestadius et al., 2024)
Coping, Self-Management & Resilience		Energy conservation and activity prioritisation	"I have to try and think well if I need to be around to look after my kids... I need to be very, very careful of what I do the day before." (IV5)<br>"I do the physical things that look after my mental health... blue skies do me the world of good." (IV7)	(Humphreys et al., 2021)
Coping, Self-Management & Resilience	Cognitive Restructuring	"Change in the mind": Opportunities for cognitive restructuring<br>"Plan B": Acceptance and reorientation	"I changed my mind suddenly and thought 'Now I am going to make it'. And indeed, things are improving now." (P3)<br>"I have the expectation that it [reading] will work again soon, so I am practicing." (P14)<br>Another helpful approach that was frequently mentioned was the practice of shifting one's attention to one's own progress, such as extending one's running route, or	(Funk et al., 2025)

Top-level Theme	Author generated Theme	Author generated Sub-Themes	Quotes	Citation
			focusing on the<br>progresses of other patients: “I focused more on the people who have found a way.” (P3)<br><br>“I trust in having a plan B. When I am unable to proceed, I reflect on what I had enjoyed in<br>the past and consider alternative ways to achieve it. Being creative can be enjoyable.” (P1)<br>“When I overload myself, I acknowledge it and try to accept it: ‘Okay, I have totally over-<br>loaded myself and that is the reality now.’” (P10)	
Coping, Self-Management & Resilience	Behaviour Change Exercise	• Promotion of self-confidence: Encouragement to carry out the exercise<br><br>• Criticism of the terms “avoidance	“That is what I did for myself. Ultimately, I realized that I didn’t need my avoidance strat-<br>egy or safety behavior because I was still able to resume after the appointment. Reflecting on<br>and comparing this experience to my expectations was beneficial for me.” (P15)	(Funk et al., 2025)

215

Top-level Theme	Author generated Theme	Author generated Sub-Themes	Quotes	Citation
		behaviour" and "result"<br>• Determining the framework conditions: Additional suggestions	"Any person with diabetes who says 'I cannot attend a cake festival because I am afraid of consuming too much sugar' is making a wise decision." (P6).<br><br>"It is very important to establish clear framework conditions before attempting any action." (P4)<br>"When I'm invited, I wash my hair the night before because it is too stressful to do it on the same day. It may be helpful to prepare if you have something planned." (P17)	
Coping, Self-Management		Self-Management of COVID-19	'...facial exercises and massages for your lymph areas and everything and that's been proven to work with chronic fatigue...' (F05; 10 months post-	(Shelley et al., 2021)

216

Top-level Theme	Author generated Theme	Author generated Sub-Themes	Quotes	Citation
& Resilience			infection)<br>'I'm taking some vitamins and I've got a, a sort of over-the-counter antihistamines...' (M13; 11 months post-infection)<br>'...what am I in control of and what am I not in control of' (F01; 7 months post-infection)<br>'...walking with friends so that it takes my mind off what I don't, what I'm so scared about...' (F08; 10 months post-infection)	
Coping, Self-Management & Resilience	Vicious Circle of Fear	• "Snail shell": Avoidance as a consequence of fear<br><br>• "Calculation": Risk assessment instead of fear<br><br>• "Illness of dashed hopes": Self-overload instead of avoidance	"I canceled all the parties and celebrations that were coming up, just out of fear that I<br>wouldn't be able to handle it. I withdraw like a snail and prefer to lie down and sleep." (P19)<br><br>"There is nothing wrong with saying 'I'm not going to go jogging' because I know I<br>am going to crash afterwards. That is protection. Long COVID is not recognized as a life-threatening<br>disease. […] I know that symptoms will occur if I do certain things." (P6)	(Funk et al., 2025)

217

Top-level Theme	Author generated Theme	Author generated Sub-Themes	Quotes	Citation
			"I do not consciously avoid things out of fear, but rather calculate what I am capable of doing." (P8)<br><br>"I was annoyed and said to myself: 'now you're running again'. [...] And then I ended up in the ditch, I was so exhausted." (P2)<br>"On good days, it is satisfying to feel like you are making progress and accomplish a lot. However, it is important not to overextend yourself and cross boundaries. Learning to hold back is crucial, even if you have the desire to do more." (P8)<br>These persistent "setbacks" would lead to a discrepancy between the individual's self-perception and their primary aspirations on the one hand and their actual disability on the other. This discrepancy would have a negative impact on the mood of some patients:<br>"I have always had a positive outlook on everything: 'You can do it'. I would feel disap- pointed if I did not succeed." (P16)<br>"I am very ambitious and performance-oriented. However, there have been	

218

Top-level Theme	Author generated Theme	Author generated Sub-Themes	Quotes	Citation
			times when<br>setbacks have affected my motivation and dragged me down." (P20)	
Coping, Self-Management & Resilience	Behavioral coping strategy	(1) physical activity<br>(2) creative activities<br>(3) recreational activities<br>(4) healthier eating habits<br>(5) volunteering	"Swimming in the pool, like was soothing to me." (ID#4)<br>"Yoga has helped me stay fluid and guided to self-awareness." (ID#1)<br>"I think that volunteering is helping also keep my own anxiety down." (ID#2)	(Aghaei et al., 2022)
Coping, Self-Management & Resilience		- Gradual adaptation<br>- Hope coexisting with uncertainty	"Things will get better in time. There is no doubt about that."<br>"Hopefully, I'm back at work and happy at work…resumed some of the activities that make me happy."<br>"I don't think I will return to the same level…but it's not something my body wants either."	(Loft et al., 2022)
Coping, Self-Management & Resilience	Cognitive and emotional coping strategy	(1) increasing knowledge<br>(2) planning<br>(3) learning new skills<br>(4) realistic goal setting<br>(5) emotions management	"Sometimes the doctors don't know how to make you better or whatever... I've always been more research-based." (ID#8)<br>"I made a list of duties to do one by one." (ID#12)<br>"I try to fix one aspect of the symptoms at a time..." (ID#5)	(Aghaei et al., 2022)

Top-level Theme	Author generated Theme	Author generated Sub-Themes	Quotes	Citation
Coping, Self-Management & Resilience	3. Personal strategies to manage everyday life	Pacing and energy conservation	“I plan my day around when I have energy, and when I know I’ll crash.”	(Leggat et al., 2024)
Coping, Self-Management & Resilience		Self-Management and Communication Strategies	“For me it’s been going from working at 110% pace to not being able to get out of bed... I’m accepting now that I need to take the time off to get better...” (Participant 5)	(Callan et al., 2022)
Coping, Self-Management & Resilience	Hypotheses to Inform Self-Management	Biological and Psychological Explanations	When my GP said ‘we’re treating it like concussion...’ I almost broke down it was the acknowledgement of the issue... takes away so much of the stress...” (Participant 8)	(Callan et al., 2022)
Coping, Self-Management & Resilience	Supervised exercise and pacing improve confidence with exercise		“I would have never pushed myself without knowing it was safe.” “They helped me find that line between doing too much and not enough.”	(Buettikofer et al., 2025)
Coping, Self-Management & Resilience	2. The everyday experience	Impact on daily functioning	“Just going to the supermarket takes all my energy for the day.	(Leggat et al., 2024)

Top-level Theme	Author generated Theme	Author generated Sub-Themes	Quotes	Citation
Coping, Self-Management & Resilience	Practical Resources and Tailoring	"Pacing" and provision of resources: Participants' wishes	"I missed how to communicate changes to your friends, family, or acquaintances. Some<br>people react with understanding while others are defiant and cannot accept that things are<br>changing for them as well." (P5)<br>"It is crucial to find empowering resources to help cope with frustration and strengthen the<br>motivation to deal with the illness." (P8)	(Funk et al., 2025)
Coping, Self-Management & Resilience		Learning to Adapt	'It's like, if you have the energy, you just, you suddenly find yourself doing more, but then you sort of do too much, so the pacing thing really has been quite crucial...' (M13; 11 months post-infection)<br>'It's amazing how much you normalise avoiding stuff that you can't handle' (F05; 10 months post-infection)<br>'I think when you go for weeks and weeks... "Well, is this it now? Is this just me now?"' (F09; 10 months post-infection)	(Shelley et al., 2021)
Coping, Self-Management & Resilience	Recovery and resilience	Caution, adapting, improvement	"She's just careful, trying to be careful."<br>"We continue to wear a mask everywhere… but we don't eat inside restaurants."<br>"I just stayed indoors, like school, I had the option to go to class but I chose to stay home."	(Messiah et al., 2023)
Coping, Self-Management & Resilience		- Acceptance as an active, emotional process<br>- Moving through	"There's a grieving process for our old lives." – P001, woman<br>"Acceptance helped me move forward." – P011, woman	(Kalfas et al., 2024)

Top-level Theme	Author generated Theme	Author generated Sub-Themes	Quotes	Citation
		grief toward adaptation		
Coping, Self-Management & Resilience		Needing flexible pacing and rest	“It was helpful if I knew there was something intense happening that day, that I could follow a day not doing as much.” (P5)	(Boutry et al., 2024)`
Coping, Self-Management & Resilience		Hope in gradual improvement despite uncertainty	"Things will get better in time. There is no doubt about that."	(Burton et al., 2024)
Coping, Self-Management & Resilience	Acceptance	- Letting go of former identity<br>- Learning to adapt or grieve the old self	“We’re giving up our old selves and becoming someone new.” – P001, woman<br>“You have to accept you’re disabled, and that’s hard.” – P012, man	(Kalfas et al., 2024)
Coping, Self-Management & Resilience		Burden of self-advocacy	“I wish I didn’t have to do that myself.”	(Brehon et al., 2023)
Coping, Self-Management & Resilience		Impact of public health restrictions and employer adaptation	“Still wearing masks... not a significant worry for me personally.” (P16)	(Boutry et al., 2024)`
Coping, Self-Management & Resilience	Emotional Resilience	Feeling more protected post-COVID and post-vaccination	“I feel like I’m more protected... had my first vaccination... my body is probably better protected.” (P12)	(Boutry et al., 2024)`

222

Top-level Theme	Author generated Theme	Author generated Sub-Themes	Quotes	Citation
Coping, Self-Management & Resilience	Methods of Coping	(a) Gratitude	“They took such good care of me… God bless those people. [Patient ID 127].” / “I’m very happy that I survived… I’m inspired to keep this momentum moving forward [Patient ID 109].”	(Duan et al., 2023)
Coping, Self-Management & Resilience	Strategies to support LC	Physical health strategies	“…don’t get carried away… be patient… Actually using breathing techniques I think is quite effective… it brought down my heart rate while I was walking.”	(Al-Jabr et al., 2024)
Coping, Self-Management & Resilience		Acceptance and stress management	“…being realistic and lowering expectations… recognising incremental progressions.”	(Al-Jabr et al., 2024)
Coping, Self-Management & Resilience		Self-developed coping strategies	"I tried not to push myself too much… but I needed to challenge my limitations… I tried to go for a few walks every day."	(Burton et al., 2024)
Coping, Self-Management & Resilience	Positive rehab experiences	Speech therapy, pacing education, practical support	“The biggest help was speaking to Speech and Language… lots about the biology of what’s going on with my [laryngeal] spasms.”	(Cooper et al., 2024)
Coping, Self-Management & Resilience	Fatalistic attitude	Resignation to uncertainty	“Some things in life just happen… and that’s it, buonanotte” (A010, male, 71); “If tomorrow they tell me I have to die, well, buonanotte” (A007, male, 70)	(Schiavi et al., 2022)
Coping, Self-Management & Resilience	Returning to (adapted) life course	Reduced functional ability	“Before took me half a day, now it takes me four or five days” (A034, male, 59); “Making breakfast seemed like a challenge” (A026, female, 59)	(Schiavi et al., 2022)
Coping, Self-		Adaptation and	Participants reflected on having passed through a frightening period and	(Schiavi et

223

Top-level Theme	Author generated Theme	Author generated Sub-Themes	Quotes	Citation
Management & Resilience		awareness	now adapt to a “new normal”	al., 2022)
Coping, Self-Management & Resilience		Pacing strategies	“Very tiring week… paced myself each day.”	(Al-Jabr et al., 2024)
Coping, Self-Management & Resilience		Self-management education	"I’m certainly more educated, already daily life has improved..." [P3203]	(Duncan et al., 2023)
Coping, Self-Management & Resilience		Emotional Coping	"Letting go is the biggest art of life. [...] I just let everything go, let it pass me." (P19NR, F)	(Schaap et al., 2022)
Coping, Self-Management & Resilience		Adaptation and Adjustment	“I can’t carry out my daily activities as well, pre-COVID diagnosis.” (N12, F, 30–49)<br>“It is just something that I've had to adjust to.” (N7, F, 18–29, HCW)	(Hitch et al., 2023)
Coping, Self-Management & Resilience	Coping mechanisms	Strategies used by participants	“Exercising, using social media, listening to religious music, watching motivational films, sharing problems, dancing, and reading books were the most effective ways to cope.”	(Silwal et al., 2023)
Coping, Self-Management & Resilience	Need to adapt to a new way of working and living with unpredictabl	Constant cost-benefit analyses in activity planning	"If I go for a walk, I can’t clean the house. If I clean the house, I can’t go out. If I go to the shops, I’ll have to rest the whole next day."	(Skilbeck et al., 2023)

224

Top-level Theme	Author generated Theme	Author generated Sub-Themes	Quotes	Citation
	e energy levels			
Coping, Self-Management & Resilience		Feelings of hopelessness and depression	I'm a lifelong survivor of bipolar disorder... I want to come back, but I can't. It's been three years and I don't see anything concrete..." (Pre-MH)	(Kennelly et al., 2023)
Coping, Self-Management & Resilience	Mental health impact post-COVID	Stress, anxiety, and recurrent thoughts	"Almost 30 percent were intensely worried about monetary reasons because of being jobless… 15 percent experienced recurrent and disturbing thoughts."	(Silwal et al., 2023)
Coping, Self-Management & Resilience		Acceptance of lower work ability	I want so badly to be a fully functioning human being… but yes, I am someone else, a pale copy, a shadow of myself…"	(Gyllensten et al., 2023)
Coping, Self-Management & Resilience	Heightened risk awareness and coping	Fear of severe outcomes, medication adherence	"I kept thinking I was one of the ones that if you got it, it's really not going to look good for you. I've had two heart attacks. I have HIV…viral load is not so great. Yeah, just didn't think it would bode well for me to have the COVID-19… I started to hear on the news something about the HIV medications or – it was good to – you could combat the virus. I don't know. There's something in there, some component. So I thought: Oh, God, you better really take your meds'" (Participant #5)	(Santiago-Rodriguez et al., 2022)
Coping, Self-Management & Resilience		Reluctant acceptance and shifting priorities	"I'm just desperate to get back to exercise but I've had to learn that I can't... a lot of mindfulness and patience..." (IV3)<br>"I might reduce my hours going forward... try and balance my work-life balance... and pace myself." (IV9)	(Humphreys et al., 2021)

225

Top-level Theme	Author generated Theme	Author generated Sub-Themes	Quotes	Citation
Healthcare Navigation, Access & System Gaps	School systems helping and hurting	Mental health support vs. infection risk	"He gets a lot of help at school from his teachers. He got a lot of support with his mental health."<br>"She said 'my teacher was coughing'… she was so scared… she could be in online school it would be better."	(Messiah et al., 2023)
Healthcare Navigation, Access & System Gaps	Perceptions of challenges and barriers to seeking services in the German healthcare system		"With the cardiologist, you call 'come in half a year'… I find that alarming in Germany." (F3, P3)	(Schmachtenberg, Königs, et al., 2023)
Healthcare Navigation, Access & System Gaps		Lack of system navigation support	"It's … not knowing who to call… there is no 1-800-post-COVID helpline"	(Brehon et al., 2023)
Healthcare Navigation, Access & System Gaps	Positive experiences with medical care		"Thank God I have a GP who also has little experience in this area, but who supports me 100%... I'm lucky to have here." (F4, P1)	(Schmachtenberg, Königs, et al., 2023)
Healthcare Navigation, Access &	Lack of insurance coverage	Medication unaffordability	"The medication was about $800… we couldn't afford it."<br>"Each time we take the child to the hospital, the doctor prescribes	(Messiah et al., 2023)

226

Top-level Theme	Author generated Theme	Author generated Sub-Themes	Quotes	Citation
System Gaps	and costly treatments		antibiotics."	
Healthcare Navigation, Access & System Gaps	Lack of clear and consistent PA-related advice	Perceived lack of professional guidance	"The GP... has been trying his best, but he is a little bit unsure himself... most things that have been useful are the support groups on Facebook..." (IV18)<br>"They [physiotherapist] offered loads of advice just about pacing really... people had already been sharing this information." (IV3)	(Humphreys et al., 2021)
Healthcare Navigation, Access & System Gaps	Informal coping mechanisms and social resources	Establishment of new habits affecting mind and body	"I found that my meditation practice has been incredibly helpful... it's okay to stop and I don't have to catch up." (Pre-MH)	(Kennelly et al., 2023)
Healthcare Navigation, Access & System Gaps	Fear of COVID-19 unknowns	Uncertainty and risk aversion	"I wanted to see more in terms of what they were doing to prevent another outbreak… so I changed her to a different school."	(Messiah et al., 2023)
Healthcare Navigation, Access & System Gaps	(In)Ability to Access Care	Identifying need for services	"Even knowing about it. That's the barrier … I had no clue … I never even thought these things existed."	(Brehon et al., 2023)
Healthcare Navigation, Access &	Other sources of support and	Lack of integration across services	"…it's just not making long-term medical conditions management… it's not easy if you don't have the same person [GP], it makes it 10 times harder."	(Al-Jabr et al., 2024)

227

Top-level Theme	Author generated Theme	Author generated Sub-Themes	Quotes	Citation
System Gaps	perceived challenges			
Healthcare Navigation, Access & System Gaps	Accessing care for people with Long COVID	Limited access to GP appointments	"You don't get face-to-face with the GP... it's exhausting telling the same story every single time." [P3208]	(Duncan et al., 2023)
Healthcare Navigation, Access & System Gaps		Emerging effective services	"Occupational therapist through LC clinic really supportive/ helpful."	(Al-Jabr et al., 2024)
Healthcare Navigation, Access & System Gaps	'So Frightening' : Dealing with the Unknown	Perceived Lack of Clinical Support	'Whenever I speak to the [general practitioner] GP, the poor GPs have not been any help at all...' (F08; 10 months post-infection)<br>'I'm very lucky in that the GPs have been understanding...' (F10; 7 months post-infection)<br>'I found it a struggle talking to my doctors, I felt like I wasn't being believed...' (F14; 10 months post-infection)<br>'...after all the blood tests and chest X-ray, is sending me to a post-COVID clinic...' (F14; 10 months post-infection)	(Shelley et al., 2021)
Healthcare Navigation, Access & System Gaps		Public perception that services were closed	"[We] need to get past the public's perception that GPs are shut..." [L3102]	(Duncan et al., 2023)

228

Top-level Theme	Author generated Theme	Author generated Sub-Themes	Quotes	Citation
Healthcare Navigation, Access & System Gaps		Long waiting times	"...the timescales were just probably horrific but understandable." [P3208]	(Duncan et al., 2023)
Healthcare Navigation, Access & System Gaps		Self-referral system issues	"...if I don't have enough information on the referral, they're just going on to the normal routine waiting list." [S2207]	(Duncan et al., 2023)
Healthcare Navigation, Access & System Gaps	different baselines, way markers, and pathways of illness experiences		IE 1. Within half an hour [of phoning for help] two paramedics were in my front room hooking me up […] giving me oxygen, checking my blood pressure. And before I knew it, I was in the back of an ambulance on the way to the hospital. (Rod, ICU)<br>IE 2. They told me I was being taken up to intensive care, because I wasn't breathing well on my own, and I was going to be put on a [CPAP machine.] Yeah, I was scared, obviously. (Beth, ICU)<br>IE 3. At the start [of the pandemic], Long COVID wasn't really a thing. [The media] was just showing the daily deaths, and people that were hospitalised. So […] you were kind of overlooked […] I didn't want to pester the A&E team, and my GP didn't have any answers for me either, so I was […] stuck in a hard place. (Murray, LC)<br>IE 4. I couldn't understand why I wasn't getting better […] there I was months [after initial infection] with evolving symptoms, different systems affected, things that the news [or press conferences]	(MacLean et al., 2025)

229

Top-level Theme	Author generated Theme	Author generated Sub-Themes	Quotes	Citation
			hadn't spoken about […]. It was really confusing, and overwhelming, and worrying. (Stephanie, LC)<br>IE 5. I feel guilty because […] I remember when [my mum] had chemo, her hair…she was so brave. And I think my God, I haven't had all of that [and] I feel traumatised by not having the same hair […] without a valid excuse, if that makes sense? I feel like it's less valid. (Joanna, LC)<br>IE 6. I hadn't been to hospital, I'd never had a definitive [COVID] diagnosis and therefore I […] felt a bit of a fraud; [I would say to myself]"What's all this about? […] It's not like you've been really ill, Jane. People have been really ill." […] I probably made assumptions about how other people were feeling about me [not recovering], which may or may not have been true. (Jane, LC)	
Healthcare Navigation, Access & System Gaps	Resilience resources and coping strategies at the health systems level	1) symptom management<br>(2) medication adherence<br>(3) realistic social responsibilities<br>(4) stress reduction by health care providers	"It helps me to know when it's time to go to the E.R." (ID#7)<br>"When I take medicine, then it helps me work throughout the day." (ID#5)<br>"My counselor really spoke with me about when I get upset at work." (ID#9)	(Aghaei et al., 2022)
Healthcare Navigation,	Understandi ng who	Symptom management	"…went out for a meal with work for a couple of hours—felt good. Got home—symptoms returned intensely."	(Al-Jabr et al., 2024)

230

Top-level Theme	Author generated Theme	Author generated Sub-Themes	Quotes	Citation
Access & System Gaps	helps Long COVID patients	challenges		
Healthcare Navigation, Access & System Gaps		Lack of pathways for complex cases	"But it’s those that have the more complex needs that I wouldn't know where to send them to." [S2103]	(Duncan et al., 2023)
Healthcare Navigation, Access & System Gaps		Appointment and access barriers	“Getting up in the morning is really hard… you’re just more and more tired as you go to the things”	(Brehon et al., 2023)
Healthcare Navigation, Access & System Gaps	Long COVID service limitations	Service disruption due to short-term funding	"...there was no further funding to continue its delivery, the service ceased..."	(Duncan et al., 2023)
Healthcare Navigation, Access & System Gaps	meanings of illness ascribed by others	influence how recovery is experienced		(MacLean et al., 2025)
Healthcare Navigation, Access & System Gaps	Navigating the Healthcare System	Difficulty Communicating Cognitive Symptoms	“Doctors, GP’s... I couldn’t get across the enormity... part of that is because my communication has actually been impaired...” (Participant 5)	(Callan et al., 2022)

231

Top-level Theme	Author generated Theme	Author generated Sub-Themes	Quotes	Citation
Healthcare Navigation, Access & System Gaps		Reluctance to promote under-resourced services	"...not a widely publicised thing, because I don't know if we could cope..." [S1102]	(Duncan et al., 2023)
Healthcare Navigation, Access & System Gaps		Lack of appropriate referral criteria	"...referrals being rejected as inappropriate as PwLC often did not meet pre-existing service criteria..."	(Duncan et al., 2023)
Healthcare Navigation, Access & System Gaps	Strengths and limitations of existing Long COVID services	Blended and flexible delivery	"It has to be a blended model... But digital advances have really made a big difference for us." [L1101]	(Duncan et al., 2023)
Healthcare Navigation, Access & System Gaps	External Systems and Policy	Mixed healthcare experiences	"At work, [long COVID] was accepted; but... [they] said to me, 'how do you know you've got long covid?'" (P9)	(Boutry et al., 2024)`
Healthcare Navigation, Access & System Gaps	Other services augment Long COVID		"The breathing physio was a game changer."<br>"Nutrition advice really helped me with my fatigue."	(Buettikofer et al., 2025)

232

Top-level Theme	Author generated Theme	Author generated Sub-Themes	Quotes	Citation
	recovery			
Healthcare Navigation, Access & System Gaps	Diagnosis facilitates access to support at school		We had to go private, get this diagnosis… just to show school.”	(Faux-Nightingale et al., 2025)
Healthcare Navigation, Access & System Gaps	Blurred boundaries between LC and comorbiditie s	Confusion and clinical uncertainty	“Then you go to your GP and they say Long Covid and then you don’t know what it is.” – Penny (Female, White British, 60s)	(Fang et al., 2024)
Healthcare Navigation, Access & System Gaps		GP referral as a bottleneck	“There is a huge barrier to people even getting an appointment with their GP to get referred.” – Service Manager 1	(Fang et al., 2024)
Healthcare Navigation, Access & System Gaps	A Lonesome Struggle: Managing LTCS Daily	- Lack of professional guidance<br>- Trial-and-error coping<br>- Energy management	“I didn’t quite know how to handle it…The doctor said to manage it like a concussion.”<br>“I really had to cut down drastically…If there was some energy left, then that would be just terrific.”	(Loft et al., 2022)

233

Top-level Theme	Author generated Theme	Author generated Sub-Themes	Quotes	Citation
Healthcare Navigation, Access & System Gaps		- Long wait times and disorganised scheduling	"I booked in December… they changed it to May and didn't tell me." – P5, 40s, Midwest	(Laestadius et al., 2024)
Healthcare Navigation, Access & System Gaps	Clinics are often not a one stop shop	- Disconnected services<br>- Lack of interdisciplinary collaboration<br>- Patient as coordinator	"They didn't interact with each other... I had to bring all my research to people myself… and I'm already tired and sick." – P17, 50s, Pacific	(Laestadius et al., 2024)
Healthcare Navigation, Access & System Gaps	Importance of being in the system	Motivation to persist through hurdles	"Definitely, I mean the support helped me recover." – Patrick (Male, British Indian, 50s	(Fang et al., 2024)
Healthcare Navigation, Access & System Gaps		Burden of self-navigation	"Because we're infamous now, I managed to get… an emergency appointment." – Heather (Female, White British, 60s)	(Fang et al., 2024)
Healthcare Navigation, Access & System Gaps	Complexity in navigating secondary	Specialised but fragmented care	"Nobody's pulling all together and sitting down… there's no conclusion." – Lucy (Female, White British, 50s)	(Fang et al., 2024)

234

Top-level Theme	Author generated Theme	Author generated Sub-Themes	Quotes	Citation
	care			
Healthcare Navigation, Access & System Gaps	Siloed Approach	Post-COVID care processes added on to existing care; multidisciplinary care often disconnected from other services		(O'Hare et al., 2022)
Healthcare Navigation, Access & System Gaps	Monitoring, Diagnostic Testing, and Referral	Use of watchful waiting, repeat imaging, referrals and testing to assess unclear etiology		(O'Hare et al., 2022)
Healthcare Navigation, Access & System Gaps	Reliance on Patient Reports	Clinicians reliant on patients' subjective reports in absence of biomarkers; some clinicians expressed scepticism		(O'Hare et al., 2022)
Healthcare Navigation, Access & System Gaps	Alternative to GP care	Self-management and emergency workarounds	"I've not been back to the GP… the only follow-up appointment I was offered was in hospital." – Sara (Female, British Pakistani, 30s)	(Fang et al., 2024)

235

Top-level Theme	Author generated Theme	Author generated Sub-Themes	Quotes	Citation
Healthcare Navigation, Access & System Gaps	Continued hurdles to accessing primary care	Ongoing access barriers	"You're almost like a leper going to the GP… I just can't see a doctor." – Barry (Male, White British, 60s)	(Fang et al., 2024)
Healthcare Navigation, Access & System Gaps	Appropriate ness	Misdiagnosis; lack of guidelines; absence of interdisciplinary care	"There should be truly interdisciplinary outpatient hospital departments [...] And that's the end of the matter." (E9)	Gamillscheg et al., 2024)
Healthcare Navigation, Access & System Gaps	Encounterin g Medical Complexity	Overlapping symptoms from multiple conditions;	Thisisa56[year-old]MALEwhopresentsfor…evaluationofongoing[shortnessofbreath]and dizziness,post-COVID—[symptom]onset[13moprior],confirmedpositive.Neverhadthese [symptoms]inthepast,onlybeganwithCOVID[diagnosis].Ptnotesdyspneaworsewhiledoing housework,andstateshetriedtowalk1-block,andhadtostopmultipletimes–previouslyveryactive, walking>20kstepsperdayatwork.…Associatedgeneralizedweakness–feelslikeIstruggletoopen bottlesandpickupheavyloadoflaundry,etc.Associateddizzinessdescribedas'roomspinning'after extensivemovement,resolvesspontaneouslyin1-2minutesaftersittingdown."	(O'Hare et al., 2022)
Healthcare Navigation,	Ability to Pay	Recurring specialist visits; statutory fund	"I would really need this [money] right now." (P13)	Gamillscheg et al., 2024)

236

Top-level Theme	Author generated Theme	Author generated Sub-Themes	Quotes	Citation
Access & System Gaps		limitations; patient prioritisation		
Healthcare Navigation, Access & System Gaps	Ability to Reach	Lack of GP guidance; repeated scheduling efforts; transport barriers	"I wrote a specialist an email every fortnight for six months [...] thank goodness he did." (P8)	Gamillscheg et al., 2024)
Healthcare Navigation, Access & System Gaps	Availability and Accommoda tion	Referral criteria; rural scarcity; waiting times; inaccessible facilities	"It's not guaranteed that you'll get an appointment at all. [...] There is an admission freeze." (E9)	Gamillscheg et al., 2024)
Healthcare Navigation, Access & System Gaps	Approachabi lity	Lack of reliable information; GP confusion; peer-led resources	"I found the main difficulty was to know what offers there are, what can I do, what is available at all? [...] There is also a lot of nonsense circulating and you have to be able to differentiate." (P5)	(Gamillscheg et al., 2024)
Healthcare Navigation, Access & System Gaps	Unique challenges to promote LC integrated care pathways	Still limited coverage and visibility	"I haven't heard about a Long COVID clinic… there's nothing." – Malcolm (Male, White British, 70s)	(Fang et al., 2024)
Healthcare	Improving	Knowledgeable	"Nobody really knows right?... I want this outcome right, so I think that	(Brehon et

237

Top-level Theme	Author generated Theme	Author generated Sub-Themes	Quotes	Citation
Navigation, Access & System Gaps	the Quality of Long COVID Services	providers	was hard”	al., 2023)
Healthcare Navigation, Access & System Gaps	A New Beginning: Navigating Hope and Despair on the Journey Towards a New Normal	- Healthcare navigation challenges<br>- Fear of misdiagnosis	“Do you have a tumour in your head or what the hell is this?”<br>“There is no support available…so I feel a little bit on my own.”	(Loft et al., 2022)
Healthcare Navigation, Access & System Gaps	Barriers to accessing Long COVID clinics	- Lack of awareness<br>- No PCP referrals<br>- Inflexible eligibility criteria<br>- Geographic/organisational obstacles	“I’m not sure how me, as a patient, is supposed to know that [hospital] has a doctor who specialises in COVID...” – P30, 30s, South	(Laestadius et al., 2024)
Healthcare Navigation, Access & System Gaps	Theme III: Survivor’s Care-Seeking Behaviour	- Use of biomedical healthcare<br>- Use of spiritual practices (holy water, prayer)	“I used to visit my doctor. He gave me different medicines... He also follows my progress with X-rays…” [50 y/o male]<br>“I pray in the church. I used holy water and eminet... I drink the holy water.” [70 y/o female]<br>“I asked my sister to apply butter to my head when I had a headache…”	(Bogale et al., 2023)

Top-level Theme	Author generated Theme	Author generated Sub-Themes	Quotes	Citation
	for Long COVID-19	- Home remedies (e.g., butter, herbal drinks)<br>- Physical exercise<br>- Avoidance or lack of engagement with care despite severity	[50 y/o male]<br>"I cannot hold my grandkid. However, I did not visit a health facility." [73 y/o male]	
Healthcare Navigation, Access & System Gaps	Importance of GP involvement in connection with clinic participation		"My GP didn't know much about Long COVID until I brought back the clinic notes."<br>"Having the clinic and my GP on the same page gave me confidence in my recovery plan."	(Buettikofer et al., 2025)
Healthcare Navigation, Access & System Gaps	Patient–HCP interactions	3.2.1 Primary care<br>- Importance of continuity and trust<br>- Feeling heard and involved in care decisions<br>- Positive experiences with open-minded GPs	"Having the same doctor was a game changer… he seemed to trust me… referred me to the rapid diagnostics team." (P-5)<br>"My current GP… willing to take advice from me… giving me the off-license medication…" (P-7)	(Turk et al., 2024)
Healthcare	Service	3.3.1 Primary care	It was a three-week wait for a GP appointment, because my GP is quite	(Turk et al.,

Top-level Theme	Author generated Theme	Author generated Sub-Themes	Quotes	Citation
Navigation, Access & System Gaps	resources and structural constraints	- Long wait times for appointments<br>- Format of consultations (in-person vs remote)<br>- Fatigue and cognitive symptoms limiting access	oversubscribed." (P-4)<br>"I don't know how I done that… I drove… shouldn't have driven… because of the brain fog." (P-7)<br>"I would check in with my GP once every two months… telephone appointment." (P-	2024)
Healthcare Navigation, Access & System Gaps		3.3.2 Specialist care<br>- Referral delays and bureaucracy<br>- Lack of communication during wait time<br>- Absence of psychological or self-management support	"It took 14 months to get to see somebody…" (P-2)<br>"My referral went off in January, I didn't hear anything for months…" (P-8)<br>"They couldn't even do the referral to a neurologist… complete waste of many, many months." (P-5)	(Turk et al., 2024)
Healthcare Navigation, Access & System Gaps	Communication and the in-patient experience	Physical barriers to communication	- "Bits flash through my mind, machines and the beeping and that confounded face mask..."<br>- "It was terrible, and if I couldn't see them at night I used to get in a panic...	(Reay et al., 2024)
Healthcare Navigation,		Mixed outcomes of clinical assessment	"No serious illness was found… but that also meant no available treatments."	(Burton et al., 2024)

240

Top-level Theme	Author generated Theme	Author generated Sub-Themes	Quotes	Citation
Access & System Gaps				
Healthcare Navigation, Access & System Gaps	Navigating the healthcare system	Delayed referrals, disbelief, and lack of diagnosis	"It amazes me… that you can keep on having constant headaches… that it can just keep on aching 24–7… I think it's strange."	(Burton et al., 2024)
Healthcare Navigation, Access & System Gaps	Support systems used	Family, school, activities	"…a lot of support from my family and the friends I have…"<br>"He gets a lot of help at school from his teachers… helped him get caught up on grades."<br>"Soccer has been helping him a lot."<br>"I resorted to video games and internet… didn't feel too isolated."	(Messiah et al., 2023)
Healthcare Navigation, Access & System Gaps	Support systems	Clinical support	- "[A GP told me] anyone who had anything in the last two years blames it on COVID... I'm not looking for a magic solution [from GPs]… Just information and maybe a little bit of reassurance." — David<br>- "There was a really big waiting, waiting list… it was just a complete void of what was gonna happen next… So just [needed] some kind of like, really basic information or updates on where you were in that process." — Ellie<br>- "To have someone who is primarily looking after you and analysing everything and that kind of overview... That would be amazing." — Rachel<br>- "It was really difficult to accept my new identity as a sick person... Having counselling really helped me to work through that." — Angela	(Miller et al., 2024)
Healthcare		New clinical tests	- "I think as well, like, more tests, because every test that you do it's fine,	(Miller et al.,

Top-level Theme	Author generated Theme	Author generated Sub-Themes	Quotes	Citation
Navigation, Access & System Gaps			like there's nothing… actually that they can say but then… it just seems like the wrong tests." — Rachel	2024)
Healthcare Navigation, Access & System Gaps	Inaccessible or generic support	Self-help materials felt irrelevant	"I got a booklet... but it's such an individual, highly differentiated set of symptoms... none of it was particularly relevant to me."	(Cooper et al., 2024)
Healthcare Navigation, Access & System Gaps	Fragmented care	Disconnected and impersonal treatment	"All I would do is type in what my symptoms still were and the chemist would deliver drugs. I didn't physically talk to anybody."	(Cooper et al., 2024)
Healthcare Navigation, Access & System Gaps	Exhaustion with the system	Inability to self-advocate due to fatigue	"I didn't have the energy to argue with the receptionist... I just didn't have it in me to phone and try and explain it all again."	(Cooper et al., 2024)
Healthcare Navigation, Access & System Gaps	Taking control of care	Relying on online info and self-research	"I've turned to Twitter... I go back to the GP and ask about stuff, so I'm just having to kind of search for it myself."	(Cooper et al., 2024)
Healthcare Navigation, Access & System Gaps	Access to care and services	- Barriers to care<br>- Lack of referrals	"Covid clinics are not readily accessible to sufferers and, certainly in my experience, long Covid is not something that all GP's are happy to acknowledge, especially without any positive test." (Participant 56)	(Ireson et al., 2022)

242

Top-level Theme	Author generated Theme	Author generated Sub-Themes	Quotes	Citation
Healthcare Navigation, Access & System Gaps	Insecure and fragmented funding	Unsustainable care and staff instability		(Fang et al., 2024)
Healthcare Navigation, Access & System Gaps	Evolving towards holistic care	Demand for a ‘One-Stop-Shop’ model	“It just seems so random, what people are getting and aren’t getting.” – Lucy (Female, White British, 50s)	(Fang et al., 2024)
Healthcare Navigation, Access & System Gaps	Uncertainty About COVID-19	Knowledge	"Even the specialists don’t know that. It could be years before we can say anything definitive." (P18NR, M)	(Schaap et al., 2022)
Healthcare Navigation, Access & System Gaps		Patients informing GPs	“I was relying on my patients… tell me what the Long COVID clinic is like.” – GP 1	(Fang et al., 2024)
Healthcare Navigation, Access & System Gaps	Persistent symptoms	symptoms	“When I take a long walk, I become short of breath” (A072, male, 63)	(Schiavi et al., 2022)
nan				(Kennelly et al., 2023)

243

244

Supplementary File S10. Professional Themes, Subthemes, Quotations and Citations

Top level Theme	Theme	Sub-theme	Quote	Citation
Individualised Management & Symptom Complexity (uncertainty, blurred boundaries)	Theme 1: selecting personalized treatments based on patient presentation and similar conditions amidst uncertainty	Adopting a personalized, symptom-based approach to helping patients manage long COVID symptoms	So individuality is absolutely essential.[…]This is not a cookie-cutter kind of a condition, and therefore care planning can't be cookie cutter. (Participant 1)	(Nguyen et al., 2025)
Individualised Management & Symptom Complexity (uncertainty, blurred boundaries)	Theme 1: selecting personalized treatments based on patient presentation and similar conditions amidst uncertainty	Uncertainty and knowledge gap as consistent challenges in the treatment of long COVID	We're constantly learning and waiting to see if there's anything novel or different. And then, like I said, we are just currently working with what we understand to be the disease. (Participant 2)	(Nguyen et al., 2025)
Individualised Management & Symptom Complexity (uncertainty, blurred boundaries)	Theme 1: selecting personalized treatments based on patient presentation and similar conditions amidst uncertainty	Uncertainty and knowledge gap as consistent challenges in the treatment of long COVID	509 The very nature of the condition itself is extremely challenging.[…]In terms of being able to interpret, as a patient, what I'm capable of doing and what the likely consequences of	(Nguyen et al., 2025)

245

Top level Theme	Theme	Sub-theme	Quote	Citation
			that are going to be on my symptoms is incredibly challenging.[…]It's the most difficult condition I've ever seen from a self-man-agement perspective, because the symptoms appear kind of random or very, very challenging to interpret. (Participant 4)	
Individualised Management & Symptom Complexity (uncertainty, blurred boundaries)	Theme 1: selecting personalized treatments based on patient presentation and similar conditions amidst uncertainty	Uncertainty and knowledge gap as consistent challenges in the treatment of long COVID	On the other side of it, because it's new and emerg-ing, there's no, as far as I'm aware, best practices quite yet developed as to how to properly rehabili-tate somebody with long COVID. The research just hasn't caught up yet. So it can sometimes seem a little bit, you know, tricky or, you know, not as kind of standardized as I'm used to treating other condi-tions. (Participant 5)	(Nguyen et al., 2025)
Individualised Management &	Theme 1: selecting personalized	Uncertainty and knowledge gap as	. And then you feel as a service	(Nguyen et al., 2025)

246

Top level Theme	Theme	Sub-theme	Quote	Citation
Symptom Complexity (uncertainty, blurred boundaries)	treatments based on patient presentation and similar conditions amidst uncertainty	consistent challenges in the treatment of long COVID	provider, I'm not doing enough. And I know that's not on me. It's on what the condition doesn't allow us to know about. So I think that frustration is two-fold. You feel it wholeheart- edly with our patients. But being the service provider to hear all of that, you feel it just the same as well. (Participant 6)	
	Theme 2: building an integrated and evidence-based model of care	Establishing a multidisciplinary team of service providers with care navigation	So I think that an interdisciplinary model without question is the right way to go. The research is there. There's data behind it in terms of improvement of symptom scores, and the functional scores and all that kind of stuff. (Participant 1)	(Nguyen et al., 2025)
	Theme 2: building an integrated and evidence-based model of care	Establishing a multidisciplinary team of service providers with care navigation	Having the complex care people come together, rather than having to send someone to a variety of	(Nguyen et al., 2025)

247

Top level Theme	Theme	Sub-theme	Quote	Citation
			places. So, having it be a more centrally functioning network, so that it's a little bit easier to get things all in one place. (Participant 7)	
	Theme 2: building an integrated and evidence-based model of care	Establishing a multidisciplinary team of service providers with care navigation	Some of the navigation to whether it's things that make life more affordable in various ways, abso- lutely. And navigation, how do we get to and from appointments and things like that. Those can be bar- riers that I think that social workers could absolutely offer a great deal of assistance. (Participant 1)	(Nguyen et al., 2025)
	Theme 2: building an integrated and evidence-based model of care	Improving the continuity and accessibility of services	I think it might be worthwhile to have, I guess, vir- tual follow-ups to see how patients are doing once they're home. And to be able to provide, I guess, virtual counselling, or treatment. Whatever it is that way. Just to make sure that the skills, and the	(Nguyen et al., 2025)

248

Top level Theme	Theme	Sub-theme	Quote	Citation
			strategies that were gained, and were learned in an inpatient setting are still being applied, like, back at home. (Participant 8)	
	Theme 2: building an integrated and evidence-based model of care	Increasing service provider awareness and preparedness to care for long COVID patients	But it's very similar to a concussion. You're not going to see evidence of it, like, in imaging. It doesn't mean that it's not there and that the consequence aren't real. I think with long COVID, there isn't, like, a definitive test for it but it doesn't mean that the experience of it is any less real or that the functional impact is any less. (Participant 9)	(Nguyen et al., 2025)
Self-management, Psychoeducation & Peer Support	Theme 3: providing holistic supports for patients and families through psychoeducation and daily living resources	Educating patients about long COVID symptoms and self-management	It doesn't even have to be like a group program. It could be like a video that they'd be asked to attend. So, some sort of like wholesome like full-some infor- mation on what long COVID	(Nguyen et al., 2025)

249

Top level Theme	Theme	Sub-theme	Quote	Citation
			is, how it can impact you. Like, some sort of resource like that, like a written resource document or something on just like psychoeducation on really what it is and how it can impact you and then with some sort of information where you can go for support. (Participant 13)	
Self-management, Psychoeducation & Peer Support	Theme 3: providing holistic supports for patients and families through psychoeducation and daily living resources	Educating patients about long COVID symptoms and self-management	What we've found very helpful is first establishing good recovery hygiene, is what we call it, or good recovery habits, which is to say, you know, if you're trying to recover from something, you need to have nutrition, sleep, hydration, activity recovery balance. (Participant 4)	(Nguyen et al., 2025)
Self-management, Psychoeducation & Peer Support	Theme 3: providing holistic supports for patients and families through psychoeducation and	Increasing support for maintaining daily living	It increases their fear and anxiety if they can't manage their day-to-day life outside of the hospital. So a lot of funding is required for	(Nguyen et al., 2025)

250

Top level Theme	Theme	Sub-theme	Quote	Citation
	daily living resources		a lot of these patients who have been struggling with long-term COVID. (Participant 2)	
Self-management, Psychoeducation & Peer Support	Theme 3: providing holistic supports for patients and families through psychoeducation and daily living resources	Providing support and family and caregivers	I think it's also very important to involve family sup- port, support services that they have within their family to share and, you know, how to educate them to be able to further support that client. (Participant 14)	(Nguyen et al., 2025)
Mental Health Impact & Supports	Theme 4: caring for mental health in long COVID	Mental health impacts of long COVID and obstacles to emotional recovery	I think you can see it's did a number on some peo- ple. Kind of like low mood, like, sometimes you'll see some of them where they're very flat. They don't want to participate in much and not many things are motivating them. (Participant 15)	(Nguyen et al., 2025)
Mental Health Impact &	Theme 4: caring for mental health in long	Mental health impacts of long COVID and	We've got people who are newly disabled with a	(Nguyen et al., 2025)

251

Top level Theme	Theme	Sub-theme	Quote	Citation
Supports	COVID	obstacles to emotional recovery	chronic illness, not able to fulfill life roles, not able to work, experiencing financial distress. Then we've got them not experiencing support from their fam- ily, friends, and most importantly, their health- care team. So, a lot of them are going through being gaslit about their symptoms, and not having a lot of support. So, that definitely adds to mental health strains. (Participant 10)	
Mental Health Impact & Supports	Theme 4: caring for mental health in long COVID	Enhancing patient mental health with professional support, community building, and provider validation of long COVID	And then, I think access to psychotherapy is always a problem, but I think incredibly useful in helping individuals process and work through their chal- Page 9 of 13 lenges and have actionable strategies that they can implement, in terms of kind of managing symptoms and also increasing resilience	(Nguyen et al., 2025)

252

Top level Theme	Theme	Sub-theme	Quote	Citation
			in coping. (Participant 12)	
Mental Health Impact & Supports	Theme 4: caring for mental health in long COVID	Enhancing patient mental health with professional support, community building, and provider validation of long COVID	Maybe having some meetings where they can go to, like groups. […] Even just not for the long term, but just like for COVID, for people that want to talk about it, because it's a lot to come off your chest sometimes. And like that's going to be the beginning steps to get people back to normalcy, is to be able to talk about it and then realize that they're not the only one. (Participant 16)	(Nguyen et al., 2025)
Mental Health Impact & Supports	Theme 4: caring for mental health in long COVID	Enhancing patient mental health with professional support, community building, and provider validation of long COVID	So I think there's a huge impact on patients, in terms of being able to see themselves in their service pro- viders and to trust their service providers, based on their own personal experiences. I think	(Nguyen et al., 2025)

253

Top level Theme	Theme	Sub-theme	Quote	Citation
			experiences of trauma for example, in the past whether that be due to sexual trauma, or discrimination, or inter-generational trauma– there's lots of reasons I think that impact a patient's trust in healthcare. And so, I think being open to that, understanding that, and accommodating that, is important. (Participant 12)	
Access & Entry to Care (GP gatekeeping, referrals, waiting lists)	continued hurdles to accessing primary care and to entering healthcare systems	Ongoing access barriers	Barry: "You're almost like a leper going to the GP, \njust can't get to the surgery… I just can't see a doctor. \nIt's all done like this. You know, sometimes just done \nover the phone, it's not even a video." (Male, White \nBritish, 60s, phase 1)	Fang et al., 2024
Access & Entry to Care (GP gatekeeping, referrals, waiting	continued hurdles to accessing primary care and to entering	Ongoing access barriers	Susan: "You don't know the fact that maybe you \nhaven't had treatment or things haven't been picked \nup,	Fang et al., 2024

254

Top level Theme	Theme	Sub-theme	Quote	Citation
lists)	healthcare systems		maybe will impact your long-term health because \nyou've been waiting for so long. I think that's also the \nthing, have I missed the time to get some treatment or \nsomething?". (Female, White British, 50s, phase 1)	
Access & Entry to Care (GP gatekeeping, referrals, waiting lists)	continued hurdles to accessing primary care and to entering healthcare systems	Alternative to GP care	Sara: "I went to hospital and had x-ray, blood tests \ndone and they said 'you've got a blood clot, we think \nit's on your lung but we need to book you for a CT \nscan'… [following the scan] I came home and they \ngave me some blood thinning tablets and after about \n10 days I had a CT scan. Whatever it was the blood \nclot was not there anymore. They said that 'there's \nsome damage on the lungs'. So they gave me some \ninhales and I got to go home. After that I wasn't well \nat all… They could clearly see that but what the long	Fang et al., 2024

255

Top level Theme	Theme	Sub-theme	Quote	Citation
			\neffect is we don't know. But I've not been back to the \nGP to find out, the only follow-up appointment I was \noffered was in hospital with the blood clot." (Female, \nBritish Pakistani, 30s, phase 1)	
Access & Entry to Care (GP gatekeeping, referrals, waiting lists)	continued hurdles to accessing primary care and to entering healthcare systems	Alternative to GP care	Luke: "That's what we're waiting on the hospital to do \nfor us. And we haven't had any referrals yet. I don't \nknow what the hold-up is. We've not, we are all geared \nup ready for them, but we haven't had anybody come \nthrough." (Male, White British, 60s, phase 1)	Fang et al., 2024
Access & Entry to Care (GP gatekeeping, referrals, waiting lists)	continued hurdles to accessing primary care and to entering healthcare systems	Increasing awareness of LC	"I've seen patterns I suppose of what people have \nhad. So certainly right at the beginning, it was all the \nlost your taste and smell and you're going to have a \ncough and breathlessness, particularly kind of neuro \ntype symptoms. Those kind	Fang et al., 2024

256

Top level Theme	Theme	Sub-theme	Quote	Citation
			of things I can reassure \npatients that I've seen and that people generally tend \nto get better, so you get a bit more idea of what kind \nof symptoms are coming." (GP 1, phase 2)	
Access & Entry to Care (GP gatekeeping, referrals, waiting lists)	continued hurdles to accessing primary care and to entering healthcare systems	Increasing awareness of LC	Craig: "She took note of it [LC], obviously, we'd had \nit– but didn't prescribe or didn't expand on the con- \nversation. She was just more reactive as opposed to \nproactive on it." (Male, White British, 50s, phase 2)	Fang et al., 2024
Care Coordination & Pathway Integration (multidisciplinary models, navigation)	complexity in navigating secondary care	Specialised but fragmented care	Lucy: "The thing I found the most difficult about the \nhealthcare system is that they don't see things as a \nround so that you know you kind of go and see a few \ndifferent specialists and one's looking at your lungs, \nand one's looking at your head, and one's looking at \nyour heart but nobody's pulling all together and sit-	Fang et al., 2024

257

Top level Theme	Theme	Sub-theme	Quote	Citation
			\nting down and going, this is what we think. There’s \nno, you’re not really sure what the journey is or what \nthey’re trying to find out, what the conclusion is.” \n(Female, White British, 50s, phase 1)	
Care Coordination & Pathway Integration (multidisciplinary models, navigation)	complexity in navigating secondary care	Specialised but fragmented care	“I want to know as a GP that my patients have been \nworked up properly. I have to ask secondary care [for \nupdates] because it’s out of my hands” (phase 1 interview).	Fang et al., 2024
Care Coordination & Pathway Integration (multidisciplinary models, navigation)	complexity in navigating secondary care	Specialised but fragmented care	“infamous”	Fang et al., 2024
Care Coordination & Pathway Integration	complexity in navigating secondary care	Specialised but fragmented care	Heather: “I phoned [hospital A] the next day \nbecause although it’s another hospital called [hos- \npital B] in	Fang et al., 2024

258

Top level Theme	Theme	Sub-theme	Quote	Citation
(multidisciplinary models, navigation)			[location C], it was through [hospital \nA] that I had to try and weave my way through. \nAnd thankfully, I'd taken so many phone numbers \nwhen my husband was at [hospital A], I man- \naged to go through to a specialist and say, 'I can't \nget through to your booking clerk, but I need to \ndo this', and because we're infamous now I think, \n[laughs], I managed to get – she got me an emer- \ngency appointment for the afternoon." (Female, \nWhite British, 60s, phase 1)	
Care Coordination & Pathway Integration (multidisciplinary models, navigation)	complexity in navigating secondary care	Specialised but fragmented care	"slightly better but \nnot significantly"	Fang et al., 2024
Care Coordination & Pathway	complexity in navigating secondary care	Specialised but fragmented care	"they [healthcare professionals, such as \nspecialists, paramedics and	Fang et al., 2024

259

Top level Theme	Theme	Sub-theme	Quote	Citation
Integration (multidisciplinary models, navigation)			receptionists] seemed to be \nlike cogs working in different directions”	
Care Coordination & Pathway Integration (multidisciplinary models, navigation)	complexity in navigating secondary care	Importance of being in the system	“They [the hospital] were very supportive \n– after I left hospital there was in total about seven \nor eight months of follow-up support. I had the gen- \neral nurse, the general consultant because they were \nworried about strokes and they were worried about \ndifferent things, neurosurgeon, a lot of different peo- \nple doing bloods. So I had to have the different peo- \nple signed off and occupational psychiatry had to \nsign me off etc. So bit by bit they all signed me off \nuntil I got out and then even thereafter each func- \ntion needed to monitor you afterwards. So I had a \nlung specialist because my lung was damaged. I had \na	Fang et al., 2024

260

Top level Theme	Theme	Sub-theme	Quote	Citation
			haematologist, so different functions… Definitely, I \nmean the support helped me recover.” (Male, British \nIndian, 50s, phase 1).	
Care Coordination & Pathway Integration (multidisciplinary models, navigation)	complexity in navigating secondary care	Importance of being in the system	“healthy enough”	Fang et al., 2024
Care Coordination & Pathway Integration (multidisciplinary models, navigation)	complexity in navigating secondary care	Importance of being in the system	“Long COVID to be logged in the NHS”	Fang et al., 2024
Care Coordination & Pathway Integration (multidisciplinary models, navigation)	complexity in navigating secondary care	Increased challenges to address blurred boundaries between LC and other health conditions	“Obviously, symptoms are still coming up and changing and sometimes new \nsymptoms are coming along that weren’t there a year ago. That’s why you want to \nget seen, so if they start to see	Fang et al., 2024

261

Top level Theme	Theme	Sub-theme	Quote	Citation
			something worrying or something that needs \nattention, it's your body. At the moment, I don't feel I'm on a list anywhere of \npeople suffering from this."	
Care Coordination & Pathway Integration (multidisciplinary models, navigation)	complexity in navigating secondary care	Increased challenges to address blurred boundaries between LC and other health conditions	"We've realised that women who are \nof menopausal age have been affected more by post COVID as well. We've done \nsome training sessions on the menopause and post COVID to try and address that, \nmaking people more aware."	Fang et al., 2024
Care Coordination & Pathway Integration (multidisciplinary models, navigation)	complexity in navigating secondary care	Increased challenges to address blurred boundaries between LC and other health conditions	"The thing [joint pain] with Covid is \nbasically I don't know what I'm supposed to be look- \nning for and nobody's actually said anything, so I get \nconfused sometimes when I feel ill, I don't think of \nLong Covid and just think, oh maybe I've got a chest \ninfection or something else. Then you go to your GP \nand they say	Fang et al., 2024

262

Top level Theme	Theme	Sub-theme	Quote	Citation
			Long Covid and then you don't know \nwhat it is."	
Care Coordination & Pathway Integration (multidisciplinary models, navigation)	complexity in navigating secondary care	Increased challenges to address blurred boundaries between LC and other health conditions	"this is the final curtain, this is the \nlast, 'cos you think, well this isn't getting any better, I don't \nknow what this is"	Fang et al., 2024
Care Coordination & Pathway Integration (multidisciplinary models, navigation)	unique challenges to promote LC integrated care pathways	Still limited coverage	Malcolm: "I haven't heard about a Long COVID \nclinic or something like that in this area if there \nwas something I think she would have said, or if you \ncould see, the nurse would have said, do you want to \ngo to a clinic? But there's nothing." (Male, white Brit- \nnish, 70s, phase 2).	Fang et al., 2024
Care Coordination & Pathway Integration (multidisciplinary models,	unique challenges to promote LC integrated care pathways	Still limited coverage	"I was relying on my patients, I was like, tell me what \nthe Long COVID clinic is like and come back to me. \nAnd then I can tell the next person". (GP 1, phase 1)	Fang et al., 2024

263

Top level Theme	Theme	Sub-theme	Quote	Citation
navigation)				
Care Coordination & Pathway Integration (multidisciplinary models, navigation)	unique challenges to promote LC integrated care pathways	Tendency to develop a pathway towards holistic and consistent care	"One-Stop-Shop"	Fang et al., 2024
Care Coordination & Pathway Integration (multidisciplinary models, navigation)	unique challenges to promote LC integrated care pathways	Tendency to develop a pathway towards holistic and consistent care	"A one-stop-shop where you can, say, 'this is Long \nCOVID, this is what people have experienced, this \nis what doctors can do, this is what they can't do, \nthis is what people have found helpful, this is what's \navailable' would be really helpful. It just seems so \nrandom, what people are getting and aren't getting." \n(Female, White British, 50s, phase 1)	Fang et al., 2024
Care Coordination & Pathway Integration	unique challenges to promote LC integrated care pathways	Patient-Initiated Follow-Ups	"What we're just at the point of starting to offer are \nthe Patient Initiated Follow-Ups, so once they've \nbeen	Fang et al., 2024

264

Top level Theme	Theme	Sub-theme	Quote	Citation
(multidisciplinary models, navigation)			through the groups that we feel have been \nappropriate and they’ve gone through them all \nonce, it’s then about some sort of self-management \nand then offering them to see how you go on. If \nyou feel you need to come back to us, you initiate \nanother follow-up with us. But as I say, we’ve still \ngot patients working through and we are not quite \nyet, but that’s our intention.” (Rehabilitation coor- \ndinator 1, phase 2)	
Care Coordination & Pathway Integration (multidisciplinary models, navigation)	unique challenges to promote LC integrated care pathways	Uncertainties in funding security	“All these patients, that we’ve just talked about end- \ning up within the community and getting referred \nback in, they’re not funded. We haven’t got any fund- \ning for increased activity related to Long COVID. \nWe’ve got at the moment recovery money which \nmeans we can put on extra activity and have	Fang et al., 2024

265

Top level Theme	Theme	Sub-theme	Quote	Citation
			extra \nsessions for the consultants that can be funded. But \nlong term it’s very difficult to predict how long this \nis going to go on for. We keep stopping and starting \nactivity depending on spikes, so we don’t know what \nour backlog is going to look like and we don’t know \nwhat the long-term implications are going to be.” \n(Specialised medicine manager, phase 1)	
Care Coordination & Pathway Integration (multidisciplinary models, navigation)	unique challenges to promote LC integrated care pathways	Uncertainties in funding security	“It’s much harder to recruit to temporary contracts \nand secondment, it’s destabilising for the service \nwhere you take the person from because it’s really \nhard for them to fill that vacancy… we are three \nseparate Trusts working together and it’s quite \ncomplicated then because each Trust has their \nown policies and procedures that aren’t	Fang et al., 2024

266

Top level Theme	Theme	Sub-theme	Quote	Citation
			necessar- \nily the same. Everyone still has a contract with \none Trust and just follow those procedures from \ntheir Trust which might be different from their col- \nleagues' procedures. So those things are quite chal- \nlenging." (Service manager 1, phase 2)	
Care Coordination & Pathway Integration (multidisciplinary models, navigation)	unique challenges to promote LC integrated care pathways	Uncertainties in funding security	"being turned away"	Fang et al., 2024
Care Coordination & Pathway Integration (multidisciplinary models, navigation)	unique challenges to promote LC integrated care pathways	Uncertainties in funding security	"being aban- \ndoned"	Fang et al., 2024
Care Coordination &	unique challenges to promote LC	Uncertainties in funding security	"Reluctant pioneer"	Fang et al., 2024

267

Top level Theme	Theme	Sub-theme	Quote	Citation
Pathway Integration (multidisciplinary models, navigation)	integrated care pathways			
Care Coordination & Pathway Integration (multidisciplinary models, navigation)	unique challenges to promote LC integrated care pathways	Uncertainties in funding security	“right”	Fang et al., 2024
Care Coordination & Pathway Integration (multidisciplinary models, navigation)	unique challenges to promote LC integrated care pathways	Uncertainties in funding security	“long Covid”	Fang et al., 2024
	Themes	Subthemes	Quotes	Citation
Individualised Management & Symptom Complexity (uncertainty, blurred	Selecting personalized treatments based on patient presentation and similar conditions amidst	Adopting a personalized, symptom-based approach to helping patients manage long COVID symptoms<br>Uncertainty and knowledge gap as	"So individuality is absolutely essential.[...] This is not a cookie-cutter kind of a condition, and therefore care planning can't be cookie cutter." <br>"We're	Nguyen et al., 2025

268

Top level Theme	Theme	Sub-theme	Quote	Citation
boundaries)	uncertainty	consistent challenges in the treatment of long COVID	constantly learning and waiting to see if there's anything novel or different. And then, like I said, we are just currently working with what we understand to be the disease." <br>"You start with pacing. You have to do pacing. If you can't do pacing, nothing else will work. Because that's the bread that's the basics the bread and butter of long COVID is that fatigue management. If you cannot manage fatigue, you can't predict, you can't function, and so it starts with the pacing." <br>"The very nature of the condition itself is extremely challenging.[...]It's the most difficult condition I've ever seen from a self-management perspective, because the symptoms appear kind of random or very, very challenging to interpret." <br>"On the other side	

269

Top level Theme	Theme	Sub-theme	Quote	Citation
			of it, because it's new and emerging, there's no, as far as I'm aware, best practices quite yet developed as to how to properly rehabilitate somebody with long COVID. The research just hasn't caught up yet." <br>"And then you feel as a service provider, I'm not doing enough... So I think that frustration is two-fold. You feel it wholeheartedly with our patients. But being the service provider to hear all of that, you feel it just the same as well."	
	Building an integrated and evidence-based model of care	Establishing a multidisciplinary team of service providers with care navigation Improving the continuity and accessibility of services Increasing service provider awareness and preparedness to care for	"So I think that an interdisciplinary model without question is the right way to go. The research is there. There's data behind it in terms of improvement of symptom scores, and the functional scores and all that kind of stuff." <br>"Having the complex care	Nguyen et al., 2025

270

Top level Theme	Theme	Sub-theme	Quote	Citation
		long COVID patients	people come together, rather than having to send someone to a variety of places. So, having it be a more centrally functioning network, so that it's a little bit easier to get things all in one place." <br>"Some of the navigation to whether it's things that make life more affordable in various ways, absolutely. And navigation, how do we get to and from appointments and things like that. Those can be barriers that I think that social workers could absolutely offer a great deal of assistance." <br>"I think it might be worthwhile to have, I guess, virtual follow-ups to see how patients are doing once they're home. And to be able to provide, I guess, virtual counselling, or treatment... Just to make sure that the skills, and the strategies that	

271

Top level Theme	Theme	Sub-theme	Quote	Citation
			were gained, and were learned in an inpatient setting are still being applied, like, back at home." <br>"But it's very similar to a concussion. You're not going to see evidence of it, like, in imaging. It doesn't mean that it's not there and that the consequence aren't real. I think with long COVID, there isn't, like, a definitive test for it but it doesn't mean that the experience of it is any less real or that the functional impact is any less." <br>"What I always emphasize is just the importance of people in the health field, getting knowledgeable and looking at what they're dealing with. There's something real happening here, just because we can't see it with our regular tests, doesn't mean it's not happening. It's just our tests aren't very good." <br>"I think definitely	

272

Top level Theme	Theme	Sub-theme	Quote	Citation
			dissemination of best practice like development and then dissemination to primary care providers and perhaps to emergency departments... because then it would not only legitimize the diagnosis, so patients could then go to a provider and not feel as dismissed or invalidated." <br>"I think this actual clinic is very- there isn't necessarily longevity in it. We don't know whether or not this program is going to be here in six months in a year... that is really hard to do." <br>"I think it also is emotionally draining and can touch close to home... for anyone who's seeing mental health patients, to ensure that they have their own coping strategies and support."	
Self-management, Psychoeducation & Peer Support	Providing holistic supports for patients and families through	Educating patients about long COVID symptoms and self-management 	"It doesn't even have to be like a group program. It could be like a video that they'd be	

273

Top level Theme	Theme	Sub-theme	Quote	Citation
	psychoeducation and daily living resources	Increasing support for maintaining daily living Providing support and family and caregivers	asked to attend... So, some sort of like wholesome like full-some information on what long COVID is, how it can impact you... [or] a written resource document or something on just like psychoeducation on really what it is and how it can impact you and then with some sort of information where you can go for support." <br>"What we've found very helpful is first establishing good recovery hygiene, is what we call it, or good recovery habits, which is to say, you know, if you're trying to recover from something, you need to have nutrition, sleep, hydration, activity recovery balance." <br>"It increases their fear and anxiety if they can't manage their day-to-day life outside of the hospital. So a lot of funding is required for a lot of	

274

Top level Theme	Theme	Sub-theme	Quote	Citation
			these patients who have been struggling with long-term COVID."	
Care Coordination & Pathway Integration (multidisciplinary models, navigation)	Patients’ efforts to navigate emerging pathways for Long Covid	Online resources and support groups	“No formal advice on it at all. It's mostly been me [online group] that's helped and like resources around that about pacing and how to actually do that.” (P-4)	Turk et al., 2024
			“Because I think it's hard to try and understand this condition unless you've got it or unless you work in healthcare and you see … in healthcare and you see it every day so … yeah being able to connect with other people that have Long Covid and just talk out, you know vent and rant and you know talk out what we feel, that has been really great and yeah it would have been nice to connect with people earlier.” (P-3)	Turk et al., 2024

275

Top level Theme	Theme	Sub-theme	Quote	Citation
			"I think them hearing other people and talking to other people who have had similar experiences so being able to hear other people's stories will be helpful, so that peer support." (HCP-6, specialist)	Turk et al., 2024
			"Compounded by the confusion which is generated by people engaging with dubious healthcare sources online and so much time is spent sort of navigating erroneous opinions, bizarre theories with patients…" (HCP-8, secondary care)	Turk et al., 2024
Care Coordination & Pathway Integration (multidisciplinary models, navigation)	Patients' efforts to navigate emerging pathways for Long Covid	Primary care	"It was probably about 11 months before somebody actually recognised, it was long-Covid, which was a locum GP who is standing in for my regular GP at the time. And who is far more knowledgeable about long-Covid, she'd been to a few conferences about it and just immediately said 'yep, that's	Turk et al., 2024

276

Top level Theme	Theme	Sub-theme	Quote	Citation
			long-Covid'." (P-7)	
			"I had to really push and say, can you please refer me to a Long Covid clinic." (P-4)	Turk et al., 2024
			"…but I just got the impression that my GPs, were just constantly overwhelmed and so I was the one that had to initiate lots of things I had to push for the blood tests and push for you do I need to chest X-Ray and I was emailing my GP with you know the questionnaires, the list of things they should include on the referral." (P-4)	Turk et al., 2024
			"Obviously we only have 10 minutes. There's (quite limits), a limit, to what we can do in 10 minutes." (HCP-4, GP)	Turk et al., 2024
			"I can't chase that, so you know it's up to them, you know the ones with more severe symptoms, I have two particular ones in my mind at	Turk et al., 2024

277

Top level Theme	Theme	Sub-theme	Quote	Citation
			the moment I've seen multiple times. Yeah, and they'll come back for review and will initiate some antihistamines or … you know… famotidine and then they come back, and we'll review that and give them more information, bit by bit…" (HCP-4, GP)	
Care Coordination & Pathway Integration (multidisciplinary models, navigation)	Patients' efforts to navigate emerging pathways for Long Covid	Specialist care	"Because it was me pushing I really feel as if me sending letters to people and pushing and chasing and making sure things happened I, the only reason I had the first MRI scan was because I kept chasing … after the Long Covid clinic letter I asked for that to be done, I had to ask again for it to be done, and then I was told that it would be done." (P-6)	Turk et al., 2024
	The interaction between patients and HCPs	Primary care	"I think having the same doctor was a game changer for me. Just having that one person who was able to sort of remember what I had said	Turk et al., 2024

278

Top level Theme	Theme	Sub-theme	Quote	Citation
			before, was able to read his own notes. Adding that for me was so key. He's also really good. He seems to trust me when I bring him a problem … He referred me to the rapid diagnostics team where that happened very quickly…" (P-5)	
			"…You know we'll sort of explore with them and give them an opportunity really to share their sort of concerns and where they're up to really and then depending on what happens then between the patient and us, we will then decide to look, you know what do you want to do next, what do you think you need? This is what we can offer. We think this will be good but it's up to you…" (HCP-3, GP)	Turk et al., 2024
			"…was my current GP who has been open-minded willing to take advice from me… and trying to make the rules work	Turk et al., 2024

279

Top level Theme	Theme	Sub-theme	Quote	Citation
			for the patients, as in giving me the off-license medication, even though the budgets not there.” (P-7)	
	The interaction between patients and HCPs	Specialist care	“I went in they kind of checked everything I think it was the first time when a doctor sat with me for like 45 minutes and talked about my entire medical history every small, tiny little … And I think it's just it felt important to like to take a person and say ‘OK we're concerned, for your health. We're going to check you out we don't want you to be sick or die, we don't want to miss anything’ so that was that was really good.” (P-4)	Turk et al., 2024
			“Listening to the patient is really important, that validation of their story and them being able to hear other people's stories and know that they're not the only person having the same, feeling the	Turk et al., 2024

280

Top level Theme	Theme	Sub-theme	Quote	Citation
			same way and have a same set of signs and symptoms, having access to somebody who can support them. And then having confidence so that they learn how to manage that, over time, so having that confidence and being able to manage their return to activity and knowing how to react to symptoms, so if they're feeling more unwell what to do about that, and how to know to progress so actually being able to have that self-mastery of their condition going forward would be what I think we're really aiming for…" (HCP-6, specialist)	
			"We have some touch points so we manage, try and talk about expectations of how long we might have people in care for. So, we we'd talk about it being about three months, but most of our patients don't end up being	Turk et al., 2024

281

Top level Theme	Theme	Sub-theme	Quote	Citation
			with us just for three months, but for longer. But we're trying to manage the fact that people aren't going to stay with us forever and necessarily aren't going to stay with us until they're 100% better.” (HCP-6, specialist)	
Resource, Capacity & Sustainability Constraints (funding, staffing)	Service resources and structural constraints	Primary care	“I made a GP appointment again, and it was a three week wait for a GP appointment, because my GP is quite over-subscribed.” (P-4)	Turk et al., 2024
			“They were initially remote. But I'm now traveling to appointments … before I moved. After the first lockdown I had to go to a GP appointment in person, I also to go for blood tests in person that I don't know how I done that, I honestly don't I drove. Shouldn't have driven because back then, I mean I was definitely dangerous to	Turk et al., 2024

282

Top level Theme	Theme	Sub-theme	Quote	Citation
			drive, but I did, and because of the brain fog." (P-7)	
			"I would check in with my GP once every two months to give him an update or something specific like when the digestive problems kicked in, call the GP practice, get a telephone appointment. He'd prescribe something like symptom control. I've had blood tests every six months, so you know, keep an eye out…" (P-5)	Turk et al., 2024
Resource, Capacity & Sustainability Constraints (funding, staffing)	Service resources and structural constraints	Specialist care	"Obviously knowing some people will be digitally challenged, don't have the technology uhm so we're hopefully mopping up everything. So, we can see people in the home or, we do that, or we might do a one to one remotely via video or telephone or we might bring them into an on-site and see them maybe more you know in in a clinic type situation so,	Turk et al., 2024

283

Top level Theme	Theme	Sub-theme	Quote	Citation
			yeah so a very blended approach.” (HCP-6, specialist)	
			“A single point of access now where all of those come through to us. We triage them on the basis of the self-assessment questionnaire, which is done through an online portal and a phone conversation with our nurse navigator. Trying to refer people direct to post COVID rehab if that's safe but finding that more than two thirds of people need some sort of medically overseen assessment, because the patients that are getting referred are quite poorly.” (HCP-5, secondary care)	Turk et al., 2024
			“I had asked for referral by this stage to see a respiratory consultant, you know, and which I repeatedly chased and chased and chased and it took 14 months to get to see	Turk et al., 2024

284

Top level Theme	Theme	Sub-theme	Quote	Citation
			somebody you know…" (P-2)	
			"We still have 200 people waiting … so that's a capacity issue." (HCP-2, secondary care)	Turk et al., 2024
			"Our waiting lists are down. So that's good we interact early on, with patients on the waiting list so we sign post them to self-help information so patients can start looking at stuff and thinking about stuff they potentially can take up the [online therapy], or the [digital physical therapy] and so, although they are waiting a couple of months to see us, there are things that they can start doing if they want to so they're not just sitting there with nothing happening." (HCP-3, GP)	Turk et al., 2024
			"It's just pointless, you know you can't leave somebody with Long Covid for six months without support, so	Turk et al., 2024

285

Top level Theme	Theme	Sub-theme	Quote	Citation
			we try to give them a triage call to you know put them in touch with some self-management advice whilst they're waiting their appointment.” (HCP-5, secondary care)	
			“Even if it was just a message saying your still on our list, don't worry we'll be in touch just so that you know. Because you know my referral went off in January, I didn't hear anything for months…” (P-8)	Turk et al., 2024
			“They couldn't even do the referral to a neurologist I have neurological symptoms, I clearly need to see a neurologist … but I wasn't really allowed to make an application sorry the GP wasn't allowed to do referral until we've been through Long Covid clinic. The Long Covid clinic couldn't even do the referral themselves all	Turk et al., 2024

286

Top level Theme	Theme	Sub-theme	Quote	Citation
			they did was write back to my GP to say make a referral to a neurologist, and so it was just a complete waste of many, many months." (P-5)	
			"We're good at communicating … That's what we always do, and that's we have a patient, and if we need extra help we do talk to the people that we need help from so, it's not really anything new to us to do that, and so, and then, if we go in and somebody and think actually this is less Long covid and more their COPD, then we'd reach out to the COPD team and potentially hand that over and make that referral and ask them to be carried forward through the COPD team, so that that's how it's been managed at the moment." (HCP-6, specialist)	Turk et al., 2024
	Theme	Subtheme	Quote & Citation	Source

287

Top level Theme	Theme	Sub-theme	Quote	Citation
	The lived experience of LC		"I was desperate, I just had no quality of life at all. I couldn't speak to my friends for coughing, couldn't look after my family because I had no energy, couldn't get out the house, I was housebound for months. It was just rubbish." [APwLC05]	Cooper et al., 2024
	The lived experience of LC		"Fortunately, I've had it, been off, but then went back to work. I've not stayed off. Maybe that's a problem as well, I've not stayed off long term and listened to my body. I've went back and really threw myself back into my workplace and then suffered in my days off." [CPwLC01]	Cooper et al., 2024
Individualised Management & Symptom Complexity (uncertainty, blurred boundaries)	The challenges of an emergent and complex chronic condition		"I don't know if we will move away from handing out the diagnosis of Long COVID. Because to be honest, I don't often suggest it to the patient as a diagnosis because our options for management are so minimal.	Cooper et al., 2024

288

Top level Theme	Theme	Sub-theme	Quote	Citation
			So, I tend to, if the patient thinks they've got it, work through that with them." [AGP01]	
Individualised Management & Symptom Complexity (uncertainty, blurred boundaries)	The challenges of an emergent and complex chronic condition		"I think we are talking about Long COVID now because everyone is looking at COVID, which is great. But I think it's not the only kind of post-[illness] treatment type problem that we have nowhere to send people. And I think it's a problem with the health system." [BGP01]	Cooper et al., 2024
Individualised Management & Symptom Complexity (uncertainty, blurred boundaries)	The challenges of an emergent and complex chronic condition		"There's definitely been a lot more media which is very helpful…there was a middle part where people were quite scathing because in the first part I remember friends saying 'I don't know anybody else that's got Long COVID', and that in itself felt a judgement, but then we hit a middle part where loads of folk were getting COVID but they'd been vaccinated or	Cooper et al., 2024

289

Top level Theme	Theme	Sub-theme	Quote	Citation
			their bodies just dealt with it differently and they weren't ill, so then there was a huge period of judgement came out, 'well, so- and- so had it and they're fine'." [BPwLC06]	
Individualised Management & Symptom Complexity (uncertainty, blurred boundaries)	The challenges of an emergent and complex chronic condition		"Oh, I've turned to Twitter…there is kind of a few people on Twitter that I follow that have been really good and kind of published research papers…I go back to the GP and ask about stuff, so I'm just having to kind of search for it myself…So you're not only having to deal with the illness you have to kind of then navigate kind of like, where am I going to get help from?" [DPwLC02]	Cooper et al., 2024
Resource, Capacity & Sustainability Constraints (funding, staffing)	Systemic challenges for LC service delivery		"I feel that if there'd been a more joined up approach, somebody would have been like 'wait a minute, she can't be on that for four months on that dose' … because my	Cooper et al., 2024

290

Top level Theme	Theme	Sub-theme	Quote	Citation
			doctor at the time had a consult thing online. So all I would do is type in what my symptoms still were and the chemist would deliver drugs. I didn't physically talk to anybody. So, that was obviously a huge failing, and I think doctors really realised that should never have happened but they had such shortages and still have such shortages" [BPwLC05]	
Resource, Capacity & Sustainability Constraints (funding, staffing)	Systemic challenges for LC service delivery		"There is a risk that we hold up rehabilitative inputs until we fully investigated things and we are entirely assured that there's nothing going on. So, we should perhaps be blending things a little better." [BGP02]	Cooper et al., 2024
Resource, Capacity & Sustainability Constraints (funding, staffing)	Systemic challenges for LC service delivery		"I didn't have the energy to argue with the receptionist at the GP surgery, and that's the honest truth, I just didn't have it in me to phone and try and explain it all again. So, I lost	Cooper et al., 2024

291

Top level Theme	Theme	Sub-theme	Quote	Citation
			nineteen pounds in four weeks because I just couldn't eat because I felt sick. But even that wasn't worth the battle I would have to get past a GP receptionist." [APwLC05]	
Resource, Capacity & Sustainability Constraints (funding, staffing)	Systemic challenges for LC service delivery		"We were getting a bit frustrated referring patients to secondary care for help. There wasn't much coming through. They were already dealing with backlog enough and they're getting piled up with these other things happening. I understand their limitations, entirely…I don't think that any I have referred [to respiratory or cardiovascular] have been seen yet. …" [CGP03]	Cooper et al., 2024
Resource, Capacity & Sustainability Constraints (funding, staffing)	Systemic challenges for LC service delivery		"I just feel sometimes having that one person, like I know a lot of people have a consultant that they go to and that's the person that they speak to, or the centre that	Cooper et al., 2024

292

Top level Theme	Theme	Sub-theme	Quote	Citation
			they go to, for support. There’s a group of people perhaps that they deal with, but they get it. They're a familiar face and that makes sense. But you feel a bit of a pariah, to be honest, with Long COVID.” [BPwLC06]	
Resource, Capacity & Sustainability Constraints (funding, staffing)	Systemic challenges for LC service delivery		“If there was a secondary care service set up to see a certain number of patients per day then presumably they would probably allow further, probably allow longer appointments and would be more of a multi-disciplinary approach so that patients would have a bit more time to kind of unpick everything that’s going on because there definitely is, probably a kind of you know, whole sort of biopsychosocial thing going on here” [AGP02]	Cooper et al., 2024
Recognition, Validation & Knowledge	Perceptions and experiences of LC and its management,		“Maybe 10–12 [Long COVID patients have presented] in total. But I don’t know	Cooper et al., 2024

293

Top level Theme	Theme	Sub-theme	Quote	Citation
(awareness, legitimacy, expectations)	including rehabilitation		whether they're all coming to us. They might be just suffering in silence." [CGP03]	
Recognition, Validation & Knowledge (awareness, legitimacy, expectations)	Perceptions and experiences of LC and its management, including rehabilitation		"Most people are pretty sensible and they know, they are educated and I am finding that patients that are coming with long covid symptoms, or being quite certain that they have long covid they have educated themselves about it so, quite often actually they would come and they would sometimes know much more than the doctor about it because they have done their own research about it and they will likely know that there is nothing else" [BGP05]	Cooper et al., 2024
Recognition, Validation & Knowledge (awareness, legitimacy, expectations)	Perceptions and experiences of LC and its management, including rehabilitation		"A lot of patients don't necessarily consult because probably they are seeing things in the media and things, you know aware that there aren't particular	Cooper et al., 2024

294

Top level Theme	Theme	Sub-theme	Quote	Citation
			treatments. So, they just think it's par for the course that they feel like that." [AGP02]	
Recognition, Validation & Knowledge (awareness, legitimacy, expectations)	Perceptions and experiences of LC and its management, including rehabilitation		"I stopped contacting the GP because I just feel I'm wasting their time" [APwLC03]	Cooper et al., 2024
Recognition, Validation & Knowledge (awareness, legitimacy, expectations)	Perceptions and experiences of LC and its management, including rehabilitation		"I know there are things that I haven't raised with a GP because I'm aware that they're time pressured, I've raised about ten symptoms already in my consultation with them and I know I've got another three sitting on my list, but I can't bring that into the situation… and I'm potentially sitting on stuff that I should have discussed" [BPwLC06]	Cooper et al., 2024
Recognition, Validation & Knowledge (awareness,	Perceptions and experiences of LC and its management, including		"But the last time I went to the GP they said, 'we find people with Long COVID know more about it than we	Cooper et al., 2024

295

Top level Theme	Theme	Sub-theme	Quote	Citation
legitimacy, expectations)	rehabilitation		do.' And I thought that doesn't really fill you with great enthusiasm." [DPwLC04]	
Recognition, Validation & Knowledge (awareness, legitimacy, expectations)	Perceptions and experiences of LC and its management, including rehabilitation		"It's just reassuring to know that you're not alone in this. Misery loves company, and it's good to know that there are other people who have this, because otherwise it would become kind of depressing. And it helps put things in perspective, that you know that as bad as you feel someone else is probably feeling worse." [DPwLC06]	Cooper et al., 2024
Recognition, Validation & Knowledge (awareness, legitimacy, expectations)	Perceptions and experiences of LC and its management, including rehabilitation		"It's not only for people, it's also for the medical professionals to believe as well that this is a problem. I think there is still some scepticism among medical professionals as well, still, about this being accepted and treated." [CGP03]	Cooper et al., 2024
Recognition,	Perceptions and		"I think it's exactly like flu.	Cooper et al.,

296

Top level Theme	Theme	Sub-theme	Quote	Citation
Validation & Knowledge (awareness, legitimacy, expectations)	experiences of LC and its management, including rehabilitation		The same applies in flu. You get lots of people that get it. Most people are not terribly well with it, few people get flu without knowing they've had it. Some people recover quickly, some take a longer time to recover and some die" [AGP04]	2024
Recognition, Validation & Knowledge (awareness, legitimacy, expectations)	Perceptions and experiences of LC and its management, including rehabilitation		"Physio, I can't really see much of a role. But that could be my lack of knowledge about it because with the patients I have spoken to it's not really so much of a physical thing, it's not like a particular joint pain as such that they would benefit from a physio. It's more the kind of cognitive aspect, maybe an OT, but I don't know what they would add." [BGP05]	Cooper et al., 2024
Recognition, Validation & Knowledge (awareness, legitimacy,	Perceptions and experiences of LC and its management, including rehabilitation		"The biggest help was speaking to Speech and Language… she gave me lots of information that was very interesting, and lots about the	Cooper et al., 2024

297

Top level Theme	Theme	Sub-theme	Quote	Citation
expectations)			biology of what's going on with my [laryngeal] spasms." [DPwLC04]	
Recognition, Validation & Knowledge (awareness, legitimacy, expectations)	Perceptions and experiences of LC and its management, including rehabilitation		"Just having somebody to help you manage what that should look like, what is too much, because you can read about pacing, you can chat about it online with other people with Long COVID, but trying to get a model that fits for you as an individual is actually really hard without support." [DPwLC06]	Cooper et al., 2024
Recognition, Validation & Knowledge (awareness, legitimacy, expectations)	Perceptions and experiences of LC and its management, including rehabilitation		"When I did refer myself to the [specialist] team, I got a booklet, a massive booklet through the post, that says this that and the other. But it's such an individual, highly differentiated set of symptoms that any one person can have, just none of it was particularly relevant to me." [DPwLC01]	Cooper et al., 2024
Recognition,	Perceptions and		"Just with having this	Cooper et al.,

298

Top level Theme	Theme	Sub-theme	Quote	Citation
Validation & Knowledge (awareness, legitimacy, expectations)	experiences of LC and its management, including rehabilitation		discussion it's like a wee light bulb moment that I'm having, that I'm thinking I've tried the PT [personal training], it was too intense, threw the towel in." [DPwLC01]	2024
Recognition, Validation & Knowledge (awareness, legitimacy, expectations)	Perceptions and experiences of LC and its management, including rehabilitation		"I think they feel quite isolated actually and I think it would be useful even if objectively…there's not a huge improvement. I think psychologically it would be really important for them. Someone to believe them, to see what's happening, and just thinking someone's looking out for them." [BGP01]	Cooper et al., 2024
	Key Theme	Subtheme	Quote + Participant ID	Source
Access & Entry to Care (GP gatekeeping, referrals, waiting lists)	Accessing care for people with Long COVID	PwLC accessing general practitioner (GP) services	"You don't get face-to-face with the GP. So again, when you get a GP, it's always a different GP you're talking to and you try your best, but you get locum, and they've all been fantastic. I'm not, again,	Duncan et al., 2023

299

Top level Theme	Theme	Sub-theme	Quote	Citation
			I wouldn't criticise them, but it’s exhausting telling the same story every single time.” (P3208)	
Access & Entry to Care (GP gatekeeping, referrals, waiting lists)	Accessing care for people with Long COVID	Awareness of services by GPs and PwLC	“[We] need to get past the public’s perception that GPs are shut so they return to their GP and get a referral to their service if required.” (L3102)	Duncan et al., 2023
Access & Entry to Care (GP gatekeeping, referrals, waiting lists)	Accessing care for people with Long COVID	Waiting lists and waiting times	“They [HB4 Long COVID service] were basically inundated. So, I had to wait, about four months or so, something like that for a slot. But it was quicker than I thought, but they were just getting overwhelmed with the workload.” (P4101)	Duncan et al., 2023
Access & Entry to Care (GP gatekeeping, referrals, waiting lists)	Accessing care for people with Long COVID	Awareness of services by GPs and PwLC	“Yeah. So, it’s [integrated service] not a widely publicised thing, because I don’t know if we could cope as a service with the numbers.” (S1102)	Duncan et al., 2023
Access & Entry to Care (GP	Accessing care for people with Long	Waiting lists and waiting times	“The referral side of it they probably done all the right	Duncan et al., 2023

300

Top level Theme	Theme	Sub-theme	Quote	Citation
gatekeeping, referrals, waiting lists)	COVID		things, but the timescales were just probably horrific but understandable. I know that in hindsight now so, but it didn't take away the frustrations of it." (P3208)	
Access & Entry to Care (GP gatekeeping, referrals, waiting lists)	Accessing care for people with Long COVID	Waiting lists and waiting times	"Difficult due to capacity. Everywhere so busy and up against it. The pressures on us are really extreme." (L3101)	Duncan et al., 2023
Access & Entry to Care (GP gatekeeping, referrals, waiting lists)	Accessing care for people with Long COVID	Self-referral pathway challenges	"I don't have self-referral at the moment. And the referrals coming through the GP or through something, some of them are coming through. But I don't know where all the others are, so, I'm not blocking access, but they are getting stuck in the system, in my system, in that if I don't have enough information on the referral, they're just going on to the normal routine waiting list." (S2207)	Duncan et al., 2023
Access & Entry	Accessing care for	Lack of medical staff /	"But it's those that have the	Duncan et al.,

301

Top level Theme	Theme	Sub-theme	Quote	Citation
to Care (GP gatekeeping, referrals, waiting lists)	people with Long COVID	pathway clarity	more complex needs that I wouldn't know where to send them to." (S2103)	2023
Recognition, Validation & Knowledge (awareness, legitimacy, expectations)	Understanding Long COVID and its management	Reluctance to diagnose / managing symptoms	"It's quite difficult and just feel like, you know, you have a list as long as your arm when you actually speak to the GP because some things have changed and you know they're very sympathetic, but very clear and honest that well, we don't really know, you know? We just don't know. So, we'll give you this pill to try and treat this symptom at the moment and, that's quite hard." (P4101)	Duncan et al., 2023
Recognition, Validation & Knowledge (awareness, legitimacy, expectations)	Understanding Long COVID and its management	Reluctance to diagnose	"What are we shaming people for having a condition? What's that all about? It's very bizarre. You get them help. I don't understand. It's like invisible illnesses." (L1202)	Duncan et al., 2023
Recognition,	Understanding Long	Reluctance to diagnose	"It was really difficult	Duncan et al.,

302

Top level Theme	Theme	Sub-theme	Quote	Citation
Validation & Knowledge (awareness, legitimacy, expectations)	COVID and its management		actually to know whether to adopt the term Long COVID 'cause it's not a medical term, but we've decided in the end it's probably got wider recognition, now, that's what the patients are using. But having to be very careful, actually even when you're writing letters to GPs, you know if they didn't have confirmed COVID in the beginning, it's a presumed COVID illness, you know symptoms consistent with Long COVID, you know. It's a lot of kind of working around the houses I feel." (S4101)	2023
Recognition, Validation & Knowledge (awareness, legitimacy, expectations)	Understanding Long COVID and its management	Being believed / validation	"It felt to me like it was a bunch of very random symptoms. It didn't seem connected, and I think probably one of the best things in that first conversation was just hearing that the symptoms I have	Duncan et al., 2023

303

Top level Theme	Theme	Sub-theme	Quote	Citation
			were very common and they are all related.” (P4102)	
Recognition, Validation & Knowledge (awareness, legitimacy, expectations)	Understanding Long COVID and its management	Being believed / validation	“I get the impression that a lot of my symptoms are replicated a lot across, across other people so and I’m getting a lot of assurance on what people’s progress like recovery-wise again its always pinned by keep your expectations to a minimum, like it’s no [not] going to be the miracle cure.” (P3208)	Duncan et al., 2023
Recognition, Validation & Knowledge (awareness, legitimacy, expectations)	Understanding Long COVID and its management	Evolving evidence base / treatment caution	“So, what I was concerned about was what if I give the wrong advice or the wrong sort of exercise prescription, and that I actually cause harm to him by something that I've done. And I watched some of the podcasts to sort of understand what we should and shouldn't be doing, and that is when I started to realize that there is a really strong link with Long COVID and the chronic	Duncan et al., 2023

304

Top level Theme	Theme	Sub-theme	Quote	Citation
			fatigue syndrome and ME population who have been saying for years and years that, actually, sometimes, graded exercise therapy is really harmful, and you absolutely should not prescribe it." (S2104)	
Recognition, Validation & Knowledge (awareness, legitimacy, expectations)	Understanding Long COVID and its management	Education and professional networking	"There's certainly discussions around what is the best way to approach management of Long COVID and there's been a few things we can do, though, because nationally there's a huge focus on physiotherapy management of Long COVID you know, the CSP [Chartered Society of Physiotherapists], I've got lots of stuff out there, but we've also got the clinicians and the COVID rehab team there that although they're not seeing patients and more than happy to signpost people to resources and help with a bit of professional advice. I	Duncan et al., 2023

305

Top level Theme	Theme	Sub-theme	Quote	Citation
			would say from a Community hub, clinician point of view, confidence hasn't been the main issue. They do seek professional advice on how to manage them.” (S3203)	
	Long COVID services — Strengths	Self-management education benefits	“I’m certainly more educated, already daily life has improved I’m not making myself ill doing things I didn’t know were doing that.” (P3203)	Duncan et al., 2023
	Long COVID services — Strengths	Being believed / validation	“…the relief to at that point to speak to a professional who was actually acknowledging that this was happening and you know, it wasn't all in my head. I don't know there, for months and months and months I think it was common there was just no-one apart from my GP, there was just no of recognition or acknowledgement of what people were going through or anything like that. So, it was just such a relief to speak to	Duncan et al., 2023

306

Top level Theme	Theme	Sub-theme	Quote	Citation
			somebody who was just genuinely interested and very supportive.” (P4107)	
	Long COVID services — Strengths	MDT coordination	“It was my GP that referred me to the psychologist then obviously the two of them were interacting so that the [COVID team] were speaking to the psychologist as well and just basically building a care package for me, so it was tailored to what I was needing physically and mentally, so they were quite good actually talking to each other as well, which was I suppose positive.” (P4105)	Duncan et al., 2023
	Long COVID services — Strengths	Cross-board professional networking	“England has been incredibly helpful in giving and sharing lots and…you know… even within the LOCO-RISE study that’s been a really helpful kind of network and relationship to support.” (L3203)	Duncan et al., 2023
	Long COVID	Digital delivery benefits	“Do you know, I think the use	Duncan et al.,

307

Top level Theme	Theme	Sub-theme	Quote	Citation
	services — Strengths		of the increased use of digital services has been fantastic. It’s meant that we've been able to reach people when we wouldn't have normally been able to reach them. It has to be a blended model. It has to be that those people that need face-to-face intervention get it at a time when it’s right. But digital advances have really made a big difference for us.” (L1101)	2023
Resource, Capacity & Sustainability Constraints (funding, staffing)	Long COVID services — Limitations	Funding and staffing constraints	“I think we're struggling even staffing wise with. We can't staff wards properly. We can't. You know all these things it’s. How will we staff a Long COVID service? How will we? How? How will we best provide that service? The NHS way has always been to kind of just absorb it into existing services.” (S1202)	Duncan et al., 2023
Resource, Capacity & Sustainability	Long COVID services — Limitations	Service development without evidence base	“I suppose the other challenge from my point of view is actually just setting up a new	Duncan et al., 2023

308

Top level Theme	Theme	Sub-theme	Quote	Citation
Constraints (funding, staffing)			service from scratch, so you know that's a big job in itself and setting up without the evidence base without kind of any benchmark you know to work with. And so, trying to do that alongside learning about a new disease you know with information coming out constantly." (S4101)	
Resource, Capacity & Sustainability Constraints (funding, staffing)	Long COVID services — Limitations	Broad symptom complexity	"Hardest thing—so many different symptoms that you can have with Long COVID." (S2204)	Duncan et al., 2023
Resource, Capacity & Sustainability Constraints (funding, staffing)	Long COVID services — Limitations	Lack of medical input	"Having the right staff, having medical support in the team, that's been a big gap I feel, and that's still a gap and I think that's really important given the complexity of patients coming through. We're doing a lot of 'safety netting' as AHP's at the moment, but I don't think,	Duncan et al., 2023

309

Top level Theme	Theme	Sub-theme	Quote	Citation
			you know, that that's our job, necessarily. We've had to take on some of that." (S4101)	
	Theme	Subtheme	Quote & Participant ID	Source
Recognition, Validation & Knowledge (awareness, legitimacy, expectations)	Importance of Patient/Provider Knowledge for Long COVID Recognition	—	"When we had all our education on this, that wasn't really part of the education… searching for long COVID." (Primary care provider – Interview 8)	Horlick et al., 2023
		—	"(Patients) are not getting the support from anywhere else; a lot of them are really shocked when they talk to their GPs (primary care physician) and the GP isn't aware that [specialty clinic] is here." (Rehabilitation care provider – Interview 1)	Horlick et al., 2023
		—	"When I was reading… (experts) were talking about 200 symptoms of long COVID, I don't know all 200 symptoms. That just blew me away… oh my gosh, we don't ask 200 symptoms on our	Horlick et al., 2023

310

Top level Theme	Theme	Sub-theme	Quote	Citation
			(long COVID) questionnaire.” (Rehabilitation care provider – Interview 1)	
		—	“Some of the patients that do come to us, they tell us that they read about a case in the newspaper and then they went and told their doctor.” (Specialty care provider – Interview 6)	Horlick et al., 2023
		—	“My clinical assessments have certainly evolved since being in this since January. Just asking more questions, being more astute… in the long COVID world, it’s things that they’re saying and it’s like let’s move over there and talk about that. So, I think that’s just me evolving in my assessment skills.” (Specialty care provider – Interview 9)	Horlick et al., 2023
Recognition, Validation &	The Role of Patient/Provider	Disbelief in COVID or Long COVID	“(Patients) are not getting the support from anywhere else.	Horlick et al., 2023

311

Top level Theme	Theme	Sub-theme	Quote	Citation
Knowledge (awareness, legitimacy, expectations)	Acknowledgement of Long COVID		(I) Just feel that a lot of our (patients), especially the long-haul ones, what we're calling long COVID, when they're 12, 14, 16 weeks out. They're just finding there's not a lot of buy-in, from friends, from family, from employers… We're finding there's not a lot of buy-in from even our medical community, right now." (Rehabilitation care provider – Interview 1)	
			"It's not the patient's fault because they don't know what's available to them… And when they do ask for help, I have heard 'my physician doesn't believe me, they don't believe in COVID, they don't believe I'm experiencing these symptoms, and they don't know where to send me if I'm experiencing these symptoms'." (Specialty care provider – Interview 6)	Horlick et al., 2023

312

Top level Theme	Theme	Sub-theme	Quote	Citation
Recognition, Validation & Knowledge (awareness, legitimacy, expectations)		Acknowledgement as Part of Care	“I think another advantage (of long COVID care) is, the patients feel supported and that they’re not losing their mind, and it’s all in their head. They’re like… I still feel (bad) and nobody knows, we don’t know ‘cause this is new. So I think that’s a big plus, a big advantage for the patients to feel that, just feel supported.” (Primary care provider – Interview 8)	Horlick et al., 2023
			“I think the support piece has been really big (at our site) and that’s what we hear in feedback is just how grateful they are that somebody is here to listen and believes them.” (Rehabilitation care provider – Interview 1)	Horlick et al., 2023
			“Most of my patients have talked to… their primary care provider about their symptoms and they’re told that long COVID is a problem and… they could be	Horlick et al., 2023

313

Top level Theme	Theme	Sub-theme	Quote	Citation
			experiencing those symptoms, but that… seems to be where the conversation ends… primarily patients… are saying that… they talked to their provider about their symptoms, but they don't know what else to do… And (are) not provided with those additional resources." (Specialty care provider – Interview 7)	
Self-management, Psychoeducation & Peer Support	Developing Recovery Expectations	—	"I can only think of one (patient) in particular off the top of my head that did not find the resources helpful, and she was really looking for much more specific (steps)… Some conversation… and some understanding of really what's appropriate for treatment was helpful for her." (Specialty care provider – Interview 7)	Horlick et al., 2023
		—	"I impart on my patients that I don't have a pill that I can give you that's going to get	Horlick et al., 2023

314

Top level Theme	Theme	Sub-theme	Quote	Citation
			rid of your post COVID symptoms. I have some medications that can help manage some of the symptoms that you may be experiencing, but overall, you've got to put in the work, I'll put in the work with you, but you've got to put in the work yourself and here are the tools I'm handing to you." (Specialty care provider – Interview 6)	
Access & Entry to Care (GP gatekeeping, referrals, waiting lists)	Navigation and Wayfinding	Poor Integration with Social and Mental Health Services	"I would love for us to have… some kind of mental health resource—so either a psychologist or a psychiatrist to refer our patients to because a lot of our patients have mental health needs that were either borderline or nonexistent before COVID, and after their COVID illness have significantly impacted them. And so, when we get one of those, where do you go from here?" (Specialty care	Horlick et al., 2023

315

Top level Theme	Theme	Sub-theme	Quote	Citation
			provider – Interview 6)	
Access & Entry to Care (GP gatekeeping, referrals, waiting lists)		Wait Times/Referrals	"Some of those people who don't connect with a family doctor, fall through the cracks. It delays referrals—that kind of thing, and potentially leads to duplication. So I think that's an issue, is maybe needing that referral from the family doc for people who don't have it." (Rehabilitation care provider – Interview 2)	Horlick et al., 2023
			"I would say there's potential (for patients to access long COVID health services), but I think the wait list is so long and depending on people's needs, like in a timely manner I would say no… and that is certainly a challenge for us, is it's just backlogged." (Rehabilitation care provider – Interview 2)	Horlick et al., 2023

316

Top level Theme	Theme	Sub-theme	Quote	Citation

317
